# Integrative Transcriptome‐Wide Association Study With Expression Quantitative Trait Loci Colocalization Identifies a Causal VAMP8 Variant for Nasopharyngeal Carcinoma Susceptibility

**DOI:** 10.1002/advs.202412580

**Published:** 2025-01-24

**Authors:** Yan Liang, Xiang‐Yu Xiong, Guo‐Wang Lin, Xiaomeng Bai, Fugui Li, Josephine Mun‐Yee Ko, Yun‐He Zhou, An‐Yi Xu, Shu‐Qiang Liu, Shuai He, Pan‐Pan Wei, Qiu‐Yan Chen, Lin‐Quan Tang, Vivien Ya‐Fan Wang, Hai‐Qiang Mai, Chun‐Ling Luo, Yanni Zeng, Maria Li Lung, Mingfang Ji, Jin‐Xin Bei

**Affiliations:** ^1^ State Key Laboratory of Oncology in South China Guangdong Key Laboratory of Nasopharyngeal Carcinoma Diagnosis and Therapy Guangdong Provincial Clinical Research Center for Cancer Sun Yat‐sen University Cancer Center Sun Yat‐sen University Guangzhou 510060 P. R. China; ^2^ Department of Experimental Research Sun Yat‐sen University Cancer Center Guangzhou 510060 P. R. China; ^3^ Department of Laboratory Medicine Zhujiang Hospital Southern Medical University Guangzhou Guangdong 510280 P. R. China; ^4^ Faculty of Forensic Medicine Guangdong Province Translational Forensic Medicine Engineering Technology Research Center Zhongshan School of Medicine Sun Yat‐sen University Guangzhou 510080 P. R. China; ^5^ Guangdong Province Key Laboratory of Brain Function and Disease Zhongshan School of Medicine Sun Yat‐sen University Guangzhou 510080 P. R. China; ^6^ Cancer Research Institute of Zhongshan City Zhongshan City People's Hospital Zhongshan 528403 P. R. China; ^7^ Department of Clinical Oncology School of Clinical Medicine University of Hong Kong Hong Kong SAR 999077 P. R. China; ^8^ Faculty of Health Sciences University of Macau Avenida da Universidade Taipa Macau SAR 999078 P. R. China; ^9^ Sun Yat‐sen University Institute of Advanced Studies Hong Kong Science Park Hong Kong SAR 999077 P. R. China; ^10^ Department of Medical Oncology National Cancer Centre Singapore Singapore 169610 Singapore

**Keywords:** expression quantitative trait loci (eQTL), genome‐wide association study (GWAS), nasopharyngeal carcinoma (NPC), transcriptome‐wide association study (TWAS), vesicle‐associated membrane protein 8 (VAMP8)

## Abstract

Nasopharyngeal carcinoma (NPC) is an Asia‐prevalent malignancy, yet its genetic underpinnings remain incompletely understood. Here, a transcriptome‐wide association study (TWAS) is conducted on NPC, leveraging gene expression prediction models based on epithelial tissues and genome‐wide association study (GWAS) summary statistics from 1577 NPC cases and 6359 controls of southern Chinese descent. The TWAS identifies *VAMP8* on chromosome 2p11.2 as a novel susceptibility gene for NPC. Further fine‐mapping analyses pinpoint rs1058588, located within *VAMP8*, as a causal variant through eQTL colocalization, and GWAS analyses across multiple cohorts, achieving GWAS significance (OR = 1.18, *P* = 3.09 × 10^−10^). Functional assays demonstrate that VAMP8 exerts a tumorigenic role in NPC, enhancing cell proliferation, migration, and tumor growth. Mechanically, it is uncovered that rs1058588 modulates *VAMP8* expression by altering its binding affinity to *miR‐185*. Furthermore, the results show that VAMP8 interacts with DHX9 to facilitate the nuclear recruitment of p65, activating the NF‐κB pathway. Collectively, the findings shed light on the genetic predisposition to NPC and underscore the critical role of the functional axis involving miR‐185, VAMP8, DHX9, and the NF‐κB pathway in NPC pathogenesis.

## Graphical abstract

Integrating transcriptome‐wide association study, expression quantitative trait loci, and genome‐wide association study identifies rs1058588 at the vesicle‐associated membrane protein 8 (VAMP8) locus as a risk variant for nasopharyngeal carcinoma (NPC). The rs1058588‐C allele reduces miR‐185 binding to VAMP8 3'‐untranslated region, upregulating VAMP8 and promoting tumorigenesis through DHX9‐mediated NF‐κB activation, increasing NPC risk.

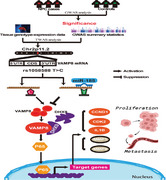

## 1 Introduction

Nasopharyngeal carcinoma (NPC), a distinct type of head and neck malignancy originating from the nasopharyngeal epithelium, exhibits a unique geographical distribution pattern.^[^ ^1^ ^]^ It is notably prevalent in Eastern and Southeast Asia, where its incidence rates range between 25 and 50 per 100 000 individuals, markedly higher than those observed in western countries.^[^ ^2^ ^]^ Familial clustering of NPC across diverse populations points to a genetic predisposition to the disease.^[^ ^3^ ^]^ Multiple genome‐wide association studies (GWASs) have identified several genetic loci associated with NPC risk.^[^ ^4^, ^5^ ^]^ However, these loci collectively account for only a small portion of the overall genetic variance underlying NPC susceptibility, suggesting the involvement of additional unidentified genes or factors contributing to the so‐called “missing heritability.”^[^ ^6^, ^7^ ^]^ Additionally, most of these disease‐associated genetic markers locate within noncoding genomic regions, putting substantial obstacles toward understanding their biological effects and relevance to NPC pathogenesis.

Transcriptome‐wide association study (TWAS) offers a strategic advantage in identifying gene‐trait associations, potentially addressing the issue of missing heritability by considering the cumulative effects of multiple genetic variants on functioning gene expression.^[^ ^8^, ^9^ ^]^ This approach has recently led to the discovery of associations between multiple MHC genes and NPC risk.^[^ ^10^ ^]^ However, this study estimated the expression of MHC genes for individuals using both genotype and expression data derived from Epstein–Barr virus (EBV)‐transformed lymphocytes, which might not fully capture NPC‐specific transcriptional regulatory mechanisms. This limitation stems from two main reasons. First, NPC originates from the malignant transformation of epithelial cells, not lymphocytes, suggesting a different cellular origin and distinct regulatory pathways. Second, the EBV‐transformed lymphocytes may not accurately represent in vivo regulatory dynamics due to the absence of tumor microenvironment (TME), where interactions among TME components are essential for NPC development.^[^ ^11^ ^]^

To identify novel susceptibility genes of NPC, we conducted a TWAS, leveraging gene expression prediction models derived from esophagus mucosa samples and GWAS summary statistics for NPC. The selection of esophagus mucosa samples as the source for gene expression models is strategically aligned with the anatomical proximity of the esophagus to the nasopharynx and the shared predominance of epithelial cells, making it highly relevant to NPC pathogenesis. Through this innovative approach, we identified *VAMP8* as a novel gene associated with NPC susceptibility. We further fine mapped the causal variant of *VAMP8* through extensive bioinformatics analyses and multiple sample collections. Our study also delved into the biological role of VAMP8, its regulatory mechanisms, and the impact on NPC tumorigenesis. This comprehensive study not only sheds light on the specific genetic factors contributing to NPC but also enhances our understanding of the complex mechanisms through which genetic variations can influence the etiology of NPC.

## 2 Results

### 2.1 TWAS Identifies *VAMP8* Association with NPC Susceptibility

To identify genes implicated in NPC risk, we conducted a TWAS using gene expression predictive models based on genotypes from the GTEx esophagus mucosa panel. These models were then applied to 1577 NPC cases and 6359 controls, integrating GWAS summary statistics generated from a SNP‐based GWAS in NPC, using MetaXcan and FUSION algorithms (see the Experimental Section; and Figures S1 and S2, Supporting Information). Our TWAS identified 21 genes with expression levels significantly associated with NPC risk, surpassing the threshold for transcriptome‐wide significance after Bonferroni correction (*p* ≤ 6.49 × 10^−6^ for MetaXcan and *p* ≤ 7.54 × 10^−6^ for FUSION **Table** **1**; and; Figure S3, Tables S1 and S2, Supporting Information). Intriguingly, five of these genes, located at 2p11.2 and over 1 Mb away from any previously reported GWAS risk loci, were considered novel findings (Table 1). The remaining 16 genes were located within the known GWAS loci, including 13 in the MHC region (Table S1, Supporting Information) and three in the 5p15.33 and 6p22.1 regions (Table S2, Supporting Information).

**Table 1:** Novel associations between gene expression and NPC risk.

Gene	Chr	Start	End	Type^a)^	Model^b)^	Top eQTL SNP^c)^	TWAS Z^d)^	TWAS P^e^ ^)^
TCF7L1	2	85 360 515	85 537 510	protein_coding	GTEx.Esophagus_Mucosa‐MetaXcan	—	−5.01	5.42 × 10^−7^
GGCX	2	85 771 843	85 788 670	protein_coding	GTEx.Esophagus_Mucosa‐FUSION	rs6757263	−5.01	5.28 × 10^−7^
VAMP8	2	85 788 685	85 809 154	protein_coding	GTEx.Esophagus_Mucosa‐MetaXcan	—	5.36	8.24 × 10^−8^
					GTEx.Esophagus_Mucosa‐FUSION	rs1058588	5.50	3.60 × 10^−8^
RNF181	2	85 822 848	85 824 831	protein_coding	GTEx.Esophagus_Mucosa‐MetaXcan	—	5.71	1.10 × 10^−8^
					GTEx.Esophagus_Mucosa‐FUSION	rs6705971	4.61	4.01 × 10^−6^
TMEM150A	2	85 825 670	85 830 319	protein_coding	GTEx.Esophagus_Mucosa‐MetaXcan	—	5.48	4.28 × 10^−8^

^a)^ Risk variants are located at least 1 Mb outside of any loci reported in previous GWASs or fine‐mapping studies (ref: 4−5);

^b)^ The models were based on the GTEx esophageal mucosa tissue dataset and MetaXcan or FUSION software;

^c)^ Variants most significantly associated with gene expression according to the FUSION results; ‐: not available from the MetaXcan results;

^d)^ Direction and effect size. Positive and negative values suggest associations of upregulated and downregulated predicted gene expression with increased NPC risk, respectively;

^e)^ Significant association *p* values. The significance thresholds are set as *p* ≤ 6.49 × 10^−6^ and *p* ≤ 7.54 × 10^−6^ for a Bonferroni correction of 8597 tests (0.05/7709) in MetaXcan and 6631 tests (0.05/6631) in FUSION, respectively.

**Figure 1:**
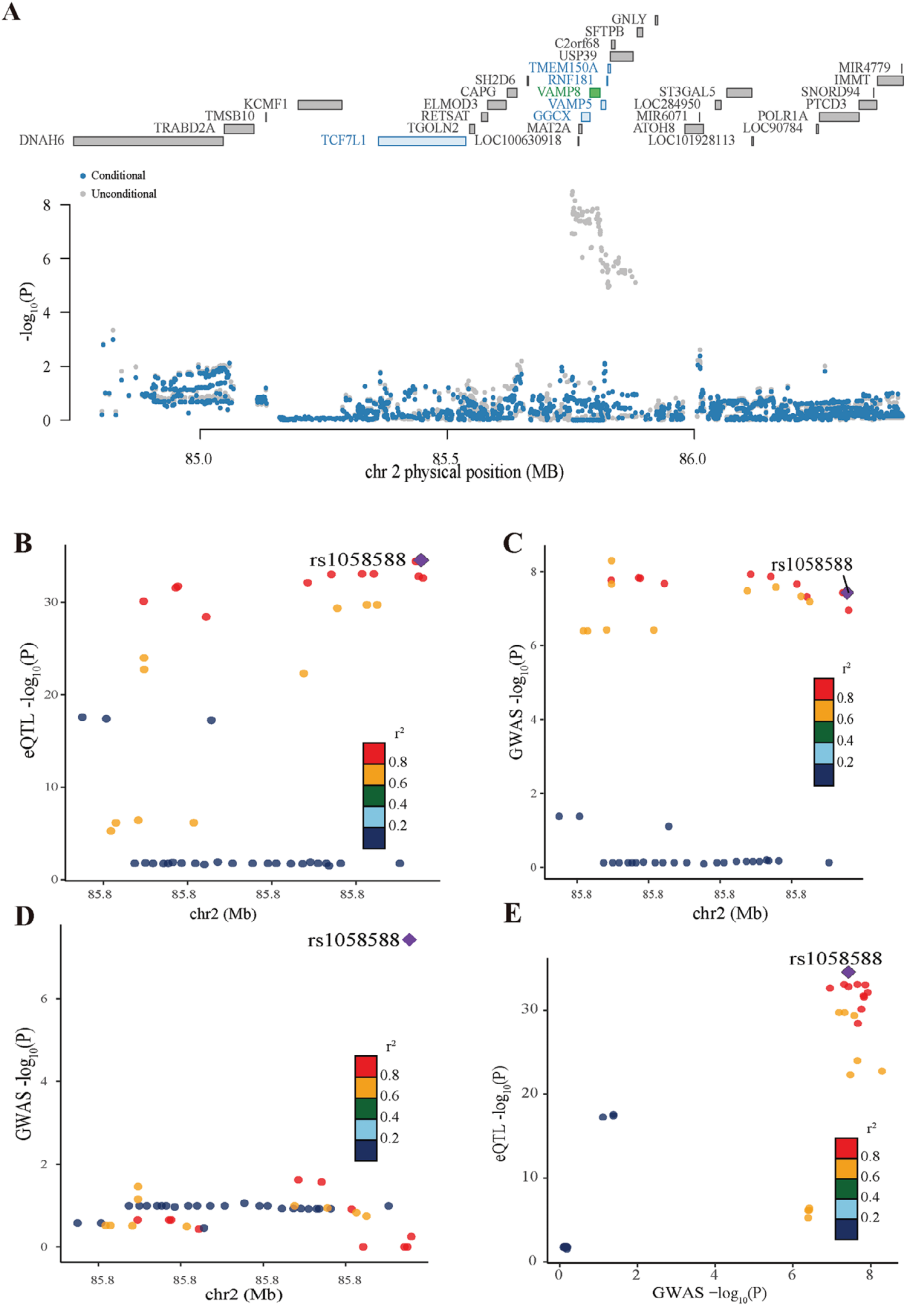
Association results in the VAMP8 locus. A) Regional Manhattan plot of the genome‐wide association study *p* values before (in gray) and after (blue) conditioning on the predicted expression of VAMP8. Each dot represents one SNP. *x*‐ and *y*‐axes present the genomic location and −log(10) *p* values, respectively. Genes in the region are indicated on top. VAMP8 with TWAS significance is highlighted in green. Nonsignificant TWAS genes after conditioning VAMP8 are highlighted in blue. Remaining genes without any significant TWAS signals are highlighted in gray. B) Scatterplot showing the eQTL associations at VAMP8 region. The *y*‐axis represents esophagus mucosa eQTL *P*‐value in −log10 scale for VAMP8. The *x*‐axis represents base pair position on chromosome 2. The extent of linkage disequilibrium for all SNPs with rs1058588 (diamond) is color‐coded as indicated. C,D) Scatterplot showing the GWAS associations at VAMP8 region before C) and after D) controlling the genetic effect of rs1058588. The *y*‐axis represents GWAS *P*‐value in −log10 scale at the VAMP8 locus. The *x*‐axis represents base pair position on chromosome 2. The extent of linkage disequilibrium for all SNPs with rs1058588 (diamond) is indicated by colors. E) Colocalization of the eQTL and GWAS associations at the VAMP8 locus. The *y*‐axis represents esophagus mucosa eQTL *P*‐value in −log10 scale for VAMP8. The *x*‐axis represents GWAS *P*‐value in −log10 scale at the VAMP8 locus.

In the 2p11.2 region, our study revealed that higher expression levels of *VAMP8*, *RNF181*, and *TMEM150A*, alongside lower expression of *TCF7L1* and *GGCX*, were associated with increased NPC risk. Correlation analyses showed a significant coexpression among these five genes in either GTEx esophagus mucosa (*n* = 444) or NPC samples (*n* = 206; see the Experimental Section; and Table S3, Supporting Information), suggesting a potential coregulatory mechanism influencing these multiple TWAS signals observed at 2p11.2. To investigate the independence of these TWAS signals, we performed summary‐based conditional analysis in this region using GTEx esophagus mucosa models implemented in the FUSION. Remarkably, upon controlling for the predicted expression of *VAMP8*, the associations of other candidate genes and relevant SNPs, as indicated by TWAS and GWAS signals, respectively, were no longer significant (*p* > 0.05; **Figure** **1**A; and Tables S4–S5, Supporting Information). These results suggest *VAMP8* as a pivotal gene at this locus, driving the association with NPC risk.

To further validate the association of *VAMP8* expression with NPC risk, we extended our analysis by conducting additional TWASs using three alternative gene expression prediction models, including NPC‐MetaXcan, NPC‐FUSION, and TCGA.HNSC‐FUSION (Table S6, Supporting Information). Analyses based on NPC‐MetaXcan or TCGA.HNSC‐FUSION consistently showed a significant correlation between upregulated *VAMP8* expression and increased NPC risk, maintaining statistical significance after Bonferroni‐correction (NPC‐MetaXcan: *Z* = 5.62, *P* = 1.86 × 10^−8^, Table S7 (Supporting Information); TCGA.HNSC‐FUSION: *Z* = 5.64, *P* = 1.66 × 10^−8^, Table S8, Supporting Information). Additionally, *VAMP8* was untestable in the NPC‐FUSION models due to an insignificant estimate of cis‐SNP heritability of gene expression implemented in GCTA (*p* = 0.207), despite its reliable prediction performance in other models (Table S6, Supporting Information). Gene‐based analysis using the MAGMA algorithm and GWAS summary statistics confirmed the significant link between *VAMP8* and NPC risk (Z = 5.57, *P* = 9.42 × 10^−8^). Comparative analysis also revealed higher *VAMP8* expression in NPC samples compared with rhinitis controls (Figure S4A, Supporting Information), and its upregulation was associated with poorer prognosis in NPC (Figure S4B,C, Supporting Information). Collectively, these findings corroborate the critical role of *VAMP8* in NPC development.

### 2.2 Identification of Causal Variant(s) at the *VAMP8* Locus Associated with NPC

**Figure 2:**
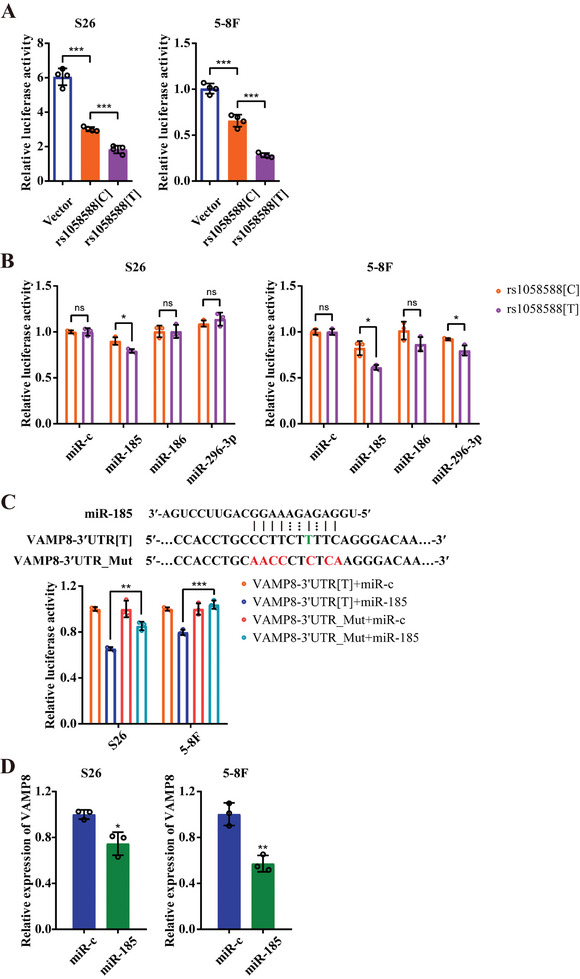
rs1058588‐[T] modulates VAMP8 expression via miR‐185 interaction. A) Dual‐luciferase reporter assay was performed in NPC cell lines S26 and 5–8F to reveal the regulatory role of rs1058588. These cells were transfected with the psiCHECK2 vector or constructs with either C or T allele at rs1058588 in VAMP8 3′‐UTR region. B) Dual‐luciferase reporter assay showed relative luciferase activity in NPC cells. S26 and 5–8F cells were cotransfected with the VAMP8‐3′‐UTR‐psiCHECK2 constructs containing rs1058588‐[T] or rs1058588‐[C] allele and miRNA mimics or scrambled miRNAs. The relative luciferase activity was normalized to scrambled miRNAs (miR‐c). C) Dual‐reporter assay reveals the mediation of rs1058588 on the binding of miR‐185 to the 3′‐UTR of VAMP8. The upper panel displayed the sequence for miR‐185 (top), the predicted targeting site of miR‐185 on the 3′‐UTR of VAMP8 (middle; rs1058588‐[T] was highlighted in green), and a negative control with seven mutations (bottom; the mutation sites were highlighted in red). S26 and 5–8F cells were cotransfected with the VAMP8‐3′UTR[T] or VAMP8‐3′UTR_Mut constructs and miR‐185 or miR‐c mimics. The relative luciferase activity was measured and normalized to VAMP8‐3′UTR[T]+miR‐c group (bottom panel). D) The mRNA expression level of VAMP8 in NPC cells influenced by miRNAs. S26 and 5–8F cells were transfected with miR‐185 or miR‐c mimics and VAMP8 mRNA was measured by using qRT‐PCR analysis. ^*^ *p* < 0.05, ^**^ *p* < 0.01, and ^***^ *p* < 0.001. All statistical analyses were performed using two‐tailed Student's *t*‐test.

GTEx survey pinpointed rs1058588 as the SNP most significantly associated with *VAMP8* expression in esophagus mucosa tissue, standing out among 77 SNPs at the *VAMP8* locus (Figure 1B; and Table S9, Supporting Information). eQTL analysis established a strong link between the rs1058588‐[C] allele and upregulated *VAMP8* mRNA levels (*β* = 0.34, *P* = 2.86 × 10^−35^, Table S9, Supporting Information), suggesting a cis‐eQTL effect of rs1058588 on *VAMP8* expression. Luciferase reporter assays demonstrated that NPC cells expressing the rs1058588‐[C] construct exhibited significantly higher luciferase activity compared to those with the rs1058588‐[T] construct (**Figure** **2**A). Additionally, COJO conditional analysis demonstrated that the TWAS signal for *VAMP8* was completely abolished upon adjusting for rs1058588 (Table S10, Supporting Information). These findings strongly indicate rs1058588 as the key functional variant responsible for eQTL signal at the *VAMP8* locus.

The GWAS identified 17 SNPs within the *VAMP8* region, including rs1058588, significantly associated with NPC risk, surpassing genome‐wide significance (OR = 1.27, 95%CI = 1.17–1.38, *P* = 3.73 × 10^−8^, Figure 1C; and Table S11, Supporting Information). Notably, rs1058588 demonstrated strong linkage disequilibrium (LD) with the other 16 SNPs and adjusting for rs1058588 abolished their associations (Figure 1D; and Table S11, Supporting Information). These findings suggest that rs1058588 is the key variant driving the associations observed at the *VAMP8* locus. To replicate this association, we conducted a SNP‐based association analysis in two additional independent case‐control cohorts from Guangdong (1941 cases and 975 controls; Validation‐1) and Hong Kong (2334 cases and 2507 controls; Validation‐2) in southern China. We observed consistent replicated association of rs1058588 with NPC risk in both cohorts (*p* < 0.05; **Table** **2**). A meta‐analysis of all samples from the discovery and replication stages reaffirmed a strong association between rs1058588 and NPC risk (OR = 1.18, 95% CI = 1.12–1.25, *P* = 3.09 × 10^−10^; Table 2). Collectively, these findings establish rs1058588 as the shared variant underlying both GWAS and eQTL signals at the *VAMP8* locus (Figure 1E), which is further supported by colocalization analysis indicating a strong coassociation of eQTL and GWAS signals (PP4 = 0.977).

**Table 2:** Association results of rs1058588 and NPC susceptibility.

rs1058588 (C>T) (chromosome 2:85 808 871)		Discovery	Validation‐1	Validation‐2	Meta‐analysis^a)^
*N*	Case	1577	1941	2334	5852
	Control	6359	975	2507	9841
Effect C allele frequency	Case	0.65	0.62	0.62	
	Control	0.60	0.59	0.59	
*P*		3.73 × 10^−8^	3.63 × 10^−2^	2.21 × 10^−3^	3.09 × 10^−10^
OR (95% CI)		1.27 (1.17–1.38)	1.12 (1.01–1.25)	1.13 (1.04–1.23)	1.18 (1.12–1.25)
P heterogeneity					0.11

^a)^ Meta‐analysis was conducted via the R package metaphor (v3.8‐1) based on a fixed effects model.

### 2.3 VAMP8 Promotes the Proliferation and Migration of NPC Cells

**Figure 3:**
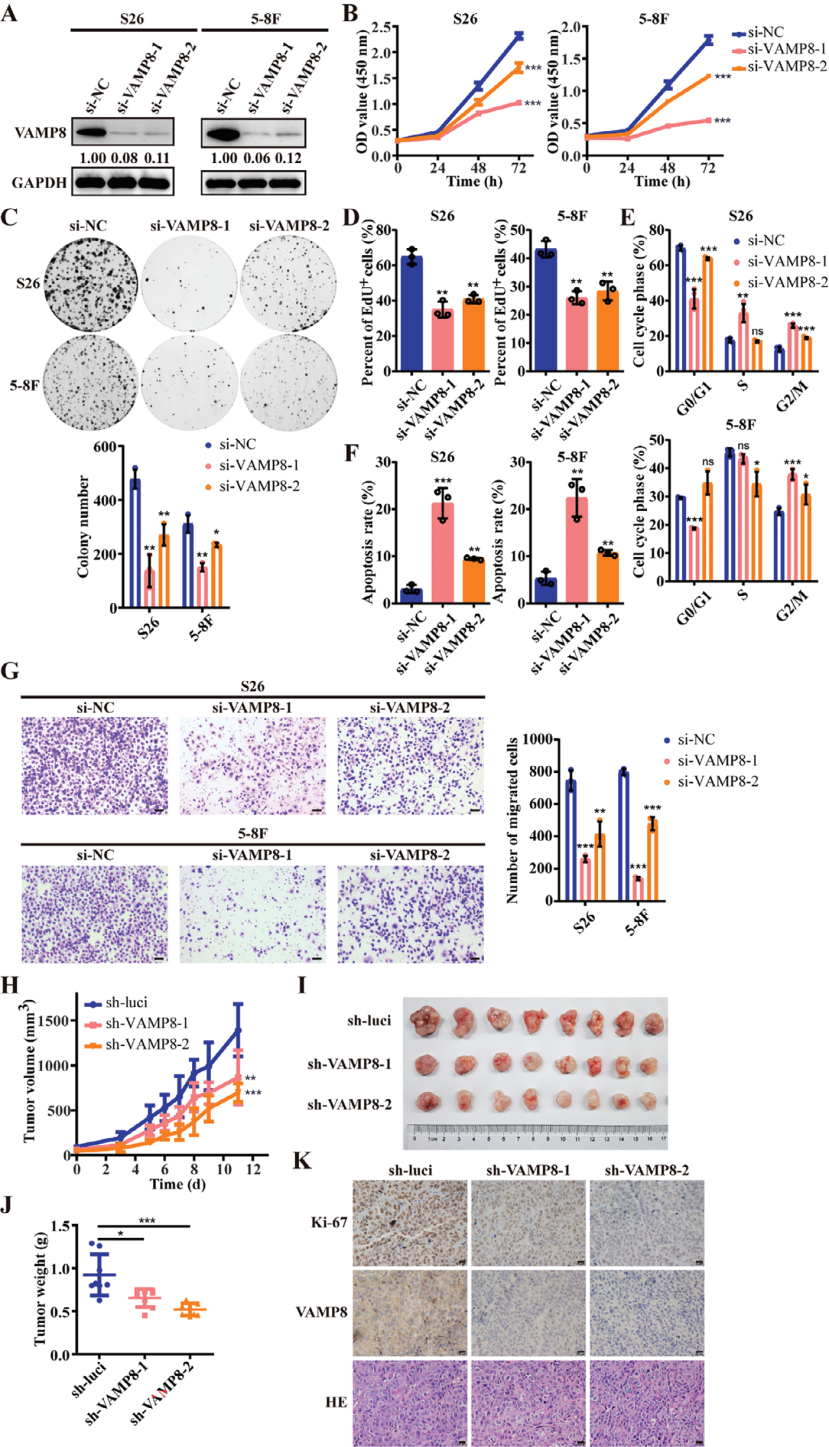
VAMP8 knockdown inhibits the proliferation, migration, and tumorigenesis of NPC cells. A) Western blot assay showed the knockdown effect of VAMP8 on S26 and 5–8F cells transfected with VAMP8 siRNAs or control siRNA. GAPDH was used as control. B) Number of viable cells reflected by the absorbance (450 nm) of CCK8 for the cells described in (A) at 0, 24, 48, and 72 h. C) Representative images of colony formation assay for the cells described in (A). Statistical analysis of colony numbers is shown at the bottom. D) The percentage of EdU staining positive cells was quantified in the cells described in (A). E) Flow cytometry analysis of cell cycle distribution after DNA labeling with propidium iodide in the cells described in (A). F) Flow cytometry analysis of apoptotic cells after staining with Annexin‐V‐FITC and propidium iodide in the cells described in (A). G) Representative images of transwell assay for the cells described in (A), with statistics analysis of migrated cell numbers shown at the right. Scale bar, 100 µm. H) Tumor growth of subcutaneous xenografts derived from S26 cells stably expressing VAMP8 shRNAs or control shRNA. The tumor volume was measured at different time points. I,J) Tumor size I) and tumor weight J) of subcutaneous xenografts dissected from the mice in (H) were shown. K) IHC staining of Ki‐67 and VAMP8 and HE staining of subcutaneous xenografts from (I). Scale bar, 20 µm. ^*^ *p* < 0.05, ^**^ *p* < 0.01, and ^***^ *p* < 0.001. All statistical analyses were performed using two‐tailed Student's *t*‐test.

To investigate the biological functions of VAMP8, we first examined its expression levels across several NPC cell lines (Figure S4D, Supporting Information). Given the higher VAMP8 expression and faster growth rates, S26 and 5–8F cell lines were selected for knockdown assays using siRNAs targeting VAMP8 (**Figure** **3**A). Strikingly, *VAMP8* knockdown led to substantial reductions in the growth and colony formation capabilities of the NPC cells compared to control groups (Figure 3B,C). Fluorescent staining assay, using EdU to mark proliferating cells, showed a remarkable decrease in cell proliferation in the *VAMP8* knockdown groups (Figure 3D; and Figure S5A, Supporting Information). Flow cytometry analysis further revealed an increase in cells arrested at the G2/M phase, accompanied by enhanced apoptosis upon *VAMP8* knockdown (Figure 3E,F; and Figure S5D,E, Supporting Information). These observations suggest that *VAMP8* knockdown suppresses cell proliferation through cell cycle arrest and promoting apoptosis. Additionally, wound healing (Figure S5B, Supporting Information) and transwell assays (Figure 3G) demonstrated a significant decline in NPC cell migration following *VAMP8* knockdown, alongside with a significant upregulation of E‐Cadherin and notable downregulation of other invasion‐promoting molecules, including N‐Cadherin, Vimentin, MMP1, and MMP9 (Figure S5C, Supporting Information). In contrast, *VAMP8* overexpression in NPC cells enhanced tumorigenic properties, as evidenced by increased proliferation (Figure S6A–C, Supporting Information), migration (Figure S6D–F, Supporting Information), and reduced cell cycle arrest at G2/M phase (Figure S6G,H, Supporting Information).

In vivo experiments using xenograft models provided further evidence of VAMP8's role in NPC. Mice in the *VAMP8* knockdown group exhibited significantly slower tumor growth rates and reduced tumor weights compared to the control groups (Figure 3H–J). Additionally, immunostaining for Ki‐67, a marker of proliferating cells, revealed a substantial decrease in proliferating cells in the xenografts derived from *VAMP8* knockdown cells compared to those from control cells (Figure 3K). Taken together, these findings strongly underscore the essential role of VAMP8 in regulating the proliferation and migration of NPC cells.

### 2.4 *miR‐185/VAMP8* Axis Regulates NPC Tumorigenesis

Considering the location of rs1058588 in the 3′‐untranslated region (3′‐UTR) of *VAMP8*, we hypothesized that it regulates *VAMP8* expression through miRNA interactions. A thorough search in the SNPinfo and mirsnpscore databases identified three candidate miRNAs (*miR‐185*, *miR‐186*, and *miR‐296‐3p*) with potential binding sites at the rs1058588 locus. Among these, luciferase reporter assay in NPC cells revealed that only *miR‐185* more substantially inhibited the luciferase activity of the rs1058588‐[T] allele than that of the rs1058588‐[C] allele (Figure 2B), suggesting that the T > C substitution likely diminishes *miR‐185* binding affinity to rs1058388 locus. Furthermore, mutations spanning rs1058588 significantly abolished this inhibitory effect (Figure 2C). Additionally, quantitative real‐time PCR assays demonstrated that *miR‐185* remarkably downregulated *VAMP8* expression in NPC cells (Figure 2D). Together, these findings suggest that the rs1058588‐[C] allele disrupts *miR‐185* binding to facilitate *VAMP8* upregulation in NPC.

**Figure 4:**
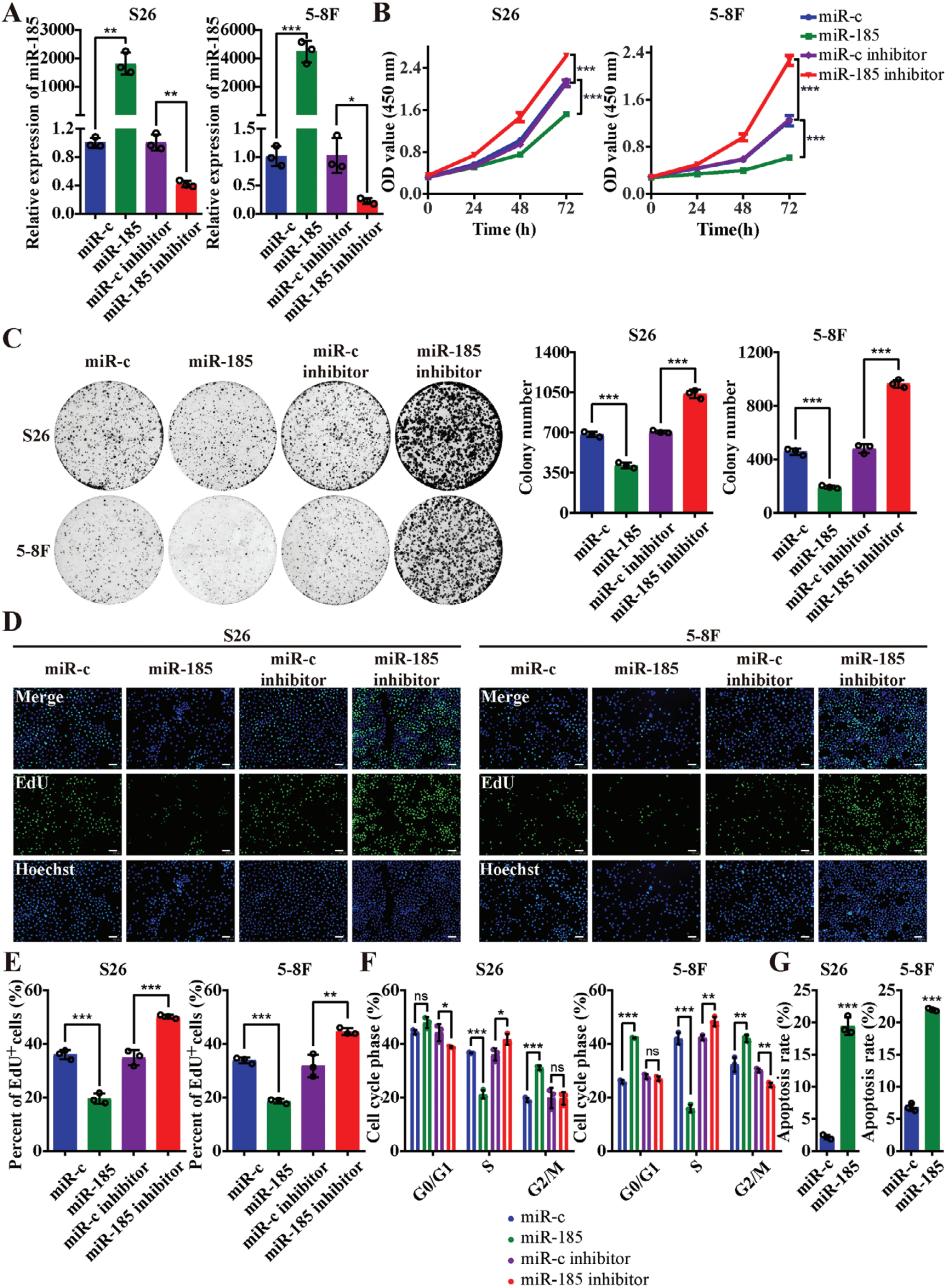
miR‐185 inhibits the proliferation of NPC cells. A) The expression level of miR‐185 measured using qRT‐PCR analysis in S26 and 5–8F cells. The cells were transfected with miR‐185 mimics, miR‐185 inhibitor or their respective negative controls. B) Number of viable cells reflected by the absorbance (450 nm) of CCK8 for the cells described in (A) at 0, 24, 48, and 72 h. C) Representative images of colony formation assay for the cells described in (A). Statistical results of colony numbers are shown at the right. D, E) Representative images D) and statistics analysis of EdU+ cells’ proportion E) of EdU assay for the cells described in (A). Scale bar, 100 µm. F) Flow cytometry analysis of cell cycle distribution after DNA labeling with propidium iodide in the cells described in (A). G) Flow cytometry analysis of apoptotic cells after staining with Annexin‐V‐FITC and propidium iodide in S26 and 5–8F cells transfected with miR‐185 or miR‐c mimics. ^*^ *p* < 0.05, ^**^ *p* < 0.01, and ^***^ *p* < 0.001. All statistical analyses were performed using two‐tailed Student's *t*‐test.

Given the observed inhibitory effects of *miR‐185* on the protumorigenic *VAMP8* expression, we next investigated the biological function of *miR‐185* in NPC cells (**Figure** **4**A). *miR‐185* overexpression significantly reduced cell proliferation (Figure 4B–E) and migration (Figure S7A–C, Supporting Information), while also induced G2/M cell cycle arrest and apoptosis (Figure 4F,G; and Figure S7D,E, Supporting Information). In contrast, inhibiting *miR‐185* augmented proliferation and migration (Figure 4; and Figure S7, Supporting Information). These findings collectively highlight a tumor suppressive role of *miR‐185*, contrasting with the protumorigenic role of VAMP8.

**Figure 5:**
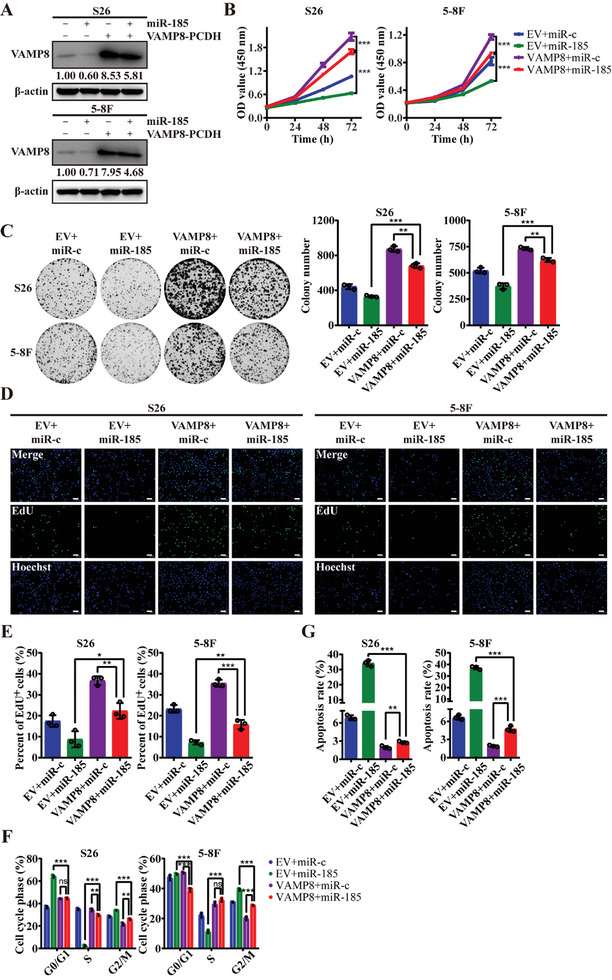
VAMP8 overexpression rescues the proliferation inhibitory effect of miR‐185 on NPC cells. A) Western blot assay showing VAMP8 protein levels in S26 and 5–8F cells or control cells with stable VAMP8 expression. These cells were transfected with miR‐185 or miR‐c mimics. β‐actin was used as control. B) Number of viable cells reflected by the absorbance (450 nm) of CCK8 for the cells described in (A) at 0, 24, 48, and 72 h. C) Representative images of colony formation assay for the cells described in (A). Statistical results of colony numbers are shown at the right. D,E) Representative images D) and statistics analysis of EdU+ cells’ proportion E) of EdU assay for the cells described in (A). Scale bar, 100 µm. F) Flow cytometry analysis of cell cycle distribution after DNA labeling with propidium iodide in the cells described in (A). G) Flow cytometry analysis of apoptotic cells after staining with Annexin‐V‐FITC and propidium iodide in the cells described in (A). ^*^ *p* < 0.05, ^**^ *p* < 0.01, and ^***^ *p* < 0.001. All statistical analyses were performed using two‐tailed Student's *t*‐test.

To further explore the interplay between *miR‐185* and VAMP8, we established NPC cell lines with overexpression of either *miR‐185*, *VAMP8*, or both. Consistent with the above observations, *miR‐185* overexpression significantly inhibited cell proliferation (**Figure** **5**) and migration (Figure S8, Supporting Information). However, simultaneous *VAMP8* overexpression abrogated these inhibitory effects (Figure 5; and Figure S8, Supporting Information). This dynamic relationship underscores the critical regulatory role of the miR‐185/VAMP8 axis in NPC development, with opposing effects on tumorigenesis.

### 2.5 VAMP8 Interacts with DHX9 to Promote NF‐κB Pathway Activation

**Figure 6:**
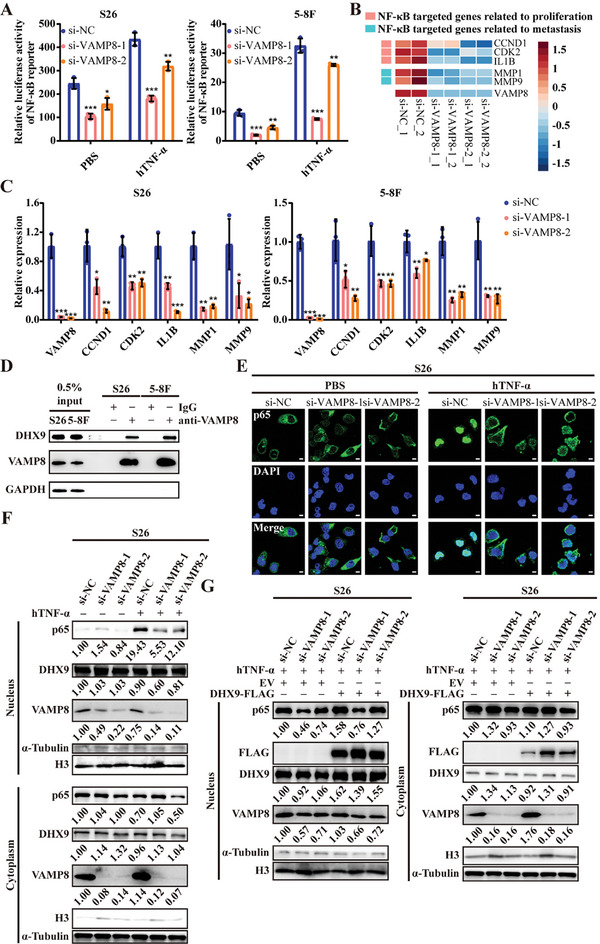
VAMP8 interacts with DHX9 to promote NF‐κB pathway activation. A) NF‐κB activity measured by Dual‐luciferase reporter assay in S26 and 5–8F cells. These NPC cells were transfected with VAMP8 siRNAs or control siRNA, together with hTNF‐α or PBS treatment for 8 h. B) Heatmap of VAMP8 and NF‐κB targeted genes related to proliferation and metastasis. The expression levels of these genes were analyzed using RNA‐seq data from S26 cells transfected with VAMP8 siRNAs or control siRNA. C) The mRNA expression levels of VAMP8 and NF‐κB targeted genes were measured by qRT‐PCR analysis in S26 and 5–8F cells transfected with VAMP8 siRNAs or control siRNA. D) Protein expression of DHX9 and VAMP8 in S26 and 5–8F cells. These NPC cell lysates were immunoprecipitated with IgG or anti‐VAMP8 antibody, followed by western blot assay to detect protein expression levels. GAPDH was used as control. E) Cellular localization of p65 in S26 cells upon VAMP8 knockdown and hTNF‐α treatment. Immunofluorescence was performed to visualize p65 (green) in S26 cells transfected with VAMP8 siRNAs or control siRNA, post‐treatment with hTNF‐α or PBS for 15 min. Nuclei were stained with DAPI (blue). Scale bar, 10 µm. F) Western blot assay showed the protein levels of p65, DHX9, VAMP8, α‐Tubulin (marker of cytoplasm), and histone H3 (marker of nucleus) in the cytoplasmic and nuclear fractions of S26 cells described in (E). G) Western blot assay showed the protein levels of indicated genes in the cytoplasmic and nuclear fractions of S26 cells. These cells were cotransfected with VAMP8 siRNAs or control siRNA and DHX9‐FLAG overexpression or empty vectors, followed by hTNF‐α stimulation for 15 min. ^*^ *p* < 0.05, ^**^ *p* < 0.01, and ^***^ *p* < 0.001. All statistical analyses were performed using two‐tailed Student's *t*‐test.

To elucidate the molecular mechanisms underlying the tumorigenic function of VAMP8, we conducted transcriptome analysis on NPC cells transfected with either *VAMP8* siRNAs or control siRNA. Differential gene expression analysis revealed 245 upregulated genes and 205 downregulated genes following *VAMP8* knockdown compared to control group (Table S12 and Figure S9A,B, Supporting Information). GSEA revealed that these differentially expressed genes were significantly associated with either the positive or negative regulation of NF‐κB signaling pathway (Figure S9C, Supporting Information). Luciferase reporter assay confirmed that *VAMP8* knockdown significantly reduced NF‐κB reporter activity in NPC cells compared to control cells, regardless the presence of hTNF‐α treatment (**Figure** **6**A). Transcriptome analysis, qRT‐PCR, and western blot assays corroborated that *VAMP8* knockdown remarkably downregulated the key NF‐κB target genes, such as CCND1, CDK2, IL1B, MPP1, and MMP9, in NPC cells^[^ ^12^, ^13^ ^]^ (Figure 6B,C; and Figures S5C and S9D, Supporting Information). These findings collectively suggest that VAMP8 may exert its tumorigenic effects through modulating the NF‐κB signaling pathway.

To explore how VAMP8 modulates the NF‐κB pathway, we first conducted coimmunoprecipitation (co‐IP) and mass spectrometry assay, identifying 269 and 117 potential VAMP8 binding proteins in S26 and 5–8F cell lines, respectively, with 85 overlapping proteins (Tables S13, S14, and Figure S10A,B, Supporting Information). Gene ontology (GO) analysis of these 301 proteins showed significant enrichment in pathway related to “positive regulation of NF‐κB transcription factor activity” (Figure S10C, Supporting Information). Notably, DHX9 emerged as the most abundant protein in this pathway (Tables S13 and S14, Supporting Information). Subsequent co‐IP assays and GST‐pulldown assays confirmed a strong direct interaction between DHX9 and VAMP8 (Figure 6D; and Figure S10D–F, Supporting Information). Immunofluorescence staining further revealed colocalizations of VAMP8 and DHX9 within the same cellular compartments (Figure S10G, Supporting Information), further supporting the direct interaction between these two proteins.

To elucidate the specific binding sites between VAMP8 and DHX9, we analyzed the domain structures of the two proteins using the UniProt database (https://www.uniprot.org), revealing a single domain for VAMP8 (v‐SNARE coiled‐coil homology domain) and four distinct domains for DHX9 (double‐stranded RNA‐binding motif 1 domain, double‐stranded RNA‐binding motif 2 domain, helicase ATP‐binding domain, and helicase C‐terminal domain). Co‐IP assays revealed that deletion of helicase C‐terminal domain completely abolished the interaction of DHX9 with VAMP8 (Figure S11, Supporting Information), suggesting its critical role in binding to VAMP8.

Considering DHX9's established role in facilitating p65 nuclear translocation through its interaction with p65,^[^ ^14^, ^15^, ^16^ ^]^ we conducted immunofluorescence and nucleoplasmic separation assays to explore VAMP8's effect on the DHX9 and p65 axis. *VAMP8* knockdown significantly impaired p65 nuclear translocation following hTNF‐α treatment in NPC cells (Figure 6E,F), while DHX9 overexpression substantially reversed this inhibitory effect (Figure 6G). Together, these findings strongly suggest that VAMP8 facilitates DHX9‐mediated p65 nuclear translocation, thereby activating the NF‐κB pathway in NPC cells.

## 3 Discussions

Here we have identified *VAMP8* as a novel susceptibility gene for NPC at chromosome 2p11.2 through a TWAS and subsequently GWAS and meta‐analysis. Furthermore, our findings demonstrate that the rs1058588 variant, located in the 3′‐UTR of *VAMP8*, acts as a functional variant, contributing to both eQTL and GWAS signals at the *VAMP8* locus. Previous studies have shown that rs1058588 contributes to cardiovascular disease risk by promoting vascular lesion formation and progression through its effects on vascular smooth muscle cell proliferation and inflammatory responses.^[^ ^17^, ^18^ ^]^ To the best of our knowledge, this is the first study linking rs1058588 to cancer susceptibility, specifically NPC, being benefited from sufficient statistical power with large sample sizes to detect this variant with modest effect size (OR = 1.18). Notably, two SNPs (rs10187424 and rs1010), which are in complete linkage disequilibrium with rs1058588 (*r* ^2 = ^1), have been previously associated with prostate cancer risk.^[^ ^19^, ^20^ ^]^ This finding further suggests the potential presence of cancer‐related gene(s) at this locus, providing additional validation for our results. Additionally, our TWAS identified 16 significant genes that colocalize within GWAS loci previously associated with NPC (Tables S1 and S2, Supporting Information), suggesting that common *cis*‐regulatory effects on gene expression mediate the functional consequences of risk variants linked to NPC susceptibility. These findings underscore the robustness of our TWAS approach in identifying novel risk loci for NPC, addressing gaps in its missing heritability.

Our study reveals that VAMP8 exhibits protumorigenic capability in NPC, enhancing cell proliferation and migration. Survival analysis further highlights a significant correlation between elevated *VAMP8* expression and poor survival outcomes in NPC patients. Collectively, these findings strongly support the role of VAMP8 as an oncogene with potential prognostic significance in NPC. VAMP8 is a member of the synaptobrevin/vesicle‐associated membrane protein subfamily of soluble N‐ethylmaleimide‐sensitive factor attachment protein receptors (SNAREs).^[^ ^21^, ^22^ ^]^ As an integral membrane protein, VAMP8 plays a crucial role in cellular secretion processes, particularly in autophagy, where it facilitates the fusion of endosomes/lysosomes with autophagosomes, thereby influencing autolysosome formation.^[^ ^21^, ^22^ ^]^ Previous studies reported that VAMP8 promotes cellular proliferation and tumor progression in glioma.^[^ ^23^ ^]^ Conversely, other studies demonstrated that VAMP8 suppresses metastasis in osteosarcoma through the DDX5/β‐catenin signaling pathway.^[^ ^24^ ^]^ These observations suggest a context‐dependent, dual role for VAMP8 in different cancer types.

Mechanistically, we show that rs1058588 modulates *VAMP8* expression by altering the binding affinity of *miR‐185*. *miR‐185* is a highly conserved microRNA encoded on human chromosome 22 and widely recognized as a tumor suppressor in multiple cancers, such as lung, liver, breast, and colorectal cancers.^[^ ^25^, ^26^ ^]^ *miR‐185* exerts its tumor‐suppressive effects by targeting oncogenes such as IGF1R, DNMT1, and components of the Wnt pathway.^[^ ^25^, ^26^ ^]^ Consistent with these findings, we observed that *miR‐185* inhibits NPC progression. Importantly, VAMP8 overexpression counteracts the tumor‐suppressive effects of miR‐185. These findings establish a functional link between VAMP8 and *miR‐185* in NPC pathogenesis, underscoring the therapeutic potential of targeting this axis.

Further mechanistic investigations reveal that VAMP8 interacts directly with DHX9 to facilitate nuclear translocation of p65, thereby activating the NF‐κB pathway, which is a well‐established tumorigenic event in NPC.^[^ ^12^ ^]^ DHX9, also known as RNA helicase A (RHA), belongs to the DEAH‐box RNA helicase family and is ubiquitously expressed in eukaryotes.^[^ ^14^ ^]^ Previous studies have demonstrated that DHX9 is upregulated in various cancers, such as lung cancer, liver cancer, and colorectal cancer.^[^ ^15^, ^16^, ^27^, ^28^ ^]^ Specifically, DHX9 interacts with p65 and RNA polymerase II to enhance the expression of NF‐κB target genes, promoting malignant phenotypes in colorectal cancer.^[^ ^15^ ^]^ Our data further unravel that the helicase C‐terminal domain of DHX9 mediates its binding to VAMP8. Taken together, these findings establish that VAMP8 promotes NPC progression by regulating the DHX9/p65 axis and activating the NF‐kB signaling pathway, highlighting VAMP8 as a critical regulator in NPC pathogenesis.

**Figure 7:**
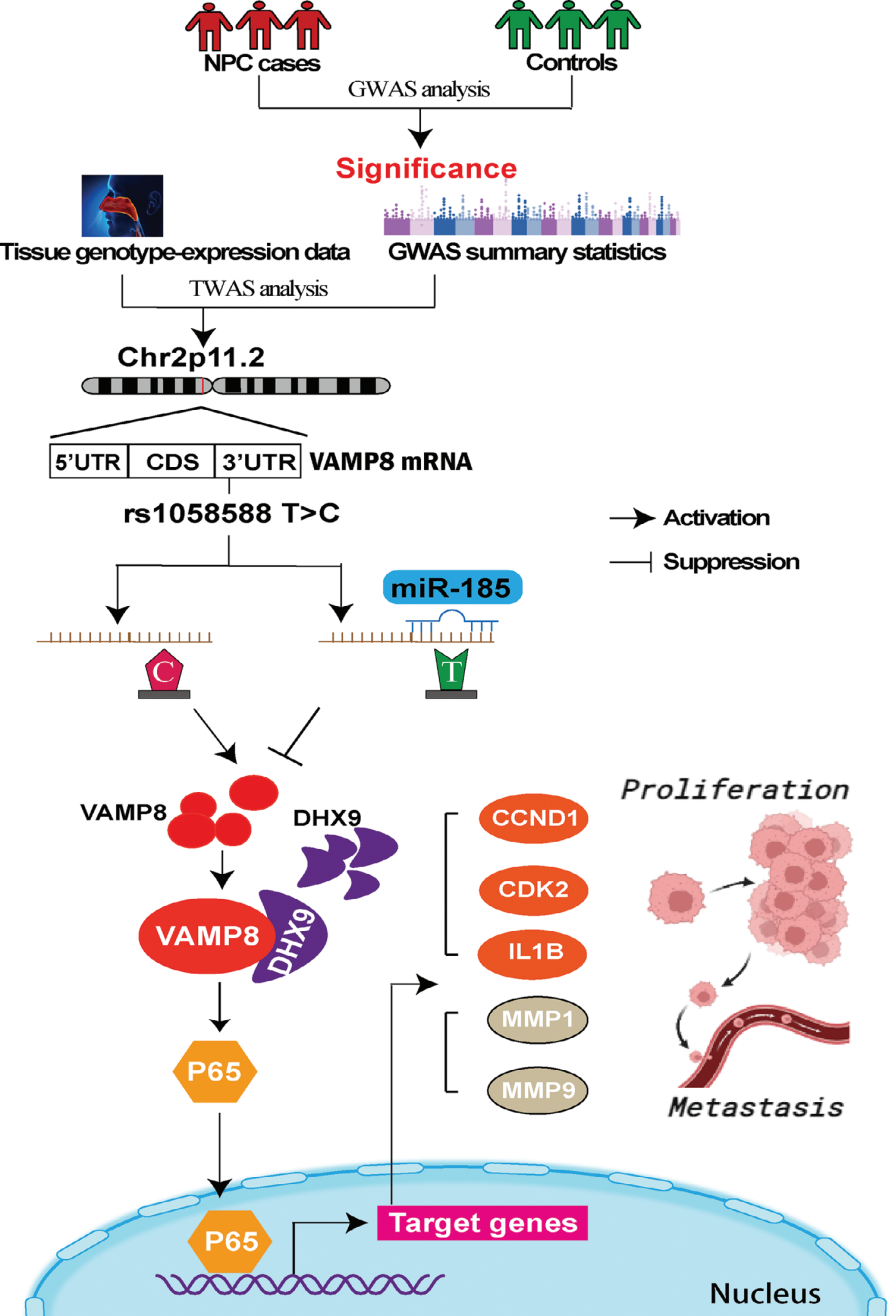
Schematic diagram for biological implication of the VAMP8 association and its underlying molecular mechanism in NPC. Integrating transcriptome‐wide association study (TWAS), expression quantitative trait loci (eQTL), and genome‐wide association study (GWAS) identifies rs1058588 as a casual variant associated with NPC risk. Individuals with rs1058588‐C risk allele may have weaker miR‐185 binding affinity to the 3′‐UTR of VAMP8, leading to a higher expression of VAMP8. VAMP8 executes protumorigenic potential through interacting with DHX9 to trigger nucleus p65 recruitment and NF‐κB pathway activation, thereby resulting in higher risk of NPC.

In summary, our TWAS, coupled with genetic associations across multiple cohorts, identifies *VAMP8* as a novel susceptibility gene for NPC. Integrating experimental evidence, we hypothesize that individuals carrying the rs1058588‐C risk allele at the *VAMP8* locus have reduced *miR‐185* binding affinity at the 3′‐UTR of *VAMP8*, leading to VAMP8 upregulation; elevated VAMP8 subsequently interacts with DHX9 to promote p65 recruitment to nucleus and the NF‐κB pathway activation, increasing NPC risk (**Figure** **7**). Our findings provide novel insights into the genetic underpinnings and pathogenesis of NPC, highlighting the critical interplay among *miR‐185*, VAMP8, DHX9, and the NF‐κB pathway. However, our study has certain limitations. First, it focuses exclusively on cis‐eQTLs in TWAS, which may overlook other mechanisms regulating gene expression. Second, while *VAMP8* is the primary focus of our study, additional studies are needed to validate other candidate genes identified with a looser FDR‐corrected *P*‐value threshold (Table S15, Supporting Information). Finally, the precise molecular mechanisms by which VAMP8 and DHX9 interaction facilitates p65 nuclear translocation require further elucidation. Our findings open venues to future studies to explore VAMP8/DHX9/p65 axis as biomarkers and therapeutic targets in NPC, with implications for understanding the broader genetic and molecular mechanisms of NPC pathogenesis.

## 4 Experimental Section

### Study Participants and Sample Inclusion

The study included NPC cases and controls for genetic association tests and whole transcriptome sequencing (WTS) analysis. All cases in this study were newly diagnosed NPC patients, with each diagnosis histopathologically confirmed by at least two pathologists according to the World Health Organization (WHO) classification criteria. The non‐NPC controls were individuals that self‐reported free of any malignancy at the time of enrolment.

To obtain GWAS summary statistics for the TWAS, 1583 NPC cases and 3040 controls of southern Chinese ancestry from the previous NPC GWAS were included.^[^ ^5^, ^29^ ^]^ To boost statistical power, an additional 3348 southern Chinese controls from the another GWAS was further incorporated.^[^ ^30^ ^]^ To construct gene expression prediction models for the TWAS, 46 NPC cases were independently recruited at the Sun Yat‐sen University Cancer Center (SYSUCC).

For the individual SNP‐based association analysis, the above GWAS data were treated as the discovery stage. Additionally, two independent case‐control samples recruited from southern China were treated as the replication stage. This included 1941 NPC cases and 975 controls from Zhongshan, Guangdong province (Validation‐1), as well as 2334 NPC cases and 2507 controls from Hong Kong (Validation‐2) in China.

For WTS analysis, biopsy samples were included from 93 NPC cases and 10 individuals with Rhinitis, a conditions marked with immune cell infiltration and inflammation in the nasal airway, from the SYSUCC.^[^ ^31^ ^]^ To further boost statistical power, an additional WTS dataset of 113 NPC tumor samples was incorporated, retrieved from the public GEO database (Accession number: GSE102349).^[^ ^32^ ^]^

### Generation of GWAS Summary Statistics

To obtain GWAS summary statistics, first stringent quality control filtering for both SNP variants and samples, using PLINK (v1.9) as described previously was first conducted. Briefly, variants were excluded with low genotyping quality, including those with a genotyping call rate below 95%, a significant disparity in the missing rate between cases and controls (Fisher's exact test, *p* < 1.0 × 10^−5^), a minor allele frequency (MAF) less than 1%, or a significant deviation from Hardy–Weinberg Equilibrium (HWE) in the controls (*p* < 1.0 × 10^−6^). At the sample level, individuals were excluded with genotyping call rates below 95% or exhibiting extreme heterozygosity (beyond three times standard deviation from the mean). Biologically related individuals were identified and excluded (first‐ or second‐degree relatives) detected through identify by descent testing, preferring to retain the individual with a higher genotyping call rate in each pair. All outliers in genetic ancestry as determined by principal component analysis (PCA) were also removed. For imputing additional variants not directly measured in the original genotype arrays, IMPUTE2 (v2.3.2) was utilized in conjunction with the 1000 Genomes Project (phase I), with a prephasing strategy with SHAPEIT (v2) to improve the imputation performance. After imputation, variants were removed with imputation quality (INFO score ≤ 0.8), MAF < 1%, or a significant deviation from HWE in the controls. Finally, a total of 3 874 508 autosomal SNPs for 1577 NPC cases and 6359 healthy controls were included in the GWAS to generate summary statistics for subsequent analyses.

### TWAS Procedures

A series of TWASs was performed using five sets of gene expression prediction models that were trained with a combination of datasets and softwares. These included three models based on GTEx esophagus mucosa tissue dataset or The Cancer Genome Atlas Head Neck Squamous Cell Carcinoma (TCGA.HNSC) dataset available at the MetaXcan (GTEx.Esophagus_Mucosa‐MetaXcan; https://github.com/hakyimlab/MetaXcan) or the FUSION website (GTEx.Esophagus_Mucosa‐FUSION and TCGA.HNSC‐FUSION; http://gusevlab.org/projects/fusion/). The remaining two prediction models were developed using in‐house scripts based on NPC data and MetaXcan (NPC‐MetaXcan) or FUSION (NPC‐FUSION) frameworks. Detailed descriptions of these customized models are provided below.

Step 1: Genomic and transcriptomic data preparation

To generate genomic data, whole blood DNA samples were extracted from 42 NPC patients for whole exome sequencing (WES) and from four NPC patients for whole genome sequencing (WGS). DNA library was constructed using Agilent SureSelect Human All Exon V6+UTR kit or Agilent SureSelect Human All Exon V5+UTR kit (Illumina, San Diego, CA) for WES as described previously and TruSeq Nano DNA Sample Prep Kit (Illumina, San Diego, CA) for WGS. Library was subsequently sequenced on an Illumina HiSeq instrument following a 150‐bp paired‐end protocol. After quality control, sequencing reads were aligned to the hg19 reference genome using the BWA mem algorithm to generate BAM file for each sample. For WES, the Sentieon toolkits (released at 2018.08.03) were employed for quality control and variant calling, with default setting parameters. Imputation of missing variants was conducted using GLIMPSE (v1.1.1) with 1000 Genomes Project (phase3) haplotypes, applying stringent quality controls to remove variants (info score < 0.8 or posterior probability < 0.9 or genotyping call rate < 95% or MAF < 1%). For WGS, duplicate reads in BAM files were marked and removed using the MarkDuplicates function of GATK (v3.5), and potential variants were called using GATK's HaplotypeCaller. Finally, the imputed WES and WGS data were combined, retaining only bi‐allelic variants for downstream analysis.

For gene expression data, total RNA samples were extracted from NPC tumors and subsequently subjected for whole transcriptome sequencing (WTS) as described previously. Sequencing reads with high quality were removed if they were aligned to ribosomal RNAs using Bowtie 2 (v2.5.1). Reads not aligned as ribosomal RNAs were realigned to the hg19 reference genomes using STAR (v2.7.10b). Gene expression levels were quantified using RNA‐SeQC (v2.3.5). Genes with low expression (raw counts < 5 or transcripts per kilobase million (TPM) < 0 in more than one‐tenths of samples) were excluded from further analysis. The TPM values for each gene were log2 transformed to normalize the data. A total of 46 NPC samples were processed to compile a dataset comprising 751302 SNPs and 25920 gene expression profiles, which were then utilized to construct gene expression prediction models.

Step 2: Building gene expression prediction models

Using MetaXcan software, expression prediction model for each gene was developed with the elastic‐net regression (Enet) algorithm implemented in the R package glmnet(v2.0‐16). The algorithm's settings included a ridge‐least absolute shrinkage and selection operator (LASSO) mixing parameter of *α* = 0.5 and a penalty parameter lambda determined through 10‐fold cross‐validation. The *cis*‐regulated expression level for each gene was estimated by including SNPs within 1 Mb range from the gene boundaries. The prediction performance of each model was assessed using R^2^ values, which is the square of the correlation between predicted and observed expression.

For FUSION software, genotypes within 0.5 Mb of each gene's boundaries were included in its mapping. For each gene, the *cis*‐heritability of gene expression was analyzed to determine genes whose expression could be partially explained by cis‐SNPs, using GCTA (v1.92.4beta). For genes with significant *cis*‐heritability (h2 g > 0 and *p* < 0.05), the genotype and expression data were used to build prediction models with five algorithms: LASSO regression, Enet, Best linear unbiased predictor (BLUP), Bayesian sparse linear mixed model (BSLMM), and Single best eQTL. The best‐performing algorithm, according to fivefold cross‐validation and highest R^2^ values, was chosen for model building.

For both softwares, prediction models were built for protein‐coding genes, long noncoding RNAs (lncRNAs), microRNAs (miRNAs), processed transcripts, immunoglobulin genes, and T cell receptor genes, as classified in the GENCODE (v19) gene annotation file. Pseudogenes were excluded due to concerns regarding potential inaccurate calling.

Step 3: Association analysis of predicted gene expression with NPC risk

Gene‐based association tests were conducted, correlating predicted gene expression levels with NPC risk, using the MetaXcan and FUSION software. These analyses utilize the weights from gene expression prediction models, GWAS summary statistics results, and correlations among SNPs predicated by the models as input variables. MetaXcan and FUSION analyses were conducted separately for each of the five sets of gene expression prediction models, each time using the same GWAS summary statistics generated earlier above.

To determine significant associations, a conservative Bonferroni correction is applied, which is calculated as 0.05 divided by the total number of tests conducted at transcriptome‐wide level. Additionally, to uncover further potential risk gene candidates, a false discovery rate (FDR)‐corrected transcriptome‐wide significance threshold was also considered.

### Conditional Analysis

To pinpoint independent risk genes within the region exhibiting multiple significant TWAS signals, summary‐based joint/conditional testing was employed using the FUSION software. Additionally, this analysis was also used to evaluate the residual association of genetic variants with GWAS results, even after accounting for the effect of genetically predicted gene expression.

To investigate whether the associations between TWAS‐identified genes and disease risk were mediated by specific SNPs, COJO conditional analyses implemented in GCTA (v1.92.4beta) were conducted. Briefly, COJO conditional analysis involves association tests between SNPs and disease risk while adjusting for a specific SNP, resulting in betas and standard errors. Subsequently, the TWAS analysis was reperformed using the updated summary association statistics.

### Colocalization Analysis

To estimate the posterior probability that eQTL and GWAS associations were driven by a shared causal variant, colocalization analysis was conducted using the R package COLOC (v5.1.0.1) with default settings. COLOC evaluates five hypotheses: H0 for no association in either eQTL or GWAS, H1 for association in eQTL only, H2 for association in GWAS only, H3 for independent associations in both eQTL and GWAS, and H4 for shared association in both eQTL and GWAS. The primary aim is to determine whether the eQTL and GWAS signals align with a shared SNP (H4). The analysis outputs five posterior probabilities including PP0, PP1, PP2, PP3, and PP4. A high posterior probability for PP4 exceeding 75% indicates that the eQTL and GWAS signals likely colocalize, suggesting a shared genetic basis for the observed associations.

### Derivation of Non‐TWAS‐Informed Gene‐Based Statistics

To validate the signals identified through TWAS, an alternative gene‐based analysis was performed. This approach aggregates a set of SNP‐level associations into a single gene‐level association signal, using MAGMA (v1.07b) with default settings. The process comprised annotation and statistic calculation steps. For annotation, a SNP was mapped onto a gene if it was located within the gene's boundaries. Reference files for the 1000 genomes East Asian panel and gene location data (NCBI 37.3) were downloaded from the MAGMA website (http://ctglab.nl/software/magma). For statistic calculation, the SNP‐wise mean model was employed to derive gene‐level *p* values, using the GWAS summary statistics.

### WTS Analysis

RNA samples were extracted from NPC tumors, rhinitis tissues, and NPC cell lines and subsequently subjected for WTS. The detailed procedures, including RNA extraction, quality control, sequence alignment, and expression quantification, were provided in the above section titled “Step 1: genomic and transcriptomic data preparation.”

### Cell Culture and Reagents

Human NPC cell lines (S26, 5–8F, C666‐1, CNE‐2, HK‐1, and HONE1) were generously provided by Professor Chaonan Qian at the SYSUCC and human embryonic kidney 293T cells were acquired from the Cell Bank of Type Culture Collection of Chinese Academy of Sciences, Shanghai Institute of Cell Biology, Chinese Academy of Sciences. All cell lines were cultured in Dulbecco's Modified Eagle Medium (DMEM, Gibco, NY) supplemented with 10% fetal bovine serum (Gibco, NY) and 1% Antibiotic‐Antimycotic (Gibco, NY) at 37 °C in a humidified atmosphere containing 5% CO_2_.

For VAMP8 knockdown, double‐stranded siRNAs oligos targeting VAMP8 were designed and purchased from GenePharma (Suzhou, China). The sense strands of siRNA duplexes are CCAGUGAAGGUGGAGGAAATT (siRNA‐VAMP8‐1) and CCUCUUCAUUGUGCUCUUUTT (siRNA‐VAMP8‐2) for VAMP8, and UUCUCCGAACGUGUCACGUTT (siRNA‐NC) as control. Cells were transfected with siRNA duplexes using Lipofectamine RNAiMAX (Invitrogen, Carlsbad, CA). Primary antibodies used in this study were commercially available, including anti‐VAMP8 (ab76021, Abcam, Cambridge, USA), anti‐E‐Cadherin (14472S, Cell Signaling Technology, Danvers, USA), anti‐N‐Cadherin (13116S, Cell Signaling Technology, Danvers, USA), anti‐Vimentin (5741S, Cell Signaling Technology, Danvers, USA), anti‐MMP9 (10375‐2‐AP, Proteintech Group, Chicago, USA), anti‐MMP1 (10371‐2‐AP, Proteintech Group, Chicago, USA), anti‐IL1B (26048‐1‐AP, Proteintech Group, Chicago, USA), anti‐CDK2 (10122‐1‐AP, Proteintech Group, Chicago, USA), anti‐CCND1 (ab134175, Abcam, Cambridge, USA), anti‐p65 (8242S, Cell Signaling Technology, Danvers, USA), anti‐DHX9 (67153‐1‐Ig, Proteintech Group, Chicago, USA), anti‐H3 (4499S, Cell Signaling Technology, Danvers, USA), anti‐α‐Tubulin (2144S, Cell Signaling Technology, Danvers, USA), anti‐GAPDH (RM2002, Rayantibody, Beijing, China), anti‐β‐actin (AC004, ABclonal, Wuhan, China), anti‐FLAG (F1804, Sigma‐Aldrich, St. Louis, USA), and anti‐HA (AE008, ABclonal, Wuhan, China).

### Quantitative Real‐Time PCR (qRT‐PCR)

Total RNA was isolated from cultured cells using RNAiso Plus (Takara, Tokyo, Japan). RT‐PCR was performed using oligo (dT) priming and M‐MLV Reverse Transcriptase according to the manufacturer's instructions (Promega Corporation, Madison, WI). To detect the expression level of specified genes, qRT‐PCR was performed using the TBGreen Mix Kit (Takara, Tokyo, Japan) and a CFX96 Real‐Time System (Bio‐Rad, Hercules, CA). ACTB expression was used as an endogenous control. The sequences of the gene‐specific primers used are listed in Table S16 (Supporting Information).

### Dual‐Luciferase Reporter Assay

Tools including SNPinfo and mirsnpscore were used to predict miRNAs that could potentially interact with a functional variant. A 331‐bp segment of the 3′‐ UTR region of VAMP8, containing either rs1058588[C] or rs1058588[T] allele, was constructed into psiCHECK‐2 plasmids (Promega, Madison, WI) using XhoI and NotI restriction enzymes (New England Biolabs, Ipswich, MA). Luciferase reporter plasmids of NF‐κB were purchased from Yeasen Biotechnology (Shanghai, China). Approximately 5×10^4^ cells per well were seeded into 24‐well plates, and then cotransfected with reporter plasmids and either miRNA mimics or siRNA duplexes using Lipofectamine 2000 (Invitrogen, Carlsbad, CA). After 48 h, luciferase activity was measured with a Dual‐Luciferase Assay Kit (Promega, Madison, WI) according to the manufacturer's protocols.

### Coimmunoprecipitation (co‐IP) and Western Blot Assays

To verify the interaction between VAMP8 and DHX9 and to explore the specific binding sites for these two proteins, coimmunoprecipitation (co‐IP) experiments were performed. For the interaction between VAMP8 and DHX9, S26 and 5–8F cells were lysed with cell lysis buffer (Cell Signaling Technology, Danvers, USA) containing 1× protease inhibitor cocktail (Beyotime, Shanghai, China) for 30 min on ice. After centrifugation, cell lysate supernatant was incubated with anti‐VAMP8 antibody overnight at 4 °C. The antibody‐protein complexes were then incubated with protein A/G magnetic beads (Thermo Fisher Scientific, Waltham, MA) for 4 h at 4 °C. The beads‐antibody‐protein complexes were washed five times with lysis buffer and then eluted using 1× dual‐color protein loading buffer (Fdbio science, Hangzhou, China). The interaction between endogenous VAMP8 and DHX9 was analyzed by western blot assay with indicate antibodies. Additionally, FLAG‐tagged DHX9 was exogenously overexpressed in S26 and 5–8F cells. Total protein extracts from these cells were subjected to immunoprecipitation using anti‐FLAG antibody. The immunoprecipitated complexes were then analyzed by western blot assay to verify the interaction between endogenous VAMP8 and the exogenously expressed FLAG‐tagged DHX9. For exploring the specific binding sites of VAMP8 and DHX9, a series of truncated DHX9 mutation constructs were generated based on the FLAG‐tagged DHX9 expression plasmid: DHX9^ΔDRBM1‐FLAG^, which lacks the double‐stranded RNA‐binding motif 1 domain; DHX9^ΔDRBM2‐FLAG^, which lacks the double‐stranded RNA‐binding motif 2 domain; DHX9^ΔATP‐FLAG^, which lacks the helicase ATP‐binding domain; DHX9^ΔC‐FLAG^, which lacks the helicase C‐terminal domain. After cotransfecting with VAMP8^HA^ and either DHX9^WT‐FLAG^, DHX9^ΔDRBM1‐FLAG^, DHX9^ΔDRBM2‐FLAG^, DHX9^ΔATP‐FLAG^, DHX9^ΔC‐FLAG^, or FLAG empty plasmids in 293T cells for 48 h, the cells were lysed with cell lysis buffer containing 1× protease inhibitor cocktail for 30 min on ice. Then the protein extracts were then subjected to immunoprecipitation using anti‐FLAG antibody.

For western blot assay, cultured cells were lysed with RIPA lysis buffer (Fdbio science, Hangzhou, China) containing 1× protease inhibitor cocktail for 15 min on ice. After centrifugation, equal amounts of cell lysate supernatant were separated using sodium dodecyl sulfate‐polyacrylamide gel electrophoresis (SDS‐PAGE) and transferred to PVDF membranes (Millipore, Billerica, MA). Membranes were blocked with 5% bovine serum albumin (BSA) for 1 h at room temperature, then incubated with primary antibodies overnight at 4 °C. After three washes with 1× tris buffered saline with Tween 20 (TBST) buffer, the membranes were incubated with HRP‐linked secondary antibodies for 1 h at room temperature. Chemiluminescence was performed using the Fdbio‐Dura ECL kit (Fdbio science, Hangzhou, China) and visualized with a Bio‐Rad ChemiDoc Touch system (Hercules, CA).

### GST Pull‐Down Assay

VAMP8 coding sequence was cloned into pGEX‐4T‐1 vector, then glutathione S‐transferase (GST) and GST‐fused VAMP8 proteins were expressed and purified from *Escherichia coli* strain BL21 (TransGen, Beijing, China) using Glutathione Sepharose 4B (GE healthcare, Chicago, IL). 293T cells transfected with FLAG‐tagged DHX9 expression plasmid were lysed with cell lysis buffer containing 1× protease inhibitor cocktail for 30 min on ice. After centrifugation, cell lysate supernatant was incubated with GST and GST‐fused VAMP8 proteins overnight at 4 °C. The sepharose beads were washed three times with cell lysis buffer and boiled for 10 min with 2× dual‐color protein loading buffer. Prepared samples were then analyzed by western blot assay with anti‐FLAG antibody.

### Immunofluorescence and Confocal Microscopy

Cells grown in the glass bottom cell culture dish were fixed using 4% paraformaldehyde (BioSharp, Hefei, China) and permeabilized using 0.1% Triton X‐100 in PBS (Gibco, NY). After blocking with 5% goat serum in permeabilization buffer, cells were incubated with specified primary antibodies overnight at 4 °C. After three washes with PBS, cells were incubated with fluorescent secondary antibodies in a darkroom for 1 h at room temperature. Cell nuclei were stained using 4′,6‐diamidino‐2‐phenylindole (DAPI) staining solution. Fluorescence was detected using a confocal laser scanning microscope (Carl Zeiss, Microscope 880, Jena, Germany).

### Cell Proliferation Assays

CCK8, cell colony formation, and EdU assays were performed to evaluate the proliferation ability of NPC cells. For CCK8 assay, cells (1 × 10^3^ per well) were seeded in sextuplicate in 96‐well plates. After incubation, 10 µL of Cell Counting Kit‐8 (DOJINDO, Kumamoto, Japan) was added to each well and incubated for 2 h at 37 °C. The optical density (OD) at 450 nm was measured using an Infinite M200PRO (Tecan, Männedorf, Switzerland). For cell colony formation assay, ≈1 × 10^3^ cells per well were seeded into 6‐well plates and cultured in a humidified incubator at 37 °C. After 2 weeks, cell colonies were fixed with 4% paraformaldehyde and stained with crystal violet staining solution (Beyotime, Shanghai, China) for 15 min at room temperature. For EdU assay, 4 × 10^3^ cells per well were seeded in 96‐well plates and were incubated with 10 µm EdU solution (Beyotime, Shanghai, China) for 2 h. After fixation with 4% paraformaldehyde and permeabilization with 0.3% Triton X‐100, cells were incubated with Azide Alexa Fluor 488 and Hoechst 33 342 to label incorporated EdU and nuclei, respectively. Fluorescence was detected using an inverted fluorescence microscope (IX73; Olympus, Tokyo, Japan). The ratio of EdU‐positive cells was calculated to evaluate the proliferation ability.

### Cell Cycle and Apoptosis Assays

For cell cycle analysis, 2 × 10^5^ cells per well were seeded into 6‐well plates and collected after 48 h. Cells were washed with cold PBS, fixed in 70% ethanol at −20 °C overnight, and then washed twice with PBS. They were subsequently digested with RNase A (Takara, Tokyo, Japan) and stained with propidium iodide (Life technologies corporation, Gaithersburg, MD) at 37 °C for 30 min. Cell cycle stages were analyzed using a spectral flow cytometer (Sony, Tokyo, Japan) and data were processed with ModFit LT 5.0 software. For cell apoptosis assay, cells were washed twice with cold PBS and stained with propidium iodide and Annexin‐V‐FITC (Invitrogen, Carlsbad, CA) for 20 min at room temperature. Apoptotic cells were identified with a spectral flow cytometer and analyzed using FlowJo software.

### Cell Migration Analyses

For wound healing assay, 5 × 10^4^ cells per well were seeded into 2‐well silicone inserts (ibidi, Munich, Germany). Upon cells attachment, the insert was carefully removed, and the medium was replaced with serum‐free medium. The percentage of wound healing area at specified time points relative to initial scratch area was calculated to assess cell migration ability. For transwell assay, 5 × 10^4^ cells in serum‐free medium were placed in the upper chamber of 8 µm pore size transwell chambers (Corning, NY) set in 24‐well plates with 600 µL DMEM containing 10% FBS. After 18 h, cells on the underside of membrane were fixed with 4% paraformaldehyde and stained with crystal violet staining solution for 20 min at room temperature. Finally, migrated cells were quantified in six random fields under a light microscope.

### In Vivo Xenograft Models

Four‐weeks‐old male BALB/c nude mice were purchased from Beijing Vital River Laboratory Animal Technology (Beijing, China), then randomly assigned to experimental groups, and housed under specific pathogen free (SPF) condition. Approximately 1 × 10^6^ S26 cells with stable knockdown of VAMP8 or a negative control were mixed with Matrigel (0.20 v/v, Corning, NY), and then subcutaneously injected into the dorsal flank of nude mice, with eight mice in each group. Tumor size was measured every two days using calipers. Tumor volume was calculated by the following formula: length × width^2^ × 0.5. Finally, all mice were humanely sacrificed, and subcutaneous xenografts were excised for further analysis. All animal experiments were performed under protocols approved by the Institutional Animal Care and Use Committee of Sun Yat‐sen University.

### Genotyping Assay for Replication Samples

Genotyping assay for replication sample were performed as described previously. In brief, capturing probes covering SNPs in the VAMP8 locus were included in a customized panel (Nanodigmbio, Nanjing, China). The library was sequenced on the MGI DNBSEQ‐T7 system (MGI Tech Co., Ltd., Shenzhen, China) for 150‐bp pair‐end reads. The Sentieon toolkits (released at 2018.08.03) were used to align sequencing reads to the human genomes (hg19) and call variants in VCF format. PLINK (v1.9) was used to convert VCF files into binary format (.bed, .bim, .fam) for subsequent association analysis.

### Statistical Analyses

For SNP‐based association analysis, the discovery stage included all individuals involved in the TWAS analysis and the replication stage comprised individuals from Zhongshan, Guangdong province and Hong Kong. The association analysis was performed using a logistic regression with an additive model. The top ten principal components were introduced as covariates to correct for subtle population stratification in the discovery stage. Odds ratios (OR) and 95% confidence intervals (CI) were calculated for the risk allele of each SNP. Genome‐wide significance was set as *p* < 5 × 10^−8^, following general practice. Independence of association was evaluated using a conditional logistic regression analysis. All these analyses were conducted using PLINK (v1.9). Meta‐analysis was conducted to test the SNP effect across multiple study groups using R package metaphor (v3.8‐1). Cochran's *Q* test was employed to determine potential heterogeneity within meta‐analysis (Phet). To visualize the results, R package qqman (v0.1.4) was used to plot association *p* values, locuscomparer (v1.0.0) was used for colocalization results from pairs of eQTL and GWAS association, and eQTL surveys were conducted against GTEx V7 portal (http://www.gtexportal.org/home/).

In WTS analysis, gene expression level was normalized using TPM to minimize the impact of a potential batch effect. Wilcoxon test was performed to identify the differentially expressed genes (DEGs) between different tissue samples. The Pearson correlation coefficient between two genes was estimated using the cor analysis, and *p* values were estimated using the cor.test analysis implemented in R. For the comparison between different cell lines, R package DESeq2 (v1.38.3) was used to determine DEGs. Function enrichment analyses, including Gene Ontology (GO) and gene set enrichment analysis (GSEA), were performed using the online tools metascape (http://metascape.org/gp/index.html) and R package clusterprofiler (v4.2.2), respectively.

To evaluate the prognostic significance of VAMP8 gene expression levels, survival analysis of 93 NPC patients was conducted. Optimal cut‐off values for gene expression were determined using Cutoff Finder, stratifying patients into groups with higher or lower levels of VAMP8 expression. The survival endpoints include overall survival (OS), defined as the duration from the first treatment postprimary diagnosis to death of any cause, and disease‐free survival (DFS), defined as the time from the first treatment to the date of first recurrence or death of any cause. The association between gene expression level and survival outcome was performed using Cox proportional hazards regression model, adjusting for known prognostic covariates, including age and sex. Hazard ratios (HRs) estimated from the Cox analysis were reported as relative risks, along with corresponding 95% confidence intervals (CIs).

For in vitro and in vivo experiments, all data presented as histograms refer to a mean value ± standard deviation (SD) across independent experiments. Statistical analysis was performed using Student's *t*‐test. Two‐sided *p* values below 0.05 were considered significant unless specified otherwise.

### Ethics Approval and Consent to Participate

The study was approved by the Sun Yat‐sen University Cancer Center Ethics Committee (reference no. SL‐B2021‐032‐03). All animal experiments were performed under protocols approved by the Institutional Animal Care and Use Committee of Sun Yat‐sen University (2019000154). Written informed consent was obtained from all participants. Ethical approval was obtained from all relevant local Institutional Review Boards in accordance with the Declaration of Helsinki.

## Conflict of Interest

The authors declare no conflict of interest.

## Author Contributions

Y.L., X.‐Y.X., and G.‐W.L. contributed equally to this work. J.‐X.B. conceived and supervised this study; M.F.J., F.G.L., J.K., M.L.L., Q.‐Y.C., L.‐Q.T., V.W., and H.‐Q.M. provided clinical samples and information; Y.‐H.Z., A.‐Y.X., S.H., and P.‐P.W. performed sample preparation; Y.L., J.‐X.B., X.‐Y.X., G.‐W.L., X.M.B., C.‐L.L., and Y.N.Z. analyzed and interpreted data; X.‐Y.X. and S.‐Q.L. performed functional experiments; Y.L., X.‐Y.X., C.‐L.L., and J.‐X.B. interpreted the results and wrote the manuscript. All authors read and approved the final manuscript.

## Supporting information



Supporting Information

## Data Availability

The genotype data generated by sequencing have been deposited in the Genome Variation Map repository (https://ngdc.cncb.ac.cn/gvm/) of National Genomics Data Center (NGDC) under controlled access due to data privacy laws related to patient consent for data sharing with accession number GVM000580 and GVM000949. The key data in this study is deposited in the Research Data Deposit (RDD; No.: RDDB2025533744; http://www.researchdata.org.cn/). The codes used for all processing and analysis is available at https://github.com/bei‐lab/ or upon request. All related materials in this study are available upon request.
