# Supplementary material for: Integrative Transcriptome‐Wide Association Study With Expression Quantitative Trait Loci Colocalization Identifies a Causal VAMP8 Variant for Nasopharyngeal Carcinoma Susceptibility
Source: Adv Sci (Weinh). 2025 Jan 24;12(11):2412580. doi: 10.1002/advs.202412580 (PMC11923910; doi:10.1002/advs.202412580)

ADVANCED SCIENCE
Open Access

Supporting Information

for *Adv. Sci.*, DOI 10.1002/advs.202412580

Integrative Transcriptome-Wide Association Study With Expression Quantitative Trait Loci Colocalization Identifies a Causal VAMP8 Variant for Nasopharyngeal Carcinoma Susceptibility

*Yan Liang, Xiang-Yu Xiong, Guo-Wang Lin, Xiaomeng Bai, Fugui Li, Josephine Mun-Yee Ko, Yun-He Zhou, An-Yi Xu, Shu-Qiang Liu, Shuai He, Pan-Pan Wei, Qiu-Yan Chen, Lin-Quan Tang, Vivien Ya-Fan Wang, Hai-Qiang Mai, Chun-Ling Luo, Yanni Zeng, Maria Li Lung, Mingfang Ji and Jin-Xin Bei**

Supplementary Materials for

Integrative Transcriptome-wide Association Study with Expression Quantitative Trait Loci Colocalization Identifies a Causal VAMP8 Variant for Nasopharyngeal Carcinoma Susceptibility

This PDF file includes:

Figures. S1 to S11
Tables. S1 to S16
Uncropped western blot images

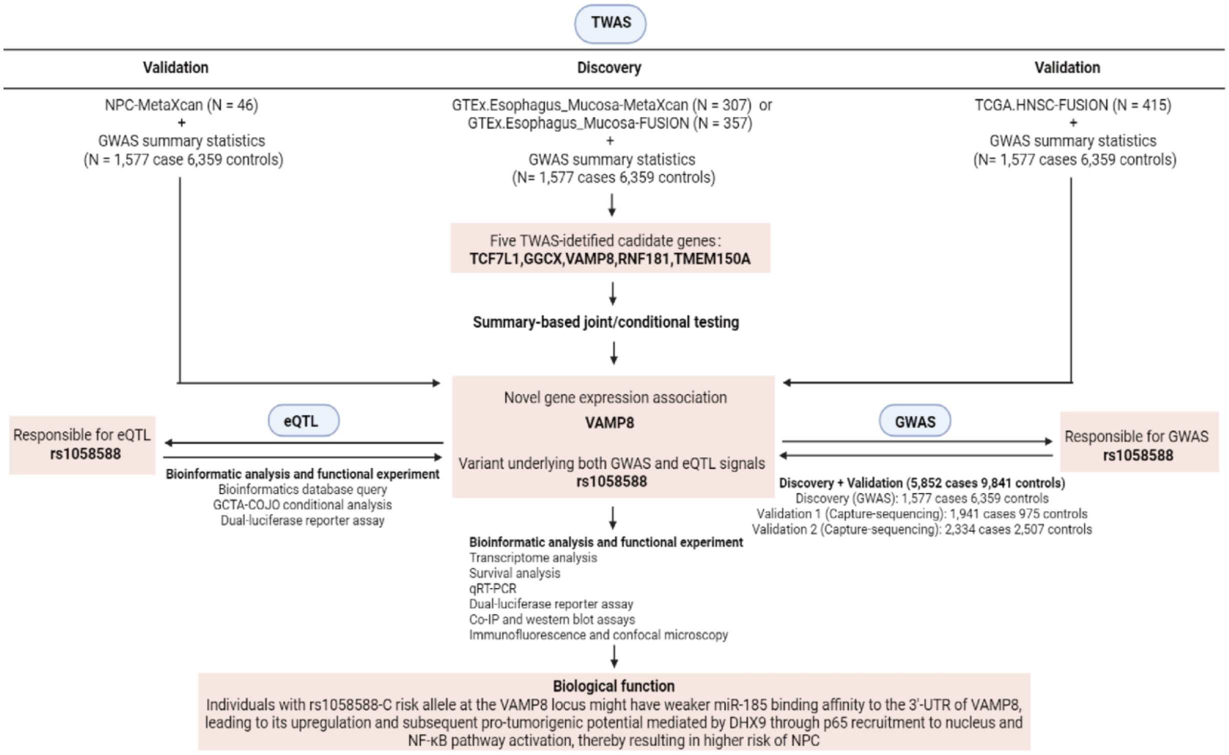

Figure S1. Study overview. We conducted a transcriptome-wide association study (TWAS) to identify novel susceptibility genes of nasopharyngeal carcinoma (NPC). The causal variant of TWAS-identified region was further fine mapped through expression quantitative trait loci (eQTL) colocalization and genome-wide association study (GWAS) analyses across various cohorts. In addition, our study delved into the biological role of TWAS-identified susceptibility genes, its regulatory mechanisms, and the impact on NPC tumorigenesis.

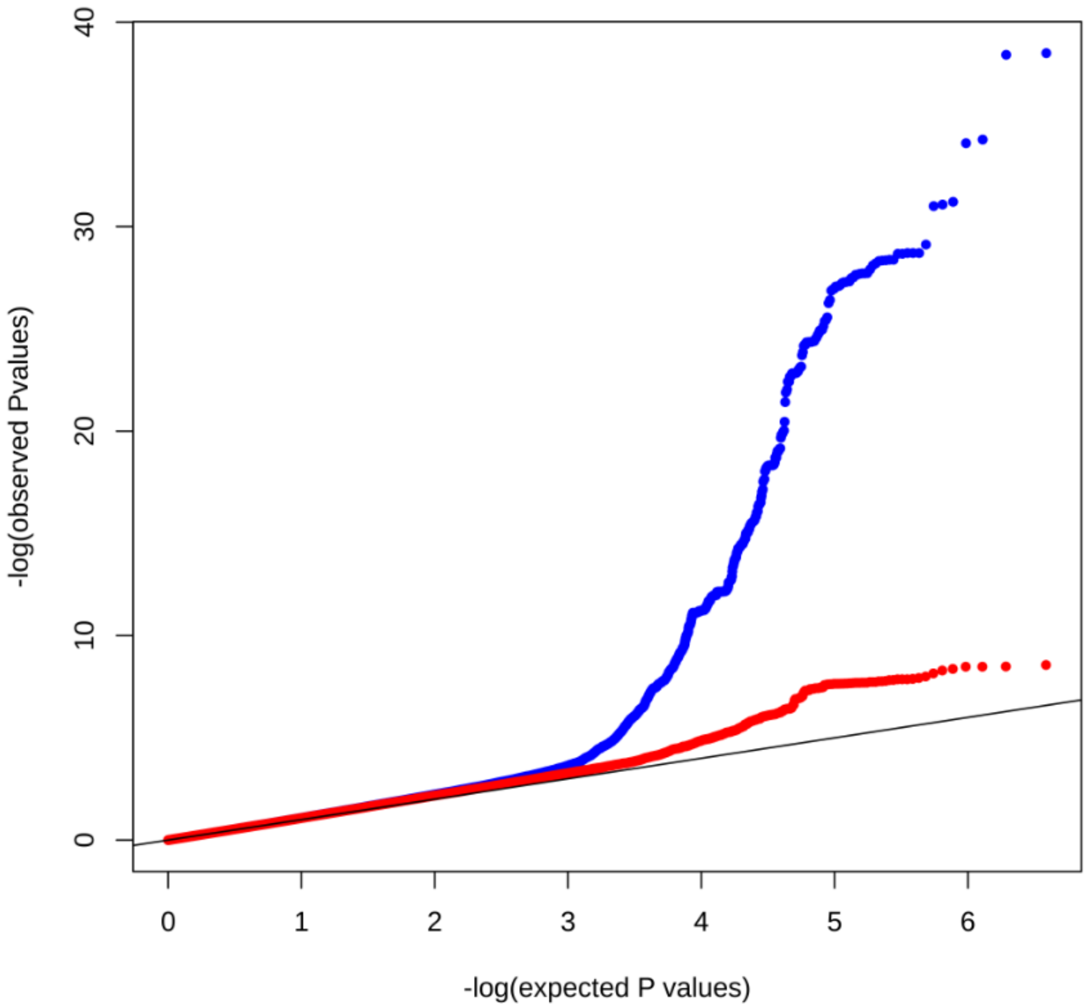

Figure S2. Quantile-quantile plot (QQ plot) of observed GWAS association *P* values. The GWAS comprises 1,577 NPC cases and 6,359 healthy controls of southern Chinese descent. The blue points represent the distribution of P values for the associations of all the autosomal SNPs, and the red points represent that for the associations of SNPs after excluding the extended MHC regional SNPs.

A

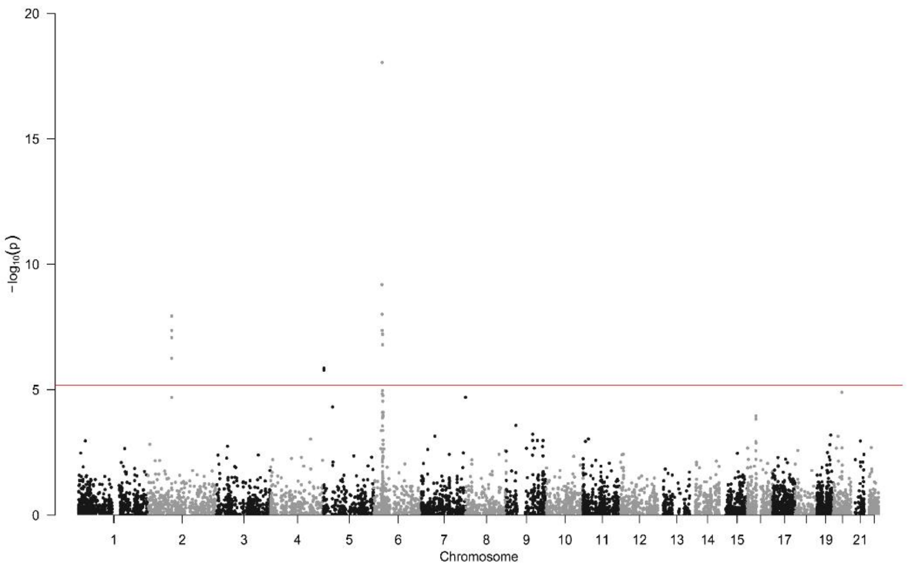

B

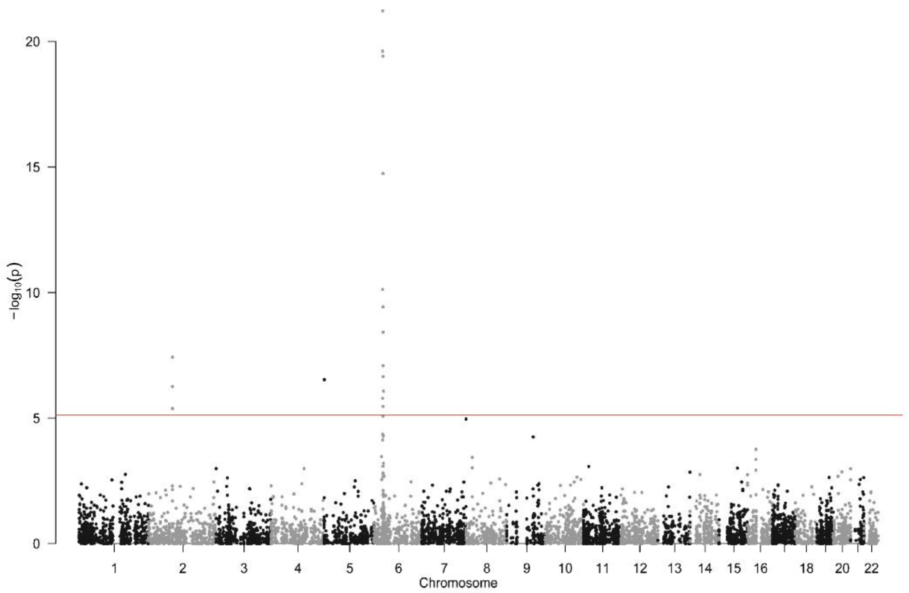

Figure S3. Manhattan plot of association results of the transcriptome-wide association study (TWAS) for nasopharyngeal carcinoma (NPC). Each point corresponds to an association test between genetically predicted gene expression for a specific gene and NPC risk. X axis represents the -log10 *P* values of genes; y axis represents the chromosomal positions of genes. **(A)** TWAS results based on GTEx.Esophagus_Mucosa-MetaXcan models with Bonferroni corrected threshold of 6.49×10^{-6} as shown by the red line; **(B)** TWAS results based on GTEx.Esophagus_Mucosa-FUSION models with Bonferroni corrected threshold of 7.54×10^{-6} as shown by the red line. GTEx: Genotype-Tissue Expression .

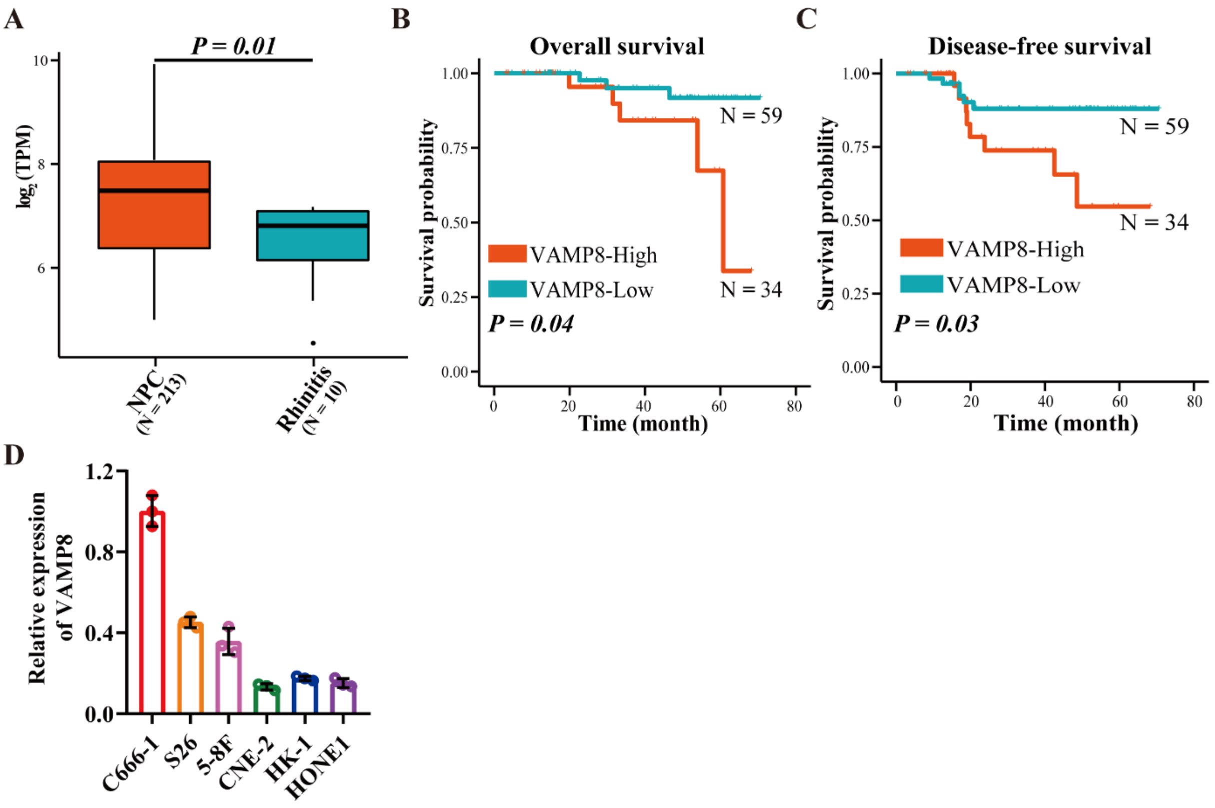

Figure S4. VAMP8 expression in tissues and various NPC cell lines. (A) Transcriptome analysis comparing the Transcripts Per Million (TPM) level of VAMP8 between NPC tumor tissues (N = 213) with control rhinitis tissues (N = 10) using Wilcoxon test. **(B, C)** Kaplan-Meier survival curves of overall survival **(B)** and disease-free survival **(C)** for patients with NPC grouped by VAMP8 expression calculated using Cox proportional hazards regression. **(D)** qRT-PCR analysis evaluated the mRNA expression levels of VAMP8 in different NPC cell lines. β-actin was used as control.

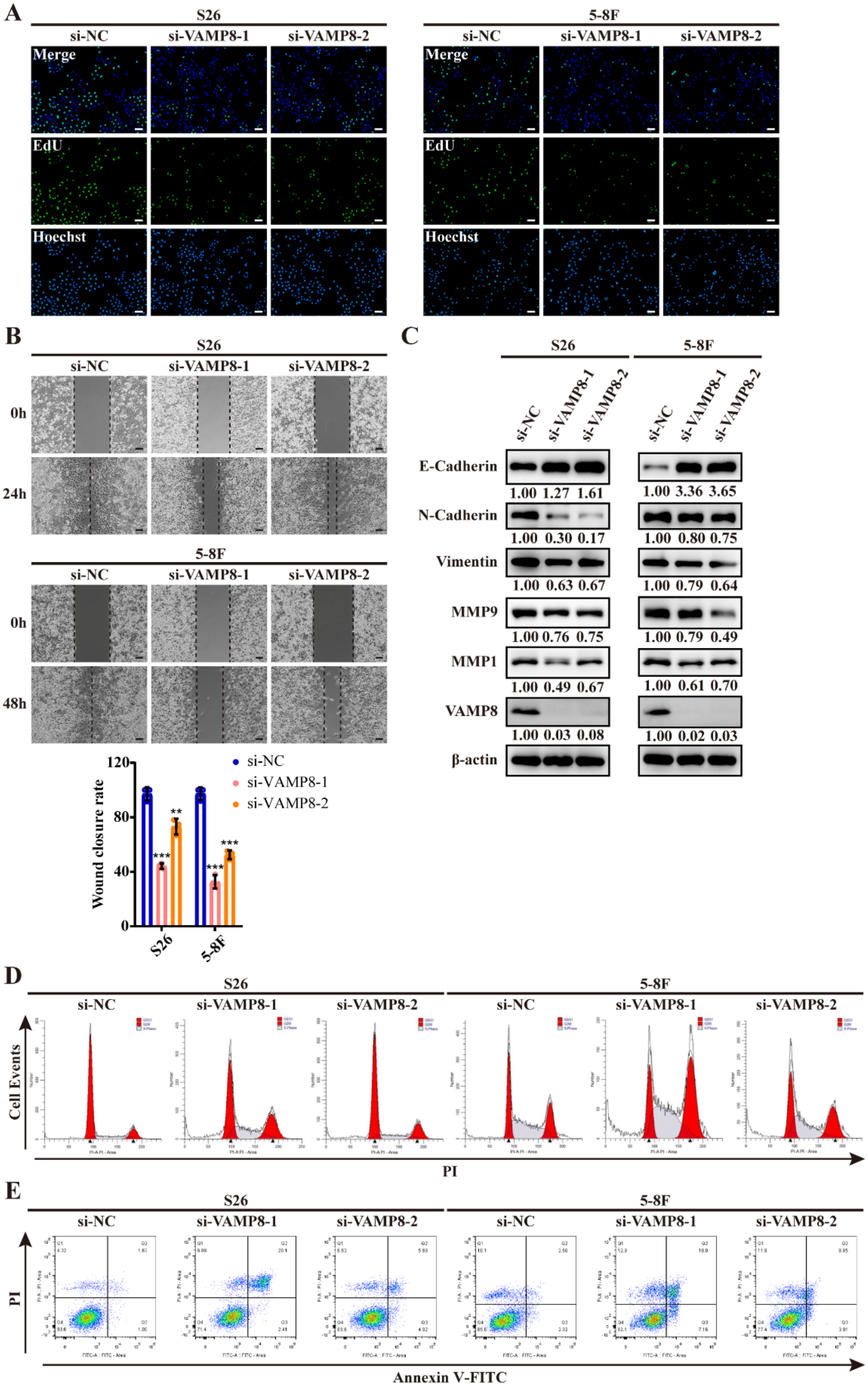
A
S26
5-8F
si-NC
si-VAMP8-1
si-VAMP8-2
Merge
EdU
Hoechst
B
0h
24h
48h
C
E-Cadherin
1.00 1.27 1.61
1.00 3.36 3.65
N-Cadherin
1.00 0.30 0.17
1.00 0.80 0.75
Vimentin
1.00 0.63 0.67
1.00 0.79 0.64
MMP9
1.00 0.76 0.75
1.00 0.79 0.49
MMP1
1.00 0.49 0.67
1.00 0.61 0.70
VAMP8
1.00 0.03 0.08
1.00 0.02 0.03
β-actin
Wound closure rate
D
Cell Events
PI
E
Annexin V-FITC

Figure S5. VAMP8 knockdown inhibits the proliferation and migration of NPC cells. (A) Representative images of EdU assay as supplement data for Figure 3D. NPC cells were knocked down with VAMP8, followed by EdU staining. Scale bar, 100 μm. **(B)** Representative images of wound healing assay for NPC cells with VAMP8 knockdown. S26 and 5-8F cells were monitored at different time points. Statistics analysis of wound closure rate are shown at the bottom. Scale bar, 100 μm. **(C)** Western blot assay showing the protein levels of E-Cadherin, N-Cadherin, Vimentin, MMP9, MMP1 and VAMP8 in S26 and 5-8F cells. These cells were transfected with siRNA duplexes targeted VAMP8 or scrambled siRNAs. β-actin was used as control. **(D)** Representative images of flow cytometry analysis for cell cycle distribution of NPC cells with VAMP8 knockdown, as supplement data for Figure 3E. x- and y- axes denote DNA content and cell number, respectively. Each cell cycle phase was calculated by ModFit LT 5.0 software. **(E)** Representative images of flow cytometry analysis for cell apoptosis of NPC cells with VAMP8 knockdown, as supplement data for Figure 3F. x- and y- axes denote Annexin V-FITC staining and PI staining, respectively. The proportion of apoptotic cells was calculated by FlowJo software. *$P < 0.05$, **$P < 0.01$, and ***$P < 0.001$. All statistical analyses were performed using two-tailed Student's t-test.

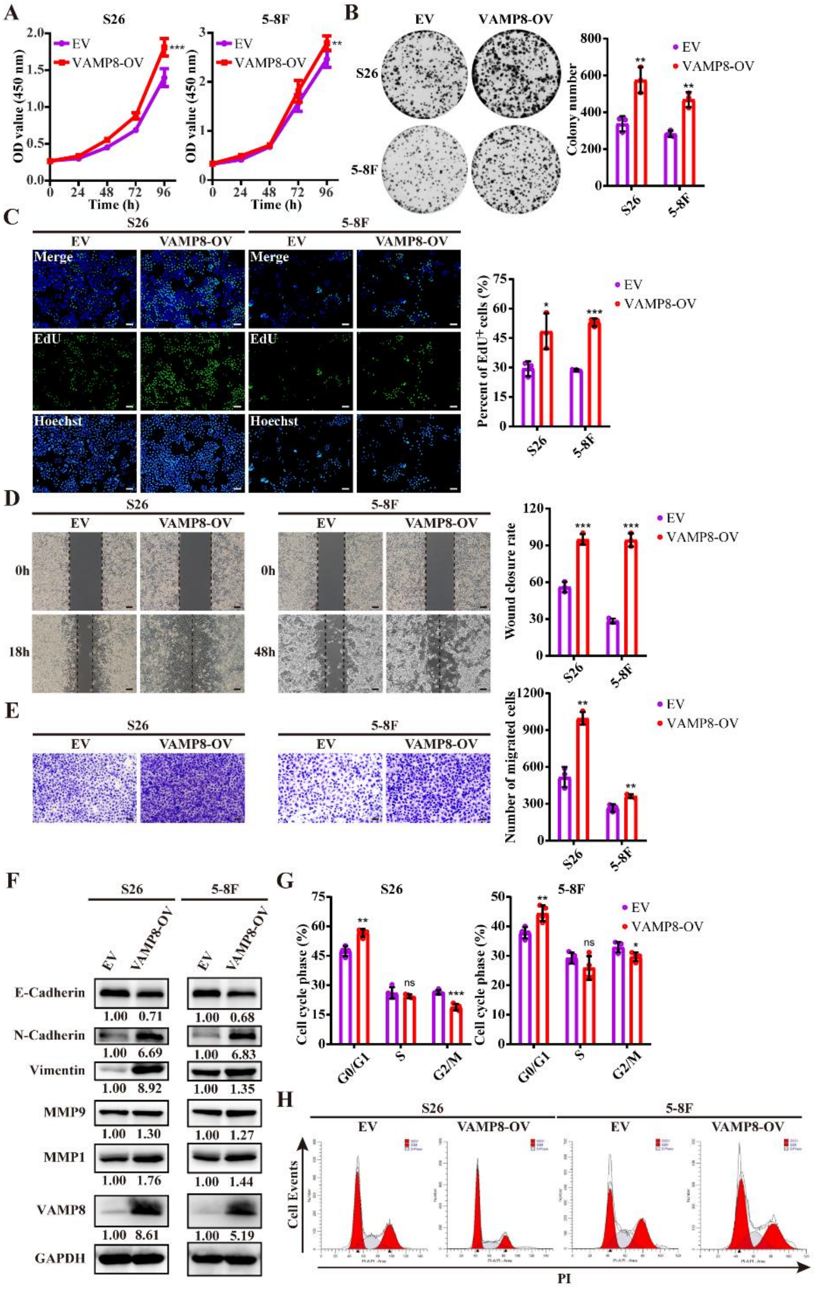
A
S26
5-8F
EV
VAMP8-OV
OD value (450 nm)
Time (h)
B
Colony number
C
Merge
EdU
Hoechst
Percent of EdU+ cells (%)
D
0h
18h
48h
Wound closure rate
E
Number of migrated cells
F
E-Cadherin
N-Cadherin
Vimentin
MMP9
MMP1
VAMP8
GAPDH
1.00 0.71
1.00 0.68
1.00 6.69
1.00 6.83
1.00 8.92
1.00 1.35
1.00 1.30
1.00 1.27
1.00 1.76
1.00 1.44
1.00 8.61
1.00 5.19
G
Cell cycle phase (%)
G0/G1
S
G2/M
ns
H
Cell Events
PI

Figure S6. VAMP8 overexpression promotes the proliferation and migration of NPC cells. (A) Number of viable cells reflected by the absorbance (450 nm) of CCK8 for S26 and 5-8F cells with stable overexpression of VAMP8 or with empty vector at 0 h, 24 h, 48 h, 72 h and 96 h. **(B)** Representative images of colony formation assay for the cells described in (A). Statistical results of colony numbers are shown at the right. **(C)** Representative images of EdU assay for the cells described in (A). Statistical results of EdU^+ cells' proportion are shown at the right. Scale bar, 100 μm. **(D)** Representative images of wound healing assay for the cells described in (A). Wound healing was monitored at different time points, with statistics analysis of wound closure rate shown at the right. Scale bar, 100 μm. **(E)** Representative images of transwell assay for the cells described in (A). Statistical results migrated cell numbers are shown at the right. Scale bar, 100 μm. **(F)** Western blot assay showing the protein levels of E-Cadherin, N-Cadherin, Vimentin, MMP9, MMP1 and VAMP8 in the cells described in (A). GAPDH was used as control. **(G)** Flow cytometry analysis of cell cycle distribution after DNA labelling with propidium iodide in the cells described in (A). **(H)** Representative images of flow cytometry analysis for cell cycle distribution in the cells described in (A), as supplement data for (G). x- and y- axes denote DNA content and cell number, respectively. Each cell cycle phase was calculated by ModFit LT 5.0 software. *$P < 0.05$, **$P < 0.01$, and ***$P < 0.001$. All statistical analyses were performed using two-tailed Student's t-test.

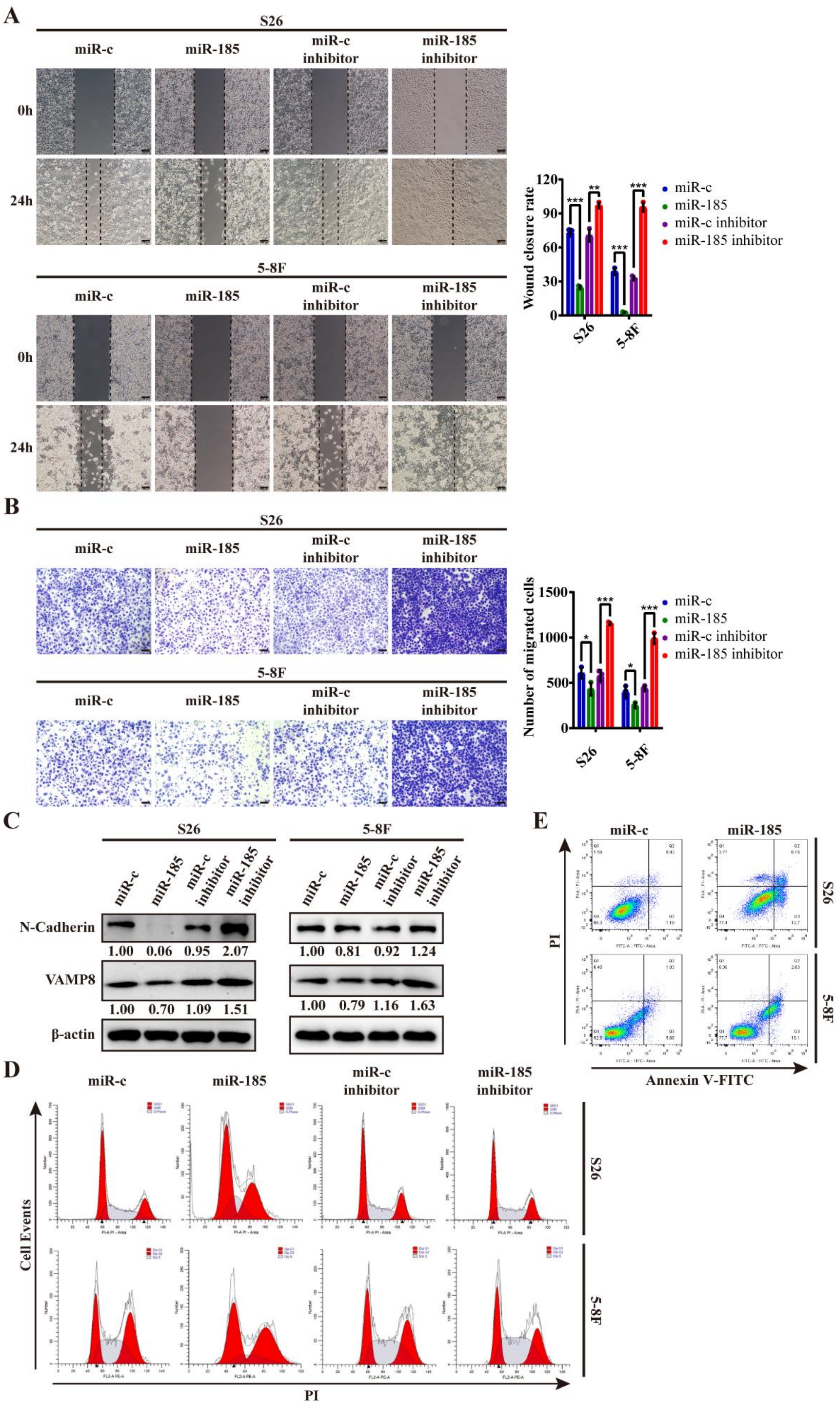
A
S26
5-8F
miR-c
miR-185
miR-c inhibitor
miR-185 inhibitor
0h
24h
Wound closure rate
B
Number of migrated cells
C
N-Cadherin
1.00 0.06 0.95 2.07
1.00 0.81 0.92 1.24
VAMP8
1.00 0.70 1.09 1.51
1.00 0.79 1.16 1.63
β-actin
D
Cell Events
PI
E
Annexin V-FITC

Figure S7. miR-185 inhibits the migration, induces G2/M cell cycle arrest and promotes the apoptosis of NPC cells. (A) Representative images of wound healing assay for the cells described in Figure 4A. Wound healing was monitored at different time points, with statistical analysis of wound closure rate shown at the right. Scale bar, 100 μm. **(B)** Representative images of transwell assay for the cells described in Figure 4A. Statistical results of migrated cell numbers are shown at the right. Scale bar, 100 μm. **(C)** Western blot assay showing the protein levels of N-Cadherin and VAMP8 in the cells described in Figure 4A. β-actin was used as control. **(D)** Representative images of flow cytometry analysis for cell cycle distribution in the cells described in Figure 4A, as supplement data for Figure 4F. x- and y- axes denote DNA content and cell number, respectively. Each cell cycle phase was calculated by ModFit LT 5.0 software. **(E)** Representative images of flow cytometry analysis for cell apoptosis in the cells described in Figure 4A, as supplement data for Figure 4G. x- and y- axes denote Annexin V-FITC staining and PI staining, respectively. The proportion of apoptotic cells was calculated by FlowJo software. *$P < 0.05$, **$P < 0.01$, and ***$P < 0.001$. All statistical analyses were performed using two-tailed Student's t-test.

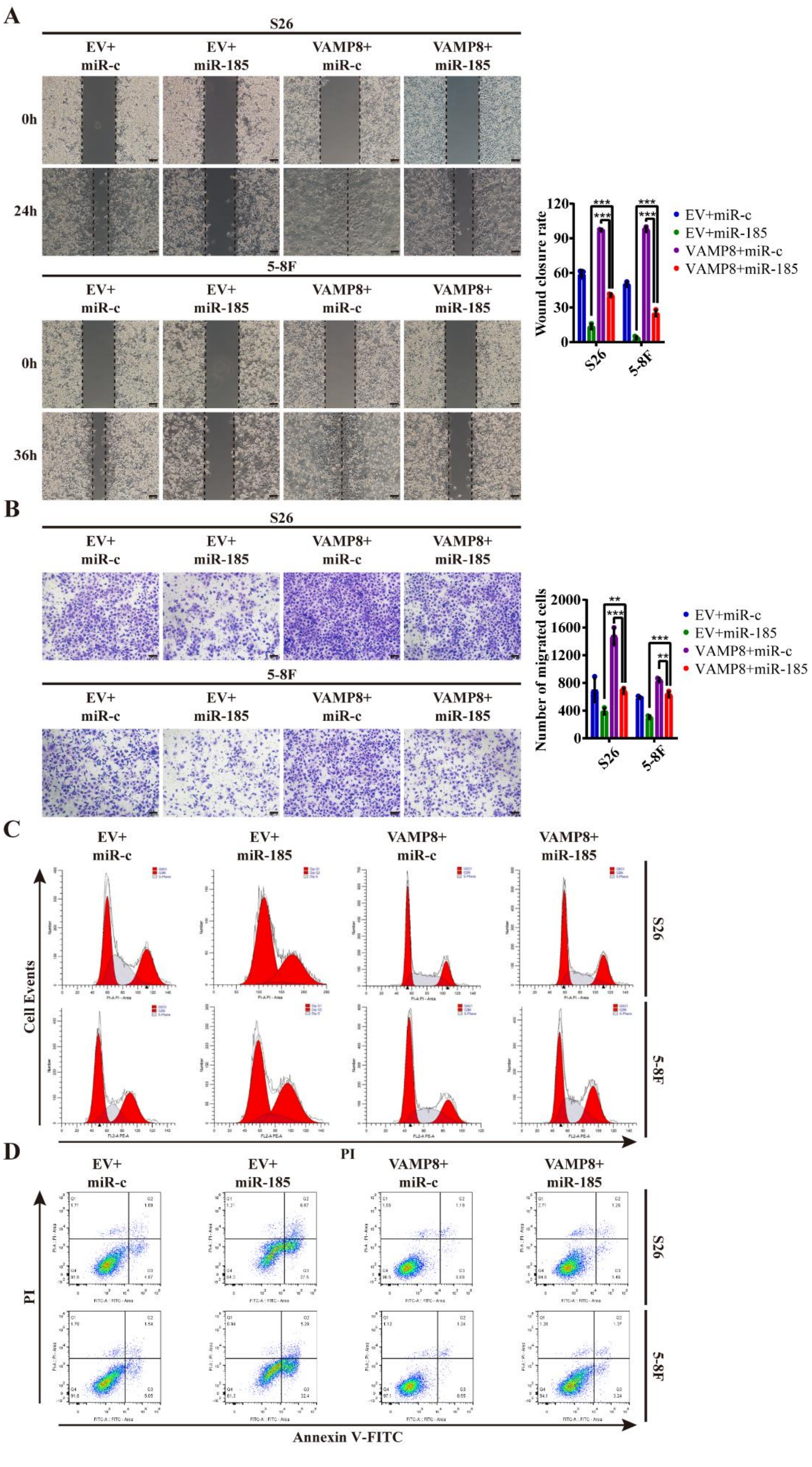
A
S26
EV+ miR-c
EV+ miR-185
VAMP8+ miR-c
VAMP8+ miR-185
0h
24h
5-8F
0h
36h
Wound closure rate
S26
5-8F
EV+miR-c
EV+miR-185
VAMP8+miR-c
VAMP8+miR-185
B
Number of migrated cells
C
Cell Events
PI
D
PI
Annexin V-FITC

Figure S8. VAMP8 overexpression rescues the migration and proliferation inhibitory effect of miR-185 on NPC cells. (A) Representative images of wound healing assay for the cells described in Figure 5A. Wound healing was monitored at different time points, with statistical results of wound closure rate shown at the right. Scale bar, 100 μm. **(B)** Representative images of transwell assay for the cells described in Figure 5A. Statistics results of migrated cell numbers are shown at the right. Scale bar, 100 μm. **(C)** Representative images of flow cytometry analysis for cell cycle distribution in the cells described in Figure 5A, as supplement data for Figure 5F. x- and y- axes denote DNA content and cell number, respectively. Each cell cycle phase was calculated by ModFit LT 5.0 software. **(D)** Representative images of flow cytometry analysis for cell apoptosis in the cells described in Figure 5A, as supplement data for Figure 5G. x- and y- axes denote Annexin V-FITC staining and PI staining, respectively. The proportion of apoptotic cells was calculated by FlowJo software. *P < 0.05, **P < 0.01, and ***P < 0.001. All statistical analyses were performed using two-tailed Student's t-test.

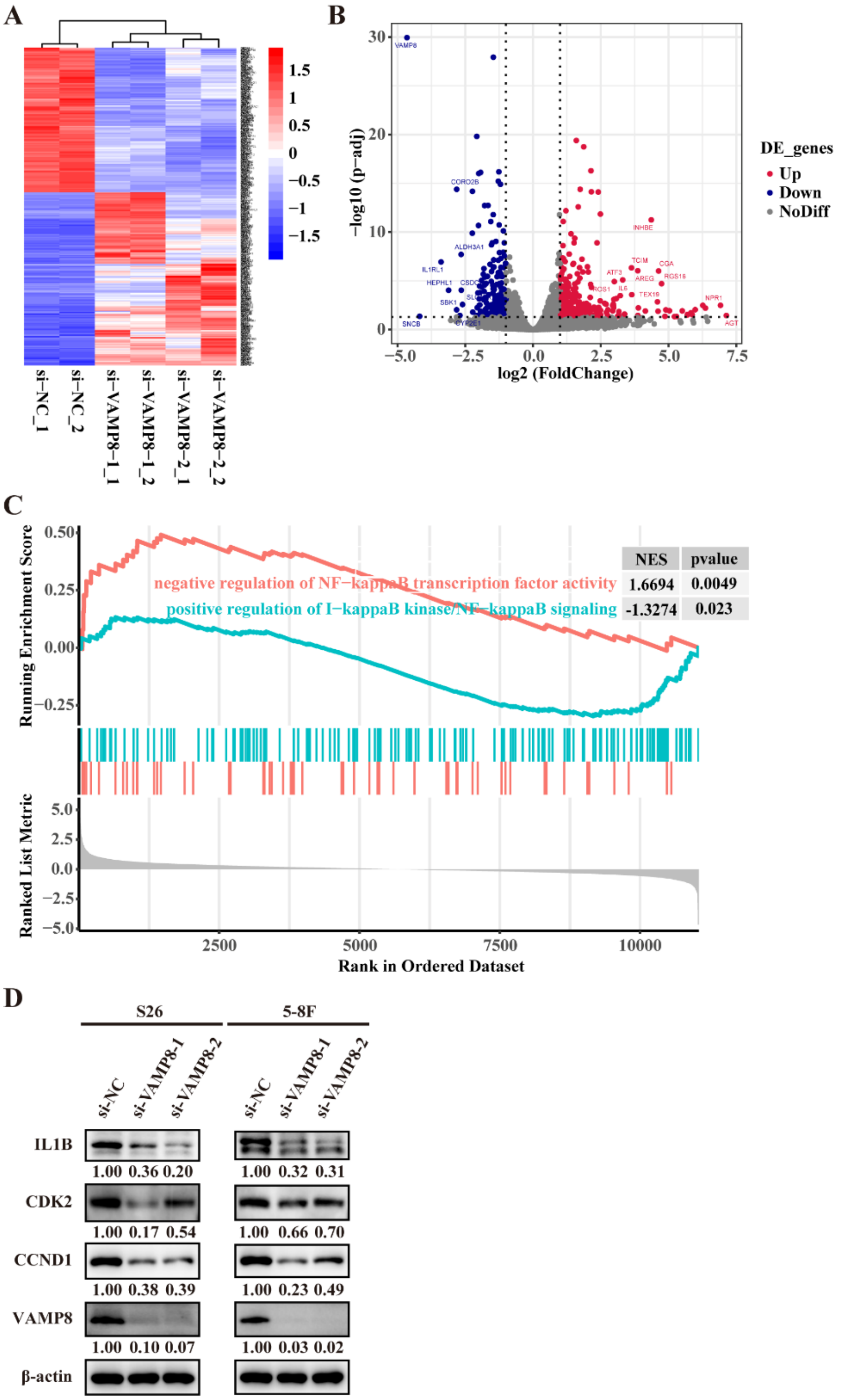
A
1.5
1
0.5
0
−0.5
−1
−1.5
si−NC_1
si−NC_2
si−VAMP8−1_1
si−VAMP8−1_2
si−VAMP8−2_1
si−VAMP8−2_2
B
−log10 (p−adj)
log2 (FoldChange)
VAMP8
CORO2B
ALDH3A1
IL1RL1
HEPHL1
SBK1
SNCB
INHBE
TCIM
CGA
ATF3
AREG
RGS16
IL6
TEX19
NPR1
AGT
DE_genes
Up
Down
NoDiff
C
Running Enrichment Score
NES
pvalue
negative regulation of NF−kappaB transcription factor activity
1.6694
0.0049
positive regulation of I−kappaB kinase/NF−kappaB signaling
-1.3274
0.023
Ranked List Metric
Rank in Ordered Dataset
D
S26
5-8F
si-NC
si-VAMP8-1
si-VAMP8-2
IL1B
1.00 0.36 0.20
1.00 0.32 0.31
CDK2
1.00 0.17 0.54
1.00 0.66 0.70
CCND1
1.00 0.38 0.39
1.00 0.23 0.49
VAMP8
1.00 0.10 0.07
1.00 0.03 0.02
β-actin

Figure S9. VAMP8 knockdown suppresses NF-κB activity. (A) Heatmap of significantly differentially expressed genes (DEGs) after knocking down VAMP8 in S26 cells. The filtering criteria for significantly DEGs are P-adj < 0.05 and |log2FoldChange| > 1. **(B)** Volcano plot for DEGs. Significantly up and down-regulated genes were filtered (P-adj < 0.05 and |log2FoldChange| > 1) and highlighted in red and blue dots, respectively. Genes that are not differentially expressed were highlighted in grey dots. **(C)** Gene set enrichment analysis (GSEA) of transcriptome data of S26 cells with VAMP8 knockdown and control. **(D)** Western blot assay showing the protein levels of IL1B, CDK2, CCND1 and VAMP8 in S26 and 5-8F cells. These cells were transfected with siRNA duplexes targeted VAMP8 or scrambled siRNAs. β-actin was used as control.

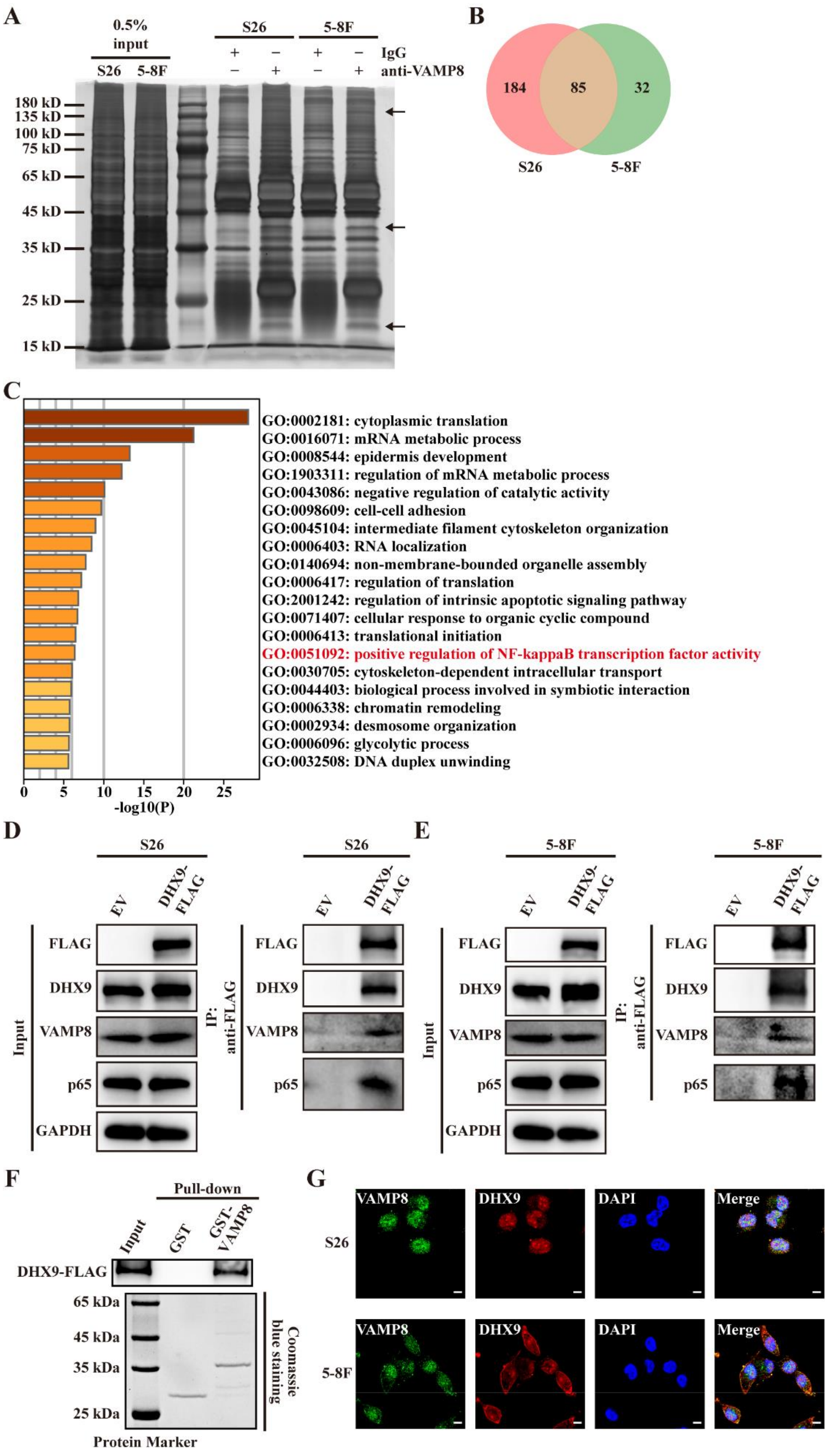
A
0.5% input
S26
5-8F
IgG
anti-VAMP8
180 kD
135 kD
100 kD
75 kD
65 kD
45 kD
35 kD
25 kD
15 kD
B
184
85
32
S26
5-8F
C
GO:0002181: cytoplasmic translation
GO:0016071: mRNA metabolic process
GO:0008544: epidermis development
GO:1903311: regulation of mRNA metabolic process
GO:0043086: negative regulation of catalytic activity
GO:0098609: cell-cell adhesion
GO:0045104: intermediate filament cytoskeleton organization
GO:0006403: RNA localization
GO:0140694: non-membrane-bounded organelle assembly
GO:0006417: regulation of translation
GO:2001242: regulation of intrinsic apoptotic signaling pathway
GO:0071407: cellular response to organic cyclic compound
GO:0006413: translational initiation
GO:0051092: positive regulation of NF-kappaB transcription factor activity
GO:0030705: cytoskeleton-dependent intracellular transport
GO:0044403: biological process involved in symbiotic interaction
GO:0006338: chromatin remodeling
GO:0002934: desmosome organization
GO:0006096: glycolytic process
GO:0032508: DNA duplex unwinding
0 5 10 15 20 25
-log10(P)
D
S26
EV
DHX9-FLAG
Input
FLAG
DHX9
VAMP8
p65
GAPDH
IP: anti-FLAG
E
5-8F
F
Pull-down
Input
GST
GST-VAMP8
DHX9-FLAG
65 kDa
45 kDa
35 kDa
25 kDa
Protein Marker
Coomassie blue staining
G
VAMP8
DHX9
DAPI
Merge
S26
5-8F

Figure S10. VAMP8 interacts with DHX9. (A) Silver staining results of immunoprecipitation in NPC cells. S26 and 5-8F cell lysates were immunoprecipitated with immunoglobulin G (IgG) or an anti-VAMP8 antibody and subsequently subjected to silver staining. The black arrows indicated protein bands specifically bound to VAMP8. **(B)** Mass spectrometry (MS) results showing the number of proteins in the protein bands indicated by the black arrows in (A). **(C)** Significant Gene Ontology (GO) processes enriched by using Metascape for total 301 proteins from MS analysis. **(D, E)** Western blotting assays for DHX9, VAMP8 and p65 expression in NPC cells. Cell lysates from S26 **(D)** and 5-8F **(E)** cells transfected with plasmid expressing DHX9-FLAG or empty vector were immunoprecipitated with anti-FLAG antibody and subsequently subjected to western blot assay with antibodies for FLAG tag, DHX9, VAMP8 and p65. GAPDH was used as control. **(F)** GST-fused VAMP8 and GST control proteins were expressed and purified from *Escherichia coli* strain BL21. The purified recombinant proteins were subsequently incubated with lysates derived from 293T cells overexpressing DHX9-FLAG. Following the pulldown using Glutathione Sepharose 4B, western blot analysis was performed to assess the interaction between VAMP8 and DHX9 (upper panel). Additionally, Coomassie blue staining confirmed the successful purification of GST (26 kDa) and GST-VAMP8 (37 kDa) proteins from *E. coli* (lower panel). **(G)** Immunofluorescent staining showed the colocalization of endogenous VAMP8 (green) and DHX9 (red) in S26 and 5-8F cells. Nuclei were stained with DAPI (blue). Scale bar, 10 μm.

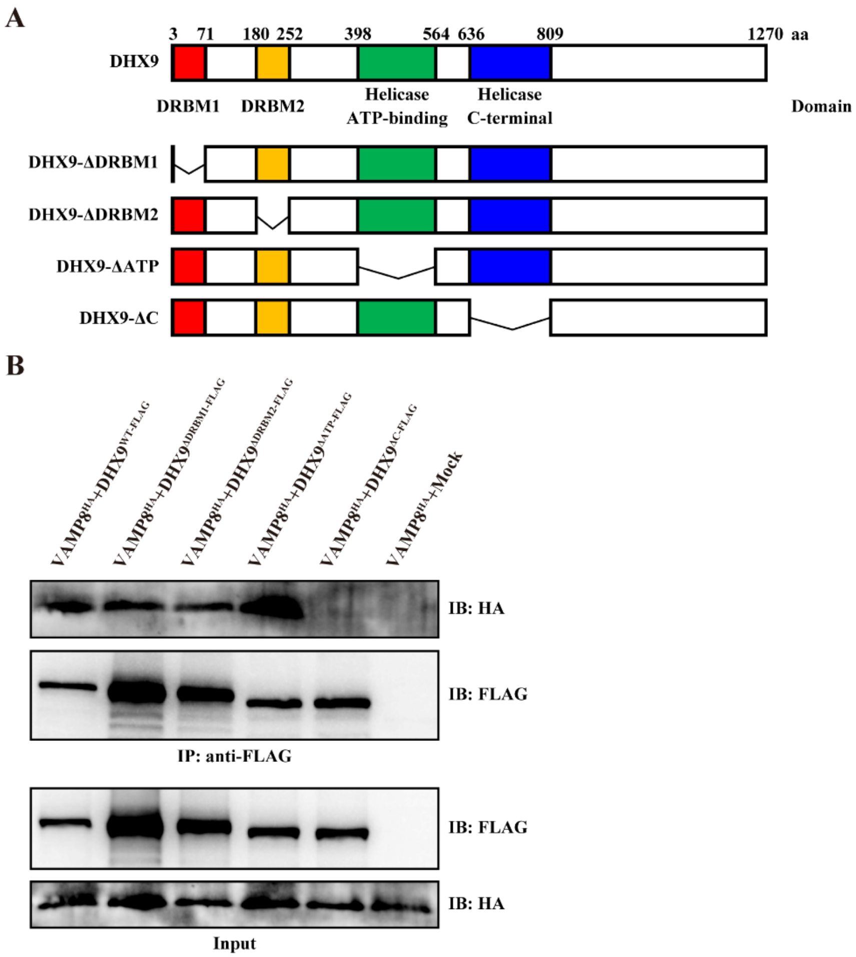
A
3
71
180
252
398
564
636
809
1270
aa
DHX9
DRBM1
DRBM2
Helicase
ATP-binding
Helicase
C-terminal
Domain
DHX9-ΔDRBM1
DHX9-ΔDRBM2
DHX9-ΔATP
DHX9-ΔC
B
VAMP8HA+DHX9WT-FLAG
VAMP8HA+DHX9ΔDRBM1-FLAG
VAMP8HA+DHX9ΔDRBM2-FLAG
VAMP8HA+DHX9ΔATP-FLAG
VAMP8HA+DHX9ΔC-FLAG
VAMP8HA+Mock
IB: HA
IB: FLAG
IP: anti-FLAG
IB: FLAG
IB: HA
Input

Figure S11. VAMP8 binds to the helicase C-terminal domain of DHX9. (A) Schematic diagram of the domain structure of wild-type DHX9 and its mutants. DHX9-ΔDRBM1, lacking the double-stranded RNA-binding motif 1 domain; DHX9-ΔDRBM2, lacking the double-stranded RNA-binding motif 2 domain; DHX9-ΔATP, lacking the helicase ATP-binding domain; DHX9-ΔC, lacking the helicase C-terminal domain. **(B)** Co-IP analysis of VAMP8 and DHX9. 293T cell lysates co-transfected with VAMP8-HA and either DHX9-WT-FLAG, DHX9-ΔDRBM1-FLAG, DHX9-ΔDRBM2-FLAG, DHX9-ΔATP-FLAG, DHX9-ΔC-FLAG, or the FLAG empty vector were immunoprecipitated using an anti-FLAG antibody. The immunoprecipitants were analyzed by western blot with antibodies against FLAG and HA tags to detect DHX9 and VAMP8, respectively.

Table S1. Expression-disease associations for genes located at genomic loci within 1Mb of any previous identified NPC risk variants but not yet implicated as target genes of risk variants[a]

Gene	Chr	Start	End	Type[b]	Model[c]	Top eQTL SNP[d]	TWAS Z[e]	TWAS P[f]
ZNF311	6	28962562	28973093	protein_coding	GTEx.Esophagus_Mucosa -MetaXcan	-	6.18	6.24×10^{-10}
ZFP57	6	29640169	29648887	protein_coding	GTEx.Esophagus_Mucosa -MetaXcan	-	5.74	9.45×10^{-9}
					GTEx.Esophagus_Mucosa -FUSION	rs375984	6.52	7.18×10^{-11}
HLA-F-AS1	6	29694378	29716826	lncRNA	GTEx.Esophagus_Mucosa -FUSION	rs4085262	-9.24	2.40×10^{-20}
HLA-G	6	29794744	29798902	protein_coding	GTEx.Esophagus_Mucosa -MetaXcan	-	8.85	8.69×10^{-19}
ZNRD1	6	30026676	30032686	protein_coding	GTEx.Esophagus_Mucosa -FUSION	rs9257940	-9.63	5.80×10^{-22}
GTF2H4	6	30875984	30881883	protein_coding	GTEx.Esophagus_Mucosa -MetaXcan	-	-5.43	5.75×10^{-8}
					GTEx.Esophagus_Mucosa -FUSION	rs1265063	-9.20	3.74×10^{-20}
HCG22	6	31021227	31027667	lncRNA	GTEx.Esophagus_Mucosa -FUSION	rs9263719	-7.96	1.77×10^{-15}
PSORS1C1	6	31082527	31107869	protein_coding	GTEx.Esophagus_Mucosa -FUSION	rs1042147	-4.65	3.26×10^{-6}
POU5F1	6	31132114	31148508	protein_coding	GTEx.Esophagus_Mucosa -FUSION	rs1265098	-6.27	3.60×10^{-10}
ATP6V1G2	6	31512239	31516204	protein_coding	GTEx.Esophagus_Mucosa -MetaXcan	-	-5.24	1.57×10^{-7}
					GTEx.Esophagus_Mucosa -FUSION	rs2239705	-5.37	7.89×10^{-8}
BAG6	6	31606805	31620482	protein_coding	GTEx.Esophagus_Mucosa -FUSION	rs1150793	-5.90	3.65×10^{-9}
MSH5	6	31707454	31730455	protein_coding	GTEx.Esophagus_Mucosa -MetaXcan	-	-5.41	6.16×10^{-8}
					GTEx.Esophagus_Mucosa -FUSION	rs1800629	-5.19	2.15×10^{-7}
HLA-DMA	6	32916390	32936871	protein_coding	GTEx.Esophagus_Mucosa -FUSION	rs2854275	4.94	8.00×10^{-7}

a Risk variants locating at last 1Mbp outside of any previously identified loci in previous GWAS or fine-mapping studies.
b lncRNA: long non-coding RNAs.

c The models were based on GTEx esophagus mucosa tissue dataset and MetaXcan or FUSION software.
d Variant most significantly associated with the gene expression from FUSION result; -: not available from MetaXcan result.
e Direction and effect size. Positive and negative value suggest associations of up-regulated and down-regulated predicted gene expression with increased NPC risk, respevtively.
f Significant association *P* values. A significance threshold is set as $P \leq 6.49 \times 10^{-6}$ and $P \leq 7.54 \times 10^{-6}$ for Bonferroni correction of 8,597 tests (0.05/7,709) in MetaXcan and 6,631 tests (0.05/6,631) in FUSION, respectively.

Table S2. Expression-disease associations for genes previously reported as potential target genes of identified NPC risk variants or genes harboring risk variants[a]

Gene	Chr	Start	End	Type	Model[b]	Top eQTL SNP[c]	TWAS Z[d]	TWAS P[e]
TERT	5	1253262	1295162	protein_coding	GTEx.Esophagus_Mucosa-MetaXcan	-	4.83	1.35×10^{-6}
					GTEx.Esophagus_Mucosa-FUSION	rs452384	5.14	2.82×10^{-7}
CLPTM1L	5	1317867	1345214	protein_coding	GTEx.Esophagus_Mucosa-MetaXcan	-	4.80	1.58×10^{-6}
HLA-F	6	29690552	29706305	protein_coding	GTEx.Esophagus_Mucosa-MetaXcan	-	-5.48	4.21×10^{-8}
					GTEx.Esophagus_Mucosa-FUSION	rs4085262	-4.81	1.55×10^{-6}

a Risk variants locating at last 1Mbp outside of any previously identified loci in previous GWAS or fine-mapping studies.

b The models were based on GTEx esophagus mucosa tissue dataset and MetaXcan or FUSION software.

c Variant most significantly associated with the gene expression from FUSION result; -: not available from MetaXcan result.

d Direction and effect size. Positive and negative value suggest associations of up-regulated and down-regulated predicted gene expression with increased NPC risk, respevtively.

e Significant association *P* values. A significance threshold is set as $P \leqslant 6.49 \times 10^{-6}$ and $P \leqslant 7.54 \times 10^{-6}$ for Bonferroni correction of 8,597 tests (0.05/7,709) in MetaXcan and 6,631 tests (0.05/6,631) in FUSION, respectively.

Table S3. The correlation *P* values among mRNA expression levels of the nearby genes identified by TWAS at 2p11.2[a]

Dataset		GGCX	VAMP8	VAMP5	RNF181	TMEM150A
Public GTEx esophagus_mucosa tissue (N=444)	GGCX	-	0.251	<0.001	<0.001	<0.001
	VAMP8		-	0.013	<0.001	0.640
	VAMP5			-	<0.001	<0.001
	RNF181				-	0.009
	TMEM150A					-
In-house NPC tumor tissue samples (N=206)	GGCX	-	<0.001	<0.001	<0.001	<0.001
	VAMP8		-	<0.001	<0.001	<0.001
	VAMP5			-	<0.001	<0.001
	RNF181				-	<0.001
	TMEM150A					-

a Correlation *P* values were based on Pearson's correlation test. *P* < 0.001 are bolded.

Table S4. The results of TWAS signals in summary-based joint/conditional testing implemented in FUSION

Gene	TWAS Z[a]	TWAS P[a]	COND Z[b]	COND P[b]	Indenpendent
TCF7L1	-2.81	4.88×10^{-3}	0.35	7.25×10^{-1}	FALSE
GGCX	-5.01	5.28×10^{-7}	0.48	6.30×10^{-1}	FALSE
VAMP8	5.50	3.60×10^{-8}	5.50	3.60×10^{-8}	TURE
RNF181	4.61	4.01×10^{-6}	-0.48	6.33×10^{-1}	FALSE
TMEM150A	2.30	2.00×10^{-2}	-1.30	1.94×10^{-1}	FALSE
VAMP5	-2.70	6.90×10^{-3}	-2.70	6.90×10^{-3}	FALSE

a The Z and P value of genes from original TWAS analysis implemented in FUSION using GTEx.Esophagus_Mucosa-FUSION models.

b The Z and P value of genes from summary-based joint/conditional testing implemented in FUSION using GTEx.Esophagus_Mucosa-FUSION models.

Table S5. The results of GWAS signals in the summary-based joint/conditional testing implemented in FUSION

CHR	SNP	POS	GWAS Z[a]	GWAS P[b]	COND Z[a]	COND P[b]
2	rs13002679	84800898	-0.72	4.70E-01	-0.48	6.35E-01
2	rs7603826	84803513	3.17	1.50E-03	3.16	1.60E-03
2	rs2018321	84807931	1.62	1.10E-01	1.65	9.82E-02
2	rs17759625	84815684	1.59	1.10E-01	1.64	1.02E-01
2	rs13013322	84823346	3.51	4.60E-04	3.28	1.00E-03
2	rs2365446	84829079	-0.70	4.80E-01	-0.45	6.52E-01
2	rs77932714	84840432	2.43	1.50E-02	2.14	3.28E-02
2	rs74677969	84840821	2.43	1.50E-02	2.14	3.28E-02
2	rs4513310	84855366	1.51	1.30E-01	1.55	1.21E-01
2	rs80339469	84869560	2.56	1.00E-02	2.22	2.62E-02
2	rs79207491	84882154	1.56	1.20E-01	1.59	1.11E-01
2	rs74723433	84887466	1.51	1.30E-01	1.49	1.35E-01
2	rs7600126	84890068	1.60	1.10E-01	1.63	1.03E-01
2	rs150296175	84892563	1.58	1.10E-01	1.61	1.08E-01
2	rs4832105	84893720	1.54	1.20E-01	1.57	1.17E-01
2	rs4832106	84899360	1.47	1.40E-01	1.50	1.34E-01
2	rs115433908	84899638	1.49	1.40E-01	1.47	1.41E-01
2	rs79176145	84900714	1.55	1.20E-01	1.56	1.20E-01
2	rs1918697	84900875	1.52	1.30E-01	1.55	1.21E-01
2	rs13407694	84902117	1.57	1.20E-01	1.55	1.21E-01
2	rs1918694	84902734	1.82	6.90E-02	1.85	6.37E-02
2	rs7578174	84905663	1.91	5.70E-02	1.98	4.77E-02
2	rs61315951	84905693	1.59	1.10E-01	1.38	1.68E-01
2	rs7564154	84905744	1.88	6.00E-02	1.96	5.04E-02
2	rs7590834	84905958	1.85	6.50E-02	1.92	5.43E-02
2	rs17025442	84906170	1.50	1.30E-01	1.26	2.06E-01
2	rs7567549	84906264	1.84	6.50E-02	1.91	5.55E-02
2	rs77295602	84906349	1.69	9.20E-02	1.49	1.37E-01
2	rs7581654	84906394	1.84	6.50E-02	1.91	5.55E-02
2	rs2178103	84906723	1.87	6.10E-02	1.92	5.51E-02
2	rs1918689	84906853	1.84	6.50E-02	1.91	5.55E-02
2	rs1918690	84906915	1.53	1.30E-01	1.60	1.09E-01
2	rs1918691	84906971	1.84	6.50E-02	1.91	5.55E-02
2	rs6749125	84907368	1.84	6.50E-02	1.91	5.55E-02
2	rs4832107	84908811	2.28	2.30E-02	2.25	2.47E-02
2	rs76459028	84909748	1.85	6.50E-02	1.89	5.81E-02
2	rs6729553	84909835	1.81	7.00E-02	1.88	5.95E-02
2	rs10520395	84909867	1.45	1.50E-01	1.24	2.13E-01
2	rs17025477	84913455	1.52	1.30E-01	1.32	1.87E-01
2	rs1192306	84913990	2.23	2.50E-02	2.20	2.77E-02
2	rs1192305	84914349	2.23	2.50E-02	2.20	2.77E-02
2	rs17025484	84916399	1.45	1.50E-01	1.25	2.13E-01
2	rs140426070	84920825	1.45	1.50E-01	1.25	2.13E-01

2	rs79745569	84924372	2.06	4.00E-02	2.06	3.91E-02
2	rs1192291	84924378	2.03	4.20E-02	2.00	4.54E-02
2	rs78190897	84924823	1.76	7.90E-02	1.79	7.37E-02
2	rs55645944	84926140	1.79	7.30E-02	1.83	6.70E-02
2	rs1192287	84926250	2.07	3.90E-02	2.04	4.16E-02
2	rs1192286	84926396	1.41	1.60E-01	1.27	2.03E-01
2	rs1192285	84926484	1.41	1.60E-01	1.27	2.03E-01
2	rs1192284	84927341	1.41	1.60E-01	1.27	2.03E-01
2	rs34110607	84927912	1.41	1.60E-01	1.27	2.03E-01
2	rs17025495	84927991	1.28	2.00E-01	1.08	2.78E-01
2	rs17025497	84929048	1.78	7.50E-02	1.82	6.80E-02
2	rs11889854	84929257	1.77	7.70E-02	1.80	7.11E-02
2	rs10178793	84929823	1.79	7.30E-02	1.83	6.70E-02
2	rs61750773	84932720	1.22	2.20E-01	1.02	3.09E-01
2	rs1192266	84936200	1.41	1.60E-01	1.27	2.03E-01
2	rs1918693	84938981	1.77	7.70E-02	1.81	7.06E-02
2	rs4832108	84940477	2.03	4.20E-02	1.98	4.78E-02
2	rs1728059	84941232	2.10	3.60E-02	2.07	3.82E-02
2	rs1192345	84943718	1.55	1.20E-01	1.40	1.61E-01
2	rs75176140	84943739	1.27	2.10E-01	1.07	2.86E-01
2	rs3810820	84945614	1.27	2.00E-01	1.07	2.85E-01
2	rs1192343	84945679	2.09	3.70E-02	2.06	3.97E-02
2	rs10199591	84946063	1.77	7.60E-02	1.81	6.99E-02
2	rs1192342	84946896	1.64	1.00E-01	1.50	1.34E-01
2	rs183566080	84947496	1.31	1.90E-01	1.12	2.64E-01
2	rs13386835	84947790	1.74	8.30E-02	1.77	7.64E-02
2	rs1192339	84948075	2.21	2.70E-02	2.18	2.91E-02
2	rs4832109	84948150	1.73	8.30E-02	1.77	7.73E-02
2	rs1192338	84948668	2.21	2.70E-02	2.18	2.91E-02
2	rs1427493	84948682	1.73	8.30E-02	1.77	7.73E-02
2	rs1192337	84949070	1.65	1.00E-01	1.50	1.32E-01
2	rs1192336	84950006	2.17	3.00E-02	2.14	3.24E-02
2	rs56156704	84950171	1.83	6.70E-02	1.82	6.81E-02
2	rs1192332	84951917	1.69	9.20E-02	1.54	1.23E-01
2	rs75124059	84955129	1.69	9.20E-02	1.70	8.90E-02
2	rs1192330	84955425	1.68	9.20E-02	1.54	1.23E-01
2	rs1192328	84956271	2.37	1.80E-02	2.32	2.04E-02
2	rs1192326	84958641	2.36	1.80E-02	2.31	2.11E-02
2	rs6705719	84958659	1.45	1.50E-01	1.25	2.12E-01
2	rs1192325	84959080	1.69	9.20E-02	1.54	1.22E-01
2	rs78528804	84959144	1.75	8.10E-02	1.75	7.93E-02
2	rs4832110	84961362	1.78	7.60E-02	1.79	7.40E-02
2	rs1192324	84961735	1.69	9.20E-02	1.54	1.22E-01
2	rs79110598	84962489	1.45	1.50E-01	1.25	2.12E-01
2	rs13386100	84963592	1.78	7.60E-02	1.79	7.40E-02
2	rs17025533	84967730	1.79	7.40E-02	1.80	7.21E-02

2	rs13407438	84967896	1.84	6.50E-02	1.85	6.38E-02
2	rs723134	84969260	1.45	1.50E-01	1.25	2.12E-01
2	rs10180799	84972910	1.76	7.90E-02	1.77	7.68E-02
2	rs1582847	84974603	2.38	1.70E-02	2.33	1.98E-02
2	rs76713005	84975933	1.78	7.60E-02	1.79	7.39E-02
2	rs1192272	84976080	2.39	1.70E-02	2.34	1.94E-02
2	rs13394245	84976150	0.60	5.50E-01	0.60	5.48E-01
2	rs890615	84978377	2.36	1.80E-02	2.31	2.07E-02
2	rs2365783	84978957	1.78	7.60E-02	1.79	7.39E-02
2	rs961343	84979327	2.39	1.70E-02	2.34	1.94E-02
2	rs75394965	84979908	1.45	1.50E-01	1.25	2.12E-01
2	rs6723303	84980717	1.78	7.60E-02	1.79	7.39E-02
2	rs6752065	84980786	1.78	7.60E-02	1.79	7.39E-02
2	rs111844909	84981239	1.78	7.60E-02	1.79	7.39E-02
2	rs1192350	84982251	2.35	1.90E-02	2.30	2.14E-02
2	rs78868251	84983745	1.75	8.10E-02	1.40	1.63E-01
2	rs79779393	84984271	1.45	1.50E-01	1.25	2.12E-01
2	rs6734517	84986249	1.74	8.20E-02	1.76	7.85E-02
2	rs13422612	84987181	1.78	7.60E-02	1.79	7.39E-02
2	rs1192382	84992011	1.69	9.20E-02	1.54	1.22E-01
2	rs117936874	84992509	1.45	1.50E-01	1.25	2.12E-01
2	rs17025543	84992658	1.86	6.30E-02	1.86	6.24E-02
2	rs73943396	84995084	1.46	1.50E-01	1.26	2.09E-01
2	rs78169976	84995639	1.46	1.50E-01	1.26	2.09E-01
2	rs6750190	84996052	1.87	6.10E-02	1.87	6.21E-02
2	rs1192379	84996296	2.62	8.70E-03	2.55	1.07E-02
2	rs13414180	84997344	1.87	6.10E-02	1.87	6.21E-02
2	rs1728036	84998646	2.43	1.50E-02	2.36	1.82E-02
2	rs1728102	85000730	2.43	1.50E-02	2.36	1.83E-02
2	rs13425682	85000982	1.87	6.10E-02	1.87	6.21E-02
2	rs13407222	85002590	1.87	6.10E-02	1.87	6.21E-02
2	rs78587480	85002874	1.46	1.40E-01	1.26	2.07E-01
2	rs13424151	85003618	1.87	6.10E-02	1.87	6.21E-02
2	rs147631533	85004136	1.46	1.40E-01	1.26	2.07E-01
2	rs1192378	85006211	2.43	1.50E-02	2.36	1.83E-02
2	rs7566739	85006710	1.42	1.60E-01	1.22	2.21E-01
2	rs1192377	85007158	2.43	1.50E-02	2.36	1.83E-02
2	rs1192375	85008373	1.88	6.10E-02	1.87	6.15E-02
2	rs1192374	85009024	1.19	2.30E-01	1.14	2.54E-01
2	rs1192373	85010878	1.88	6.10E-02	1.87	6.15E-02
2	rs1192372	85011546	2.33	2.00E-02	2.24	2.54E-02
2	rs73946004	85011776	1.46	1.40E-01	1.26	2.07E-01
2	rs1427490	85012156	2.43	1.50E-02	2.36	1.80E-02
2	rs1192316	85013202	1.87	6.10E-02	1.86	6.28E-02
2	rs1204440	85013566	2.53	1.10E-02	2.47	1.37E-02
2	rs58645050	85013662	1.40	1.60E-01	1.21	2.28E-01

2	rs78895235	85014460	1.52	1.30E-01	1.32	1.88E-01
2	rs1192314	85016517	1.88	6.10E-02	1.87	6.15E-02
2	rs1192312	85017662	2.35	1.90E-02	2.26	2.40E-02
2	rs137939880	85018772	1.52	1.30E-01	1.32	1.88E-01
2	rs1192311	85019331	1.90	5.70E-02	1.89	5.81E-02
2	rs1192310	85019680	2.40	1.60E-02	2.31	2.11E-02
2	rs17025551	85019906	1.42	1.60E-01	1.22	2.21E-01
2	rs1192309	85020144	1.97	4.90E-02	1.96	5.06E-02
2	rs1192308	85020329	2.40	1.60E-02	2.31	2.10E-02
2	rs17025556	85020702	2.40	1.60E-02	2.31	2.10E-02
2	rs1192402	85021070	2.40	1.60E-02	2.31	2.10E-02
2	rs1192401	85021085	2.40	1.60E-02	2.31	2.10E-02
2	rs1192400	85022274	2.40	1.60E-02	2.31	2.10E-02
2	rs754273	85023554	2.40	1.60E-02	2.31	2.10E-02
2	rs1192396	85024354	2.43	1.50E-02	2.34	1.93E-02
2	rs1192395	85024731	1.95	5.10E-02	1.94	5.22E-02
2	rs1192394	85026800	2.50	1.20E-02	2.43	1.52E-02
2	rs1192393	85026899	2.50	1.20E-02	2.43	1.52E-02
2	rs74644324	85026932	1.52	1.30E-01	1.32	1.88E-01
2	rs1007696	85027237	2.41	1.60E-02	2.31	2.08E-02
2	rs1192391	85027271	2.41	1.60E-02	2.31	2.08E-02
2	rs77804980	85029462	1.56	1.20E-01	1.36	1.75E-01
2	rs1192388	85029626	2.49	1.30E-02	2.41	1.59E-02
2	rs11688720	85031277	2.47	1.30E-02	2.40	1.62E-02
2	rs1192370	85031681	2.50	1.30E-02	2.43	1.53E-02
2	rs1192369	85033032	1.71	8.80E-02	1.64	1.01E-01
2	rs1192368	85033339	2.53	1.20E-02	2.46	1.41E-02
2	rs77653606	85034068	1.42	1.60E-01	1.21	2.26E-01
2	rs1192366	85035056	2.01	4.40E-02	2.00	4.53E-02
2	rs1192365	85037000	2.01	4.40E-02	2.00	4.53E-02
2	rs1192364	85037529	2.49	1.30E-02	2.40	1.65E-02
2	rs1192363	85038751	2.58	9.90E-03	2.51	1.21E-02
2	rs11687426	85039937	1.76	7.90E-02	1.61	1.08E-01
2	rs2276609	85040405	0.90	3.70E-01	0.92	3.55E-01
2	rs1192361	85040418	2.03	4.30E-02	2.02	4.35E-02
2	rs1192360	85041295	2.04	4.20E-02	2.03	4.26E-02
2	rs1192359	85041316	2.59	9.50E-03	2.52	1.16E-02
2	rs17025592	85041636	1.48	1.40E-01	1.27	2.03E-01
2	rs1192358	85042336	2.04	4.20E-02	2.03	4.26E-02
2	rs1192357	85042621	2.52	1.20E-02	2.43	1.52E-02
2	rs1192356	85044104	2.58	1.00E-02	2.51	1.22E-02
2	rs7584703	85046918	1.47	1.40E-01	1.27	2.05E-01
2	rs1192353	85047344	2.60	9.30E-03	2.53	1.14E-02
2	rs1192352	85049982	2.04	4.10E-02	2.04	4.17E-02
2	rs11674022	85050488	1.67	9.60E-02	1.51	1.30E-01
2	rs1649292	85051122	2.58	9.90E-03	2.47	1.34E-02

2	rs17025621	85052293	1.48	1.40E-01	1.28	2.02E-01
2	rs57396217	85052340	1.48	1.40E-01	1.28	2.02E-01
2	rs1649291	85052808	2.43	1.50E-02	2.42	1.53E-02
2	rs1627220	85053456	2.25	2.40E-02	2.23	2.60E-02
2	rs11684585	85053679	2.38	1.70E-02	2.38	1.74E-02
2	rs28714646	85053839	1.48	1.40E-01	1.28	2.01E-01
2	rs1192282	85054382	2.38	1.70E-02	2.38	1.74E-02
2	rs1192281	85054593	2.41	1.60E-02	2.41	1.61E-02
2	rs75346087	85055500	0.79	4.30E-01	0.83	4.07E-01
2	rs77937401	85055743	1.51	1.30E-01	1.31	1.92E-01
2	rs1192277	85055822	2.25	2.50E-02	2.23	2.57E-02
2	rs1192276	85057399	2.32	2.00E-02	2.31	2.09E-02
2	rs117271784	85058458	1.40	1.60E-01	1.20	2.29E-01
2	rs1192273	85059063	2.58	9.80E-03	2.67	7.60E-03
2	rs72926830	85062110	2.09	3.70E-02	1.89	5.87E-02
2	rs74389143	85063627	1.33	1.80E-01	1.22	2.23E-01
2	rs1346341	85067159	1.91	5.60E-02	1.82	6.88E-02
2	rs6728804	85115111	1.26	2.10E-01	1.38	1.67E-01
2	rs6761287	85116141	1.12	2.60E-01	1.24	2.17E-01
2	rs56175709	85116434	1.37	1.70E-01	1.46	1.44E-01
2	rs6736747	85117089	1.24	2.10E-01	1.34	1.79E-01
2	rs4832120	85117824	1.33	1.80E-01	1.42	1.57E-01
2	rs4831986	85118080	1.33	1.80E-01	1.42	1.57E-01
2	rs4831987	85118124	1.33	1.90E-01	1.41	1.57E-01
2	rs724012	85118358	1.14	2.50E-01	1.25	2.13E-01
2	rs724013	85118459	1.25	2.10E-01	1.35	1.77E-01
2	rs2054458	85118804	1.37	1.70E-01	1.46	1.44E-01
2	rs4832121	85119497	1.37	1.70E-01	1.46	1.44E-01
2	rs1344144	85121388	1.23	2.20E-01	1.33	1.84E-01
2	rs4404282	85122199	1.24	2.10E-01	1.35	1.78E-01
2	rs4375877	85122314	1.24	2.10E-01	1.35	1.78E-01
2	rs4377349	85122407	1.24	2.10E-01	1.35	1.78E-01
2	rs2886472	85122503	1.24	2.10E-01	1.35	1.78E-01
2	rs12465497	85122548	1.24	2.20E-01	1.34	1.80E-01
2	rs12469751	85122586	1.24	2.10E-01	1.35	1.78E-01
2	rs12465478	85122615	1.24	2.10E-01	1.35	1.78E-01
2	rs12465572	85122925	1.37	1.70E-01	1.46	1.44E-01
2	rs13406430	85124047	1.16	2.50E-01	1.27	2.05E-01
2	rs12477637	85124078	1.18	2.40E-01	1.30	1.95E-01
2	rs13427388	85124131	1.17	2.40E-01	1.27	2.02E-01
2	rs12469611	85124209	1.12	2.60E-01	1.23	2.19E-01
2	rs12477813	85124399	1.27	2.10E-01	1.36	1.75E-01
2	rs12469726	85124466	1.15	2.50E-01	1.26	2.09E-01
2	rs12477824	85124572	1.23	2.20E-01	1.34	1.82E-01
2	rs12477856	85124681	1.19	2.40E-01	1.29	1.97E-01
2	rs12466013	85124756	1.19	2.40E-01	1.29	1.97E-01

2	rs12477962	85125033	1.14	2.50E-01	1.24	2.13E-01
2	rs13405915	85125526	1.19	2.40E-01	1.29	1.97E-01
2	rs13406023	85125593	1.19	2.40E-01	1.29	1.97E-01
2	rs13394151	85125627	1.31	1.90E-01	1.40	1.63E-01
2	rs6547580	85127192	1.25	2.10E-01	1.35	1.78E-01
2	rs6547581	85127275	1.25	2.10E-01	1.35	1.77E-01
2	rs13388085	85127645	1.38	1.70E-01	1.49	1.37E-01
2	rs9309619	85129346	1.30	1.90E-01	1.41	1.57E-01
2	rs13411704	85130215	1.36	1.70E-01	1.47	1.42E-01
2	rs6547582	85131577	1.26	2.10E-01	1.35	1.76E-01
2	rs58844675	85132767	1.77	7.70E-02	1.85	6.41E-02
2	rs13409738	85133861	1.07	2.80E-01	1.18	2.38E-01
2	rs4832127	85160423	0.22	8.20E-01	0.26	7.92E-01
2	rs7592408	85161360	0.23	8.20E-01	0.27	7.85E-01
2	rs6547583	85161731	0.23	8.20E-01	0.27	7.84E-01
2	rs77017060	85161800	-0.26	7.90E-01	-0.27	7.89E-01
2	rs4359669	85162699	0.16	8.80E-01	0.19	8.46E-01
2	rs4832128	85163536	-0.09	9.30E-01	-0.04	9.66E-01
2	rs11899097	85164501	0.21	8.30E-01	0.24	8.09E-01
2	rs7575352	85165439	0.22	8.30E-01	0.25	8.02E-01
2	rs6728755	85166120	0.22	8.30E-01	0.25	8.02E-01
2	rs4832129	85167040	0.22	8.30E-01	0.25	8.05E-01
2	rs1979403	85168571	-0.06	9.60E-01	-0.01	9.95E-01
2	rs6726213	85169420	0.19	8.50E-01	0.22	8.26E-01
2	rs56187027	85171014	-0.04	9.70E-01	0.00	9.97E-01
2	rs7591327	85173517	-0.04	9.70E-01	0.01	9.91E-01
2	rs2886506	85173984	0.03	9.80E-01	0.06	9.52E-01
2	rs6737758	85177955	0.00	1.00E+00	0.03	9.75E-01
2	rs6737853	85178007	-0.07	9.50E-01	-0.02	9.87E-01
2	rs72928835	85178194	0.01	1.00E+00	0.04	9.65E-01
2	rs76700701	85178611	-0.07	9.40E-01	-0.04	9.67E-01
2	rs17025737	85181724	-0.11	9.10E-01	-0.08	9.39E-01
2	rs4832130	85182022	0.05	9.60E-01	0.08	9.37E-01
2	rs3884259	85185886	0.10	9.20E-01	0.12	9.07E-01
2	rs10865476	85188624	-0.05	9.60E-01	-0.01	9.96E-01
2	rs6714113	85189147	-0.11	9.20E-01	-0.06	9.51E-01
2	rs936540	85190325	-0.04	9.70E-01	0.01	9.95E-01
2	rs7583930	85190907	-0.11	9.10E-01	-0.06	9.49E-01
2	rs12618058	85191146	-0.09	9.30E-01	-0.06	9.54E-01
2	rs7572990	85191263	-0.08	9.30E-01	-0.03	9.72E-01
2	rs7599358	85191357	-0.11	9.10E-01	-0.08	9.35E-01
2	rs6547586	85191409	-0.17	8.70E-01	-0.14	8.86E-01
2	rs6547587	85191565	-0.05	9.60E-01	0.00	9.98E-01
2	rs76702861	85192847	-0.17	8.70E-01	-0.17	8.65E-01
2	rs72928861	85194193	-0.26	8.00E-01	-0.27	7.90E-01
2	rs59579068	85200677	-0.12	9.00E-01	-0.12	9.01E-01

2	rs1947785	85201198	-0.20	8.40E-01	-0.20	8.38E-01
2	rs17025763	85204425	-0.10	9.20E-01	-0.09	9.28E-01
2	rs17025765	85210234	-0.12	9.00E-01	-0.11	9.09E-01
2	rs78060911	85211405	-0.18	8.60E-01	-0.18	8.60E-01
2	rs6547588	85211432	0.29	7.70E-01	0.32	7.52E-01
2	rs79487529	85211856	-0.18	8.60E-01	-0.18	8.60E-01
2	rs17025770	85212658	-0.18	8.60E-01	-0.18	8.60E-01
2	rs17025771	85214123	-0.11	9.10E-01	-0.10	9.21E-01
2	rs56073933	85219442	-0.30	7.70E-01	-0.29	7.71E-01
2	rs74394764	85220768	-0.18	8.60E-01	-0.18	8.60E-01
2	rs11883641	85221110	-0.13	8.90E-01	-0.13	8.96E-01
2	rs56128602	85221714	-0.18	8.60E-01	-0.18	8.60E-01
2	rs56323234	85222334	-0.18	8.60E-01	-0.18	8.60E-01
2	rs3883936	85224246	-0.18	8.60E-01	-0.18	8.60E-01
2	rs75254301	85226313	-0.06	9.50E-01	-0.05	9.63E-01
2	rs12620326	85235001	-0.18	8.60E-01	-0.18	8.60E-01
2	rs57329805	85236610	-0.18	8.60E-01	-0.18	8.60E-01
2	rs114462151	85238127	-0.18	8.60E-01	-0.18	8.60E-01
2	rs12613871	85241835	0.01	9.90E-01	0.00	9.99E-01
2	rs56000191	85243783	-0.21	8.30E-01	-0.21	8.37E-01
2	rs137988243	85244238	-0.21	8.30E-01	-0.21	8.37E-01
2	rs55812335	85246106	-0.21	8.30E-01	-0.21	8.36E-01
2	rs17498115	85249295	-0.24	8.10E-01	-0.24	8.13E-01
2	rs80215069	85252080	-0.18	8.60E-01	-0.18	8.58E-01
2	rs56367002	85252792	-0.08	9.40E-01	-0.07	9.43E-01
2	rs56026694	85252797	-0.11	9.10E-01	-0.10	9.19E-01
2	rs11896509	85253059	-0.13	9.00E-01	-0.12	9.04E-01
2	rs75789682	85259152	-0.18	8.60E-01	-0.18	8.58E-01
2	rs76175650	85260323	-0.18	8.60E-01	-0.18	8.58E-01
2	rs75000390	85263856	-0.21	8.40E-01	-0.20	8.40E-01
2	rs7607064	85267356	-0.14	8.90E-01	-0.13	8.95E-01
2	rs3769766	85271558	-0.14	8.90E-01	-0.13	8.96E-01
2	rs60433576	85272379	-0.14	8.90E-01	-0.13	8.96E-01
2	rs3769765	85273149	-0.08	9.40E-01	-0.07	9.46E-01
2	rs17025830	85276866	-0.21	8.40E-01	-0.21	8.37E-01
2	rs17025837	85282879	-0.18	8.50E-01	-0.18	8.56E-01
2	rs79908510	85283395	-0.09	9.30E-01	-0.12	9.08E-01
2	rs10175216	85287777	-0.98	3.30E-01	-0.80	4.22E-01
2	rs1882271	85288572	-0.21	8.30E-01	-0.19	8.51E-01
2	rs2702028	85289624	-0.11	9.10E-01	-0.07	9.41E-01
2	rs115930007	85290008	0.72	4.70E-01	0.73	4.65E-01
2	rs116301790	85290975	0.69	4.90E-01	0.70	4.84E-01
2	rs2702031	85291839	-0.23	8.10E-01	-0.21	8.36E-01
2	rs2702032	85294918	1.56	1.20E-01	1.43	1.52E-01
2	rs76538561	85295018	-0.29	7.70E-01	-0.26	7.93E-01
2	rs2583550	85295212	1.46	1.40E-01	1.33	1.83E-01

2	rs4019832	85296388	0.32	7.50E-01	0.45	6.56E-01
2	rs113625787	85296601	0.35	7.30E-01	0.46	6.43E-01
2	rs72838164	85296781	0.46	6.40E-01	0.54	5.89E-01
2	rs55869261	85298955	0.25	8.00E-01	0.34	7.36E-01
2	rs62162805	85300366	0.29	7.70E-01	0.37	7.08E-01
2	rs2702033	85302530	1.44	1.50E-01	1.35	1.77E-01
2	rs59640363	85304912	-0.11	9.10E-01	-0.03	9.79E-01
2	rs11890007	85306122	0.14	8.90E-01	0.18	8.59E-01
2	rs118114015	85312888	0.49	6.20E-01	0.38	7.02E-01
2	rs147690904	85314764	0.46	6.40E-01	0.33	7.43E-01
2	rs77808282	85315514	-0.05	9.60E-01	-0.06	9.53E-01
2	rs12994253	85315647	-0.16	8.70E-01	-0.36	7.18E-01
2	rs114181978	85318612	0.36	7.20E-01	0.25	8.00E-01
2	rs7561093	85318807	0.80	4.20E-01	0.79	4.32E-01
2	rs7561211	85318939	0.98	3.30E-01	0.99	3.21E-01
2	rs2583570	85320215	0.72	4.70E-01	0.69	4.92E-01
2	rs4831989	85321051	1.01	3.10E-01	1.03	3.05E-01
2	rs6721254	85321298	0.48	6.30E-01	0.48	6.34E-01
2	rs2583569	85321746	1.10	2.70E-01	1.10	2.73E-01
2	rs2568227	85321901	1.00	3.20E-01	1.00	3.15E-01
2	rs56082832	85321922	0.48	6.30E-01	0.48	6.34E-01
2	rs142876549	85323065	0.40	6.90E-01	0.29	7.75E-01
2	rs79320324	85323405	0.48	6.30E-01	0.47	6.39E-01
2	rs2583568	85323685	0.82	4.10E-01	0.79	4.31E-01
2	rs55781382	85324338	0.44	6.60E-01	0.44	6.61E-01
2	rs113376782	85325453	0.98	3.30E-01	0.99	3.21E-01
2	rs2915659	85326827	0.75	4.50E-01	0.72	4.71E-01
2	rs2568212	85327431	0.75	4.50E-01	0.72	4.69E-01
2	rs2163646	85327888	0.77	4.40E-01	0.76	4.50E-01
2	rs2583563	85328480	0.97	3.30E-01	0.98	3.25E-01
2	rs2583561	85329092	0.92	3.60E-01	0.93	3.50E-01
2	rs115922588	85329093	0.21	8.30E-01	0.11	9.16E-01
2	rs2583559	85329317	0.81	4.20E-01	0.82	4.10E-01
2	rs2583558	85331425	0.61	5.50E-01	0.63	5.27E-01
2	rs2583557	85331663	0.59	5.50E-01	0.62	5.36E-01
2	rs2583556	85331686	0.26	7.90E-01	0.25	8.02E-01
2	rs4831990	85333390	-1.92	5.50E-02	-1.91	5.64E-02
2	rs4831991	85333421	0.34	7.40E-01	0.26	7.98E-01
2	rs75332039	85333634	0.67	5.00E-01	0.66	5.11E-01
2	rs4832136	85334034	0.23	8.10E-01	0.18	8.58E-01
2	rs6759185	85334209	0.25	8.00E-01	0.20	8.45E-01
2	rs57716843	85334277	0.25	8.00E-01	0.20	8.45E-01
2	rs57363422	85334310	0.20	8.50E-01	0.15	8.85E-01
2	rs56059339	85334923	-0.41	6.80E-01	-0.40	6.92E-01
2	rs56101385	85334927	-0.41	6.80E-01	-0.40	6.92E-01
2	rs10167862	85336421	0.13	9.00E-01	0.08	9.36E-01

2	rs10167871	85336432	0.18	8.60E-01	0.13	8.98E-01
2	rs10179005	85336492	0.13	9.00E-01	0.08	9.36E-01
2	rs147214385	85336521	0.64	5.20E-01	0.62	5.35E-01
2	rs10179118	85336639	0.13	9.00E-01	0.08	9.36E-01
2	rs59944734	85336757	0.13	9.00E-01	0.08	9.36E-01
2	rs10203218	85336862	0.13	9.00E-01	0.08	9.36E-01
2	rs10179383	85336868	0.13	9.00E-01	0.08	9.36E-01
2	rs13400944	85337089	0.13	9.00E-01	0.08	9.36E-01
2	rs10205874	85337409	0.13	9.00E-01	0.08	9.36E-01
2	rs10171611	85337781	0.13	9.00E-01	0.08	9.35E-01
2	rs10206436	85337782	0.13	9.00E-01	0.08	9.35E-01
2	rs115746090	85337816	0.87	3.80E-01	0.76	4.46E-01
2	rs13404546	85338146	0.12	9.00E-01	0.07	9.41E-01
2	rs79924122	85338381	0.87	3.80E-01	0.76	4.46E-01
2	rs77157377	85338560	-1.37	1.70E-01	-1.30	1.92E-01
2	rs3893079	85339019	0.64	5.20E-01	0.62	5.35E-01
2	rs7560476	85339385	0.12	9.00E-01	0.07	9.41E-01
2	rs7573011	85339546	0.12	9.00E-01	0.07	9.41E-01
2	rs11884056	85339839	0.14	8.90E-01	-0.01	9.96E-01
2	rs77036580	85340017	0.87	3.80E-01	0.76	4.46E-01
2	rs60467469	85340018	0.12	9.00E-01	0.07	9.41E-01
2	rs10496315	85340059	0.14	8.90E-01	0.09	9.31E-01
2	rs57727662	85340243	0.12	9.00E-01	0.07	9.41E-01
2	rs58467618	85340385	0.15	8.80E-01	0.10	9.19E-01
2	rs73943051	85340461	0.12	9.00E-01	0.07	9.41E-01
2	rs13387509	85340922	0.05	9.60E-01	0.00	9.99E-01
2	rs184091338	85341311	0.12	9.00E-01	0.07	9.41E-01
2	rs55895821	85341771	0.12	9.00E-01	0.07	9.41E-01
2	rs55804440	85342067	0.12	9.00E-01	0.07	9.41E-01
2	rs56163927	85342552	0.12	9.00E-01	0.07	9.41E-01
2	rs17025918	85342692	0.12	9.00E-01	0.07	9.41E-01
2	rs13394562	85342854	0.12	9.10E-01	0.07	9.47E-01
2	rs13394583	85343011	-1.37	1.70E-01	-1.30	1.92E-01
2	rs6706580	85343060	0.12	9.00E-01	0.07	9.41E-01
2	rs6709816	85343124	0.12	9.00E-01	0.07	9.41E-01
2	rs17711141	85343598	0.87	3.80E-01	0.76	4.46E-01
2	rs4831992	85343642	-1.37	1.70E-01	-1.30	1.92E-01
2	rs6721134	85344303	0.12	9.00E-01	0.07	9.41E-01
2	rs58016144	85344329	-1.37	1.70E-01	-1.31	1.91E-01
2	rs55658647	85344603	0.87	3.80E-01	0.76	4.46E-01
2	rs4832138	85344776	0.12	9.00E-01	0.07	9.41E-01
2	rs4832139	85344915	0.12	9.00E-01	0.07	9.41E-01
2	rs10167530	85345078	0.13	9.00E-01	0.08	9.36E-01
2	rs10167655	85345206	0.12	9.00E-01	0.07	9.41E-01
2	rs10167762	85345273	0.12	9.00E-01	0.07	9.41E-01
2	rs112091851	85345624	0.15	8.80E-01	0.11	9.16E-01

2	rs111541747	85345629	0.15	8.80E-01	0.11	9.16E-01
2	rs76397877	85345998	0.12	9.00E-01	0.07	9.41E-01
2	rs78163036	85346392	0.87	3.80E-01	0.76	4.46E-01
2	rs7606461	85346762	0.12	9.00E-01	0.07	9.41E-01
2	rs7567600	85347014	0.12	9.00E-01	0.07	9.41E-01
2	rs7606764	85347015	0.12	9.00E-01	0.07	9.41E-01
2	rs6733776	85347744	0.12	9.00E-01	0.07	9.41E-01
2	rs75975689	85347751	0.17	8.60E-01	0.12	9.03E-01
2	rs141544864	85348206	0.12	9.00E-01	0.07	9.41E-01
2	rs76069810	85348518	0.12	9.00E-01	0.07	9.41E-01
2	rs4832140	85348915	0.12	9.00E-01	0.07	9.41E-01
2	rs113487157	85349339	0.12	9.00E-01	0.07	9.41E-01
2	rs76470222	85349484	0.12	9.00E-01	0.07	9.41E-01
2	rs6713119	85349588	0.12	9.00E-01	0.07	9.41E-01
2	rs73943053	85349634	0.12	9.00E-01	0.07	9.41E-01
2	rs4832141	85349883	0.12	9.00E-01	0.07	9.41E-01
2	rs4832142	85349903	0.19	8.50E-01	0.14	8.88E-01
2	rs74968033	85350324	0.65	5.20E-01	0.62	5.33E-01
2	rs77747141	85350351	0.15	8.80E-01	0.10	9.23E-01
2	rs6745957	85350902	0.22	8.30E-01	0.17	8.65E-01
2	rs6720893	85351150	0.22	8.20E-01	0.18	8.60E-01
2	rs6721006	85351197	0.22	8.20E-01	0.18	8.60E-01
2	rs113238819	85351603	0.22	8.20E-01	0.18	8.60E-01
2	rs193077754	85351644	0.68	5.00E-01	0.62	5.36E-01
2	rs140909185	85351787	-1.25	2.10E-01	-1.19	2.36E-01
2	rs74354184	85352522	0.20	8.40E-01	0.14	8.91E-01
2	rs143172695	85353729	-0.07	9.50E-01	-0.21	8.36E-01
2	rs55681013	85353889	0.26	8.00E-01	0.19	8.51E-01
2	rs4832143	85354274	0.25	8.00E-01	0.19	8.51E-01
2	rs4831994	85354545	0.30	7.70E-01	0.23	8.19E-01
2	rs56024098	85354553	0.72	4.70E-01	0.67	5.03E-01
2	rs13416083	85355393	-1.20	2.30E-01	-1.13	2.58E-01
2	rs13428324	85355725	0.26	7.90E-01	0.18	8.57E-01
2	rs35255486	85356366	-0.22	8.30E-01	-0.35	7.30E-01
2	rs113436581	85356584	0.26	7.90E-01	0.18	8.56E-01
2	rs113227342	85356606	0.26	7.90E-01	0.18	8.56E-01
2	rs146684755	85356870	0.35	7.20E-01	0.27	7.88E-01
2	rs10202291	85357101	0.25	8.10E-01	0.15	8.77E-01
2	rs35276090	85358373	-0.31	7.60E-01	-0.41	6.78E-01
2	rs150720257	85360059	-0.57	5.70E-01	-0.69	4.89E-01
2	rs10195517	85364093	-2.01	4.40E-02	-2.09	3.68E-02
2	rs72838188	85364138	1.04	3.00E-01	0.99	3.20E-01
2	rs72838189	85364307	-1.23	2.20E-01	-1.28	1.99E-01
2	rs12474138	85364537	-2.40	1.60E-02	-2.37	1.78E-02
2	rs55984939	85364689	-1.21	2.20E-01	-1.33	1.85E-01
2	rs111462901	85364805	-1.32	1.90E-01	-1.41	1.58E-01

2	rs7584403	85365367	-1.20	2.30E-01	-1.31	1.91E-01
2	rs72838190	85365711	-1.14	2.60E-01	-1.24	2.16E-01
2	rs56168805	85366987	1.27	2.00E-01	1.16	2.48E-01
2	rs55974301	85367278	1.42	1.60E-01	1.33	1.84E-01
2	rs62162827	85367808	1.43	1.50E-01	1.29	1.98E-01
2	rs17762108	85370407	-0.80	4.30E-01	-0.96	3.36E-01
2	rs2568216	85372094	0.52	6.10E-01	0.39	6.99E-01
2	rs2583541	85372139	0.47	6.40E-01	0.34	7.32E-01
2	rs113082906	85373615	-0.98	3.30E-01	-1.15	2.49E-01
2	rs1808640	85373749	0.82	4.10E-01	0.83	4.06E-01
2	rs1808639	85373817	0.51	6.10E-01	0.38	7.04E-01
2	rs151147084	85373827	1.96	5.00E-02	1.83	6.78E-02
2	rs72838196	85373976	1.96	5.00E-02	1.83	6.78E-02
2	rs2248065	85374197	0.45	6.50E-01	0.32	7.53E-01
2	rs2248068	85374315	0.58	5.70E-01	0.44	6.60E-01
2	rs2248072	85374417	0.47	6.40E-01	0.34	7.33E-01
2	rs2248076	85374482	0.84	4.00E-01	0.86	3.88E-01
2	rs2248079	85374526	0.54	5.90E-01	0.40	6.90E-01
2	rs2568217	85375001	0.76	4.50E-01	0.78	4.33E-01
2	rs2568218	85375132	0.73	4.70E-01	0.75	4.52E-01
2	rs17025940	85375282	-1.08	2.80E-01	-1.22	2.24E-01
2	rs2568219	85375574	0.47	6.40E-01	0.33	7.40E-01
2	rs6754217	85375676	-1.08	2.80E-01	-1.22	2.24E-01
2	rs2436192	85376121	0.75	4.50E-01	0.77	4.41E-01
2	rs55886208	85376194	-0.99	3.20E-01	-1.17	2.42E-01
2	rs72838199	85377070	1.79	7.40E-02	1.66	9.79E-02
2	rs7570315	85377179	-1.08	2.80E-01	-1.22	2.24E-01
2	rs2568221	85377576	-0.22	8.20E-01	-0.33	7.41E-01
2	rs2568222	85377979	-0.36	7.20E-01	-0.47	6.41E-01
2	rs62162851	85378295	-1.05	3.00E-01	-1.22	2.21E-01
2	rs7560555	85378666	-0.99	3.20E-01	-1.17	2.42E-01
2	rs2568225	85379914	0.03	9.80E-01	-0.10	9.19E-01
2	rs55733107	85380504	1.33	1.80E-01	1.23	2.20E-01
2	rs17025951	85381076	-1.05	3.00E-01	-1.22	2.21E-01
2	rs72840107	85381467	1.33	1.80E-01	1.23	2.20E-01
2	rs62162853	85385438	-1.41	1.60E-01	-1.57	1.18E-01
2	rs77707396	85388993	0.31	7.50E-01	0.38	7.02E-01
2	rs74504675	85389185	0.45	6.50E-01	0.52	6.03E-01
2	rs1560585	85389705	0.18	8.60E-01	0.27	7.87E-01
2	rs75241717	85392975	0.22	8.20E-01	0.30	7.61E-01
2	rs77433347	85396058	0.14	8.90E-01	0.26	7.95E-01
2	rs17711688	85399316	-1.10	2.70E-01	-0.90	3.66E-01
2	rs150350898	85400749	0.71	4.80E-01	0.69	4.90E-01
2	rs17025989	85402493	0.29	7.80E-01	0.46	6.45E-01
2	rs113353353	85407570	0.26	7.90E-01	0.43	6.67E-01
2	rs7594695	85408298	0.14	8.90E-01	0.31	7.58E-01

2	rs7594708	85408364	0.31	7.60E-01	0.49	6.27E-01
2	rs72840120	85408966	0.15	8.80E-01	0.31	7.55E-01
2	rs78109461	85409060	0.34	7.40E-01	0.52	6.05E-01
2	rs76050564	85409300	0.33	7.40E-01	0.51	6.13E-01
2	rs77708381	85409359	0.33	7.40E-01	0.51	6.13E-01
2	rs6758096	85411399	0.21	8.40E-01	0.42	6.71E-01
2	rs11126982	85412776	0.09	9.30E-01	0.33	7.41E-01
2	rs61342907	85413175	-1.36	1.70E-01	-1.37	1.71E-01
2	rs13432342	85414647	-1.68	9.30E-02	-1.68	9.24E-02
2	rs117886899	85420225	0.37	7.10E-01	0.38	7.01E-01
2	rs10183679	85420530	-1.57	1.20E-01	-1.58	1.14E-01
2	rs4374376	85420702	0.08	9.30E-01	0.33	7.44E-01
2	rs880092	85420870	-1.57	1.20E-01	-1.58	1.14E-01
2	rs874838	85421691	-1.50	1.30E-01	-1.50	1.33E-01
2	rs13423910	85422983	-1.75	8.00E-02	-1.75	8.04E-02
2	rs6728057	85423414	0.11	9.20E-01	0.35	7.28E-01
2	rs61276534	85424825	0.11	9.10E-01	0.35	7.25E-01
2	rs57871178	85425048	-1.65	9.80E-02	-1.66	9.69E-02
2	rs6733735	85426269	0.10	9.20E-01	0.34	7.32E-01
2	rs6747032	85426463	0.10	9.20E-01	0.34	7.32E-01
2	rs56691726	85428394	0.10	9.20E-01	0.34	7.32E-01
2	rs10192927	85428860	-1.77	7.80E-02	-1.76	7.83E-02
2	rs62165563	85430588	-1.89	5.80E-02	-1.90	5.69E-02
2	rs17026022	85432460	-0.57	5.70E-01	-0.27	7.87E-01
2	rs72932687	85433402	-0.57	5.70E-01	-0.27	7.87E-01
2	rs4832148	85434540	1.65	9.90E-02	1.38	1.69E-01
2	rs4831995	85434612	-1.83	6.70E-02	-1.80	7.24E-02
2	rs17026034	85437484	-0.49	6.30E-01	-0.19	8.53E-01
2	rs4831997	85442488	-0.75	4.50E-01	-0.56	5.77E-01
2	rs4832149	85443369	-0.49	6.20E-01	-0.34	7.32E-01
2	rs13396934	85443820	-0.54	5.90E-01	-0.37	7.13E-01
2	rs6709476	85443924	-0.72	4.70E-01	-0.41	6.85E-01
2	rs80092311	85446292	-0.22	8.30E-01	0.08	9.35E-01
2	rs72932696	85446444	-0.34	7.30E-01	-0.05	9.64E-01
2	rs6547598	85446486	-0.50	6.20E-01	-0.18	8.58E-01
2	rs10179888	85449320	-0.14	8.90E-01	0.00	9.97E-01
2	rs4494743	85449498	-0.46	6.50E-01	-0.17	8.69E-01
2	rs13402421	85450361	-0.18	8.60E-01	-0.05	9.59E-01
2	rs6743132	85453593	0.28	7.80E-01	0.55	5.82E-01
2	rs72934610	85455675	0.50	6.20E-01	0.74	4.61E-01
2	rs11902445	85455966	0.17	8.60E-01	0.39	6.95E-01
2	rs113230662	85456241	-1.87	6.20E-02	-1.90	5.74E-02
2	rs6749942	85460510	0.14	8.90E-01	0.36	7.15E-01
2	rs6750175	85460679	0.09	9.30E-01	0.30	7.63E-01
2	rs7588165	85461359	0.40	6.90E-01	0.59	5.53E-01
2	rs72934626	85469657	0.47	6.40E-01	0.66	5.10E-01

2	rs6753357	85470105	0.65	5.10E-01	0.83	4.09E-01
2	rs6758805	85472055	-0.79	4.30E-01	-0.52	6.04E-01
2	rs74772985	85474425	-1.70	9.00E-02	-1.71	8.71E-02
2	rs62165594	85480011	0.82	4.10E-01	0.30	7.65E-01
2	rs17763748	85480107	0.82	4.20E-01	0.31	7.60E-01
2	rs7580690	85480422	0.17	8.70E-01	0.26	7.93E-01
2	rs17712901	85480425	0.58	5.60E-01	0.09	9.28E-01
2	rs62165595	85480661	0.60	5.50E-01	0.11	9.09E-01
2	rs17763853	85480715	0.64	5.20E-01	0.18	8.58E-01
2	rs7581133	85480817	0.56	5.80E-01	0.08	9.34E-01
2	rs11684211	85481524	0.66	5.10E-01	0.19	8.52E-01
2	rs78012323	85482746	0.25	8.00E-01	0.34	7.30E-01
2	rs2043229	85483003	0.66	5.10E-01	0.26	7.91E-01
2	rs17026108	85483145	0.37	7.10E-01	0.44	6.60E-01
2	rs79026474	85483396	1.33	1.90E-01	1.44	1.50E-01
2	rs72840024	85483704	0.73	4.60E-01	0.27	7.86E-01
2	rs78544253	85483809	1.63	1.00E-01	1.35	1.75E-01
2	rs11904127	85484818	0.73	4.70E-01	0.35	7.29E-01
2	rs11904105	85484959	0.78	4.40E-01	0.40	6.88E-01
2	rs11674662	85485915	-0.13	8.90E-01	-0.55	5.81E-01
2	rs2366264	85489011	-0.25	8.10E-01	-0.71	4.75E-01
2	rs1991738	85489280	-0.25	8.00E-01	-0.70	4.82E-01
2	rs11689667	85491365	-0.22	8.20E-01	-0.68	4.96E-01
2	rs6547607	85491420	-0.22	8.20E-01	-0.69	4.90E-01
2	rs883650	85491747	-0.24	8.10E-01	-0.66	5.07E-01
2	rs883651	85491770	-0.19	8.50E-01	-0.65	5.14E-01
2	rs908301	85492141	-0.18	8.60E-01	-0.65	5.17E-01
2	rs882883	85492391	-0.18	8.60E-01	-0.65	5.17E-01
2	rs1554109	85493002	-0.18	8.60E-01	-0.65	5.17E-01
2	rs7340255	85497900	0.01	9.90E-01	-0.50	6.15E-01
2	rs11675489	85498783	0.01	1.00E+00	-0.50	6.18E-01
2	rs62162671	85498886	0.28	7.80E-01	-0.22	8.26E-01
2	rs11686782	85498968	0.01	9.90E-01	-0.50	6.15E-01
2	rs55654088	85499562	-0.18	8.60E-01	-0.75	4.55E-01
2	rs7340132	85500278	-0.25	8.00E-01	-0.80	4.23E-01
2	rs10201321	85501073	-0.13	9.00E-01	-0.55	5.80E-01
2	rs10201483	85501384	-0.21	8.30E-01	-0.60	5.48E-01
2	rs10201660	85501390	-0.34	7.30E-01	-0.73	4.68E-01
2	rs10201489	85501409	-0.24	8.10E-01	-0.63	5.26E-01
2	rs4441469	85501999	-0.26	7.90E-01	-0.67	5.00E-01
2	rs12714137	85502264	1.73	8.50E-02	1.86	6.28E-02
2	rs12714138	85502419	-0.22	8.20E-01	-0.12	9.04E-01
2	rs6745529	85503139	-0.21	8.30E-01	-0.76	4.50E-01
2	rs10184690	85503568	-0.24	8.10E-01	-0.65	5.14E-01
2	rs10184810	85503674	-0.25	8.10E-01	-0.67	5.05E-01
2	rs4832151	85503996	-0.08	9.40E-01	0.04	9.67E-01

2	rs4832152	85504191	-0.19	8.50E-01	-0.60	5.47E-01
2	rs908300	85504704	-0.13	9.00E-01	-0.58	5.64E-01
2	rs882831	85504877	-0.13	9.00E-01	-0.58	5.64E-01
2	rs4346385	85504989	0.02	9.80E-01	0.09	9.31E-01
2	rs6747629	85505562	-0.15	8.80E-01	-0.60	5.52E-01
2	rs6733190	85505920	-0.18	8.60E-01	-0.73	4.67E-01
2	rs6733515	85506000	-0.15	8.80E-01	-0.60	5.48E-01
2	rs6736154	85506142	-0.25	8.10E-01	-0.66	5.06E-01
2	rs6736366	85506160	0.02	9.80E-01	0.08	9.33E-01
2	rs10165984	85506588	-0.19	8.50E-01	-0.63	5.26E-01
2	rs11126990	85507334	0.00	1.00E+00	0.07	9.45E-01
2	rs10204252	85507657	-0.26	7.90E-01	-0.68	4.93E-01
2	rs4832154	85508399	0.06	9.50E-01	-0.41	6.79E-01
2	rs4831998	85508454	0.29	7.70E-01	-0.18	8.60E-01
2	rs7598047	85510018	0.35	7.30E-01	0.50	6.19E-01
2	rs112134288	85512095	1.39	1.60E-01	1.58	1.15E-01
2	rs59353316	85521088	-1.77	7.70E-02	-1.46	1.44E-01
2	rs7583983	85521912	-0.16	8.70E-01	-0.63	5.30E-01
2	rs12471961	85522926	0.75	4.50E-01	0.11	9.13E-01
2	rs4832155	85523220	0.75	4.50E-01	0.11	9.13E-01
2	rs4832156	85523276	-0.25	8.10E-01	-0.71	4.78E-01
2	rs4832157	85523305	0.75	4.50E-01	0.11	9.13E-01
2	rs4832158	85523476	-0.17	8.70E-01	-0.63	5.27E-01
2	rs4832159	85523541	0.69	4.90E-01	0.06	9.56E-01
2	rs10178604	85524876	0.04	9.70E-01	-0.40	6.86E-01
2	rs10178705	85525035	0.82	4.10E-01	0.18	8.58E-01
2	rs75912143	85525339	0.04	9.70E-01	-0.39	6.98E-01
2	rs7564589	85525490	0.02	9.90E-01	-0.43	6.70E-01
2	rs10184914	85526399	0.81	4.20E-01	0.18	8.57E-01
2	rs9753285	85526806	-1.59	1.10E-01	-1.25	2.13E-01
2	rs8179703	85526913	0.17	8.70E-01	-0.28	7.77E-01
2	rs73943085	85527451	-1.59	1.10E-01	-1.27	2.04E-01
2	rs13001442	85529981	0.33	7.40E-01	-0.10	9.23E-01
2	rs73943088	85530027	-1.16	2.50E-01	-0.90	3.66E-01
2	rs13033165	85530065	1.13	2.60E-01	0.47	6.37E-01
2	rs10174520	85530561	0.77	4.40E-01	0.12	9.05E-01
2	rs12714139	85531807	0.37	7.10E-01	-0.23	8.22E-01
2	rs2271629	85533227	-1.29	2.00E-01	-1.46	1.44E-01
2	rs41288843	85534039	-1.32	1.90E-01	-0.94	3.45E-01
2	rs59059406	85534392	0.63	5.30E-01	0.01	9.95E-01
2	rs61326180	85534567	0.78	4.40E-01	0.13	8.93E-01
2	rs73943091	85534569	0.63	5.30E-01	0.01	9.94E-01
2	rs59653199	85534574	0.63	5.30E-01	0.01	9.95E-01
2	rs4641936	85535187	1.57	1.20E-01	0.96	3.36E-01
2	rs72934702	85535304	1.42	1.50E-01	0.84	4.01E-01
2	rs9248	85537312	0.95	3.40E-01	0.61	5.43E-01

2	rs13027998	85538995	0.91	3.60E-01	0.59	5.57E-01
2	rs4832160	85539170	0.91	3.60E-01	0.59	5.57E-01
2	rs58748749	85539589	1.97	4.90E-02	1.39	1.64E-01
2	rs60111186	85540386	1.94	5.20E-02	1.37	1.72E-01
2	rs4832161	85540766	0.88	3.80E-01	0.56	5.76E-01
2	rs4832162	85540780	0.96	3.40E-01	0.64	5.24E-01
2	rs4832163	85541083	0.83	4.10E-01	0.50	6.17E-01
2	rs78272818	85541449	1.83	6.70E-02	1.26	2.09E-01
2	rs118059108	85542506	1.83	6.70E-02	1.26	2.09E-01
2	rs7608892	85542718	-1.44	1.50E-01	-1.08	2.81E-01
2	rs4446071	85543064	-2.11	3.50E-02	-1.82	6.85E-02
2	rs111571392	85543386	1.79	7.40E-02	1.23	2.18E-01
2	rs184734901	85545048	1.83	6.70E-02	1.26	2.09E-01
2	rs7428	85545490	0.63	5.30E-01	0.50	6.18E-01
2	rs117888525	85545637	0.02	9.80E-01	-0.56	5.77E-01
2	rs1053561	85546052	0.71	4.80E-01	0.38	7.05E-01
2	rs1053560	85546192	-1.11	2.70E-01	-0.74	4.62E-01
2	rs142889579	85546520	1.84	6.70E-02	1.26	2.08E-01
2	rs1061782	85546615	-1.53	1.30E-01	-1.16	2.48E-01
2	rs4832164	85546932	-1.44	1.50E-01	-1.08	2.82E-01
2	rs4459734	85547429	-1.44	1.50E-01	-1.08	2.82E-01
2	rs1554110	85547963	-1.44	1.50E-01	-1.08	2.82E-01
2	rs6547611	85548639	-1.53	1.30E-01	-1.20	2.30E-01
2	rs3184781	85549527	0.72	4.70E-01	0.39	6.98E-01
2	rs3184780	85549547	0.72	4.70E-01	0.39	6.98E-01
2	rs4240199	85549868	-1.51	1.30E-01	-1.18	2.38E-01
2	rs3637	85549874	0.70	4.90E-01	0.57	5.71E-01
2	rs6547612	85551184	0.67	5.00E-01	0.33	7.43E-01
2	rs144636827	85551288	1.81	7.00E-02	1.23	2.17E-01
2	rs17026212	85551696	0.48	6.30E-01	0.15	8.84E-01
2	rs2366404	85551779	0.77	4.40E-01	0.45	6.54E-01
2	rs7572750	85552186	-1.44	1.50E-01	-1.08	2.82E-01
2	rs4444526	85552709	0.77	4.40E-01	0.43	6.69E-01
2	rs4435452	85552820	0.88	3.80E-01	0.55	5.84E-01
2	rs1044973	85553784	0.92	3.60E-01	0.56	5.72E-01
2	rs28365983	85555191	1.71	8.80E-02	1.14	2.55E-01
2	rs10460585	85555262	-1.33	1.80E-01	-0.99	3.22E-01
2	rs10460586	85555277	0.69	4.90E-01	0.36	7.19E-01
2	rs17026228	85555357	1.75	8.00E-02	1.18	2.39E-01
2	rs118153646	85556956	1.69	9.20E-02	1.11	2.65E-01
2	rs117331882	85558452	1.68	9.20E-02	1.11	2.67E-01
2	rs7605890	85559525	0.24	8.10E-01	-0.09	9.32E-01
2	rs6733795	85560483	0.63	5.30E-01	0.19	8.48E-01
2	rs144039626	85560538	-0.40	6.90E-01	-0.25	8.06E-01
2	rs76680630	85562100	0.96	3.40E-01	0.47	6.36E-01
2	rs3923229	85562172	0.86	3.90E-01	0.39	6.94E-01

2	rs141728078	85564313	-0.96	3.40E-01	-0.58	5.60E-01
2	rs6747513	85565634	-0.01	9.90E-01	-0.22	8.23E-01
2	rs11689412	85566026	0.11	9.10E-01	-0.12	9.05E-01
2	rs4832165	85566966	0.09	9.30E-01	-0.14	8.90E-01
2	rs11901337	85567661	0.10	9.20E-01	-0.14	8.93E-01
2	rs118107900	85567712	-0.37	7.10E-01	-0.97	3.34E-01
2	rs75265779	85568499	0.97	3.30E-01	0.49	6.25E-01
2	rs138718326	85568546	-0.99	3.20E-01	-0.62	5.37E-01
2	rs15413	85569379	-1.17	2.40E-01	-0.80	4.23E-01
2	rs7600799	85570273	-0.85	4.00E-01	-0.47	6.39E-01
2	rs76973216	85571433	1.05	3.00E-01	0.56	5.73E-01
2	rs3755018	85572227	0.46	6.50E-01	0.16	8.72E-01
2	rs3755017	85572232	0.49	6.20E-01	0.20	8.43E-01
2	rs116909993	85572837	1.05	2.90E-01	0.56	5.72E-01
2	rs908302	85573033	-1.24	2.10E-01	-0.85	3.95E-01
2	rs908303	85573042	1.05	2.90E-01	0.56	5.72E-01
2	rs908305	85573382	1.05	2.90E-01	0.56	5.72E-01
2	rs908306	85573403	0.20	8.50E-01	-0.05	9.64E-01
2	rs938373	85573722	1.05	2.90E-01	0.56	5.72E-01
2	rs938376	85573977	1.05	2.90E-01	0.56	5.72E-01
2	rs4474882	85574015	-1.23	2.20E-01	-0.84	4.03E-01
2	rs73945709	85574198	1.05	2.90E-01	0.56	5.72E-01
2	rs6547615	85574262	0.16	8.70E-01	-0.08	9.37E-01
2	rs58344528	85574538	1.05	2.90E-01	0.56	5.72E-01
2	rs73945710	85574961	1.05	2.90E-01	0.56	5.72E-01
2	rs17026273	85575008	1.05	2.90E-01	0.56	5.72E-01
2	rs57657474	85575312	1.05	2.90E-01	0.56	5.72E-01
2	rs144286394	85575686	1.05	2.90E-01	0.56	5.72E-01
2	rs189334137	85575708	-1.68	9.30E-02	-1.36	1.75E-01
2	rs146588147	85575913	1.28	2.00E-01	0.79	4.28E-01
2	rs150186091	85576150	1.05	2.90E-01	0.57	5.69E-01
2	rs950361	85577369	1.05	3.00E-01	0.56	5.74E-01
2	rs73945715	85577857	1.05	3.00E-01	0.56	5.74E-01
2	rs74648047	85578391	1.05	3.00E-01	0.56	5.74E-01
2	rs76651235	85578593	1.05	3.00E-01	0.56	5.74E-01
2	rs73945718	85578702	0.99	3.20E-01	0.50	6.16E-01
2	rs58549064	85579370	0.99	3.20E-01	0.50	6.16E-01
2	rs62162714	85579389	1.02	3.10E-01	0.53	5.94E-01
2	rs145918353	85579481	0.99	3.20E-01	0.50	6.16E-01
2	rs79184300	85580301	0.99	3.20E-01	0.51	6.10E-01
2	rs28364653	85581759	0.50	6.20E-01	0.05	9.63E-01
2	rs17026280	85581946	0.63	5.30E-01	0.17	8.66E-01
2	rs118123312	85582407	0.64	5.20E-01	0.19	8.52E-01
2	rs78967493	85586108	0.55	5.90E-01	0.07	9.40E-01
2	rs80309578	85586367	0.57	5.70E-01	0.10	9.24E-01
2	rs147860159	85586632	0.57	5.70E-01	0.09	9.24E-01

2	rs145153970	85589032	0.62	5.30E-01	0.15	8.81E-01
2	rs148142265	85589816	0.66	5.10E-01	0.21	8.35E-01
2	rs79473775	85591275	-1.16	2.50E-01	-0.78	4.35E-01
2	rs72840078	85592341	-0.93	3.50E-01	-0.58	5.64E-01
2	rs143258597	85592416	0.78	4.30E-01	0.32	7.50E-01
2	rs57959843	85592759	-1.81	7.00E-02	-1.46	1.46E-01
2	rs61593569	85592841	-1.42	1.60E-01	-1.13	2.58E-01
2	rs76640737	85593994	0.99	3.20E-01	0.52	6.02E-01
2	rs78872575	85594074	0.98	3.30E-01	0.53	5.99E-01
2	rs7340340	85594106	0.16	8.70E-01	-0.15	8.80E-01
2	rs12104980	85596026	1.05	2.90E-01	0.59	5.53E-01
2	rs75561228	85596626	1.12	2.60E-01	0.68	4.94E-01
2	rs3821021	85596739	0.87	3.80E-01	0.57	5.66E-01
2	rs78809694	85596906	1.05	2.90E-01	0.60	5.51E-01
2	rs72840081	85598056	0.90	3.70E-01	0.58	5.61E-01
2	rs117334637	85599013	1.04	3.00E-01	0.59	5.56E-01
2	rs116728233	85599270	0.36	7.20E-01	0.17	8.62E-01
2	rs116467062	85599278	0.36	7.20E-01	0.17	8.62E-01
2	rs72840082	85599291	0.36	7.20E-01	0.17	8.62E-01
2	rs72840084	85600646	0.28	7.80E-01	0.11	9.15E-01
2	rs139452523	85600719	0.28	7.80E-01	0.11	9.15E-01
2	rs72840085	85602138	0.82	4.10E-01	0.52	6.05E-01
2	rs76488771	85602351	0.95	3.40E-01	0.49	6.21E-01
2	rs79634832	85602742	0.97	3.30E-01	0.50	6.16E-01
2	rs6741368	85603064	0.01	1.00E+00	-0.17	8.65E-01
2	rs72840088	85603665	0.83	4.10E-01	0.53	5.99E-01
2	rs116960859	85603845	0.98	3.30E-01	0.51	6.09E-01
2	rs56265944	85604838	-1.37	1.70E-01	-1.01	3.13E-01
2	rs6731223	85605304	-1.25	2.10E-01	-0.88	3.78E-01
2	rs7584492	85606149	-1.42	1.60E-01	-1.06	2.91E-01
2	rs7558094	85606311	0.02	9.90E-01	-0.21	8.34E-01
2	rs144918007	85606507	0.17	8.70E-01	-0.21	8.32E-01
2	rs7585585	85607055	-0.30	7.60E-01	-0.53	5.98E-01
2	rs74832685	85608050	0.11	9.20E-01	-0.26	7.98E-01
2	rs145541570	85608185	0.11	9.20E-01	-0.26	7.98E-01
2	rs113233216	85609284	-0.95	3.40E-01	-0.89	3.72E-01
2	rs116695506	85609287	-1.39	1.60E-01	-1.03	3.03E-01
2	rs72831535	85609931	-0.58	5.60E-01	-0.67	5.02E-01
2	rs11680448	85611043	-0.94	3.50E-01	-0.88	3.79E-01
2	rs149638957	85614538	0.16	8.80E-01	-0.23	8.22E-01
2	rs17026285	85616758	-1.92	5.50E-02	-1.38	1.67E-01
2	rs190782493	85619279	0.15	8.80E-01	-0.23	8.17E-01
2	rs111384899	85620188	-1.90	5.70E-02	-1.39	1.63E-01
2	rs75382021	85621573	0.15	8.80E-01	-0.23	8.18E-01
2	rs11690650	85622317	-0.97	3.30E-01	-0.90	3.69E-01
2	rs139743093	85622926	0.15	8.80E-01	-0.23	8.18E-01

2	rs72831541	85623310	-1.83	6.70E-02	-1.32	1.86E-01
2	rs80248085	85623360	-1.34	1.80E-01	-0.97	3.31E-01
2	rs117284777	85625892	0.21	8.40E-01	-0.19	8.46E-01
2	rs35666320	85626524	-0.36	7.20E-01	-0.58	5.60E-01
2	rs75048601	85626606	-1.83	6.70E-02	-1.45	1.48E-01
2	rs2229668	85628983	-0.34	7.30E-01	-0.57	5.67E-01
2	rs114395253	85630169	-1.88	6.00E-02	-1.50	1.34E-01
2	rs147055812	85630751	-1.92	5.50E-02	-1.54	1.24E-01
2	rs78969947	85631278	0.20	8.40E-01	-0.20	8.43E-01
2	rs138364243	85631743	-1.92	5.50E-02	-1.54	1.24E-01
2	rs56287599	85631819	-1.33	1.80E-01	-1.25	2.10E-01
2	rs72831558	85632928	-1.85	6.40E-02	-1.47	1.42E-01
2	rs72831561	85633053	-1.85	6.40E-02	-1.47	1.42E-01
2	rs59102074	85633410	-1.37	1.70E-01	-1.29	1.96E-01
2	rs72831562	85633500	-1.85	6.40E-02	-1.47	1.42E-01
2	rs77069566	85635427	-1.42	1.60E-01	-1.37	1.72E-01
2	rs56047935	85635629	-1.91	5.60E-02	-1.53	1.25E-01
2	rs11696093	85636195	-2.18	2.90E-02	-1.78	7.54E-02
2	rs3770102	85637837	-2.14	3.30E-02	-2.10	3.58E-02
2	rs79292482	85637979	-0.41	6.80E-01	-0.78	4.34E-01
2	rs11682055	85638323	-2.18	3.00E-02	-2.14	3.27E-02
2	rs74900207	85638839	-0.59	5.60E-01	-0.98	3.29E-01
2	rs62162747	85638865	-2.23	2.60E-02	-1.84	6.51E-02
2	rs113321638	85639358	-0.59	5.50E-01	-0.97	3.32E-01
2	rs62162748	85639417	-2.17	3.00E-02	-1.79	7.39E-02
2	rs142573475	85640667	-0.65	5.20E-01	-1.08	2.80E-01
2	rs62162749	85641824	-2.61	9.10E-03	-2.23	2.55E-02
2	rs62162752	85645545	-2.22	2.60E-02	-2.23	2.55E-02
2	rs62162753	85645593	-2.23	2.60E-02	-2.24	2.52E-02
2	rs77588582	85646308	-0.75	4.50E-01	-1.19	2.34E-01
2	rs62162755	85646417	-2.56	1.00E-02	-2.17	2.98E-02
2	rs62162756	85647672	-2.48	1.30E-02	-2.09	3.63E-02
2	rs11678613	85648552	-2.42	1.60E-02	-2.03	4.21E-02
2	rs79274494	85649575	-0.74	4.60E-01	-1.17	2.42E-01
2	rs142460654	85650283	-2.72	6.50E-03	-2.33	1.96E-02
2	rs11894268	85652690	1.14	2.50E-01	0.93	3.51E-01
2	rs36118887	85653209	1.10	2.70E-01	0.89	3.75E-01
2	rs1877954	85654299	0.99	3.20E-01	0.86	3.93E-01
2	rs58573623	85667717	-0.47	6.40E-01	0.64	5.25E-01
2	rs13401255	85668274	-0.52	6.00E-01	0.07	9.40E-01
2	rs13426753	85668294	-0.53	6.00E-01	0.06	9.53E-01
2	rs13426754	85668296	-0.53	6.00E-01	0.06	9.53E-01
2	rs13426945	85668312	-0.53	6.00E-01	0.06	9.51E-01
2	rs11682343	85668988	-0.68	5.00E-01	-0.08	9.36E-01
2	rs11695023	85669521	-0.60	5.50E-01	0.00	1.00E+00
2	rs118169597	85669526	-0.36	7.20E-01	0.78	4.34E-01

2	rs17026335	85669971	0.20	8.40E-01	0.75	4.51E-01
2	rs79601988	85670100	-0.29	7.70E-01	0.85	3.97E-01
2	rs7580275	85672192	-0.37	7.10E-01	-1.34	1.80E-01
2	rs4832176	85672868	-0.47	6.40E-01	-1.50	1.35E-01
2	rs12614441	85673487	-0.49	6.20E-01	-1.51	1.30E-01
2	rs998496	85674026	-0.47	6.40E-01	-1.50	1.33E-01
2	rs7594872	85674576	-0.46	6.40E-01	-0.58	5.62E-01
2	rs7571491	85675126	-0.53	6.00E-01	-0.64	5.21E-01
2	rs1466036	85675604	-0.53	6.00E-01	-0.64	5.22E-01
2	rs13410895	85676225	-0.61	5.40E-01	-0.72	4.73E-01
2	rs13399100	85676316	-0.59	5.50E-01	-0.70	4.83E-01
2	rs9309621	85677542	-0.61	5.40E-01	-0.72	4.73E-01
2	rs9309622	85677633	-0.60	5.50E-01	-0.71	4.76E-01
2	rs7583141	85678425	-0.52	6.00E-01	-0.64	5.22E-01
2	rs6547619	85679990	-0.40	6.90E-01	-0.53	5.97E-01
2	rs6713075	85680550	-0.41	6.80E-01	-0.52	6.00E-01
2	rs12471415	85682622	-0.44	6.60E-01	-0.55	5.80E-01
2	rs59511452	85683064	-0.38	7.10E-01	-0.52	6.03E-01
2	rs17026352	85683099	-0.50	6.10E-01	-0.64	5.20E-01
2	rs10496317	85683370	-0.42	6.80E-01	-0.56	5.75E-01
2	rs2121395	85683571	-0.47	6.40E-01	-0.61	5.42E-01
2	rs2121396	85683722	-0.49	6.30E-01	-0.63	5.28E-01
2	rs13411569	85683966	-0.49	6.30E-01	-0.63	5.28E-01
2	rs117642985	85684588	0.47	6.40E-01	1.37	1.70E-01
2	rs57365768	85686214	0.55	5.80E-01	1.46	1.44E-01
2	rs56966849	85686597	0.59	5.50E-01	1.50	1.33E-01
2	rs111676519	85686689	0.31	7.60E-01	1.30	1.94E-01
2	rs111920750	85687479	0.57	5.70E-01	1.48	1.39E-01
2	rs111354057	85688571	0.63	5.30E-01	1.54	1.23E-01
2	rs182307472	85689227	-0.10	9.20E-01	0.91	3.62E-01
2	rs113656657	85689487	-0.10	9.20E-01	0.91	3.61E-01
2	rs143388852	85690239	-0.10	9.20E-01	0.90	3.66E-01
2	rs139918327	85691475	0.20	8.40E-01	1.11	2.68E-01
2	rs149792932	85691725	0.45	6.60E-01	1.40	1.62E-01
2	rs188770742	85692078	0.16	8.70E-01	1.09	2.77E-01
2	rs146547398	85692724	0.57	5.70E-01	1.44	1.49E-01
2	rs144406513	85694951	-0.33	7.40E-01	0.56	5.72E-01
2	rs150881308	85695277	0.02	9.80E-01	-0.64	5.24E-01
2	rs76090996	85698857	0.02	9.80E-01	0.87	3.85E-01
2	rs7577642	85699750	0.54	5.90E-01	0.59	5.58E-01
2	rs960066	85700924	-1.29	2.00E-01	-0.92	3.57E-01
2	rs4268923	85701392	-1.51	1.30E-01	-0.32	7.46E-01
2	rs4286278	85701425	0.88	3.80E-01	0.25	8.05E-01
2	rs11883636	85701648	-0.65	5.20E-01	-0.02	9.81E-01
2	rs7585235	85701849	-1.58	1.10E-01	-0.78	4.36E-01
2	rs7585678	85702020	-1.62	1.10E-01	-0.44	6.61E-01

2	rs13017730	85702382	0.89	3.70E-01	0.26	7.96E-01
2	rs1867881	85702983	0.89	3.80E-01	0.26	7.98E-01
2	rs4993940	85703112	-0.89	3.80E-01	-0.27	7.88E-01
2	rs4993939	85703122	-0.85	3.90E-01	-0.23	8.16E-01
2	rs6714421	85703308	-0.80	4.30E-01	-0.18	8.56E-01
2	rs1470501	85703993	-0.88	3.80E-01	-0.26	7.94E-01
2	rs59429327	85706306	-0.11	9.20E-01	0.82	4.12E-01
2	rs72838581	85706407	-1.37	1.70E-01	-1.01	3.12E-01
2	rs117550981	85707367	-0.09	9.30E-01	0.84	4.00E-01
2	rs11680227	85707654	-1.95	5.10E-02	-1.16	2.47E-01
2	rs13422970	85707968	-0.02	9.80E-01	0.72	4.71E-01
2	rs13423156	85708020	-0.02	9.80E-01	0.72	4.71E-01
2	rs78571852	85708706	0.01	9.90E-01	0.83	4.06E-01
2	rs28805618	85709894	0.06	9.50E-01	0.89	3.75E-01
2	rs77142217	85715307	0.19	8.50E-01	1.02	3.08E-01
2	rs9973557	85717355	0.52	6.10E-01	1.34	1.79E-01
2	rs7593195	85721246	-0.22	8.20E-01	0.61	5.42E-01
2	rs72844410	85723006	-0.72	4.70E-01	-0.36	7.21E-01
2	rs72844411	85723859	-0.64	5.20E-01	-0.28	7.79E-01
2	rs72844412	85724009	-0.63	5.30E-01	-0.27	7.87E-01
2	rs72844414	85724103	-0.35	7.30E-01	-0.01	9.92E-01
2	rs73943229	85728580	0.78	4.30E-01	2.12	3.41E-02
2	rs72844420	85729520	-0.68	5.00E-01	-0.32	7.49E-01
2	rs13420118	85731652	1.14	2.60E-01	2.55	1.06E-02
2	rs13420256	85731890	1.02	3.10E-01	2.44	1.46E-02
2	rs71411829	85732718	1.04	3.00E-01	2.43	1.51E-02
2	rs111680529	85736386	-0.92	3.60E-01	-0.52	6.00E-01
2	rs13422558	85737719	0.12	9.00E-01	1.37	1.71E-01
2	rs140242727	85738010	-2.27	2.30E-02	-0.96	3.37E-01
2	rs2044475	85738944	-2.30	2.20E-02	-0.98	3.25E-01
2	rs12994163	85741613	0.27	7.90E-01	1.54	1.23E-01
2	rs7579665	85742616	-2.07	3.80E-02	-0.73	4.64E-01
2	rs12714142	85743260	-0.57	5.70E-01	0.78	4.38E-01
2	rs10174551	85743336	0.08	9.30E-01	1.37	1.72E-01
2	rs10198001	85743357	-0.51	6.10E-01	0.84	4.03E-01
2	rs10172793	85744485	-0.06	9.50E-01	1.28	1.99E-01
2	rs12714143	85744885	0.00	1.00E+00	1.35	1.76E-01
2	rs10190585	85746568	-0.55	5.80E-01	0.86	3.88E-01
2	rs10167018	85746699	-0.49	6.20E-01	0.92	3.57E-01
2	rs10496316	85747371	-0.54	5.90E-01	0.87	3.85E-01
2	rs28404046	85747965	-2.12	3.40E-02	-0.78	4.36E-01
2	rs17735885	85748719	0.15	8.80E-01	1.47	1.42E-01
2	rs28398892	85749918	0.06	9.50E-01	1.42	1.55E-01
2	rs28810460	85750016	0.08	9.40E-01	1.43	1.52E-01
2	rs28479378	85750407	0.06	9.50E-01	1.42	1.54E-01
2	rs6727614	85750853	0.07	9.50E-01	1.43	1.52E-01

2	rs58644336	85752471	-5.30	1.20E-07	-1.44	1.51E-01
2	rs60012532	85752474	-5.87	4.30E-09	-2.14	3.21E-02
2	rs13406611	85752584	0.07	9.40E-01	1.44	1.50E-01
2	rs13431962	85752707	0.04	9.60E-01	1.41	1.57E-01
2	rs6735663	85752906	-5.30	1.20E-07	-1.44	1.50E-01
2	rs11895341	85753553	-5.92	3.30E-09	-2.24	2.54E-02
2	rs11895399	85753806	-5.92	3.30E-09	-2.23	2.59E-02
2	rs11895401	85753847	-5.92	3.30E-09	-2.23	2.59E-02
2	rs34824628	85753888	0.06	9.50E-01	1.43	1.52E-01
2	rs13394343	85754342	-5.59	2.20E-08	0.00	1.00E+00
2	rs6735152	85754450	-5.61	2.00E-08	0.00	1.00E+00
2	rs6721924	85754563	-5.61	2.00E-08	0.00	1.00E+00
2	rs56819945	85755139	-5.64	1.70E-08	-1.68	9.25E-02
2	rs6547620	85755928	-5.69	1.30E-08	0.00	1.00E+00
2	rs58307667	85755967	-5.29	1.20E-07	-1.41	1.59E-01
2	rs6719046	85757371	-5.61	2.00E-08	0.00	1.00E+00
2	rs6733913	85757379	-5.61	2.00E-08	0.00	1.00E+00
2	rs28800839	85757616	-0.04	9.60E-01	1.34	1.79E-01
2	rs6747828	85758015	-5.29	1.20E-07	0.00	1.00E+00
2	rs6750832	85758090	-5.61	2.00E-08	0.00	1.00E+00
2	rs13408713	85758299	-0.04	9.60E-01	1.34	1.79E-01
2	rs6722691	85758352	-5.79	7.00E-09	-1.95	5.10E-02
2	rs2044474	85758790	-5.61	2.10E-08	0.00	1.00E+00
2	rs17026396	85759127	-5.59	2.30E-08	0.00	1.00E+00
2	rs59877521	85759494	-5.59	2.30E-08	0.00	1.00E+00
2	rs6731005	85760395	-5.51	3.50E-08	0.00	1.00E+00
2	rs6705971	85761417	-5.58	2.40E-08	0.00	1.00E+00
2	rs10175792	85761654	-5.58	2.50E-08	0.00	1.00E+00
2	rs10202828	85762675	-0.22	8.30E-01	1.19	2.33E-01
2	rs10179195	85762761	-5.58	2.40E-08	0.00	1.00E+00
2	rs6714157	85763274	-5.59	2.30E-08	0.00	1.00E+00
2	rs6743030	85763520	-5.59	2.20E-08	0.00	1.00E+00
2	rs3755014	85764006	-5.59	2.30E-08	0.00	1.00E+00
2	rs3755015	85764041	-5.59	2.30E-08	0.00	1.00E+00
2	rs1446668	85764960	-5.57	2.60E-08	0.00	1.00E+00
2	rs6729475	85765205	-2.03	4.20E-02	-0.70	4.83E-01
2	rs4258795	85765439	-2.03	4.20E-02	-0.70	4.83E-01
2	rs2289972	85766545	-5.49	4.00E-08	-1.31	1.90E-01
2	rs2028900	85767735	-5.68	1.40E-08	0.00	1.00E+00
2	rs2043675	85769975	-5.49	4.00E-08	-1.31	1.90E-01
2	rs7605975	85772548	-5.63	1.90E-08	0.00	1.00E+00
2	rs62165899	85772628	-0.28	7.80E-01	1.13	2.60E-01
2	rs12473819	85773061	-5.58	2.40E-08	0.00	1.00E+00
2	rs72643495	85773305	-4.91	9.10E-07	-0.77	4.43E-01
2	rs34669893	85774010	-0.28	7.80E-01	1.13	2.60E-01
2	rs6547621	85774676	-5.63	1.90E-08	0.00	1.00E+00

2	rs2028898	85777270	-5.48	4.20E-08	-1.30	1.94E-01
2	rs10179904	85780107	-0.37	7.20E-01	1.03	3.01E-01
2	rs2592551	85780131	-5.48	4.20E-08	-1.30	1.94E-01
2	rs699664	85780536	-5.46	4.80E-08	-1.24	2.14E-01
2	rs762684	85782023	-5.49	4.00E-08	-1.32	1.88E-01
2	rs6738645	85783128	-5.64	1.70E-08	0.00	1.00E+00
2	rs67988001	85787123	-5.52	3.50E-08	-1.37	1.71E-01
2	rs12714145	85787341	-5.46	4.90E-08	0.00	1.00E+00
2	rs10172544	85788270	-5.46	4.90E-08	0.00	1.00E+00
2	rs115669754	85788738	-2.03	4.20E-02	-0.70	4.83E-01
2	rs11890182	85790165	-2.03	4.20E-02	-0.70	4.83E-01
2	rs58768785	85790435	-5.07	4.10E-07	-0.98	3.26E-01
2	rs12615553	85790734	-5.07	4.10E-07	-0.98	3.26E-01
2	rs12714146	85791852	-0.31	7.60E-01	1.09	2.76E-01
2	rs60788310	85792063	-5.08	3.80E-07	-1.01	3.11E-01
2	rs35215812	85792384	-5.64	1.70E-08	0.00	1.00E+00
2	rs139775513	85792487	-0.32	7.50E-01	1.08	2.78E-01
2	rs62165905	85792936	-0.32	7.50E-01	1.08	2.78E-01
2	rs13409377	85793562	-0.32	7.50E-01	1.08	2.78E-01
2	rs10187218	85794114	-0.32	7.50E-01	1.08	2.78E-01
2	rs10187424	85794297	-5.66	1.50E-08	0.00	1.00E+00
2	rs10198569	85794415	-5.66	1.50E-08	0.00	1.00E+00
2	rs17430670	85795456	-0.32	7.50E-01	1.08	2.79E-01
2	rs34212016	85795993	-0.32	7.50E-01	1.08	2.79E-01
2	rs142825030	85796407	-1.76	7.80E-02	-0.47	6.35E-01
2	rs28564788	85796788	-0.32	7.50E-01	1.08	2.79E-01
2	rs147036651	85797227	0.99	3.20E-01	1.84	6.57E-02
2	rs13429968	85797665	-0.32	7.50E-01	1.08	2.79E-01
2	rs2121397	85798858	-0.24	8.10E-01	1.15	2.52E-01
2	rs17508727	85799776	-0.32	7.50E-01	1.08	2.79E-01
2	rs13000511	85800265	-0.32	7.50E-01	1.08	2.79E-01
2	rs62165907	85801137	-0.39	7.00E-01	1.00	3.17E-01
2	rs13432584	85801837	-0.39	7.00E-01	1.00	3.17E-01
2	rs6547622	85801902	-5.52	3.30E-08	-1.38	1.68E-01
2	rs34565621	85802012	-0.39	7.00E-01	1.00	3.17E-01
2	rs34214220	85802294	-0.39	7.00E-01	1.00	3.17E-01
2	rs56359002	85802660	-0.39	7.00E-01	1.00	3.16E-01
2	rs55892665	85802754	-0.39	7.00E-01	1.00	3.17E-01
2	rs13425821	85803242	-0.47	6.40E-01	0.94	3.45E-01
2	rs6757263	85803542	-5.68	1.40E-08	0.00	1.00E+00
2	rs3755008	85803897	-5.56	2.70E-08	-1.48	1.40E-01
2	rs10166612	85804096	-0.43	6.70E-01	0.99	3.22E-01
2	rs3770098	85805367	-5.60	2.20E-08	0.00	1.00E+00
2	rs7579147	85805654	-5.46	4.70E-08	-1.24	2.15E-01
2	rs3731827	85806068	-5.46	4.80E-08	0.00	1.00E+00
2	rs3731828	85806266	-5.40	6.50E-08	-1.09	2.76E-01

2	rs13421434	85807610	-0.32	7.50E-01	1.09	2.74E-01
2	rs1972297	85808573	-5.50	3.70E-08	0.00	1.00E+00
2	rs1009	85808737	-5.50	3.70E-08	0.00	1.00E+00
2	rs1058588	85808871	-5.50	3.70E-08	0.00	1.00E+00
2	rs1010	85808982	-5.31	1.10E-07	0.00	1.00E+00
2	rs7593969	85809670	-5.28	1.30E-07	-0.83	4.05E-01
2	rs7608074	85809703	-5.28	1.30E-07	-0.83	4.05E-01
2	rs1562323	85809711	-5.34	9.10E-08	0.00	1.00E+00
2	rs2366639	85809955	-5.34	9.20E-08	0.00	1.00E+00
2	rs1561198	85809989	-5.32	1.10E-07	0.00	1.00E+00
2	rs116306855	85810670	-4.86	1.20E-06	-0.63	5.31E-01
2	rs11311	85811592	-5.07	3.90E-07	-0.49	6.27E-01
2	rs13387999	85812074	-0.60	5.50E-01	0.88	3.79E-01
2	rs55971080	85812746	-5.03	5.00E-07	0.00	1.00E+00
2	rs12714147	85813834	-0.43	6.70E-01	1.06	2.90E-01
2	rs150679559	85814139	-4.67	3.00E-06	-0.29	7.73E-01
2	rs10206961	85814984	-4.81	1.50E-06	0.00	1.00E+00
2	rs13431159	85815651	0.09	9.30E-01	1.52	1.27E-01
2	rs13394092	85815954	-0.31	7.60E-01	1.19	2.36E-01
2	rs35776719	85817167	0.90	3.70E-01	2.36	1.81E-02
2	rs1374370	85818273	-0.03	9.80E-01	2.11	3.46E-02
2	rs2289976	85818797	0.58	5.60E-01	2.67	7.60E-03
2	rs14976	85818886	0.54	5.90E-01	2.63	8.60E-03
2	rs72843872	85820027	0.37	7.10E-01	2.42	1.53E-02
2	rs14242	85820412	-4.77	1.90E-06	-0.84	4.00E-01
2	rs3755009	85820906	-4.96	7.20E-07	-1.09	2.75E-01
2	rs3755011	85821599	-4.78	1.80E-06	-0.88	3.81E-01
2	rs1562322	85821956	-4.74	2.10E-06	-0.83	4.07E-01
2	rs2232745	85823632	-4.78	1.80E-06	-1.01	3.13E-01
2	rs2232747	85824073	-4.66	3.20E-06	-0.78	4.33E-01
2	rs2232750	85824468	-4.45	8.70E-06	-0.52	6.02E-01
2	rs3198089	85825337	-4.48	7.40E-06	-0.57	5.68E-01
2	rs10302	85825575	-4.39	1.20E-05	-0.44	6.63E-01
2	rs1044158	85826132	-4.71	2.50E-06	0.30	7.63E-01
2	rs11891495	85826721	-0.26	8.00E-01	1.66	9.79E-02
2	rs6733550	85828014	-4.41	1.00E-05	0.78	4.36E-01
2	rs62166770	85834641	-0.21	8.30E-01	1.71	8.75E-02
2	rs13418207	85836891	-4.76	2.00E-06	-1.01	3.12E-01
2	rs76849874	85840358	-4.84	1.30E-06	-1.13	2.57E-01
2	rs10190428	85841558	-4.84	1.30E-06	-1.13	2.57E-01
2	rs6732858	85843881	-0.11	9.10E-01	1.18	2.36E-01
2	rs6743051	85844161	-4.84	1.30E-06	-1.15	2.50E-01
2	rs146368185	85845238	-4.81	1.50E-06	-1.10	2.73E-01
2	rs13383605	85846329	-4.84	1.30E-06	-1.13	2.57E-01
2	rs9678632	85847085	-4.65	3.30E-06	0.25	8.04E-01
2	rs112756646	85847272	-4.91	9.30E-07	-0.92	3.59E-01

2	rs7569073	85852143	-0.39	7.00E-01	0.90	3.66E-01
2	rs2304564	85852667	-4.69	2.70E-06	-0.94	3.49E-01
2	rs12463933	85854015	-0.32	7.50E-01	1.59	1.13E-01
2	rs13406807	85854476	-4.66	3.20E-06	0.20	8.39E-01
2	rs2118176	85855300	-4.69	2.70E-06	-0.94	3.49E-01
2	rs71411832	85857175	-0.39	6.90E-01	0.90	3.69E-01
2	rs10191899	85859602	-4.63	3.60E-06	-0.85	3.93E-01
2	rs10191726	85859603	-4.69	2.70E-06	-0.93	3.50E-01
2	rs115235685	85861325	-0.39	7.00E-01	0.90	3.66E-01
2	rs34623563	85864710	-0.37	7.10E-01	1.55	1.22E-01
2	rs62166779	85865440	-0.37	7.10E-01	1.55	1.22E-01
2	rs17508809	85869770	-0.39	7.00E-01	0.90	3.66E-01
2	rs3821020	85869922	-4.68	2.90E-06	-0.92	3.60E-01
2	rs58588820	85874044	-4.58	4.70E-06	0.35	7.27E-01
2	rs17508843	85874845	-0.34	7.40E-01	1.58	1.14E-01
2	rs7607208	85878158	-0.31	7.50E-01	1.60	1.10E-01
2	rs11887442	85879037	-0.34	7.30E-01	1.58	1.15E-01
2	rs11887443	85879041	-0.34	7.30E-01	1.58	1.15E-01
2	rs1992151	85880892	-4.47	7.80E-06	-0.62	5.38E-01
2	rs56192500	85882919	-0.34	7.40E-01	1.58	1.14E-01
2	rs1130866	85893741	0.41	6.80E-01	0.64	5.25E-01
2	rs78502505	85901579	-0.35	7.30E-01	0.07	9.46E-01
2	rs41369245	85901719	-0.11	9.10E-01	0.31	7.57E-01
2	rs55654080	85906280	0.06	9.50E-01	0.48	6.33E-01
2	rs11695312	85910799	-0.81	4.20E-01	-0.12	9.08E-01
2	rs2070966	85922860	-0.98	3.30E-01	0.45	6.50E-01
2	rs13408224	85923936	-0.95	3.40E-01	0.51	6.13E-01
2	rs34943381	85923997	-0.89	3.70E-01	0.69	4.90E-01
2	rs1561284	85924295	-0.90	3.70E-01	0.68	4.94E-01
2	rs11127	85924729	-0.94	3.50E-01	0.52	6.05E-01
2	rs1437743	85924989	-0.94	3.50E-01	0.52	6.05E-01
2	rs1437744	85925084	-0.95	3.40E-01	0.51	6.09E-01
2	rs1866141	85925386	-0.94	3.50E-01	0.52	6.05E-01
2	rs4240200	85925529	-1.09	2.80E-01	0.48	6.30E-01
2	rs4240201	85925667	-0.95	3.40E-01	0.51	6.14E-01
2	rs4240202	85925680	-0.95	3.40E-01	0.51	6.14E-01
2	rs10192752	85926142	-0.91	3.60E-01	0.67	5.02E-01
2	rs10203826	85926190	-0.91	3.60E-01	0.67	5.02E-01
2	rs10180451	85926238	-0.91	3.60E-01	0.67	5.02E-01
2	rs10180547	85926305	-0.95	3.40E-01	0.63	5.30E-01
2	rs10180391	85926345	-1.09	2.80E-01	0.48	6.30E-01
2	rs2366807	85926598	-0.95	3.40E-01	0.50	6.15E-01
2	rs34702373	85927404	-1.11	2.70E-01	0.46	6.42E-01
2	rs2886768	85927555	-1.31	1.90E-01	0.06	9.51E-01
2	rs62163092	85927661	-1.01	3.10E-01	0.44	6.58E-01
2	rs59373431	85927699	-0.94	3.50E-01	0.51	6.12E-01

2	rs10210321	85928128	-0.98	3.30E-01	0.47	6.36E-01
2	rs1437745	85928283	-0.94	3.50E-01	0.64	5.22E-01
2	rs1837115	85928411	-1.09	2.80E-01	0.48	6.30E-01
2	rs10187314	85928550	-0.97	3.30E-01	0.60	5.46E-01
2	rs10187495	85928556	-1.00	3.20E-01	0.44	6.58E-01
2	rs61191323	85928631	-0.96	3.40E-01	0.49	6.23E-01
2	rs4832186	85929674	-0.98	3.30E-01	0.62	5.34E-01
2	rs4832187	85929675	-0.84	4.00E-01	0.64	5.21E-01
2	rs2118402	85931078	-0.90	3.70E-01	0.55	5.81E-01
2	rs12714150	85932089	-1.07	2.80E-01	0.29	7.70E-01
2	rs12714151	85932210	-1.00	3.20E-01	0.25	8.04E-01
2	rs11126996	85932948	-0.99	3.20E-01	0.25	8.02E-01
2	rs12151742	85933003	0.00	1.00E+00	0.80	4.21E-01
2	rs6547632	85933367	-0.99	3.20E-01	0.26	7.98E-01
2	rs17026595	85934115	0.33	7.40E-01	1.00	3.18E-01
2	rs12151621	85934499	-0.19	8.50E-01	0.61	5.41E-01
2	rs12151752	85934652	-0.17	8.70E-01	0.63	5.30E-01
2	rs7603438	85935182	-0.03	9.80E-01	0.80	4.22E-01
2	rs7577293	85935282	-0.10	9.20E-01	0.72	4.74E-01
2	rs12620113	85936833	-1.10	2.70E-01	0.48	6.31E-01
2	rs7585576	85937785	-1.04	3.00E-01	0.53	5.95E-01
2	rs4525696	85938000	-0.28	7.80E-01	0.29	7.75E-01
2	rs1545141	85938152	0.29	7.70E-01	0.96	3.35E-01
2	rs751163	85938407	-0.07	9.50E-01	0.76	4.45E-01
2	rs1123105	85938588	0.31	7.60E-01	0.95	3.42E-01
2	rs34020122	85947081	0.40	6.90E-01	0.90	3.70E-01
2	rs6759087	85948793	0.53	6.00E-01	0.94	3.46E-01
2	rs11126997	85949396	0.76	4.50E-01	1.11	2.65E-01
2	rs13030230	85953030	-0.22	8.20E-01	0.06	9.51E-01
2	rs2886769	85954533	-0.24	8.10E-01	0.12	9.08E-01
2	rs2366812	85954544	0.09	9.30E-01	0.45	6.55E-01
2	rs34204234	85955027	-0.32	7.50E-01	0.04	9.68E-01
2	rs35369134	85957976	0.09	9.30E-01	0.40	6.88E-01
2	rs11685875	85959436	0.04	9.70E-01	0.35	7.24E-01
2	rs1914820	85960147	0.09	9.30E-01	0.40	6.91E-01
2	rs2164807	85962551	-1.43	1.50E-01	-0.67	5.03E-01
2	rs4832189	85963577	-0.56	5.80E-01	-0.29	7.73E-01
2	rs11686918	85965150	-0.22	8.20E-01	0.04	9.65E-01
2	rs6727520	85965477	-1.05	2.90E-01	-1.04	2.97E-01
2	rs1561283	85965769	-0.12	9.10E-01	0.16	8.75E-01
2	rs6735639	85967773	-1.08	2.80E-01	-1.11	2.66E-01
2	rs6735655	85967815	-1.18	2.40E-01	-1.20	2.29E-01
2	rs1437741	85968579	-0.62	5.40E-01	-0.42	6.75E-01
2	rs6743301	85969520	-0.31	7.60E-01	0.48	6.35E-01
2	rs13430978	85970703	-0.82	4.10E-01	-0.85	3.96E-01
2	rs6750921	85971108	-0.47	6.40E-01	0.31	7.55E-01

2	rs4832191	85971451	0.12	9.10E-01	-0.06	9.50E-01
2	rs4832007	85972406	-0.25	8.00E-01	-0.47	6.37E-01
2	rs4832008	85972565	-0.13	9.00E-01	-0.33	7.41E-01
2	rs56085927	85972684	-0.56	5.80E-01	0.24	8.14E-01
2	rs1356623	85972920	0.14	8.90E-01	-0.09	9.27E-01
2	rs1356621	85973114	-0.14	8.90E-01	-0.28	7.83E-01
2	rs10172667	85973618	-0.49	6.20E-01	-0.38	7.00E-01
2	rs732155	85973993	-0.26	8.00E-01	-0.08	9.34E-01
2	rs1568310	85975013	-0.31	7.60E-01	-0.21	8.32E-01
2	rs17026610	85975594	-1.09	2.80E-01	-0.26	7.93E-01
2	rs1465822	85976395	-0.20	8.40E-01	-0.18	8.58E-01
2	rs55654402	85976601	-0.62	5.40E-01	0.18	8.60E-01
2	rs55678683	85976604	-0.99	3.20E-01	-0.16	8.74E-01
2	rs1465823	85976645	-0.28	7.80E-01	-0.25	8.01E-01
2	rs60250855	85977130	-1.25	2.10E-01	-0.96	3.36E-01
2	rs74674287	86005554	0.35	7.30E-01	0.71	4.75E-01
2	rs10207516	86006187	0.34	7.30E-01	0.71	4.80E-01
2	rs117659482	86007084	-2.75	5.90E-03	-2.61	9.10E-03
2	rs113634581	86011571	-3.03	2.50E-03	-2.87	4.10E-03
2	rs78539868	86011956	-2.71	6.60E-03	-2.55	1.08E-02
2	rs79922307	86012069	-2.66	7.90E-03	-2.50	1.23E-02
2	rs750443	86012676	-0.87	3.80E-01	-0.86	3.87E-01
2	rs750442	86012885	-0.93	3.50E-01	-0.92	3.59E-01
2	rs13386681	86013029	1.16	2.40E-01	1.48	1.39E-01
2	rs736991	86014345	-0.78	4.30E-01	-0.77	4.40E-01
2	rs13402783	86015121	1.35	1.80E-01	1.62	1.05E-01
2	rs13428557	86015289	1.28	2.00E-01	1.56	1.20E-01
2	rs2366818	86015651	0.67	5.00E-01	0.90	3.69E-01
2	rs55882144	86016463	1.47	1.40E-01	1.76	7.82E-02
2	rs1914821	86017065	0.99	3.20E-01	1.19	2.34E-01
2	rs10190632	86017386	0.89	3.70E-01	1.10	2.70E-01
2	rs3731826	86017956	1.37	1.70E-01	1.65	9.85E-02
2	rs727123	86018433	1.37	1.70E-01	1.65	9.85E-02
2	rs17617977	86019382	-0.89	3.70E-01	-0.73	4.63E-01
2	rs13405363	86028150	-0.49	6.20E-01	-0.33	7.45E-01
2	rs13405488	86028257	-0.33	7.40E-01	-0.17	8.65E-01
2	rs76192473	86028397	-0.35	7.20E-01	-0.33	7.42E-01
2	rs72842012	86028904	-0.25	8.00E-01	-0.11	9.10E-01
2	rs76716028	86032392	-1.41	1.60E-01	-1.44	1.50E-01
2	rs58383548	86033012	-0.51	6.10E-01	-0.58	5.59E-01
2	rs11674128	86033046	-0.52	6.00E-01	-0.59	5.55E-01
2	rs17431285	86033287	-1.39	1.60E-01	-1.45	1.47E-01
2	rs10203272	86033879	-0.90	3.70E-01	-0.99	3.20E-01
2	rs72842014	86034007	-0.89	3.70E-01	-0.94	3.49E-01
2	rs56054119	86035133	-0.59	5.60E-01	-0.65	5.16E-01
2	rs78170368	86035154	-1.08	2.80E-01	-1.17	2.43E-01

2	rs6726470	86035852	-1.05	3.00E-01	-1.15	2.48E-01
2	rs76208338	86036705	-0.37	7.10E-01	-0.44	6.62E-01
2	rs59797249	86038664	-0.22	8.30E-01	-0.28	7.82E-01
2	rs13400884	86039821	-1.06	2.90E-01	-1.18	2.37E-01
2	rs79366826	86040003	-0.73	4.70E-01	-0.81	4.19E-01
2	rs13391970	86040220	-0.29	7.70E-01	-0.37	7.14E-01
2	rs13392384	86040596	-0.93	3.50E-01	-1.06	2.88E-01
2	rs57356103	86040834	-0.86	3.90E-01	-0.99	3.20E-01
2	rs58320050	86040843	-0.86	3.90E-01	-0.99	3.20E-01
2	rs11679628	86041354	-0.23	8.20E-01	-0.31	7.56E-01
2	rs11679726	86041539	-0.74	4.60E-01	-0.88	3.79E-01
2	rs11679814	86041681	-0.13	9.00E-01	-0.25	8.06E-01
2	rs11673983	86041824	-0.06	9.60E-01	-0.14	8.91E-01
2	rs13399063	86042133	-0.06	9.60E-01	-0.16	8.75E-01
2	rs11895873	86042515	-0.05	9.60E-01	-0.16	8.75E-01
2	rs11676743	86043203	-0.07	9.50E-01	-0.17	8.66E-01
2	rs72842027	86043672	-0.75	4.50E-01	-0.85	3.97E-01
2	rs7590113	86044275	-0.06	9.50E-01	-0.16	8.70E-01
2	rs72842029	86044389	-0.53	5.90E-01	-0.63	5.27E-01
2	rs72842031	86044397	-0.59	5.60E-01	-0.68	4.98E-01
2	rs11692513	86044595	-0.08	9.30E-01	-0.19	8.50E-01
2	rs57085204	86044902	-0.15	8.80E-01	-0.27	7.90E-01
2	rs10445962	86045396	-0.29	7.70E-01	-0.39	6.94E-01
2	rs57482155	86045749	-0.03	9.70E-01	-0.12	9.05E-01
2	rs2139199	86046509	-0.25	8.10E-01	-0.34	7.36E-01
2	rs1518985	86047094	-1.38	1.70E-01	-1.52	1.30E-01
2	rs11675032	86047497	-0.11	9.10E-01	-0.22	8.24E-01
2	rs6753123	86047505	-0.89	3.70E-01	-0.98	3.28E-01
2	rs6724442	86047529	-0.88	3.80E-01	-0.97	3.33E-01
2	rs72842037	86047891	-0.60	5.50E-01	-0.69	4.93E-01
2	rs11682180	86048139	-0.08	9.40E-01	-0.19	8.51E-01
2	rs6756890	86048211	-1.09	2.80E-01	-1.24	2.14E-01
2	rs11676487	86048593	-1.31	1.90E-01	-1.45	1.47E-01
2	rs9677383	86048688	-1.12	2.60E-01	-1.27	2.04E-01
2	rs9678088	86048933	-0.96	3.40E-01	-1.12	2.62E-01
2	rs7609014	86049656	-0.75	4.60E-01	-0.89	3.72E-01
2	rs7609098	86049729	-1.07	2.80E-01	-1.22	2.22E-01
2	rs7609429	86049774	-1.07	2.90E-01	-1.22	2.23E-01
2	rs11690888	86050857	-0.38	7.00E-01	-0.44	6.61E-01
2	rs1518986	86050967	-1.07	2.90E-01	-1.22	2.22E-01
2	rs1518987	86051235	-1.05	2.90E-01	-1.21	2.25E-01
2	rs72842047	86051648	-0.68	4.90E-01	-0.77	4.41E-01
2	rs77469076	86052723	-0.61	5.50E-01	-0.73	4.65E-01
2	rs72842051	86053331	-0.63	5.30E-01	-0.72	4.73E-01
2	rs56035936	86054300	-0.90	3.70E-01	-0.99	3.22E-01
2	rs56363459	86054301	-0.90	3.70E-01	-0.99	3.22E-01

2	rs56075967	86054799	-0.91	3.60E-01	-1.00	3.16E-01
2	rs55637101	86054810	-0.88	3.80E-01	-0.98	3.29E-01
2	rs11127000	86055154	-1.10	2.70E-01	-1.24	2.14E-01
2	rs56232523	86056459	-0.91	3.70E-01	-0.99	3.22E-01
2	rs73943774	86057820	-0.91	3.60E-01	-0.98	3.27E-01
2	rs72842058	86057945	-0.88	3.80E-01	-0.96	3.39E-01
2	rs1607466	86060958	1.06	2.90E-01	1.29	1.98E-01
2	rs2366946	86061013	1.08	2.80E-01	1.30	1.93E-01
2	rs76799954	86065494	0.30	7.60E-01	0.03	9.74E-01
2	rs1000374	86066173	0.24	8.10E-01	-0.03	9.77E-01
2	rs13014890	86068243	-1.29	2.00E-01	-1.19	2.35E-01
2	rs73943776	86070373	0.30	7.70E-01	0.06	9.56E-01
2	rs73943777	86070374	0.30	7.70E-01	0.06	9.56E-01
2	rs62147514	86070438	1.21	2.30E-01	1.31	1.91E-01
2	rs2280314	86071864	0.30	7.70E-01	0.06	9.56E-01
2	rs2280313	86071885	0.23	8.20E-01	-0.04	9.68E-01
2	rs2280312	86072076	-1.28	2.00E-01	-1.18	2.38E-01
2	rs58315750	86072432	0.23	8.20E-01	-0.04	9.68E-01
2	rs3770097	86073100	-1.28	2.00E-01	-1.18	2.38E-01
2	rs17026666	86074388	0.33	7.40E-01	0.27	7.86E-01
2	rs13020072	86074512	1.19	2.40E-01	1.28	2.00E-01
2	rs3770096	86075429	-0.02	9.90E-01	-0.12	9.04E-01
2	rs11694145	86075582	-1.20	2.30E-01	-1.26	2.08E-01
2	rs34403285	86075967	-1.19	2.40E-01	-1.12	2.61E-01
2	rs13028295	86076024	1.16	2.50E-01	1.25	2.10E-01
2	rs13033743	86076231	-1.19	2.30E-01	-1.25	2.10E-01
2	rs11679283	86076702	1.12	2.60E-01	1.22	2.22E-01
2	rs13034643	86076747	1.12	2.60E-01	1.22	2.22E-01
2	rs17026675	86077198	-1.28	2.00E-01	-1.18	2.39E-01
2	rs59500769	86077538	0.23	8.20E-01	-0.04	9.68E-01
2	rs35493914	86077643	1.19	2.30E-01	1.29	1.98E-01
2	rs10496320	86078478	-1.18	2.40E-01	-1.08	2.81E-01
2	rs17026684	86079129	1.18	2.40E-01	1.28	2.01E-01
2	rs10496319	86079214	1.18	2.40E-01	1.28	2.01E-01
2	rs1518990	86079341	1.08	2.80E-01	1.18	2.39E-01
2	rs78748985	86080990	0.24	8.10E-01	-0.03	9.80E-01
2	rs13014421	86081032	-1.28	2.00E-01	-1.18	2.39E-01
2	rs75654014	86081208	0.24	8.10E-01	-0.03	9.80E-01
2	rs73945524	86082097	0.37	7.10E-01	0.31	7.57E-01
2	rs57150081	86082482	0.37	7.10E-01	0.31	7.57E-01
2	rs6716233	86082593	1.18	2.40E-01	1.28	2.01E-01
2	rs78468492	86083029	0.34	7.30E-01	0.28	7.77E-01
2	rs35477883	86083571	1.12	2.60E-01	1.21	2.27E-01
2	rs147345772	86084188	0.29	7.70E-01	0.23	8.18E-01
2	rs76257081	86084423	1.17	2.40E-01	1.27	2.05E-01
2	rs62147536	86084531	1.16	2.50E-01	1.26	2.08E-01

2	rs6759239	86085558	1.16	2.50E-01	1.26	2.08E-01
2	rs4832210	86085811	-0.90	3.70E-01	-0.86	3.92E-01
2	rs17026701	86086855	0.31	7.60E-01	0.29	7.75E-01
2	rs62147537	86087159	-1.32	1.90E-01	-1.22	2.21E-01
2	rs2366947	86088009	0.74	4.60E-01	0.88	3.80E-01
2	rs2176627	86088966	0.75	4.60E-01	0.89	3.76E-01
2	rs12613609	86089137	0.75	4.50E-01	0.89	3.76E-01
2	rs3770092	86089742	0.19	8.50E-01	-0.08	9.40E-01
2	rs7572209	86090072	-1.40	1.60E-01	-1.32	1.87E-01
2	rs7558479	86090221	-1.40	1.60E-01	-1.32	1.87E-01
2	rs6547635	86091031	0.23	8.20E-01	0.02	9.87E-01
2	rs1533605	86092253	0.75	4.50E-01	0.89	3.72E-01
2	rs62147538	86094686	-1.42	1.60E-01	-1.34	1.81E-01
2	rs4553826	86095201	0.77	4.40E-01	0.91	3.64E-01
2	rs11689889	86095529	0.15	8.80E-01	-0.12	9.05E-01
2	rs12614498	86096042	-1.40	1.60E-01	-1.32	1.87E-01
2	rs56049132	86096311	-1.40	1.60E-01	-1.32	1.87E-01
2	rs4832015	86096810	0.78	4.40E-01	0.92	3.59E-01
2	rs3810821	86096832	-1.41	1.60E-01	-1.34	1.82E-01
2	rs11681915	86097642	0.15	8.80E-01	-0.12	9.05E-01
2	rs11681961	86097701	1.21	2.30E-01	1.30	1.93E-01
2	rs11127003	86098034	-1.41	1.60E-01	-1.33	1.82E-01
2	rs11127004	86098237	-1.41	1.60E-01	-1.33	1.82E-01
2	rs57178127	86098254	0.29	7.70E-01	0.27	7.85E-01
2	rs59757945	86098359	0.02	9.90E-01	-0.23	8.17E-01
2	rs11127006	86099145	-1.40	1.60E-01	-1.32	1.86E-01
2	rs73945530	86099368	0.29	7.70E-01	0.27	7.86E-01
2	rs55656327	86099605	0.23	8.20E-01	0.02	9.85E-01
2	rs6750686	86099691	-0.89	3.70E-01	-0.85	3.94E-01
2	rs11674097	86099921	0.14	8.90E-01	-0.13	8.95E-01
2	rs62147540	86100129	-1.40	1.60E-01	-1.32	1.86E-01
2	rs6547636	86100563	-0.75	4.60E-01	-0.73	4.68E-01
2	rs6547637	86100607	0.75	4.60E-01	0.88	3.78E-01
2	rs2030275	86101899	1.18	2.40E-01	1.28	2.01E-01
2	rs17026729	86102178	-0.21	8.30E-01	-0.17	8.66E-01
2	rs6734488	86102582	0.70	4.90E-01	0.84	4.02E-01
2	rs4832016	86103273	-0.76	4.50E-01	-0.74	4.60E-01
2	rs13422640	86103655	0.67	5.10E-01	0.81	4.20E-01
2	rs150484740	86103690	-0.44	6.60E-01	-0.44	6.59E-01
2	rs13016226	86103740	-1.23	2.20E-01	-1.18	2.37E-01
2	rs6721125	86104729	0.67	5.10E-01	0.82	4.12E-01
2	rs4832213	86105152	0.63	5.30E-01	0.77	4.41E-01
2	rs6753350	86105498	0.59	5.60E-01	0.73	4.66E-01
2	rs115597253	86106058	0.06	9.50E-01	-0.20	8.40E-01
2	rs11687628	86106393	0.48	6.30E-01	0.63	5.31E-01
2	rs12622987	86106547	-1.23	2.20E-01	-1.16	2.48E-01

2	rs78622110	86106628	0.35	7.30E-01	0.08	9.36E-01
2	rs11127008	86107840	-0.83	4.10E-01	-0.80	4.26E-01
2	rs11127009	86107987	-0.80	4.20E-01	-0.77	4.41E-01
2	rs6751743	86108218	-0.79	4.30E-01	-0.77	4.42E-01
2	rs7587003	86108830	-0.82	4.10E-01	-0.80	4.23E-01
2	rs11694341	86110738	0.04	9.70E-01	-0.22	8.28E-01
2	rs6720161	86111250	-0.96	3.40E-01	-0.94	3.46E-01
2	rs1878905	86111688	-0.97	3.30E-01	-0.95	3.43E-01
2	rs12992339	86112625	-1.00	3.20E-01	-0.90	3.70E-01
2	rs7565996	86113282	-0.48	6.30E-01	-0.58	5.65E-01
2	rs1518991	86113514	-0.99	3.20E-01	-0.92	3.57E-01
2	rs1878904	86114252	-1.01	3.10E-01	-0.94	3.48E-01
2	rs59317756	86115214	0.06	9.50E-01	-0.19	8.52E-01
2	rs13024999	86115314	0.91	3.60E-01	0.96	3.37E-01
2	rs138722478	86115870	-0.08	9.40E-01	-0.30	7.61E-01
2	rs28364795	86116168	0.90	3.70E-01	0.95	3.41E-01
2	rs28364794	86116169	-1.08	2.80E-01	-1.01	3.14E-01
2	rs11674537	86117654	0.06	9.50E-01	-0.19	8.52E-01
2	rs12714154	86117958	1.03	3.00E-01	1.09	2.77E-01
2	rs112067849	86118756	0.07	9.40E-01	0.10	9.24E-01
2	rs4313976	86119187	1.04	3.00E-01	1.10	2.71E-01
2	rs11677643	86119403	0.09	9.30E-01	-0.16	8.70E-01
2	rs11683466	86119542	0.09	9.30E-01	-0.16	8.70E-01
2	rs4832217	86119859	-1.10	2.70E-01	-1.02	3.07E-01
2	rs79148864	86120069	0.09	9.30E-01	-0.16	8.70E-01
2	rs6735629	86120668	-1.23	2.20E-01	-1.15	2.51E-01
2	rs1878898	86121484	-1.24	2.10E-01	-1.15	2.50E-01
2	rs6726662	86121938	1.05	2.90E-01	1.10	2.71E-01
2	rs111517594	86122309	0.13	9.00E-01	0.17	8.67E-01
2	rs1401383	86122874	1.07	2.90E-01	1.13	2.60E-01
2	rs1401384	86122942	-1.27	2.00E-01	-1.19	2.36E-01
2	rs76057167	86123277	0.13	9.00E-01	0.16	8.71E-01
2	rs6747179	86123333	-1.27	2.00E-01	-1.19	2.36E-01
2	rs6734115	86123389	-1.27	2.00E-01	-1.19	2.35E-01
2	rs6747294	86123405	-1.27	2.00E-01	-1.19	2.35E-01
2	rs112661520	86123437	0.13	9.00E-01	0.16	8.71E-01
2	rs60313984	86123461	-0.05	9.60E-01	-0.28	7.80E-01
2	rs58686314	86123967	0.13	9.00E-01	0.16	8.71E-01
2	rs4832218	86124229	-1.27	2.00E-01	-1.19	2.36E-01
2	rs2438307	86125299	0.11	9.10E-01	-0.04	9.68E-01
2	rs1543972	86125413	-1.27	2.00E-01	-1.19	2.36E-01
2	rs2679710	86127757	-1.27	2.10E-01	-1.18	2.37E-01
2	rs2679711	86128431	-1.27	2.10E-01	-1.18	2.37E-01
2	rs4832219	86128563	1.08	2.80E-01	1.13	2.59E-01
2	rs62150576	86128647	-1.81	7.00E-02	-1.68	9.22E-02
2	rs2679712	86128761	-1.27	2.10E-01	-1.18	2.37E-01

2	rs2577768	86128841	-1.27	2.10E-01	-1.18	2.37E-01
2	rs2679713	86128855	-1.27	2.10E-01	-1.18	2.37E-01
2	rs4832220	86128929	1.08	2.80E-01	1.13	2.59E-01
2	rs764471	86129498	-1.26	2.10E-01	-1.18	2.38E-01
2	rs2577769	86129877	-1.26	2.10E-01	-1.18	2.38E-01
2	rs2679714	86130073	-1.27	2.00E-01	-1.19	2.32E-01
2	rs2679715	86130087	-1.27	2.00E-01	-1.19	2.32E-01
2	rs2679716	86130206	-1.28	2.00E-01	-1.19	2.34E-01
2	rs2577770	86130270	-1.27	2.00E-01	-1.19	2.33E-01
2	rs151253510	86130368	-0.07	9.40E-01	-0.30	7.66E-01
2	rs12994053	86130435	1.09	2.80E-01	1.17	2.44E-01
2	rs2679717	86130439	-1.31	1.90E-01	-1.25	2.13E-01
2	rs2577771	86130500	-1.26	2.10E-01	-1.18	2.39E-01
2	rs2679718	86130555	-1.23	2.20E-01	-1.15	2.49E-01
2	rs2577772	86130582	-1.23	2.20E-01	-1.15	2.49E-01
2	rs2679719	86130593	-1.23	2.20E-01	-1.15	2.49E-01
2	rs1878899	86130719	-1.27	2.00E-01	-1.20	2.32E-01
2	rs2577774	86132022	-1.34	1.80E-01	-1.27	2.05E-01
2	rs13008291	86132307	-1.83	6.70E-02	-1.70	8.84E-02
2	rs893444	86132521	0.10	9.20E-01	-0.15	8.79E-01
2	rs2577775	86132943	-1.49	1.40E-01	-1.43	1.54E-01
2	rs4832221	86133028	1.05	2.90E-01	1.13	2.59E-01
2	rs2577776	86133031	0.12	9.00E-01	-0.03	9.77E-01
2	rs76371556	86133094	-0.11	9.10E-01	-0.33	7.41E-01
2	rs13422697	86133608	1.07	2.90E-01	1.14	2.53E-01
2	rs55967160	86133729	0.13	9.00E-01	0.17	8.68E-01
2	rs2366950	86134168	1.07	2.90E-01	1.14	2.53E-01
2	rs2577777	86134320	-1.45	1.50E-01	-1.39	1.65E-01
2	rs59968392	86134613	0.13	9.00E-01	0.16	8.69E-01
2	rs2577778	86134679	-1.45	1.50E-01	-1.39	1.65E-01
2	rs2577779	86134910	-1.44	1.50E-01	-1.38	1.68E-01
2	rs2679722	86135154	-1.14	2.50E-01	-1.06	2.89E-01
2	rs4331504	86135201	1.03	3.00E-01	1.11	2.69E-01
2	rs2945824	86135300	-1.13	2.60E-01	-1.05	2.92E-01
2	rs2577780	86135425	-1.43	1.50E-01	-1.37	1.72E-01
2	rs2679723	86135606	0.14	8.90E-01	-0.11	9.12E-01
2	rs2083169	86139049	0.12	9.00E-01	-0.11	9.12E-01
2	rs4292089	86139180	0.17	8.70E-01	0.20	8.39E-01
2	rs2945825	86139804	-1.34	1.80E-01	-1.27	2.03E-01
2	rs2918014	86139868	-1.34	1.80E-01	-1.27	2.03E-01
2	rs2945826	86139914	-1.31	1.90E-01	-1.25	2.12E-01
2	rs55716007	86140088	0.09	9.30E-01	0.12	9.01E-01
2	rs2218037	86140312	0.13	8.90E-01	-0.10	9.20E-01
2	rs79608146	86140574	0.09	9.30E-01	0.12	9.01E-01
2	rs190316395	86140662	0.09	9.30E-01	0.12	9.01E-01
2	rs2577783	86141103	0.13	8.90E-01	-0.10	9.20E-01

2	rs7570836	86141433	0.06	9.50E-01	0.10	9.20E-01
2	rs2679725	86141844	-1.27	2.00E-01	-1.21	2.28E-01
2	rs1518983	86142202	-1.28	2.00E-01	-1.21	2.26E-01
2	rs80304739	86142539	0.08	9.30E-01	0.14	8.92E-01
2	rs2577786	86143143	-1.21	2.30E-01	-1.15	2.50E-01
2	rs2918015	86143565	-1.26	2.10E-01	-1.21	2.26E-01
2	rs2918016	86143754	-1.27	2.00E-01	-1.23	2.20E-01
2	rs140725178	86144435	-1.07	2.90E-01	-1.04	3.00E-01
2	rs12714155	86144635	0.28	7.80E-01	0.04	9.72E-01
2	rs35153287	86144769	-1.09	2.80E-01	-1.05	2.92E-01
2	rs12714156	86144861	0.28	7.80E-01	0.04	9.72E-01
2	rs17026750	86145233	-0.71	4.80E-01	-0.63	5.29E-01
2	rs1438156	86147123	-1.44	1.50E-01	-1.31	1.89E-01
2	rs10192998	86148616	1.29	2.00E-01	1.30	1.94E-01
2	rs10193207	86148796	1.29	2.00E-01	1.30	1.92E-01
2	rs10185207	86149532	1.30	1.90E-01	1.31	1.91E-01
2	rs893449	86149765	1.30	1.90E-01	1.31	1.91E-01
2	rs893448	86149895	1.30	1.90E-01	1.31	1.91E-01
2	rs4613279	86150194	1.30	1.90E-01	1.31	1.91E-01
2	rs12714158	86150631	1.30	1.90E-01	1.31	1.91E-01
2	rs9309627	86152055	1.31	1.90E-01	1.32	1.86E-01
2	rs11693421	86152367	1.35	1.80E-01	1.36	1.73E-01
2	rs11693423	86152370	1.35	1.80E-01	1.36	1.73E-01
2	rs11693435	86152413	1.35	1.80E-01	1.36	1.73E-01
2	rs4832222	86152708	1.35	1.80E-01	1.36	1.73E-01
2	rs1025708	86152899	1.35	1.80E-01	1.36	1.73E-01
2	rs1025707	86152973	1.35	1.80E-01	1.36	1.73E-01
2	rs1025706	86152981	1.35	1.80E-01	1.36	1.73E-01
2	rs2008559	86153447	-1.49	1.40E-01	-1.36	1.74E-01
2	rs1025705	86153537	1.38	1.70E-01	1.40	1.62E-01
2	rs1025704	86153669	1.38	1.70E-01	1.40	1.62E-01
2	rs2164878	86153855	-1.47	1.40E-01	-1.34	1.80E-01
2	rs17026756	86154004	1.38	1.70E-01	1.40	1.62E-01
2	rs934828	86154744	1.38	1.70E-01	1.40	1.62E-01
2	rs1866244	86155244	1.39	1.60E-01	1.41	1.60E-01
2	rs6547647	86155271	-1.47	1.40E-01	-1.34	1.79E-01
2	rs34138456	86155395	-1.80	7.20E-02	-1.71	8.70E-02
2	rs35104020	86156244	-1.80	7.20E-02	-1.71	8.72E-02
2	rs4832224	86156419	0.19	8.50E-01	-0.07	9.42E-01
2	rs4832225	86156434	0.19	8.50E-01	-0.07	9.42E-01
2	rs4832226	86156977	1.39	1.60E-01	1.41	1.60E-01
2	rs6547648	86157215	0.19	8.50E-01	-0.07	9.45E-01
2	rs10181978	86157595	1.39	1.60E-01	1.41	1.60E-01
2	rs12616165	86157728	-1.49	1.40E-01	-1.37	1.72E-01
2	rs13401298	86157997	1.39	1.70E-01	1.40	1.61E-01
2	rs6717839	86158539	0.13	9.00E-01	0.21	8.33E-01

2	rs10185280	86158755	1.41	1.60E-01	1.42	1.55E-01
2	rs6733593	86158992	1.41	1.60E-01	1.42	1.55E-01
2	rs7586560	86159615	1.31	1.90E-01	1.32	1.87E-01
2	rs4832017	86160417	1.27	2.00E-01	1.28	1.99E-01
2	rs61477982	86160532	0.26	8.00E-01	0.34	7.32E-01
2	rs7576135	86160562	-1.32	1.90E-01	-1.18	2.36E-01
2	rs147927322	86160696	-1.23	2.20E-01	-0.97	3.32E-01
2	rs80305577	86160876	-0.14	8.90E-01	-0.39	6.96E-01
2	rs10166563	86163082	0.15	8.80E-01	-0.11	9.15E-01
2	rs10202148	86163767	-0.24	8.10E-01	-0.22	8.30E-01
2	rs4832228	86165385	0.23	8.20E-01	0.15	8.79E-01
2	rs11691934	86165443	0.29	7.70E-01	0.22	8.30E-01
2	rs10167259	86165765	-0.25	8.00E-01	-0.21	8.31E-01
2	rs10460588	86167494	-0.48	6.30E-01	-0.50	6.19E-01
2	rs2366955	86167546	0.77	4.40E-01	0.68	4.97E-01
2	rs13431245	86167602	0.91	3.60E-01	0.73	4.66E-01
2	rs2033771	86167716	0.78	4.40E-01	0.68	4.95E-01
2	rs2033770	86167829	0.71	4.80E-01	0.61	5.39E-01
2	rs4832229	86168686	0.32	7.50E-01	0.15	8.77E-01
2	rs4832230	86168705	-0.29	7.70E-01	-0.39	6.95E-01
2	rs113059202	86168762	-0.88	3.80E-01	-0.82	4.15E-01
2	rs2218036	86169110	-0.29	7.70E-01	-0.39	6.94E-01
2	rs2218035	86169544	-0.17	8.70E-01	-0.05	9.63E-01
2	rs2366956	86169781	-0.19	8.50E-01	-0.07	9.43E-01
2	rs2366957	86169952	-0.29	7.70E-01	-0.39	6.94E-01
2	rs2366958	86169989	-0.12	9.10E-01	0.00	9.98E-01
2	rs2366959	86170311	-0.51	6.10E-01	-0.55	5.80E-01
2	rs2196701	86170704	-0.33	7.40E-01	-0.44	6.61E-01
2	rs57339071	86171163	0.96	3.40E-01	0.74	4.57E-01
2	rs6749218	86171822	0.96	3.40E-01	0.74	4.57E-01
2	rs6752561	86172231	0.96	3.40E-01	0.75	4.54E-01
2	rs1370699	86172274	-0.48	6.30E-01	-0.59	5.56E-01
2	rs9309628	86172328	-0.21	8.40E-01	-0.08	9.38E-01
2	rs2366960	86174213	0.45	6.50E-01	0.26	7.95E-01
2	rs6759489	86176032	-0.99	3.20E-01	-0.93	3.53E-01
2	rs6718632	86176084	-1.05	2.90E-01	-0.99	3.21E-01
2	rs11127010	86176194	-0.31	7.60E-01	-0.37	7.09E-01
2	rs10185771	86176218	0.66	5.10E-01	0.73	4.66E-01
2	rs6747424	86176224	-1.04	3.00E-01	-0.98	3.25E-01
2	rs6705122	86176258	-1.05	3.00E-01	-0.99	3.24E-01
2	rs60981340	86177046	-1.12	2.60E-01	-1.06	2.90E-01
2	rs735737	86177101	-1.13	2.60E-01	-1.07	2.84E-01
2	rs735738	86177322	0.66	5.10E-01	0.73	4.68E-01
2	rs893443	86178058	0.50	6.20E-01	0.31	7.58E-01
2	rs11891333	86178394	-1.12	2.60E-01	-1.07	2.84E-01
2	rs7603766	86178466	0.74	4.60E-01	0.63	5.27E-01

2	rs6756342	86178946	-0.38	7.10E-01	-0.48	6.34E-01
2	rs76945217	86180226	0.62	5.30E-01	0.46	6.46E-01
2	rs6706720	86180754	0.80	4.30E-01	0.68	4.96E-01
2	rs12472588	86181221	0.66	5.10E-01	0.57	5.71E-01
2	rs12476390	86181427	0.66	5.10E-01	0.57	5.71E-01
2	rs11890514	86181874	0.61	5.40E-01	0.51	6.07E-01
2	rs4514874	86182648	0.69	4.90E-01	0.59	5.52E-01
2	rs2012798	86182756	-1.03	3.00E-01	-1.05	2.95E-01
2	rs1866243	86182842	0.61	5.40E-01	0.49	6.25E-01
2	rs117788935	86182994	-1.33	1.80E-01	-1.38	1.67E-01
2	rs12470643	86183224	0.66	5.10E-01	0.56	5.76E-01
2	rs60497765	86183319	-0.08	9.40E-01	-0.14	8.86E-01
2	rs10186005	86183427	0.66	5.10E-01	0.55	5.80E-01
2	rs58926604	86183592	-0.07	9.40E-01	-0.14	8.87E-01
2	rs1438155	86183897	0.48	6.30E-01	0.38	7.05E-01
2	rs1438154	86184026	0.49	6.30E-01	0.39	6.99E-01
2	rs1438153	86184027	0.49	6.30E-01	0.39	6.99E-01
2	rs6708525	86184199	0.46	6.40E-01	0.37	7.12E-01
2	rs76380182	86184239	-0.16	8.80E-01	-0.22	8.28E-01
2	rs6737454	86184441	0.51	6.10E-01	0.39	6.94E-01
2	rs6547651	86184486	0.49	6.30E-01	0.37	7.11E-01
2	rs13397189	86184866	-0.38	7.00E-01	-0.49	6.24E-01
2	rs12714161	86185013	-0.33	7.50E-01	-0.43	6.69E-01
2	rs10181091	86185165	-0.35	7.30E-01	-0.45	6.50E-01
2	rs6713002	86185291	-0.34	7.40E-01	-0.45	6.54E-01
2	rs6728146	86185319	-0.34	7.40E-01	-0.45	6.54E-01
2	rs61371265	86185775	-0.15	8.80E-01	-0.21	8.37E-01
2	rs58468324	86186122	-0.15	8.80E-01	-0.21	8.37E-01
2	rs73943920	86186195	-1.04	3.00E-01	-1.06	2.88E-01
2	rs77691906	86187221	-0.12	9.00E-01	-0.17	8.64E-01
2	rs1866242	86187595	0.83	4.10E-01	0.69	4.87E-01
2	rs189303047	86188387	-0.22	8.30E-01	-0.27	7.90E-01
2	rs74509951	86188519	-0.12	9.10E-01	-0.17	8.66E-01
2	rs117376463	86188737	-0.12	9.10E-01	-0.17	8.66E-01
2	rs80044847	86189091	-1.93	5.40E-02	-1.85	6.36E-02
2	rs79159000	86189540	-0.17	8.70E-01	-0.22	8.30E-01
2	rs75636569	86189717	-0.12	9.10E-01	-0.17	8.66E-01
2	rs13000793	86189720	0.90	3.70E-01	0.77	4.41E-01
2	rs76722999	86189963	-0.06	9.60E-01	-0.10	9.21E-01
2	rs60471596	86190238	-0.12	9.10E-01	-0.17	8.67E-01
2	rs117998426	86191061	-0.19	8.50E-01	-0.24	8.08E-01
2	rs188964249	86191367	-0.21	8.30E-01	-0.26	7.92E-01
2	rs148959698	86192240	-0.23	8.20E-01	-0.28	7.76E-01
2	rs190594477	86192931	-1.25	2.10E-01	-1.29	1.97E-01
2	rs138651655	86193175	-0.28	7.80E-01	-0.33	7.39E-01
2	rs144869902	86193263	0.32	7.50E-01	0.04	9.65E-01

2	rs140338843	86194655	-1.23	2.20E-01	-1.27	2.03E-01
2	rs192092024	86196813	-0.27	7.90E-01	-0.32	7.50E-01
2	rs74742101	86197295	-0.28	7.80E-01	-0.33	7.39E-01
2	rs117382740	86197978	-0.28	7.80E-01	-0.33	7.39E-01
2	rs74586076	86199231	-0.33	7.40E-01	-0.38	7.03E-01
2	rs76368495	86199351	-0.34	7.30E-01	-0.43	6.70E-01
2	rs75008958	86199431	-0.28	7.80E-01	-0.33	7.39E-01
2	rs80211933	86199735	-0.28	7.80E-01	-0.33	7.39E-01
2	rs117963811	86199794	-0.28	7.80E-01	-0.33	7.39E-01
2	rs6760112	86202212	-0.32	7.50E-01	-0.37	7.12E-01
2	rs77279568	86202724	-0.28	7.80E-01	-0.33	7.39E-01
2	rs56977454	86204014	-1.11	2.70E-01	-1.18	2.40E-01
2	rs57963460	86205089	0.06	9.50E-01	0.01	9.92E-01
2	rs144999421	86205288	0.05	9.60E-01	0.01	9.94E-01
2	rs57318396	86206297	0.06	9.50E-01	0.01	9.90E-01
2	rs1561330	86206482	1.17	2.40E-01	1.05	2.94E-01
2	rs61199255	86206707	0.01	9.90E-01	-0.04	9.71E-01
2	rs77691633	86208057	0.04	9.70E-01	-0.01	9.91E-01
2	rs143707237	86208566	0.04	9.70E-01	-0.02	9.85E-01
2	rs79968864	86208649	0.04	9.70E-01	-0.02	9.85E-01
2	rs934830	86208780	1.02	3.10E-01	0.89	3.74E-01
2	rs77692351	86208952	-0.02	9.80E-01	-0.07	9.44E-01
2	rs3937593	86210273	-0.42	6.70E-01	-0.57	5.71E-01
2	rs10181916	86210305	0.79	4.30E-01	0.67	5.06E-01
2	rs56861544	86210364	-0.02	9.80E-01	-0.07	9.46E-01
2	rs77213255	86211593	-0.02	9.80E-01	-0.07	9.48E-01
2	rs140564603	86211758	-0.02	9.80E-01	-0.07	9.48E-01
2	rs112282891	86211775	-1.08	2.80E-01	-1.15	2.50E-01
2	rs56326127	86212201	-1.05	3.00E-01	-1.12	2.63E-01
2	rs10189191	86212808	0.77	4.40E-01	0.64	5.23E-01
2	rs58365138	86213105	-1.05	2.90E-01	-1.12	2.62E-01
2	rs1866245	86214360	0.61	5.40E-01	0.48	6.31E-01
2	rs4480982	86215059	0.62	5.40E-01	0.49	6.22E-01
2	rs4346389	86215100	0.65	5.20E-01	0.52	6.03E-01
2	rs75787769	86215273	0.13	9.00E-01	0.11	9.16E-01
2	rs75015204	86215411	0.13	9.00E-01	0.11	9.16E-01
2	rs60336356	86216273	0.17	8.60E-01	0.15	8.82E-01
2	rs1020095	86216275	0.68	5.00E-01	0.55	5.80E-01
2	rs12714162	86216343	0.40	6.90E-01	0.39	6.93E-01
2	rs12714163	86216518	0.72	4.70E-01	0.59	5.55E-01
2	rs58898686	86216909	-1.11	2.70E-01	-1.18	2.39E-01
2	rs4832232	86217793	0.66	5.10E-01	0.55	5.86E-01
2	rs12714164	86217927	0.56	5.70E-01	0.44	6.58E-01
2	rs12714165	86218161	0.56	5.70E-01	0.44	6.57E-01
2	rs58186343	86218400	-1.10	2.70E-01	-1.17	2.42E-01
2	rs4832019	86219227	0.23	8.20E-01	0.23	8.18E-01

2	rs4832020	86219285	0.54	5.90E-01	0.42	6.76E-01
2	rs10198260	86220680	0.54	5.90E-01	0.42	6.72E-01
2	rs75722763	86224925	0.23	8.10E-01	0.27	7.85E-01
2	rs61094144	86224996	0.28	7.80E-01	0.32	7.47E-01
2	rs11903682	86225428	0.49	6.20E-01	0.40	6.91E-01
2	rs13004643	86225535	0.14	8.90E-01	0.17	8.63E-01
2	rs7599498	86226374	0.96	3.40E-01	0.72	4.71E-01
2	rs72847838	86226397	0.83	4.10E-01	0.75	4.55E-01
2	rs1978842	86226539	0.80	4.20E-01	0.72	4.74E-01
2	rs73943946	86226623	-1.05	3.00E-01	-1.15	2.49E-01
2	rs76297018	86227007	-1.05	3.00E-01	-1.15	2.49E-01
2	rs6740044	86227341	0.09	9.30E-01	0.13	9.00E-01
2	rs74743393	86227421	-1.05	2.90E-01	-1.16	2.44E-01
2	rs9679308	86227455	0.95	3.40E-01	0.71	4.75E-01
2	rs10168997	86227724	0.82	4.10E-01	0.74	4.62E-01
2	rs78296101	86228945	0.40	6.90E-01	0.44	6.62E-01
2	rs78708991	86229116	0.35	7.20E-01	0.39	6.99E-01
2	rs72847843	86229378	0.94	3.50E-01	0.70	4.84E-01
2	rs13384563	86230921	-1.06	2.90E-01	-1.17	2.42E-01
2	rs183133363	86230956	-0.77	4.40E-01	-0.54	5.87E-01
2	rs17579744	86231059	0.83	4.10E-01	0.75	4.56E-01
2	rs112648158	86232592	-1.05	2.90E-01	-1.17	2.44E-01
2	rs60201381	86233115	1.00	3.20E-01	0.77	4.41E-01
2	rs7569121	86234545	0.73	4.70E-01	0.60	5.47E-01
2	rs144209318	86234640	-0.52	6.00E-01	-0.31	7.58E-01
2	rs140192842	86235790	0.59	5.60E-01	0.61	5.40E-01
2	rs1036554	86236099	-1.06	2.90E-01	-1.18	2.39E-01
2	rs11885520	86236720	1.15	2.50E-01	0.91	3.61E-01
2	rs149643224	86236875	-1.11	2.70E-01	-1.20	2.31E-01
2	rs13390702	86237935	-0.99	3.20E-01	-1.11	2.66E-01
2	rs137984711	86238826	-0.98	3.30E-01	-0.87	3.87E-01
2	rs75675321	86238994	-1.00	3.20E-01	-1.11	2.66E-01
2	rs10178540	86239346	0.21	8.30E-01	0.30	7.61E-01
2	rs1469981	86239772	1.02	3.10E-01	0.92	3.57E-01
2	rs72847853	86240361	1.07	2.80E-01	0.84	4.00E-01
2	rs80225690	86240796	0.45	6.50E-01	0.47	6.40E-01
2	rs57361751	86241006	0.50	6.20E-01	0.52	6.06E-01
2	rs879649	86241531	-0.33	7.40E-01	-0.43	6.67E-01
2	rs10185452	86241607	-0.12	9.00E-01	-0.08	9.34E-01
2	rs78929944	86241990	0.40	6.90E-01	0.42	6.77E-01
2	rs57405155	86242680	0.39	7.00E-01	0.40	6.89E-01
2	rs6708805	86244465	0.87	3.80E-01	0.78	4.37E-01
2	rs10178120	86244704	-0.15	8.80E-01	-0.11	9.16E-01
2	rs11895453	86244982	1.10	2.70E-01	0.87	3.84E-01
2	rs6741224	86245417	0.84	4.00E-01	0.75	4.55E-01
2	rs77225252	86247745	0.63	5.30E-01	0.72	4.71E-01

2	rs75834045	86247862	0.38	7.00E-01	0.40	6.90E-01
2	rs12622009	86248325	-1.04	3.00E-01	-1.20	2.31E-01
2	rs3731822	86248494	0.38	7.00E-01	0.40	6.90E-01
2	rs191060460	86249837	0.35	7.30E-01	0.36	7.17E-01
2	rs76873798	86250600	0.40	6.90E-01	0.41	6.81E-01
2	rs6707736	86251148	1.08	2.80E-01	0.85	3.95E-01
2	rs116583796	86251200	0.40	6.90E-01	0.41	6.81E-01
2	rs79781390	86251511	0.40	6.90E-01	0.41	6.81E-01
2	rs58077381	86251951	1.13	2.60E-01	0.92	3.60E-01
2	rs144557854	86252094	0.40	6.90E-01	0.41	6.81E-01
2	rs77962929	86253572	0.33	7.40E-01	0.29	7.71E-01
2	rs10173155	86253581	-0.27	7.90E-01	-0.39	6.97E-01
2	rs12997506	86254268	0.07	9.40E-01	0.17	8.64E-01
2	rs11127012	86256097	-0.22	8.30E-01	-0.42	6.78E-01
2	rs11899576	86256193	-1.04	3.00E-01	-1.19	2.33E-01
2	rs13420958	86256868	1.09	2.70E-01	0.88	3.78E-01
2	rs11890563	86257727	0.95	3.40E-01	0.75	4.51E-01
2	rs2278536	86258784	-0.08	9.40E-01	0.02	9.81E-01
2	rs75657314	86259141	0.47	6.40E-01	0.48	6.33E-01
2	rs2276626	86259443	-0.65	5.20E-01	-0.79	4.30E-01
2	rs4832234	86259741	-0.08	9.30E-01	0.02	9.86E-01
2	rs118166086	86260170	0.47	6.40E-01	0.48	6.33E-01
2	rs117549724	86260535	0.46	6.40E-01	0.48	6.34E-01
2	rs78239085	86260785	0.44	6.60E-01	0.45	6.49E-01
2	rs61056382	86261097	0.42	6.80E-01	0.43	6.69E-01
2	rs3755005	86261514	0.46	6.40E-01	0.48	6.34E-01
2	rs2164879	86261579	-1.06	2.90E-01	-1.21	2.27E-01
2	rs148585955	86262006	0.46	6.40E-01	0.48	6.34E-01
2	rs3770091	86263166	0.46	6.40E-01	0.48	6.34E-01
2	rs3770090	86263288	-1.07	2.90E-01	-1.22	2.24E-01
2	rs3770088	86263941	0.28	7.80E-01	0.24	8.10E-01
2	rs118125930	86264472	2.58	9.90E-03	2.42	1.54E-02
2	rs717144	86264630	-0.36	7.20E-01	-0.48	6.30E-01
2	rs114068877	86265740	0.64	5.20E-01	0.40	6.92E-01
2	rs3770087	86267068	-1.08	2.80E-01	-1.23	2.19E-01
2	rs3770086	86267278	0.48	6.30E-01	0.49	6.23E-01
2	rs9677675	86268494	1.08	2.80E-01	0.87	3.86E-01
2	rs34892520	86269163	1.08	2.80E-01	0.87	3.86E-01
2	rs2278534	86269883	0.40	6.90E-01	0.42	6.74E-01
2	rs2278533	86269900	0.45	6.50E-01	0.47	6.39E-01
2	rs13024123	86270966	-0.07	9.50E-01	0.04	9.70E-01
2	rs34478066	86271073	-0.07	9.50E-01	0.04	9.70E-01
2	rs7572386	86272191	-0.03	9.80E-01	0.07	9.42E-01
2	rs61254540	86272309	0.45	6.50E-01	0.47	6.41E-01
2	rs73947314	86273116	-1.04	3.00E-01	-1.19	2.33E-01
2	rs57150140	86273167	0.46	6.40E-01	0.48	6.30E-01

2	rs60329449	86273247	0.42	6.80E-01	0.43	6.65E-01
2	rs2886885	86273945	-0.42	6.80E-01	-0.54	5.92E-01
2	rs6755228	86273997	0.46	6.40E-01	0.48	6.30E-01
2	rs75362844	86274124	0.46	6.40E-01	0.48	6.30E-01
2	rs4832236	86274890	-0.05	9.60E-01	0.05	9.57E-01
2	rs11891096	86275698	1.08	2.80E-01	0.87	3.86E-01
2	rs2289239	86275985	-0.04	9.70E-01	0.06	9.49E-01
2	rs10208184	86276894	-0.45	6.50E-01	-0.56	5.72E-01
2	rs9789563	86277190	-1.11	2.70E-01	-1.28	2.00E-01
2	rs3770085	86278947	0.41	6.80E-01	0.42	6.72E-01
2	rs3770084	86279005	0.38	7.00E-01	0.40	6.91E-01
2	rs17026854	86280496	-1.06	2.90E-01	-1.21	2.26E-01
2	rs3770083	86280786	0.46	6.50E-01	0.47	6.37E-01
2	rs3770082	86280922	-1.06	2.90E-01	-1.21	2.26E-01
2	rs3770081	86280925	-1.21	2.20E-01	-1.36	1.75E-01
2	rs2278085	86281844	-1.06	2.90E-01	-1.21	2.26E-01
2	rs2278086	86281905	-0.74	4.60E-01	-0.87	3.87E-01
2	rs2278087	86281946	-1.06	2.90E-01	-1.21	2.26E-01
2	rs2278088	86281967	-1.06	2.90E-01	-1.21	2.26E-01
2	rs58026145	86282454	0.46	6.50E-01	0.47	6.37E-01
2	rs74732648	86282735	0.46	6.50E-01	0.47	6.37E-01
2	rs311581	86282960	1.01	3.10E-01	0.80	4.23E-01
2	rs60649958	86283000	-1.06	2.90E-01	-1.21	2.26E-01
2	rs78646493	86283195	-0.74	4.60E-01	-0.87	3.87E-01
2	rs111295372	86283357	-0.73	4.70E-01	-0.85	3.98E-01
2	rs57083584	86283648	-1.09	2.80E-01	-1.24	2.15E-01
2	rs60479377	86283948	-0.74	4.60E-01	-0.87	3.86E-01
2	rs311582	86284286	0.99	3.20E-01	0.79	4.32E-01
2	rs58861315	86284302	0.46	6.50E-01	0.47	6.37E-01
2	rs311583	86284340	1.00	3.20E-01	0.79	4.31E-01
2	rs60719866	86284378	-0.76	4.50E-01	-0.89	3.73E-01
2	rs76081054	86285037	-0.76	4.50E-01	-0.89	3.73E-01
2	rs311584	86285146	1.00	3.20E-01	0.79	4.30E-01
2	rs113149714	86285379	-1.11	2.70E-01	-1.25	2.10E-01
2	rs311585	86286010	-0.28	7.80E-01	-0.38	7.07E-01
2	rs77550688	86287477	0.46	6.50E-01	0.47	6.37E-01
2	rs74383353	86288007	0.41	6.80E-01	0.42	6.72E-01
2	rs311586	86288519	1.04	3.00E-01	0.82	4.13E-01
2	rs311587	86288981	-0.44	6.60E-01	-0.63	5.29E-01
2	rs17618001	86289303	-1.10	2.70E-01	-1.25	2.11E-01
2	rs59947468	86289436	-0.76	4.50E-01	-0.89	3.74E-01
2	rs75109362	86289889	-1.10	2.70E-01	-1.25	2.11E-01
2	rs311588	86290186	-0.30	7.60E-01	-0.40	6.91E-01
2	rs75713195	86290659	-1.10	2.70E-01	-1.25	2.11E-01
2	rs311561	86291298	1.04	3.00E-01	0.81	4.16E-01
2	rs57398459	86291326	-1.10	2.70E-01	-1.25	2.11E-01

2	rs311562	86291900	-0.75	4.50E-01	-0.97	3.34E-01
2	rs311563	86292656	-0.45	6.50E-01	-0.64	5.21E-01
2	rs77309268	86293294	-1.10	2.70E-01	-1.25	2.10E-01
2	rs311564	86293498	-0.49	6.20E-01	-0.59	5.55E-01
2	rs3821019	86293624	0.41	6.80E-01	0.43	6.70E-01
2	rs311565	86293829	-0.44	6.60E-01	-0.63	5.31E-01
2	rs311567	86294662	-0.74	4.60E-01	-0.95	3.40E-01
2	rs148274377	86294823	0.03	9.80E-01	0.21	8.35E-01
2	rs311568	86295271	1.05	2.90E-01	0.82	4.10E-01
2	rs311569	86295296	-0.42	6.80E-01	-0.61	5.42E-01
2	rs311570	86296086	1.05	2.90E-01	0.82	4.10E-01
2	rs6735014	86296413	0.25	8.00E-01	0.38	7.08E-01
2	rs77298352	86296422	0.46	6.50E-01	0.47	6.36E-01
2	rs311572	86296566	-0.42	6.70E-01	-0.61	5.42E-01
2	rs311576	86298098	-0.29	7.70E-01	-0.39	6.98E-01
2	rs12714166	86298377	0.29	7.70E-01	0.42	6.74E-01
2	rs311578	86298588	-0.29	7.70E-01	-0.39	6.94E-01
2	rs1019591	86298599	-1.07	2.90E-01	-1.22	2.23E-01
2	rs79150666	86298864	0.28	7.80E-01	0.24	8.11E-01
2	rs311579	86299122	-0.35	7.30E-01	-0.44	6.57E-01
2	rs3770077	86299895	-0.48	6.30E-01	-0.40	6.87E-01
2	rs6750765	86300308	-1.02	3.10E-01	-1.17	2.44E-01
2	rs4832021	86301218	-0.39	7.00E-01	-0.57	5.66E-01
2	rs12994838	86301305	0.08	9.40E-01	0.21	8.34E-01
2	rs3770076	86301899	0.41	6.80E-01	0.42	6.74E-01
2	rs3770075	86301978	0.09	9.30E-01	0.21	8.30E-01
2	rs3815840	86302103	-0.73	4.70E-01	-0.94	3.49E-01
2	rs2288116	86302409	0.46	6.50E-01	0.47	6.39E-01
2	rs3770073	86302939	1.10	2.70E-01	0.87	3.82E-01
2	rs12614260	86303079	-1.09	2.80E-01	-1.24	2.16E-01
2	rs7340327	86303749	1.10	2.70E-01	0.87	3.82E-01
2	rs2288117	86305123	1.10	2.70E-01	0.87	3.82E-01
2	rs74607112	86305534	-0.89	3.70E-01	-0.98	3.27E-01
2	rs929916	86305814	1.10	2.70E-01	0.87	3.82E-01
2	rs4832239	86306111	1.12	2.60E-01	0.90	3.70E-01
2	rs7562837	86306251	-0.65	5.10E-01	-0.79	4.30E-01
2	rs12328100	86307128	0.01	9.90E-01	0.06	9.50E-01
2	rs17584578	86307624	-1.09	2.70E-01	-1.24	2.16E-01
2	rs76158656	86307809	0.43	6.70E-01	0.44	6.58E-01
2	rs4832240	86308459	-0.36	7.20E-01	-0.43	6.64E-01
2	rs78032828	86309032	-1.13	2.60E-01	-1.27	2.03E-01
2	rs4832241	86309299	-0.42	6.70E-01	-0.61	5.41E-01
2	rs2288118	86310110	-0.42	6.70E-01	-0.61	5.41E-01
2	rs79060839	86310782	0.91	3.60E-01	0.98	3.29E-01
2	rs58810051	86311099	0.26	8.00E-01	0.21	8.33E-01
2	rs13028023	86311485	0.29	7.70E-01	0.44	6.58E-01

2	rs12714167	86312176	0.25	8.00E-01	0.41	6.80E-01
2	rs111264437	86312185	-1.13	2.60E-01	-1.27	2.03E-01
2	rs113143529	86312222	-1.13	2.60E-01	-1.27	2.03E-01
2	rs10187547	86312274	-0.42	6.70E-01	-0.61	5.41E-01
2	rs7559420	86314753	-0.42	6.70E-01	-0.61	5.41E-01
2	rs2288119	86315526	-0.42	6.70E-01	-0.61	5.42E-01
2	rs2288120	86315741	-0.42	6.70E-01	-0.61	5.41E-01
2	rs1991106	86316174	-0.42	6.70E-01	-0.61	5.42E-01
2	rs1424522	86316382	0.26	8.00E-01	0.42	6.74E-01
2	rs2288121	86316834	-0.69	4.90E-01	-0.90	3.67E-01
2	rs10187150	86317735	0.27	7.90E-01	0.43	6.68E-01
2	rs6720327	86317757	-0.43	6.70E-01	-0.62	5.36E-01
2	rs61620607	86319467	-0.45	6.50E-01	-0.63	5.28E-01
2	rs139549579	86319681	0.43	6.70E-01	0.44	6.56E-01
2	rs10427394	86320542	-0.72	4.70E-01	-0.93	3.51E-01
2	rs10176454	86321548	-0.42	6.80E-01	-0.61	5.43E-01
2	rs715334	86322071	0.21	8.40E-01	0.37	7.12E-01
2	rs2367200	86323069	-0.39	7.00E-01	-0.46	6.42E-01
2	rs74884653	86323339	0.43	6.70E-01	0.45	6.56E-01
2	rs11686963	86324784	-0.44	6.60E-01	-0.63	5.28E-01
2	rs62147717	86325316	-0.40	6.90E-01	-0.48	6.31E-01
2	rs75485563	86325524	0.58	5.60E-01	0.34	7.36E-01
2	rs11127015	86326133	1.12	2.60E-01	0.90	3.68E-01
2	rs12053229	86326201	-0.44	6.60E-01	-0.63	5.28E-01
2	rs10179090	86327820	-0.73	4.70E-01	-0.94	3.47E-01
2	rs725046	86328183	-0.44	6.60E-01	-0.63	5.28E-01
2	rs76052260	86328727	-1.14	2.60E-01	-1.28	2.00E-01
2	rs58919291	86329452	0.39	6.90E-01	0.41	6.84E-01
2	rs11694973	86329561	-0.44	6.60E-01	-0.63	5.28E-01
2	rs77621359	86330353	-1.14	2.60E-01	-1.28	2.00E-01
2	rs145230582	86330539	-0.57	5.70E-01	-0.40	6.86E-01
2	rs10779967	86330726	1.12	2.60E-01	0.90	3.68E-01
2	rs12714168	86331347	-0.44	6.60E-01	-0.63	5.28E-01
2	rs117338609	86332217	0.39	6.90E-01	0.41	6.84E-01
2	rs4832244	86332656	-0.46	6.50E-01	-0.65	5.15E-01
2	rs4832245	86332809	-0.46	6.50E-01	-0.65	5.15E-01
2	rs17026904	86333051	0.39	6.90E-01	0.41	6.84E-01
2	rs2367201	86334199	-0.46	6.50E-01	-0.65	5.15E-01
2	rs3810827	86334386	-1.16	2.50E-01	-1.31	1.91E-01
2	rs3810828	86334566	1.12	2.60E-01	0.90	3.68E-01
2	rs11679343	86335208	-0.46	6.50E-01	-0.65	5.15E-01
2	rs979940	86335718	1.12	2.60E-01	0.90	3.68E-01
2	rs2194486	86336048	-0.42	6.80E-01	-0.50	6.20E-01
2	rs75356890	86336173	0.43	6.70E-01	0.44	6.59E-01
2	rs76870573	86336215	0.39	6.90E-01	0.41	6.84E-01
2	rs56104203	86336256	-1.16	2.50E-01	-1.31	1.91E-01

2	rs11127016	86337104	-0.36	7.20E-01	-0.44	6.58E-01
2	rs1075622	86337930	-0.53	6.00E-01	-0.72	4.71E-01
2	rs59053280	86338400	0.39	6.90E-01	0.41	6.84E-01
2	rs4344936	86338568	-0.42	6.80E-01	-0.50	6.20E-01
2	rs116970226	86338867	0.39	6.90E-01	0.41	6.84E-01
2	rs13013916	86339331	0.17	8.70E-01	0.33	7.41E-01
2	rs115235811	86339474	-1.15	2.50E-01	-1.29	1.95E-01
2	rs3731821	86340791	0.37	7.10E-01	0.33	7.43E-01
2	rs10184246	86341328	0.17	8.70E-01	0.33	7.41E-01
2	rs4832246	86342206	-0.46	6.50E-01	-0.65	5.15E-01
2	rs4832247	86342261	-0.46	6.50E-01	-0.65	5.15E-01
2	rs12617717	86342303	-1.47	1.40E-01	-1.59	1.11E-01
2	rs12624126	86342366	-1.42	1.60E-01	-1.54	1.23E-01
2	rs4832022	86343476	-0.46	6.50E-01	-0.65	5.15E-01
2	rs4832248	86343494	-0.46	6.50E-01	-0.65	5.15E-01
2	rs60460813	86343840	0.37	7.10E-01	0.33	7.43E-01
2	rs3764950	86343911	-0.43	6.70E-01	-0.51	6.12E-01
2	rs3764949	86344095	-0.43	6.70E-01	-0.51	6.12E-01
2	rs3764948	86344237	0.39	7.00E-01	0.35	7.29E-01
2	rs2059353	86344367	1.12	2.60E-01	0.90	3.68E-01
2	rs12621992	86345349	-1.42	1.60E-01	-1.54	1.23E-01
2	rs2367202	86345536	-0.46	6.50E-01	-0.65	5.15E-01
2	rs2303339	86346180	-1.43	1.50E-01	-1.55	1.21E-01
2	rs6715623	86346308	1.12	2.60E-01	0.90	3.68E-01
2	rs147509292	86346517	0.37	7.10E-01	0.33	7.43E-01
2	rs10183744	86346840	-0.46	6.50E-01	-0.65	5.15E-01
2	rs6720143	86347843	-0.91	3.60E-01	-1.11	2.69E-01
2	rs75038217	86348025	-1.42	1.60E-01	-1.54	1.23E-01
2	rs17584697	86348069	-0.40	6.90E-01	-0.48	6.35E-01
2	rs77630851	86348228	0.25	8.00E-01	0.21	8.35E-01
2	rs4832249	86348960	-0.45	6.60E-01	-0.64	5.24E-01
2	rs75104385	86349490	0.37	7.10E-01	0.33	7.43E-01
2	rs1025104	86350664	-0.45	6.60E-01	-0.64	5.24E-01
2	rs6750581	86351240	-0.45	6.60E-01	-0.64	5.24E-01
2	rs144356159	86351684	-0.56	5.80E-01	-0.48	6.31E-01
2	rs4832250	86352010	-0.74	4.60E-01	-0.96	3.37E-01
2	rs4618054	86352785	-0.45	6.60E-01	-0.64	5.24E-01
2	rs6740228	86352825	-0.45	6.60E-01	-0.64	5.24E-01
2	rs74262955	86353401	0.62	5.40E-01	0.70	4.83E-01
2	rs2018608	86353786	-1.41	1.60E-01	-1.53	1.26E-01
2	rs116882766	86353886	0.11	9.10E-01	0.16	8.69E-01
2	rs2018547	86353954	-0.45	6.50E-01	-0.64	5.22E-01
2	rs1019592	86354704	-0.45	6.50E-01	-0.65	5.19E-01
2	rs1834163	86358459	-0.48	6.30E-01	-0.68	4.99E-01
2	rs1424517	86358999	-0.48	6.30E-01	-0.68	4.99E-01
2	rs1559517	86359086	-0.48	6.30E-01	-0.68	4.99E-01

2	rs1559516	86359216	-0.76	4.50E-01	-0.97	3.32E-01
2	rs1559515	86359405	-0.48	6.30E-01	-0.68	4.99E-01
2	rs2241438	86360225	0.01	9.90E-01	0.17	8.61E-01
2	rs2241437	86360573	-1.17	2.40E-01	-1.32	1.88E-01
2	rs2241436	86360809	-0.44	6.60E-01	-0.63	5.26E-01
2	rs7606421	86362204	-0.45	6.50E-01	-0.64	5.21E-01
2	rs3770069	86363074	-0.45	6.60E-01	-0.64	5.25E-01
2	rs2241434	86364353	0.40	6.90E-01	0.35	7.30E-01
2	rs79465176	86364629	-0.31	7.60E-01	-0.09	9.28E-01
2	rs2241433	86364653	0.39	7.00E-01	0.34	7.32E-01
2	rs11395	86365607	1.07	2.90E-01	1.18	2.38E-01
2	rs3088038	86365713	-0.56	5.80E-01	-0.75	4.51E-01
2	rs7598787	86365884	-0.56	5.80E-01	-0.75	4.51E-01
2	rs76096409	86368370	0.49	6.20E-01	0.51	6.12E-01
2	rs10174095	86368392	-1.28	2.00E-01	-1.44	1.51E-01
2	rs3731818	86368804	-0.60	5.50E-01	-0.70	4.86E-01
2	rs4832027	86369700	1.09	2.70E-01	0.87	3.85E-01
2	rs876242	86371012	0.25	8.10E-01	0.41	6.82E-01
2	rs8244	86371883	0.25	8.00E-01	0.41	6.80E-01
2	rs80015710	86375260	0.59	5.60E-01	0.58	5.59E-01
2	rs13018652	86375470	0.13	9.00E-01	0.29	7.73E-01
2	rs1124686	86376474	1.16	2.50E-01	1.34	1.82E-01
2	rs74381597	86376636	0.48	6.30E-01	0.47	6.39E-01
2	rs4569473	86377549	0.54	5.90E-01	0.63	5.27E-01
2	rs12714169	86378292	0.57	5.70E-01	0.67	5.04E-01
2	rs6731535	86380113	0.25	8.00E-01	0.38	7.07E-01
2	rs7608547	86381063	0.25	8.00E-01	0.38	7.07E-01
2	rs10200018	86381496	0.25	8.00E-01	0.38	7.07E-01
2	rs10202995	86382151	0.25	8.00E-01	0.38	7.07E-01
2	rs114534559	86382938	0.51	6.10E-01	0.25	8.01E-01
2	rs6547660	86383171	0.27	7.90E-01	0.39	6.99E-01
2	rs12465983	86383946	0.25	8.00E-01	0.38	7.07E-01
2	rs7561589	86385235	0.25	8.00E-01	0.38	7.07E-01
2	rs1057893	86385668	0.25	8.00E-01	0.38	7.07E-01
2	rs9973320	86388127	0.25	8.00E-01	0.38	7.07E-01
2	rs6737950	86388605	0.25	8.00E-01	0.38	7.07E-01
2	rs11685551	86388889	-0.47	6.40E-01	-0.58	5.60E-01
2	rs10168635	86389421	-1.45	1.50E-01	-1.61	1.09E-01
2	rs6731358	86390714	-0.47	6.40E-01	-0.58	5.60E-01
2	rs12620810	86391481	-1.37	1.70E-01	-1.52	1.29E-01
2	rs3770067	86392071	0.25	8.00E-01	0.38	7.07E-01
2	rs62150072	86394290	0.27	7.90E-01	0.39	6.99E-01
2	rs6740666	86394563	0.30	7.70E-01	0.41	6.84E-01
2	rs111289536	86394870	-1.45	1.50E-01	-1.61	1.09E-01
2	rs1424516	86395273	1.22	2.20E-01	1.00	3.17E-01
2	rs6729732	86395433	0.25	8.00E-01	0.37	7.11E-01

2	rs12478097	86395961	0.25	8.00E-01	0.38	7.07E-01
2	rs2278083	86396719	0.25	8.00E-01	0.38	7.07E-01
2	rs12714171	86397090	0.25	8.00E-01	0.38	7.07E-01
2	rs2288115	86398074	0.27	7.90E-01	0.39	6.99E-01
2	rs2288114	86398206	0.27	7.90E-01	0.39	6.99E-01
2	rs2288113	86398291	0.25	8.00E-01	0.38	7.07E-01
2	rs12477274	86398785	0.27	7.90E-01	0.39	6.98E-01
2	rs6547661	86399219	0.27	7.90E-01	0.39	6.93E-01
2	rs6547662	86399249	0.27	7.90E-01	0.39	6.93E-01
2	rs7609008	86399884	0.25	8.00E-01	0.38	7.07E-01
2	rs12478369	86399954	0.28	7.80E-01	0.41	6.85E-01
2	rs59185549	86400068	0.55	5.80E-01	0.55	5.84E-01
2	rs6707029	86400084	0.25	8.00E-01	0.38	7.07E-01
2	rs6749439	86400092	0.59	5.60E-01	0.69	4.92E-01
2	rs117903699	86400745	-1.51	1.30E-01	-1.65	9.83E-02
2	rs59649940	86400791	0.59	5.60E-01	0.59	5.57E-01
2	rs3770065	86401381	0.25	8.00E-01	0.38	7.07E-01
2	rs6725372	86401723	0.25	8.00E-01	0.38	7.07E-01
2	rs7566721	86402456	0.25	8.00E-01	0.38	7.07E-01
2	rs2367203	86402937	0.25	8.00E-01	0.38	7.07E-01
2	rs3770064	86403415	0.59	5.60E-01	0.59	5.57E-01
2	rs3770062	86403747	0.25	8.00E-01	0.38	7.07E-01
2	rs6720431	86403935	1.22	2.20E-01	1.00	3.17E-01
2	rs3770061	86404101	0.25	8.00E-01	0.38	7.07E-01
2	rs2367204	86404411	0.27	7.90E-01	0.39	6.99E-01
2	rs13403186	86405016	0.59	5.60E-01	0.69	4.92E-01
2	rs6706204	86405407	0.59	5.60E-01	0.69	4.92E-01
2	rs6749090	86405804	0.63	5.30E-01	0.73	4.64E-01
2	rs6547663	86406438	0.25	8.00E-01	0.38	7.07E-01
2	rs3770060	86406889	0.25	8.00E-01	0.38	7.07E-01
2	rs12998025	86407549	0.25	8.00E-01	0.38	7.07E-01
2	rs13024884	86407784	0.25	8.00E-01	0.38	7.07E-01
2	rs6731995	86408385	0.25	8.00E-01	0.38	7.07E-01
2	rs2303338	86408768	-0.46	6.40E-01	-0.58	5.60E-01
2	rs918856	86409239	1.22	2.20E-01	1.00	3.17E-01
2	rs9646999	86410715	0.25	8.00E-01	0.38	7.07E-01
2	rs12469762	86411275	0.27	7.90E-01	0.39	6.99E-01
2	rs7566961	86411607	0.25	8.00E-01	0.38	7.07E-01
2	rs7567380	86411982	-0.81	4.20E-01	-0.87	3.85E-01
2	rs12151585	86412251	0.25	8.00E-01	0.38	7.07E-01
2	rs113324637	86412257	-1.45	1.50E-01	-1.61	1.09E-01
2	rs965636	86413450	-0.46	6.40E-01	-0.58	5.60E-01
2	rs10175330	86414114	0.25	8.00E-01	0.38	7.07E-01
2	rs10188059	86414283	0.25	8.00E-01	0.38	7.07E-01
2	rs77052518	86414323	0.55	5.80E-01	0.55	5.84E-01
2	rs2081322	86414822	-0.46	6.40E-01	-0.58	5.60E-01

2	rs13010906	86414870	0.25	8.00E-01	0.38	7.07E-01
2	rs13011498	86414984	0.25	8.00E-01	0.38	7.07E-01
2	rs7588325	86415573	0.25	8.00E-01	0.38	7.07E-01
2	rs117476275	86415798	0.59	5.60E-01	0.59	5.57E-01
2	rs182530414	86415898	0.66	5.10E-01	0.43	6.67E-01
2	rs58002521	86416426	0.55	5.80E-01	0.55	5.85E-01
2	rs6730226	86416915	0.27	7.90E-01	0.39	6.99E-01
2	rs6719136	86417908	0.25	8.00E-01	0.38	7.07E-01
2	rs6747474	86417975	0.25	8.00E-01	0.38	7.07E-01
2	rs141258610	86418767	0.55	5.80E-01	0.55	5.84E-01
2	rs11884038	86418955	0.25	8.00E-01	0.38	7.07E-01
2	rs76647001	86419523	0.47	6.40E-01	0.42	6.76E-01
2	rs11885380	86419602	0.25	8.00E-01	0.38	7.07E-01
2	rs11897696	86420255	0.25	8.00E-01	0.38	7.07E-01
2	rs12475079	86421209	0.25	8.10E-01	0.37	7.13E-01
2	rs12475126	86421332	0.27	7.90E-01	0.39	6.99E-01
2	rs11899254	86421507	0.25	8.00E-01	0.38	7.07E-01
2	rs12714172	86421702	0.25	8.00E-01	0.38	7.07E-01

a The Z and P value of SNPs from GWAS summary association statistics used in original TWAS analysis implemented in FUSION using GTEx.Esophagus_Mucosa-FUSION models.
b The Z and P value of GWAS signals from the summary-based joint/conditional testing implemented in FUSION using GTEx.Esophagus_Mucosa-FUSION models.

Table S6. Performance of gene expression prediction models for VAMP8 gene

Model[a]	Number[b]	h^2_g	$P\_h^2_g$	Algorithm[c]	R^2	P_R^2	Predicting Variants
GTEx.Esophagus_Mucosa-MetaXcan	307	-	-	Enet	0.23	1.92×10^{-19}	rs4831984, rs11900293, rs17025827, rs874838,rs6731223, rs2289972, rs2028900, rs2592551, rs762684, rs6757263, rs3770098, rs7579147, rs3731828, rs1009, rs1058588, rs7593969, rs1254900, rs1374370, rs7355681, rs11894251, rs11679490, rs11890686, rs1518809, rs1033287, rs11899750, rs10865482
GTEx.Esophagus_Mucosa-FUSION	357	0.39	4.77×10^{-2}	LASSO	0.35	5.35×10^{-35}	rs2028900, rs2028898, rs2592551, rs3731828, rs1058588, rs7593969, rs1254900, rs1374370
NPC-MetaXcan	46	-	-	Enet	0.20	2.00×10^{-3}	rs7578174, rs7564154, rs7590834, rs7567549, rs7581654, rs1918689, rs1918690, rs1918691, rs6749125,rs4832107, rs6729553
NPC-FUSION	46	-0.04	2.07×10^{-1}	-	-	-	-
TCGA.HNSC-FUSION	415	0.05	2.57×10^{-2}	LASSO	0.03	1.25×10^{-4}	rs1446669, rs2028898, rs2592551, rs2232739

a GTEx.Esophagus_Mucosa-MetaXcan: The models building using the GTEx esophagus mucosa tissue dataset computed in MetaXcan software;
GTEx.Esophagus_Mucosa-FUSION: The models building using the GTEx esophagus mucosa tissue dataset computed in FUSION software;
NPC-MetaXcan: The models building using the NPC sample dataset computed in MetaXcan software;
NPC-FUSION: The models building using the NPC sample dataset computed in FUSION software;
TCGA.HNSC-FUSION: The models building using the TCGA.HNSC dataset computed in FUSION software.
b The number of samples used in building gene expression prediction models.
c Enet: elastic-net regression; LASSO: least absolute shrinkage and selection operator.

Table S7. Genes whose predicted expression was associated with risk of NPC after Bonferroni correction in TWAS analysis using NPC-MetaXcan models[a]

Gene	Chr	Start	End	Type	TWAS Z[a]	TWAS P[c]
MICB	6	31462658	31478901	protein_coding	-10.52	7.36×10^{-26}
GABBR1	6	29523406	29601753	protein_coding	8.55	1.21×10^{-17}
GNL1	6	30509154	30524951	protein_coding	8.14	4.10×10^{-16}
TUBB	6	30688129	30693203	protein_coding	7.37	1.72×10^{-13}
ABCF1	6	30539170	30564956	protein_coding	7.37	1.72×10^{-13}
TRIM39	6	30294256	30311506	protein_coding	7.37	1.75×10^{-13}
TRIM26	6	30152232	30181204	protein_coding	7.31	2.65×10^{-13}
NRM	6	30655824	30659197	protein_coding	6.47	9.78×10^{-11}
TRIM27	6	28870779	28891765	protein_coding	6.28	3.31×10^{-10}
VAMP8	2	85788685	85809154	protein_coding	5.62	1.86×10^{-8}
ZDHHC11B	5	710470	784844	protein_coding	4.98	6.35×10^{-7}
TGOLN2	2	85545143	85555419	protein_coding	-4.65	3.34×10^{-6}
IMMT	2	86371055	86422893	protein_coding	-4.65	3.34×10^{-6}

a NPC-MetaXcan: The models building using the NPC sample dataset computed in MetaXcan software.
b Direction and effect size. Positive and negative value suggest associations of up-regulated and down-regulated predicted gene expression with increased NPC risk, respevtively.
c Associations with $P \leqslant 4.05 \times 10^{-6}$ considered statistically significant based on Bonferroni correction of 12,346 tests (0.05/12,346).

Table S8. Genes whose predicted expression was associated with risk of NPC after Bonferroni correction in TWAS analysis using TCGA.HNSC-FUSION models[a]

Gene	Chr	Start	End	Type	TWAS Z[a]	TWAS P[c]
VAMP8	2	85788685	85809154	protein_coding	5.64	1.66×10^{-8}

a TCGA.HNSC-FUSION: The models building using the TCGA.HNSC dataset computed in FUSION software.
b Direction and effect size. Positive value suggests associations of up-regulated predicted gene expression with increased NPC risk.
c Associations with $P \leqslant 2.46 \times 10^{-5}$ considered statistically significant based on Bonferroni correction of 2,031 tests (0.05/2,031).

Table S9. GTEx esophagus mucosa tissue-specific expression quantitative trait loci (eQTL) result at VAMP8 region[a]

SNP[b]	GTEx_variant_id	Effect allele	Transcription_start_site_distance(bp)	β	β_se	P
rs1058588	2_85808871_C_T_b37	C	20186	0.34	0.02	2.86×10^{-35}
rs1972297	2_85808573_T_C_b37	T	19888	0.33	0.02	3.73×10^{-35}
rs3770098	2_85805367_C_A_b37	C	16682	0.34	0.02	8.52×10^{-34}
rs3731827	2_85806068_T_C_b37	T	17383	0.34	0.02	8.52×10^{-34}
rs6757263	2_85803542_T_C_b37	T	14857	0.34	0.02	9.77×10^{-34}
rs1009	2_85808737_A_G_b37	A	20052	0.33	0.02	1.62×10^{-33}
rs1010	2_85808982_T_C_b37	T	20297	0.33	0.02	2.38×10^{-33}
rs6547624	2_85802134_A_T_b37	A	13449	0.34	0.02	7.69×10^{-33}
rs10198569	2_85794415_A_G_b37	A	5730	0.34	0.03	1.87×10^{-32}
rs10187424	2_85794297_T_C_b37	T	5612	0.33	0.02	2.82×10^{-32}
rs35215812	2_85792384_G_A_b37	G	3699	0.33	0.03	7.83×10^{-31}
rs7579147	2_85805654_G_A_b37	G	16969	0.34	0.03	1.89×10^{-30}
rs3731828	2_85806266_C_T_b37	C	17581	0.34	0.03	1.89×10^{-30}
rs3755008	2_85803897_T_C_b37	T	15212	0.34	0.03	4.42×10^{-30}
rs11891260	2_85796101_G_C_b37	G	7416	0.32	0.03	3.90×10^{-29}
rs55692129	2_85792404_A_T_b37	A	3719	0.32	0.03	1.04×10^{-24}
rs11126995	2_85792408_A_T_b37	A	3723	0.31	0.03	1.85×10^{-23}
rs6547622	2_85801902_C_T_b37	C	13217	0.31	0.03	4.96×10^{-23}
rs115669754	2_85788738_C_A_b37	C	53	0.31	0.03	2.70×10^{-18}
rs11890182	2_85790165_C_T_b37	C	1480	0.30	0.03	3.97×10^{-18}
rs142825030	2_85796407_C_T_b37	C	7722	0.30	0.03	5.65×10^{-18}
rs60788310	2_85792063_G_A_b37	G	3378	0.30	0.06	3.72×10^{-7}
rs73943244	2_85795361_G_C_b37	G	6676	0.28	0.06	6.70×10^{-7}
rs12615553	2_85790734_G_A_b37	G	2049	0.28	0.06	6.93×10^{-7}
rs58768785	2_85790435_A_G_b37	A	1750	0.26	0.06	5.41×10^{-6}
rs75577639	2_85800960_C_T_b37	C	12275	0.26	0.07	2.75×10^{-4}
rs145376552	2_85796216_G_A_b37	G	7531	0.23	0.07	1.64×10^{-3}

rs539383605	2_85793394_G_A_b37	G	4709	0.34	0.11	1.86×10^{-3}
rs75830997	2_85789135_G_T_b37	G	450	0.30	0.10	2.25×10^{-3}
rs28564788	2_85796788_C_T_b37	C	8103	0.11	0.05	1.21×10^{-2}
rs34214220	2_85802294_C_T_b37	C	13609	0.11	0.05	1.24×10^{-2}
rs10187218	2_85794114_T_C_b37	T	5429	0.11	0.05	1.30×10^{-2}
rs139775513	2_85792487_A_G_b37	A	3802	0.11	0.05	1.52×10^{-2}
rs10198761	2_85794634_A_T_b37	A	5949	0.11	0.05	1.60×10^{-2}
rs12714146	2_85791852_A_G_b37	A	3167	0.11	0.05	1.71×10^{-2}
rs17430670	2_85795456_G_T_b37	G	6771	0.11	0.05	1.71×10^{-2}
rs13429968	2_85797665_A_G_b37	A	8980	0.11	0.05	1.71×10^{-2}
rs2121397	2_85798858_G_A_b37	G	10173	0.11	0.05	1.71×10^{-2}
rs17508727	2_85799776_G_A_b37	G	11091	0.11	0.05	1.71×10^{-2}
rs13000511	2_85800265_A_C_b37	A	11580	0.11	0.05	1.71×10^{-2}
rs55892665	2_85802754_A_G_b37	A	14069	0.11	0.05	1.71×10^{-2}
rs13425821	2_85803242_G_T_b37	G	14557	0.11	0.05	1.71×10^{-2}
rs10166612	2_85804096_A_G_b37	A	15411	0.11	0.05	1.71×10^{-2}
rs13421434	2_85807610_C_A_b37	C	18925	0.11	0.05	1.71×10^{-2}
rs62165905	2_85792936_G_T_b37	G	4251	0.11	0.05	1.74×10^{-2}
rs71390081	2_85793839_C_CCT_b37	C	5154	0.11	0.05	1.75×10^{-2}
rs13409377	2_85793562_G_A_b37	G	4877	0.11	0.05	1.81×10^{-2}
rs62165907	2_85801137_G_A_b37	G	12452	0.11	0.05	1.85×10^{-2}
rs13432584	2_85801837_T_C_b37	T	13152	0.11	0.05	1.89×10^{-2}
rs34212016	2_85795993_T_C_b37	T	7308	0.10	0.05	2.27×10^{-2}
rs13426038	2_85803398_G_C_b37	G	14713	0.10	0.04	3.05×10^{-2}
rs113903338	2_85790873_G_A_b37	G	2188	-0.24	0.14	9.64×10^{-2}
rs1378933346	2_85798535_TGA_T_b37	TGA	9850	-0.20	0.14	1.35×10^{-1}
rs200007992	2_85798530_C_CTAAA_b37	C	9845	-0.20	0.14	1.36×10^{-1}
rs1456628402	2_85798531_ACG_A_b37	ACG	9846	-0.20	0.14	1.36×10^{-1}
rs145176428	2_85791341_T_A_b37	T	2656	-0.20	0.14	1.38×10^{-1}

rs142869834	2_85792286_C_T_b37	C	3601	-0.20	0.14	1.38×10^{-1}
rs144476440	2_85797912_C_T_b37	C	9227	-0.20	0.14	1.38×10^{-1}
rs139977225	2_85805373_T_C_b37	T	16688	-0.20	0.14	1.38×10^{-1}
rs140052824	2_85806332_G_C_b37	G	17647	-0.20	0.14	1.38×10^{-1}
rs72843861	2_85808087_C_T_b37	C	19402	-0.09	0.06	1.49×10^{-1}
rs62165906	2_85801108_C_T_b37	C	12423	0.08	0.05	1.55×10^{-1}
rs148224585	2_85803963_G_A_b37	G	15278	-0.16	0.12	1.67×10^{-1}
rs140720322	2_85796060_G_A_b37	G	7375	-0.15	0.11	1.74×10^{-1}
rs114662252	2_85798049_G_A_b37	G	9364	-0.15	0.11	1.74×10^{-1}
rs964716373	2_85801066_AATT_A_b37	AATT	12381	0.15	0.13	2.32×10^{-1}
rs147698609	2_85802709_C_T_b37	C	14024	-0.13	0.11	2.41×10^{-1}
rs141917851	2_85791786_T_C_b37	T	3101	-0.16	0.13	2.47×10^{-1}
rs143580678	2_85792493_G_A_b37	G	3808	-0.16	0.13	2.48×10^{-1}
rs147424158	2_85792446_C_T_b37	C	3761	-0.15	0.13	2.50×10^{-1}
rs75259224	2_85804055_G_A_b37	G	15370	-0.15	0.13	2.50×10^{-1}
rs60190586	2_85805115_C_T_b37	C	16430	-0.15	0.13	2.50×10^{-1}
rs112616182	2_85799361_G_C_b37	G	10676	-0.15	0.13	2.64×10^{-1}
rs111868538	2_85799203_A_G_b37	A	10518	-0.15	0.13	2.70×10^{-1}
rs79470465	2_85788818_A_G_b37	A	133	-0.09	0.11	4.31×10^{-1}
rs6707308	2_85789906_G_A_b37	G	1221	-0.05	0.07	4.69×10^{-1}
rs72843854	2_85797329_T_C_b37	T	8644	-0.01	0.08	8.62×10^{-1}

a VAMP8 region is defined as chr2: 85,788,685-85,809,154 (hg19) based on the ENSEMBL database.
b SNPs in bold were included in GWAS summary association statistics uesd in TWAS analysis.

Table S10. The results of COJO conditional testing of VAMP8 signal

Model[a]	TWAS Z[b]	TWAS P[b]	Adjusted Z[c]	Adjusted P[c]
GTEx.Esophagus_Mucosa-MetaXcan	5.36	8.24×10^{-8}	0.15	8.77×10^{-1}
GTEx.Esophagus_Mucosa-FUSION	5.50	3.60×10^{-8}	0.03	9.78×10^{-1}

a GTEx.Esophagus_Mucosa-MetaXcan: The models building using the GTEx esophagus mucosa tissue dataset computed in MetaXcan software;
GTEx.Esophagus_Mucosa-FUSION: The models building using the GTEx esophagus mucosa tissue dataset computed in FUSION software.
b The Z and P value of VAMP8 signal from original TWAS analysis.
c The Z and P value of VAMP8 signal from COJO conditional testing.

Table S11. Associations for SNPs on VAMP8 region surpassing genome-wide significance in the GWAS summary data used in TWAS analysis

CHR[a]	SNP	POS[a]	Effect allele	Reference allele	Effect allele frequency		OR[b]	P	r^{2c}	Adjusted P[d]	Function
					Case	Control					
2	rs35215812	85792384	G	A	0.65	0.60	1.28	1.73×10^{-8}	0.99	0.22	intronic
2	rs55692129	85792404	A	T	0.74	0.69	1.31	2.20×10^{-8}	0.70	0.07	intronic
2	rs11126995	85792408	A	T	0.71	0.66	1.31	5.10×10^{-9}	0.75	0.03	intronic
2	rs10187424	85794297	T	C	0.65	0.60	1.28	1.48×10^{-8}	0.99	0.22	intronic
2	rs10198569	85794415	A	G	0.65	0.60	1.28	1.51×10^{-8}	0.99	0.22	intronic
2	rs11891260	85796101	G	C	0.65	0.60	1.28	2.13×10^{-8}	0.99	0.37	intronic
2	rs6547622	85801902	C	T	0.72	0.67	1.29	3.33×10^{-8}	0.73	0.10	intronic
2	rs6547623	85801964	T	A	0.71	0.67	1.30	1.01×10^{-8}	0.76	0.05	intronic
2	rs6547624	85802134	A	T	0.65	0.60	1.28	1.19×10^{-8}	0.99	0.02	intronic
2	rs6757263	85803542	T	C	0.65	0.60	1.28	1.38×10^{-8}	1.00	0.03	intronic
2	rs3755008	85803897	T	C	0.72	0.67	1.30	2.65×10^{-8}	0.73	0.12	intronic
2	rs3770098	85805367	C	A	0.65	0.60	1.28	2.20×10^{-8}	1.00	0.12	intronic
2	rs7579147	85805654	G	A	0.72	0.67	1.29	4.70×10^{-8}	0.73	0.15	intronic
2	rs3731827	85806068	T	C	0.65	0.60	1.27	4.82×10^{-8}	1	NA	intronic
2	rs1972297	85808573	T	C	0.65	0.60	1.27	3.73×10^{-8}	1	NA	intronic
2	rs1009	85808737	A	G	0.65	0.60	1.27	3.73×10^{-8}	1	NA	synonymous
2	rs1058588	85808871	C	T	0.65	0.60	1.27	3.73×10^{-8}	-	NA	3'UTR

a VAMP8 region is defined as chr2: 85,788,685-85,809,154 (hg19) based on the ENSEMBL database; CHR, chromosome number; POS, base pair position on chromosome 2.
b Odds Ratio.
c Linkage disequilibrium with rs1058588.
d P value after adjustment for rs1058588.

Table S12. Differentially expressed genes identified by RNA-seq analysis on S26 cells with VAMP8 knockdown

Row.names	baseMean	log2FoldChange	lfcSE	stat	P	Padj
GT	16.7	7.1	2.4	3.0	2.48E-03	3.64E-02
NPR1	14.4	6.9	1.8	3.9	1.01E-04	3.29E-03
NTS	9.8	6.4	1.7	3.7	2.45E-04	6.28E-03
FLNC	50.2	6.3	1.6	3.9	1.01E-04	3.30E-03
F7	7.5	6.0	1.7	3.5	4.08E-04	9.14E-03
TTYH1	6.9	5.9	1.9	3.1	1.84E-03	2.91E-02
RSPO2	6.8	5.8	1.8	3.3	1.06E-03	1.91E-02
FGF21	6.2	5.7	1.8	3.1	1.77E-03	2.83E-02
AQP6	6.2	5.7	1.8	3.1	2.03E-03	3.14E-02
MFAP4	29.8	5.5	1.6	3.4	5.82E-04	1.21E-02
CRYAB	4.7	5.3	1.8	2.9	3.46E-03	4.56E-02
CCL20	97.4	5.2	1.8	2.9	3.61E-03	4.70E-02
INHBA	450.9	5.0	1.5	3.4	5.63E-04	1.18E-02
KRT34	68.6	4.9	1.4	3.5	4.40E-04	9.54E-03
SLC6A20	19.1	4.9	1.7	2.9	3.28E-03	4.42E-02
RGS16	35.3	4.7	0.9	5.2	1.78E-07	1.96E-05
CGA	49.2	4.6	0.8	5.8	5.93E-09	9.82E-07
C11orf96	32.3	4.6	1.4	3.4	6.60E-04	1.32E-02
TEX19	95.5	4.6	1.1	4.1	3.42E-05	1.43E-03
INHBE	109.3	4.4	0.6	7.7	1.05E-14	5.64E-12
PRAP1	46.9	4.1	1.2	3.4	6.06E-04	1.25E-02
IL21R	14.8	3.9	1.1	3.7	2.30E-04	5.96E-03
PRSS56	39.2	3.9	1.3	3.0	2.38E-03	3.53E-02
AREG	253.4	3.9	0.7	5.8	5.54E-09	9.30E-07
IL6	96.9	3.6	0.8	4.6	3.80E-06	2.64E-04
TCIM	237.9	3.6	0.6	6.0	2.63E-09	4.66E-07
FAM186B	10.6	3.4	1.1	3.2	1.56E-03	2.57E-02
ATF3	1217.3	3.3	0.6	5.4	6.43E-08	8.07E-06
RSPO3	31.8	3.2	0.7	4.6	5.09E-06	3.31E-04
HIST1H2BG	15.4	3.2	0.9	3.4	7.29E-04	1.42E-02
INSL4	82.4	3.2	1.0	3.1	2.23E-03	3.37E-02
SH2D6	14.9	3.1	1.1	2.9	3.31E-03	4.44E-02
NEDD9	115.6	3.1	0.7	4.4	1.30E-05	6.63E-04
ROS1	140.0	3.0	0.6	5.3	1.02E-07	1.20E-05
EDN1	198.5	3.0	0.7	4.3	1.69E-05	8.16E-04
MMP13	64.8	3.0	0.8	3.7	2.01E-04	5.45E-03
CXCL8	480.7	3.0	0.8	3.9	1.17E-04	3.68E-03
ANKRD1	305.7	3.0	0.7	4.0	7.05E-05	2.50E-03
KLRC4	31.7	2.9	0.9	3.4	5.81E-04	1.21E-02
COL6A3	31.6	2.9	0.8	3.9	1.02E-04	3.32E-03
COL2A1	18.8	2.8	0.8	3.5	4.34E-04	9.43E-03

TSPAN8	42.0	2.7	0.9	3.0	2.38E-03	3.53E-02
P2RY6	127.3	2.7	0.8	3.3	9.47E-04	1.74E-02
TAGLN	85.5	2.7	0.7	4.0	7.28E-05	2.56E-03
SPRR2D	28.5	2.6	0.7	4.0	5.62E-05	2.09E-03
IFNL1	25.8	2.5	0.8	3.1	1.96E-03	3.06E-02
GPR156	17.8	2.5	0.7	3.5	5.00E-04	1.06E-02
DUSP2	1032.9	2.5	0.3	7.9	2.32E-15	1.43E-12
CX3CL1	51.0	2.5	0.6	4.2	2.55E-05	1.13E-03
CHAC1	459.5	2.4	0.8	3.1	2.13E-03	3.25E-02
ALPI	286.5	2.4	0.7	3.7	1.97E-04	5.41E-03
BMP2	1089.9	2.4	0.3	8.6	9.65E-18	7.79E-15
RRAD	198.5	2.4	0.7	3.2	1.17E-03	2.04E-02
ZBTB32	156.4	2.4	0.8	3.0	3.15E-03	4.30E-02
CREB5	126.0	2.4	0.3	6.9	3.65E-12	1.28E-09
KLRK1	100.2	2.3	0.7	3.5	5.00E-04	1.06E-02
PTGS2	481.6	2.3	0.7	3.1	1.91E-03	3.01E-02
ARC	328.9	2.3	0.6	3.7	1.79E-04	5.02E-03
QRFP	18.3	2.2	0.8	2.9	3.72E-03	4.80E-02
IGF2	62.9	2.2	0.6	3.5	4.29E-04	9.36E-03
IFI27	168.4	2.2	0.4	5.0	4.94E-07	4.66E-05
GANAB	22528.8	2.2	0.3	8.6	8.62E-18	7.43E-15
SPP1	456.8	2.1	0.2	9.2	2.39E-20	5.15E-17
NR4A1	1884.5	2.1	0.4	5.2	2.17E-07	2.34E-05
SGK1	1342.2	2.1	0.7	3.2	1.59E-03	2.61E-02
MPZ	95.0	2.1	0.5	4.4	8.75E-06	5.03E-04
FABP3	42.4	2.1	0.6	3.5	4.30E-04	9.36E-03
JSRP1	28.7	2.1	0.5	3.9	1.00E-04	3.28E-03
BMPER	95.5	2.0	0.4	5.8	8.09E-09	1.26E-06
ACSBG1	23.6	2.0	0.7	3.1	1.92E-03	3.01E-02
BBC3	692.8	2.0	0.3	6.0	1.81E-09	3.59E-07
CPS1	16899.7	2.0	0.5	3.9	1.03E-04	3.34E-03
CLIC5	71.6	2.0	0.5	4.0	5.70E-05	2.10E-03
NPC1L1	16.9	1.9	0.7	2.9	3.73E-03	4.80E-02
KLRC2	188.0	1.9	0.6	3.5	5.53E-04	1.16E-02
DQX1	37.8	1.9	0.6	3.3	8.93E-04	1.66E-02
CES1	32.1	1.9	0.6	3.3	9.02E-04	1.67E-02
DNAH3	20.9	1.9	0.6	3.0	2.89E-03	4.06E-02
HOXB9	114.9	1.9	0.5	4.0	6.37E-05	2.29E-03
FN1	12840.5	1.9	0.2	9.8	6.95E-23	1.80E-19
SOD2	10255.7	1.9	0.5	3.6	3.64E-04	8.45E-03
ADM2	438.5	1.8	0.3	5.8	7.75E-09	1.24E-06
WNT10B	366.9	1.8	0.6	3.1	2.25E-03	3.38E-02
AKR1C2	1975.2	1.8	0.5	3.8	1.30E-04	3.96E-03
DDIT3	448.6	1.8	0.3	6.3	2.40E-10	6.08E-08
PLXNA4	50.3	1.8	0.5	3.8	1.52E-04	4.45E-03
RPS29	1730.2	1.8	0.5	3.6	3.26E-04	7.79E-03

MCOLN3	21.8	1.8	0.6	2.9	3.21E-03	4.36E-02
IQCN	58.6	1.8	0.5	3.3	1.02E-03	1.84E-02
DIO2	1411.7	1.7	0.2	8.7	4.13E-18	4.10E-15
ZNF616	295.7	1.7	0.3	5.5	4.11E-08	5.30E-06
SMOC1	257.4	1.7	0.3	6.0	2.47E-09	4.43E-07
RASD1	208.5	1.7	0.4	4.1	3.52E-05	1.46E-03
EPHA4	101.0	1.7	0.4	4.5	5.88E-06	3.71E-04
DCAF4L1	24.3	1.7	0.6	2.9	3.57E-03	4.67E-02
SVEP1	435.5	1.7	0.5	3.2	1.40E-03	2.35E-02
CYP51A1	404.1	1.7	0.5	3.6	3.26E-04	7.79E-03
PMAIP1	2184.3	1.7	0.5	3.2	1.53E-03	2.53E-02
C1S	911.2	1.7	0.4	4.0	6.63E-05	2.36E-03
COL4A1	5602.2	1.7	0.2	8.1	3.82E-16	2.60E-13
RPLP1	27231.7	1.7	0.3	5.3	1.13E-07	1.31E-05
CCDC17	38.9	1.6	0.5	3.4	6.77E-04	1.35E-02
FLRT2	275.2	1.6	0.3	5.7	1.31E-08	1.90E-06
NR4A2	246.1	1.6	0.5	3.4	6.19E-04	1.26E-02
VGF	217.6	1.6	0.5	3.0	2.75E-03	3.91E-02
HLA-B	416.0	1.6	0.5	3.2	1.54E-03	2.55E-02
PDGFRB	54.2	1.6	0.5	3.3	1.12E-03	1.98E-02
SESN2	2066.1	1.6	0.4	3.6	2.96E-04	7.25E-03
FOXP4	3053.7	1.6	0.2	10.0	1.25E-23	4.03E-20
TCN1	111.2	1.6	0.5	2.9	3.42E-03	4.53E-02
NTN1	959.1	1.6	0.3	4.7	2.76E-06	2.00E-04
RHEBL1	311.7	1.6	0.3	4.7	2.38E-06	1.79E-04
RPS12	20689.0	1.6	0.4	4.2	2.44E-05	1.11E-03
NECTIN4	276.7	1.6	0.3	5.0	6.11E-07	5.56E-05
PCSK9	18068.4	1.6	0.5	3.3	8.58E-04	1.61E-02
MRPL53	55.5	1.5	0.5	3.4	6.10E-04	1.25E-02
NFKBIZ	352.2	1.5	0.2	7.1	1.06E-12	4.28E-10
COL1A1	3148.3	1.5	0.3	5.2	1.97E-07	2.14E-05
SLC7A2	856.1	1.5	0.3	4.4	9.66E-06	5.40E-04
TPM4	12453.9	1.5	0.2	7.0	3.56E-12	1.28E-09
RPS21	7334.6	1.5	0.3	4.4	1.10E-05	5.88E-04
SLC27A5	430.1	1.5	0.4	3.6	3.00E-04	7.34E-03
FLRT3	65.6	1.5	0.5	3.0	2.55E-03	3.71E-02
ZNF134	802.3	1.5	0.2	6.2	7.56E-10	1.66E-07
MGAT2	69.7	1.5	0.5	3.3	9.27E-04	1.71E-02
PI16	105.7	1.5	0.4	4.1	3.67E-05	1.50E-03
RPIA	1405.7	1.5	0.3	4.9	8.19E-07	7.20E-05
DACT2	317.4	1.5	0.5	3.0	2.91E-03	4.06E-02
KLHDC3	4507.4	1.5	0.2	6.1	9.94E-10	2.04E-07
SYNE4	104.4	1.5	0.3	4.3	1.61E-05	7.80E-04
BAMBI	543.3	1.5	0.3	4.6	3.65E-06	2.55E-04
ZBED8	96.0	1.4	0.3	4.5	6.63E-06	4.06E-04
HIST1H2BJ	112.3	1.4	0.3	4.3	1.75E-05	8.38E-04

AP1M2	2060.0	1.4	0.3	4.2	3.32E-05	1.39E-03
KCNJ12	901.9	1.4	0.4	3.8	1.72E-04	4.92E-03
ADM5	140.1	1.4	0.5	3.0	2.67E-03	3.82E-02
ZNF544	616.6	1.4	0.2	7.3	3.64E-13	1.52E-10
IFI6	457.8	1.4	0.4	3.2	1.33E-03	2.27E-02
GDF15	1601.3	1.4	0.2	5.6	2.63E-08	3.54E-06
PSAT1	3614.2	1.3	0.4	3.3	8.77E-04	1.63E-02
GADD45G	107.8	1.3	0.4	3.7	2.15E-04	5.69E-03
SP8	262.2	1.3	0.2	6.5	6.05E-11	1.82E-08
PLCG2	609.8	1.3	0.3	4.1	4.10E-05	1.65E-03
IER2	3970.4	1.3	0.4	3.0	3.01E-03	4.16E-02
GPR3	189.4	1.3	0.3	5.1	3.44E-07	3.45E-05
MAPK13	161.9	1.3	0.2	5.2	1.61E-07	1.84E-05
DIP2C	421.2	1.3	0.3	4.6	5.02E-06	3.28E-04
PODXL2	1630.5	1.3	0.3	4.8	1.92E-06	1.50E-04
DUSP8	1122.9	1.3	0.3	4.6	4.28E-06	2.87E-04
LMTK3	954.6	1.3	0.3	3.8	1.75E-04	4.96E-03
JOSD2	549.0	1.3	0.3	3.9	1.07E-04	3.44E-03
COL7A1	27944.4	1.3	0.2	5.2	2.52E-07	2.67E-05
LONP1	10227.9	1.3	0.3	5.0	5.30E-07	4.89E-05
CDK14	217.9	1.3	0.3	4.5	7.49E-06	4.42E-04
VASN	2373.2	1.3	0.4	3.0	2.42E-03	3.56E-02
KCP	233.4	1.3	0.3	3.8	1.22E-04	3.76E-03
S100P	5725.2	1.3	0.3	4.7	2.70E-06	1.98E-04
ID3	13407.8	1.2	0.3	4.2	3.24E-05	1.36E-03
KLF4	3823.6	1.2	0.3	4.0	7.77E-05	2.71E-03
ISG15	8797.6	1.2	0.3	4.5	6.58E-06	4.05E-04
LYPD6	135.2	1.2	0.4	3.3	8.36E-04	1.59E-02
NPM3	1508.2	1.2	0.4	3.4	6.07E-04	1.25E-02
ACVRL1	173.3	1.2	0.4	3.3	9.15E-04	1.69E-02
BICD2	3050.2	1.2	0.2	5.4	7.66E-08	9.27E-06
DENND5A	3436.3	1.2	0.2	8.0	1.01E-15	6.53E-13
CLDN15	270.0	1.2	0.3	4.6	3.86E-06	2.65E-04
PGBD5	245.8	1.2	0.3	4.4	1.17E-05	6.10E-04
SPRN	233.8	1.2	0.4	3.0	2.55E-03	3.71E-02
GPATCH3	661.5	1.2	0.2	5.0	5.04E-07	4.69E-05
CHST14	625.3	1.2	0.3	4.5	6.29E-06	3.91E-04
ECI1	2063.4	1.2	0.2	5.1	2.96E-07	3.01E-05
INSIG1	4050.9	1.2	0.2	6.2	4.50E-10	1.06E-07
STK40	2824.8	1.2	0.2	5.4	5.18E-08	6.63E-06
DHRS2	109.1	1.2	0.4	2.9	3.65E-03	4.73E-02
LAMA4	80.4	1.2	0.3	3.7	1.96E-04	5.41E-03
LRRC37A	58.0	1.2	0.4	3.0	2.31E-03	3.45E-02
CSRNP1	1071.6	1.2	0.4	3.0	2.47E-03	3.63E-02
DDAH2	808.1	1.2	0.2	5.4	6.49E-08	8.07E-06
VANGL2	91.3	1.2	0.4	3.1	1.92E-03	3.01E-02

FAM83E	80.8	1.2	0.4	3.0	2.61E-03	3.77E-02
OTUD1	1070.3	1.1	0.2	4.9	1.13E-06	9.57E-05
LPCAT1	9374.8	1.1	0.3	3.3	9.92E-04	1.81E-02
RXRB	1096.7	1.1	0.3	4.1	3.80E-05	1.55E-03
PRMT6	1023.4	1.1	0.2	5.9	3.52E-09	6.06E-07
TSPAN9	2267.2	1.1	0.4	3.0	2.76E-03	3.91E-02
DHX58	840.1	1.1	0.3	4.2	2.88E-05	1.24E-03
R3HCC1	1037.2	1.1	0.3	3.6	3.47E-04	8.12E-03
HDAC9	170.9	1.1	0.4	3.0	2.73E-03	3.89E-02
ZFYVE21	683.3	1.1	0.3	4.5	7.90E-06	4.62E-04
ZCCHC2	822.1	1.1	0.3	4.4	1.01E-05	5.53E-04
DDN	362.7	1.1	0.3	3.9	8.60E-05	2.92E-03
MESD	2192.6	1.1	0.2	6.4	1.99E-10	5.15E-08
BRF2	401.0	1.1	0.3	3.7	2.07E-04	5.54E-03
PERP	13027.6	1.1	0.1	7.7	1.58E-14	8.19E-12
KAZALD1	803.1	1.1	0.3	4.3	1.43E-05	7.17E-04
KIAA0319	964.1	1.1	0.3	3.9	1.15E-04	3.63E-03
ZSCAN29	1049.0	1.1	0.2	6.8	7.39E-12	2.45E-09
PSMC3	12617.1	1.1	0.2	6.4	1.70E-10	4.56E-08
FRAT2	927.8	1.1	0.2	5.8	5.43E-09	9.23E-07
RGS3	465.6	1.1	0.3	3.9	8.67E-05	2.93E-03
NOSIP	2851.3	1.1	0.3	4.1	4.48E-05	1.76E-03
ATF4	12576.6	1.1	0.3	3.4	6.03E-04	1.25E-02
ZNRD1	284.8	1.1	0.4	3.0	2.45E-03	3.61E-02
USP18	946.1	1.1	0.2	4.6	4.24E-06	2.87E-04
SERPINI1	67.3	1.1	0.3	3.1	2.10E-03	3.21E-02
CNPPD1	980.0	1.1	0.3	3.9	8.85E-05	2.96E-03
SIAH2	1977.4	1.1	0.3	3.4	6.88E-04	1.37E-02
ZCCHC3	1008.3	1.1	0.3	3.8	1.23E-04	3.79E-03
APOL1	1193.7	1.1	0.4	3.0	2.70E-03	3.85E-02
POLR2C	2876.2	1.1	0.2	5.1	4.08E-07	4.00E-05
SDF2L1	990.2	1.1	0.3	3.1	1.71E-03	2.77E-02
ARID3A	1572.3	1.1	0.3	3.8	1.34E-04	4.06E-03
PCNX3	9093.8	1.1	0.2	5.7	1.30E-08	1.90E-06
NAB2	1912.2	1.1	0.2	4.3	1.46E-05	7.22E-04
GABARAPL1	5512.9	1.0	0.3	3.1	1.82E-03	2.89E-02
ZNF84	109.6	1.0	0.3	3.0	2.36E-03	3.51E-02
CENPW	609.7	1.0	0.2	6.0	2.06E-09	3.91E-07
ACO2	4865.1	1.0	0.2	4.4	1.31E-05	6.67E-04
RHBDD3	1709.9	1.0	0.2	5.1	3.53E-07	3.48E-05
CHRNA9	797.6	1.0	0.2	4.5	6.77E-06	4.07E-04
PHF5A	1665.3	1.0	0.3	3.0	2.52E-03	3.68E-02
ZBTB42	157.0	1.0	0.3	3.4	7.15E-04	1.40E-02
PLEKHJ1	3109.3	1.0	0.3	3.8	1.21E-04	3.76E-03
AUP1	6309.9	1.0	0.3	3.8	1.63E-04	4.68E-03
OGFR	5543.7	1.0	0.2	5.5	3.19E-08	4.17E-06

TMED9	7468.2	1.0	0.2	4.2	3.22E-05	1.36E-03
RPL26L1	788.8	1.0	0.2	4.2	3.06E-05	1.31E-03
MZT1	591.4	1.0	0.3	3.9	1.17E-04	3.67E-03
CSF1	4755.6	1.0	0.3	3.1	1.95E-03	3.05E-02
PTGER4	179.5	1.0	0.3	3.3	8.52E-04	1.60E-02
EGLN2	3699.3	1.0	0.2	5.4	5.51E-08	6.98E-06
GRB10	3032.6	1.0	0.2	5.6	2.42E-08	3.37E-06
FRRS1	426.2	1.0	0.3	2.9	3.28E-03	4.42E-02
CORO6	381.1	1.0	0.3	4.1	4.88E-05	1.86E-03
ZW10	899.4	1.0	0.3	3.6	3.19E-04	7.72E-03
HMGCS1	4060.2	1.0	0.2	4.4	1.16E-05	6.10E-04
NAT14	974.6	1.0	0.3	3.1	1.77E-03	2.83E-02
LSM7	1589.6	1.0	0.2	4.2	3.13E-05	1.33E-03
PHGDH	4124.6	1.0	0.3	3.4	6.32E-04	1.28E-02
GPX2	472.2	1.0	0.2	4.5	6.71E-06	4.07E-04
PLAU	208.2	1.0	0.3	4.0	6.49E-05	2.33E-03
MCRIP2	2106.1	1.0	0.3	3.8	1.63E-04	4.69E-03
NDUFAF8	1663.7	1.0	0.2	4.3	1.87E-05	8.76E-04
DNASE1	815.4	1.0	0.2	5.0	4.78E-07	4.55E-05
DUSP16	2178.8	1.0	0.2	6.1	8.95E-10	1.90E-07
VAMP8	1302.2	-4.7	0.4	-12.3	8.85E-35	1.14E-30
SNCB	7.0	-4.2	1.4	-2.9	3.20E-03	4.36E-02
IL1RL1	87.1	-3.4	0.5	-6.2	5.05E-10	1.16E-07
HEPHL1	32.2	-3.1	0.6	-4.9	1.09E-06	9.23E-05
SBK1	12.6	-2.8	0.8	-3.5	4.29E-04	9.36E-03
CORO2B	130.8	-2.8	0.3	-8.7	3.96E-18	4.10E-15
CYP2E1	13.9	-2.7	0.9	-3.0	2.41E-03	3.56E-02
ALDH3A1	442.7	-2.7	0.4	-6.5	6.76E-11	1.99E-08
CSDC2	54.9	-2.6	0.5	-4.9	1.06E-06	9.10E-05
SLC45A1	25.1	-2.6	0.7	-4.0	7.67E-05	2.68E-03
ATP6V0E2	136.5	-2.2	0.3	-7.3	3.03E-13	1.30E-10
CTSS	145.0	-2.2	0.3	-8.6	7.16E-18	6.61E-15
CD300C	15.0	-2.2	0.7	-3.3	8.68E-04	1.62E-02
GRAMD1C	29.4	-2.1	0.7	-3.0	2.35E-03	3.50E-02
TGM2	12885.8	-2.1	0.2	-10.1	3.56E-24	1.53E-20
TMEM158	141.5	-2.1	0.6	-3.7	2.13E-04	5.66E-03
EVI2B	69.9	-2.0	0.4	-4.7	2.29E-06	1.74E-04
SEC14L2	365.2	-2.0	0.3	-7.5	4.57E-14	2.11E-11
TRNP1	326.6	-2.0	0.2	-9.1	6.82E-20	9.79E-17
SCAMP5	52.2	-2.0	0.5	-4.3	1.45E-05	7.19E-04
BEST3	33.9	-2.0	0.5	-3.9	9.72E-05	3.21E-03
PLCH2	49.9	-2.0	0.4	-5.1	3.48E-07	3.46E-05
ELK3	1457.4	-2.0	0.5	-3.9	8.67E-05	2.93E-03
SCART1	44.0	-2.0	0.4	-4.7	2.07E-06	1.60E-04
NPHP1	33.2	-1.9	0.6	-3.4	6.33E-04	1.28E-02
SLC47A2	38.9	-1.9	0.6	-3.1	1.89E-03	2.98E-02

NT5E	15960.8	-1.9	0.2	-9.2	4.72E-20	7.63E-17
DNAH5	58.0	-1.9	0.4	-5.4	7.68E-08	9.27E-06
ARPIN	201.1	-1.9	0.4	-5.1	4.33E-07	4.21E-05
DPEP1	27.8	-1.9	0.6	-3.0	2.38E-03	3.53E-02
ZNF117	79.7	-1.9	0.6	-3.2	1.48E-03	2.47E-02
MMP1	499.5	-1.8	0.3	-5.7	1.45E-08	2.08E-06
FKBP7	27.2	-1.8	0.6	-3.1	2.16E-03	3.29E-02
SLC66A3	308.5	-1.8	0.3	-5.9	3.21E-09	5.60E-07
IL1B	597.5	-1.8	0.4	-4.3	1.93E-05	8.95E-04
SULT2B1	257.5	-1.8	0.4	-4.3	1.43E-05	7.17E-04
MBOAT2	2052.7	-1.8	0.4	-4.8	1.47E-06	1.20E-04
GM2A	467.8	-1.8	0.3	-5.1	2.74E-07	2.84E-05
MALL	590.9	-1.8	0.2	-8.2	2.67E-16	1.92E-13
FHAD1	21.5	-1.8	0.6	-2.9	3.26E-03	4.40E-02
IFITM10	440.0	-1.8	0.3	-5.5	3.14E-08	4.14E-06
CRAT	49.9	-1.8	0.4	-4.4	9.24E-06	5.26E-04
TMCC3	381.9	-1.8	0.5	-3.6	3.84E-04	8.76E-03
CAPN5	403.2	-1.8	0.5	-3.5	3.85E-04	8.76E-03
IQCK	33.4	-1.7	0.5	-3.2	1.39E-03	2.34E-02
KRT13	5982.3	-1.7	0.5	-3.5	4.26E-04	9.36E-03
GGT5	212.3	-1.7	0.4	-4.4	1.33E-05	6.75E-04
EI24	2631.2	-1.7	0.3	-5.2	1.67E-07	1.90E-05
CDK5R2	29.5	-1.7	0.5	-3.2	1.57E-03	2.58E-02
HCAR2	222.3	-1.7	0.4	-4.1	4.42E-05	1.75E-03
SUSD2	3151.9	-1.7	0.5	-3.7	2.03E-04	5.46E-03
PHOSPHO1	20.4	-1.7	0.5	-3.1	1.77E-03	2.83E-02
FBLN5	24.4	-1.7	0.5	-3.2	1.16E-03	2.03E-02
NUDT18	198.5	-1.7	0.3	-5.6	2.55E-08	3.50E-06
ALDH1A3	1500.2	-1.7	0.2	-8.2	2.46E-16	1.87E-13
NAV1	983.5	-1.6	0.4	-4.6	3.87E-06	2.65E-04
C3orf18	100.8	-1.6	0.5	-3.1	1.75E-03	2.82E-02
VAMP5	44.8	-1.6	0.4	-3.8	1.27E-04	3.89E-03
LHX1	421.2	-1.6	0.3	-6.1	9.68E-10	2.02E-07
IGF2BP2	3139.5	-1.6	0.2	-7.7	1.68E-14	8.36E-12
IRF5	85.4	-1.5	0.4	-3.9	8.39E-05	2.86E-03
MMP28	491.3	-1.5	0.3	-5.3	9.79E-08	1.16E-05
TGFB1I1	240.6	-1.5	0.2	-6.9	6.00E-12	2.04E-09
TRPV4	168.5	-1.5	0.3	-5.8	6.93E-09	1.12E-06
PKIA	84.1	-1.5	0.5	-3.3	8.72E-04	1.63E-02
PKDCC	87.7	-1.5	0.5	-2.9	3.36E-03	4.48E-02
ANGPTL4	693.8	-1.5	0.4	-3.5	3.86E-04	8.76E-03
ABCA3	103.0	-1.5	0.4	-3.7	1.77E-04	4.99E-03
BHLHE41	751.5	-1.5	0.2	-7.0	3.41E-12	1.28E-09
CYSRT1	261.1	-1.5	0.5	-3.1	2.28E-03	3.43E-02
SLC44A3	100.3	-1.5	0.3	-5.1	2.72E-07	2.84E-05
LIMA1	9343.2	-1.5	0.3	-4.7	2.42E-06	1.81E-04

ZBTB47	385.4	-1.5	0.3	-5.0	7.22E-07	6.52E-05
KIAA0040	840.7	-1.5	0.2	-7.9	2.79E-15	1.64E-12
ANXA2	141497.2	-1.5	0.1	-11.9	1.80E-32	1.16E-28
CDK19	470.5	-1.5	0.2	-6.0	2.37E-09	4.31E-07
ARL4C	478.5	-1.5	0.2	-6.3	2.88E-10	7.15E-08
LTBP2	381.0	-1.4	0.2	-6.0	1.95E-09	3.77E-07
MYO15B	413.0	-1.4	0.3	-5.0	5.03E-07	4.69E-05
HMGA2	5886.6	-1.4	0.3	-4.9	8.92E-07	7.79E-05
ANTXR2	1309.6	-1.4	0.4	-4.0	6.32E-05	2.29E-03
LOXL4	147.0	-1.4	0.3	-4.1	4.83E-05	1.85E-03
LOXL2	6549.2	-1.4	0.3	-4.6	5.34E-06	3.43E-04
MAP3K12	342.6	-1.4	0.3	-4.3	1.75E-05	8.38E-04
TMCO4	279.7	-1.4	0.4	-3.7	2.57E-04	6.49E-03
CCDC136	47.1	-1.4	0.5	-3.1	2.22E-03	3.36E-02
FOXQ1	1055.5	-1.4	0.3	-4.3	1.87E-05	8.76E-04
HOXA1	387.6	-1.4	0.2	-7.0	2.32E-12	9.09E-10
NIPAL2	421.3	-1.4	0.5	-3.0	3.03E-03	4.18E-02
PTP4A3	125.4	-1.4	0.4	-3.6	3.41E-04	8.04E-03
REEP6	1697.2	-1.4	0.4	-3.8	1.75E-04	4.96E-03
SPOCD1	155.5	-1.3	0.4	-3.3	1.08E-03	1.93E-02
NAV3	788.3	-1.3	0.4	-3.1	2.25E-03	3.38E-02
MECOM	451.2	-1.3	0.4	-3.8	1.51E-04	4.45E-03
ASPH	40806.3	-1.3	0.3	-4.2	2.93E-05	1.26E-03
LIFR	749.6	-1.3	0.3	-5.2	2.26E-07	2.41E-05
DENND6A	296.7	-1.3	0.2	-5.6	2.81E-08	3.74E-06
DAB2	262.8	-1.3	0.5	-2.9	3.44E-03	4.55E-02
APOBEC3C	1598.1	-1.3	0.3	-3.8	1.25E-04	3.84E-03
CNOT11	1669.1	-1.3	0.3	-4.4	1.00E-05	5.52E-04
RGL1	86.4	-1.3	0.3	-4.5	6.78E-06	4.07E-04
SPRED3	141.3	-1.3	0.3	-4.5	6.53E-06	4.04E-04
RASSF6	53.9	-1.3	0.4	-3.4	7.46E-04	1.45E-02
RAB40B	113.5	-1.3	0.3	-4.6	4.70E-06	3.10E-04
NRGN	89.3	-1.3	0.3	-4.1	4.76E-05	1.83E-03
HMGA1	61774.4	-1.3	0.2	-6.3	3.23E-10	7.72E-08
SLC16A5	3343.8	-1.3	0.2	-5.7	9.57E-09	1.45E-06
LDLRAP1	2247.6	-1.3	0.1	-8.9	4.70E-19	6.07E-16
EPOR	263.4	-1.3	0.3	-3.8	1.32E-04	3.99E-03
PIGB	324.0	-1.3	0.3	-4.1	4.69E-05	1.82E-03
GPAT3	1679.0	-1.3	0.2	-5.7	1.12E-08	1.69E-06
FBLIM1	2294.6	-1.3	0.1	-9.2	3.57E-20	6.59E-17
EHF	178.1	-1.3	0.3	-3.9	1.14E-04	3.62E-03
GNG12	2812.8	-1.3	0.4	-3.5	4.22E-04	9.36E-03
SH3BGRL3	6591.0	-1.3	0.2	-7.6	3.85E-14	1.84E-11
TTC22	381.1	-1.3	0.3	-3.7	1.93E-04	5.35E-03
NLGN2	1308.6	-1.2	0.2	-6.6	4.06E-11	1.28E-08
CAV1	11348.9	-1.2	0.4	-2.9	3.41E-03	4.51E-02

NPAS2	1684.6	-1.2	0.2	-5.8	8.02E-09	1.26E-06
ADORA1	239.9	-1.2	0.2	-5.2	1.70E-07	1.91E-05
C1QL1	110.6	-1.2	0.4	-3.0	2.97E-03	4.12E-02
HR	2642.5	-1.2	0.4	-2.9	3.54E-03	4.64E-02
SHISA4	368.5	-1.2	0.3	-4.2	2.19E-05	1.01E-03
CD59	11797.9	-1.2	0.1	-8.8	1.07E-18	1.26E-15
LGALS9	211.0	-1.2	0.3	-3.7	2.33E-04	6.02E-03
FAM20C	2050.4	-1.2	0.2	-5.6	2.40E-08	3.37E-06
VSIR	1471.3	-1.2	0.2	-5.7	1.29E-08	1.90E-06
FAM171B	55.2	-1.2	0.3	-3.6	3.31E-04	7.87E-03
ALPP	2644.0	-1.2	0.3	-3.8	1.48E-04	4.39E-03
KRT5	829.5	-1.2	0.2	-5.0	4.59E-07	4.43E-05
VSIG10	1236.9	-1.2	0.2	-6.4	1.98E-10	5.15E-08
GRAMD1B	5215.8	-1.2	0.2	-6.0	2.26E-09	4.23E-07
HIVEP3	637.2	-1.2	0.3	-4.5	7.79E-06	4.58E-04
ARHGAP31	610.5	-1.2	0.2	-5.0	5.57E-07	5.10E-05
ATP6V1B1	84.4	-1.2	0.3	-4.0	5.42E-05	2.03E-03
LRRFIP2	2272.1	-1.2	0.3	-3.9	1.15E-04	3.63E-03
IL22RA1	120.3	-1.2	0.3	-4.1	4.27E-05	1.70E-03
ICA1L	55.3	-1.2	0.4	-3.2	1.43E-03	2.40E-02
KIF3A	528.5	-1.2	0.3	-3.9	1.10E-04	3.51E-03
IDNK	43.2	-1.2	0.4	-3.1	1.87E-03	2.96E-02
KDM1A	17413.7	-1.2	0.4	-2.9	3.31E-03	4.44E-02
TSPAN10	298.7	-1.2	0.3	-3.8	1.35E-04	4.08E-03
IDH2	437.6	-1.2	0.3	-3.5	4.46E-04	9.62E-03
ZNF487	66.3	-1.2	0.4	-3.0	2.30E-03	3.44E-02
MEGF6	2004.3	-1.2	0.2	-5.3	1.43E-07	1.65E-05
TNFSF14	82.2	-1.2	0.3	-3.6	2.80E-04	6.92E-03
CYP4V2	251.0	-1.2	0.3	-3.9	9.13E-05	3.03E-03
ARHGAP18	598.6	-1.2	0.2	-4.8	1.85E-06	1.46E-04
CDC42	4667.2	-1.2	0.3	-3.9	8.07E-05	2.77E-03
IFT46	133.8	-1.2	0.2	-4.8	1.56E-06	1.25E-04
LHX2	185.1	-1.2	0.3	-3.7	1.98E-04	5.42E-03
EML6	378.6	-1.1	0.2	-4.7	2.27E-06	1.74E-04
KRT19	15803.3	-1.1	0.3	-4.0	5.33E-05	2.01E-03
SECISBP2L	1200.3	-1.1	0.3	-3.4	6.39E-04	1.29E-02
SLC25A45	260.0	-1.1	0.2	-4.7	2.88E-06	2.08E-04
OAF	4021.1	-1.1	0.3	-3.7	2.20E-04	5.80E-03
LDAH	300.6	-1.1	0.3	-3.8	1.41E-04	4.23E-03
FTH1	210683.0	-1.1	0.2	-5.6	2.59E-08	3.53E-06
WWTR1	2594.7	-1.1	0.2	-4.7	3.27E-06	2.32E-04
SMPDL3B	48.7	-1.1	0.4	-3.2	1.37E-03	2.31E-02
SEMA4F	289.7	-1.1	0.3	-3.6	3.43E-04	8.08E-03
DAGLA	331.5	-1.1	0.3	-4.5	8.14E-06	4.72E-04
CDH13	1193.9	-1.1	0.3	-3.3	8.55E-04	1.61E-02
RNF182	232.3	-1.1	0.3	-3.5	4.51E-04	9.69E-03

ACTN4	53045.5	-1.1	0.3	-3.4	6.95E-04	1.38E-02
HOXA5	149.8	-1.1	0.3	-4.1	4.32E-05	1.72E-03
MYO5C	257.2	-1.1	0.2	-4.9	8.15E-07	7.20E-05
SNRNP27	593.3	-1.1	0.3	-3.2	1.20E-03	2.09E-02
WDR97	71.5	-1.1	0.3	-3.4	6.14E-04	1.25E-02
UBASH3B	756.4	-1.1	0.2	-6.6	3.67E-11	1.19E-08
PRXL2A	1229.4	-1.1	0.4	-3.0	2.45E-03	3.61E-02
NEK3	439.5	-1.1	0.3	-4.3	1.79E-05	8.51E-04
KRT80	9991.5	-1.1	0.1	-7.4	1.70E-13	7.56E-11
POPDC3	442.5	-1.1	0.2	-6.0	2.34E-09	4.31E-07
CDCP1	21695.2	-1.1	0.4	-3.0	3.12E-03	4.27E-02
FAM117A	89.4	-1.1	0.3	-3.1	1.65E-03	2.68E-02
DMTN	639.9	-1.1	0.3	-3.6	3.29E-04	7.85E-03
KLF12	702.4	-1.1	0.3	-4.1	3.90E-05	1.58E-03
EVC	374.5	-1.1	0.3	-3.7	2.54E-04	6.44E-03
ABCC3	8833.5	-1.1	0.3	-3.6	3.79E-04	8.75E-03
CAVIN2	417.2	-1.1	0.3	-3.1	1.82E-03	2.89E-02
ITGA3	45105.6	-1.1	0.2	-4.9	8.02E-07	7.16E-05
SNPH	155.6	-1.1	0.3	-3.2	1.25E-03	2.16E-02
ECM1	1725.4	-1.1	0.2	-7.0	3.52E-12	1.28E-09
TRIM62	319.2	-1.1	0.3	-3.6	3.72E-04	8.60E-03
FUT8	2519.2	-1.1	0.2	-6.5	9.60E-11	2.76E-08
PARVA	2144.9	-1.0	0.3	-3.6	3.18E-04	7.71E-03
CABLES2	595.8	-1.0	0.2	-4.3	1.77E-05	8.44E-04
TPMT	1095.7	-1.0	0.3	-3.7	2.58E-04	6.49E-03
NABP1	1289.5	-1.0	0.3	-3.5	4.21E-04	9.36E-03
NRP1	1761.7	-1.0	0.3	-3.6	3.82E-04	8.76E-03
AIF1L	573.0	-1.0	0.3	-3.6	3.32E-04	7.88E-03
SCD5	226.9	-1.0	0.3	-3.5	4.51E-04	9.69E-03
FAM126A	877.0	-1.0	0.3	-3.1	1.90E-03	3.00E-02
GPR137B	248.6	-1.0	0.3	-3.1	1.76E-03	2.82E-02
SOX7	3004.2	-1.0	0.2	-4.4	1.26E-05	6.50E-04
CCDC115	563.1	-1.0	0.2	-4.4	1.10E-05	5.88E-04
RIN1	1929.7	-1.0	0.2	-4.1	4.12E-05	1.65E-03
ADGRB2	1189.1	-1.0	0.2	-4.5	5.95E-06	3.73E-04
DKK1	6337.7	-1.0	0.2	-6.2	7.14E-10	1.62E-07
ZFYVE28	90.3	-1.0	0.3	-3.4	6.51E-04	1.31E-02
SOS2	552.9	-1.0	0.2	-4.3	1.93E-05	8.95E-04
ARL8B	1721.6	-1.0	0.2	-4.4	9.34E-06	5.28E-04
TSPAN5	357.1	-1.0	0.3	-3.0	2.96E-03	4.12E-02
ST6GALNAC1	413.5	-1.0	0.2	-4.3	1.54E-05	7.52E-04

Table S13. Mass spectrometry data for VAMP8 binding proteins in S26 cell line.

Protein Group	Protein ID	Accession	-10lgP	Coverage (%)	Area S26	#Peptides	#Unique	#Spec S26	Avg. Mass	Description
18	16	Q15149|PLEC_HUMAN	316.32	20	1.03E+08	83	76	96	531796	Plectin OS=Homo sapiens OX=9606 GN=PLEC PE=1 SV=3
20	11	Q6ZRV2|FA83H_HUMAN	307.55	56	2.65E+08	60	59	92	127122	Protein FAM83H OS=Homo sapiens OX=9606 GN=FAM83H PE=1 SV=3
10	29	P15924|DESP_HUMAN	297.69	33	2.13E+08	99	95	126	331774	Desmoplakin OS=Homo sapiens OX=9606 GN=DSP PE=1 SV=3
45	27	Q08211|DHX9_HUMAN	282.38	38	1.03E+08	43	43	57	140958	ATP-dependent RNA helicase A OS=Homo sapiens OX=9606 GN=DHX9 PE=1 SV=4
25	26	Q8WWM7|ATX2L_HUMAN	271.63	46	2.54E+08	44	43	75	113374	Ataxin-2-like protein OS=Homo sapiens OX=9606 GN=ATXN2L PE=1 SV=2
50	24	P14923|PLAK_HUMAN	255.01	45	9.43E+07	31	31	47	81745	Junction plakoglobin OS=Homo sapiens OX=9606 GN=JUP PE=1 SV=3
48	30	Q9Y2W1|TR150_HUMAN	252.83	32	1.46E+08	32	31	51	108666	Thyroid hormone receptor-associated protein 3 OS=Homo sapiens OX=9606 GN=THRAP3 PE=1 SV=2

39	23	Q9NYF8|BCLF1_HUMAN	250.59	37	2.13E+08	33	31	60	106122	Bcl-2-associated transcription factor 1 OS=Homo sapiens OX=9606 GN=BCLAF1 PE=1 SV=2
56	25	Q02413|DSG1_HUMAN	238.44	30	1.17E+08	25	24	37	113748	Desmoglein-1 OS=Homo sapiens OX=9606 GN=DSG1 PE=1 SV=2
55	32	Q8WWI1|LMO7_HUMAN	235.99	25	5.75E+07	35	35	39	192694	LIM domain only protein 7 OS=Homo sapiens OX=9606 GN=LMO7 PE=1 SV=3
58	49	Q15424|SAFB1_HUMAN	233.13	27	2.99E+07	26	14	31	102642	Scaffold attachment factor B1 OS=Homo sapiens OX=9606 GN=SAFB PE=1 SV=4
60	39	Q6PKG0|LARP1_HUMAN	230.73	33	3.04E+07	29	23	35	123510	La-related protein 1 OS=Homo sapiens OX=9606 GN=LARP1 PE=1 SV=2
75	31	Q9UPN4|CP131_HUMAN	227.14	24	1.61E+07	22	21	24	122149	Centrosomal protein of 131 kDa OS=Homo sapiens OX=9606 GN=CEP131 PE=1 SV=3
53	50	P58107|EPIPL_HUMAN	227.14	8	2.40E+07	32	26	38	555666	Epiplakin OS=Homo sapiens OX=9606 GN=EPPK1 PE=1 SV=3
70	67	Q14151|SAFB2_HUMAN	217.85	22	1.11E+07	21	9	27	107474	Scaffold attachment factor B2 OS=Homo sapiens OX=9606 GN=SAFB2

										PE=1 SV=1
85	77	P60709|ACTB_HUMAN	205.24	46	3.03E+07	14	5	20	41737	Actin cytoplasmic 1 OS=Homo sapiens OX=9606 GN=ACTB PE=1 SV=1
80	63	P07355|ANXA2_HUMAN	202.99	45	2.84E+07	18	17	23	38604	Annexin A2 OS=Homo sapiens OX=9606 GN=ANXA2 PE=1 SV=2
74	149	Q969G5|CAVN3_HUMAN	199.05	35	1.19E+08	14	14	27	27701	Caveolae-associated protein 3 OS=Homo sapiens OX=9606 GN=CAVIN3 PE=1 SV=3
73	60	Q9C0C2|TB182_HUMAN	198.92	20	2.22E+07	23	23	27	181795	182 kDa tankyrase-1-binding protein OS=Homo sapiens OX=9606 GN=TNKS1BP1 PE=1 SV=4
65	59	Q08188|TGM3_HUMAN	198.55	29	3.22E+07	22	20	31	76632	Protein-glutamine gamma-glutamyltransferase E OS=Homo sapiens OX=9606 GN=TGM3 PE=1 SV=4
79	65	P42704|LPPRC_HUMAN	195.02	16	1.63E+07	21	21	23	157904	Leucine-rich PPR motif-containing protein mitochondrial OS=Homo sapiens OX=9606 GN=LRPPRC PE=1 SV=3
82	72	P41252|SYIC_HUMAN	192.8	18	1.85E+07	21	21	23	144498	Isoleucine--tRNA ligase cytoplasmic OS=Homo sapiens OX=9606 GN=IARS1

										PE=1 SV=2
83	58	Q9NZB2|F120A_HUMAN	187.54	20	2.03E+07	18	18	23	121888	Constitutive coactivator of PPAR-gamma-like protein 1 OS=Homo sapiens OX=9606 GN=FAM120A PE=1 SV=2
64	81	Q03135|CAV1_HUMAN	185.8	74	1.94E+08	15	15	30	20472	Caveolin-1 OS=Homo sapiens OX=9606 GN=CAV1 PE=1 SV=4
84	70	Q13835|PKP1_HUMAN	182.72	23	3.76E+07	17	16	21	82861	Plakophilin-1 OS=Homo sapiens OX=9606 GN=PKP1 PE=1 SV=2
97	119	P63267|ACTH_HUMAN	180.27	26	1.06E+06	10	1	15	41877	Actin gamma-enteric smooth muscle OS=Homo sapiens OX=9606 GN=ACTG2 PE=1 SV=1
98	88	P26640|SYVC_HUMAN	175.21	14	1.08E+07	15	15	15	140476	Valine--tRNA ligase OS=Homo sapiens OX=9606 GN=VARS1 PE=1 SV=4
93	98	Q08554|DSC1_HUMAN	174.79	16	2.63E+07	11	11	16	99987	Desmocollin-1 OS=Homo sapiens OX=9606 GN=DSC1 PE=1 SV=2
78	75	O75533|SF3B1_HUMAN	174.73	18	2.05E+07	19	19	24	145830	Splicing factor 3B subunit 1 OS=Homo sapiens OX=9606 GN=SF3B1 PE=1 SV=3
115	109	P68104|EF1A1_HUMAN	171.09	24	1.61E+07	10	10	12	50141	Elongation factor 1-alpha 1 OS=Homo sapiens OX=9606 GN=EEF1A1 PE=1 SV=1

90	150	P84103|SRSF3_HUMAN	169.77	48	4.66E+07	8	8	16	19330	Serine/arginine-rich splicing factor 3 OS=Homo sapiens OX=9606 GN=SRSF3 PE=1 SV=1
92	76	Q13435|SF3B2_HUMAN	167.41	21	1.67E+07	14	12	17	100228	Splicing factor 3B subunit 2 OS=Homo sapiens OX=9606 GN=SF3B2 PE=1 SV=2
108	64	Q6WCQ1|MPRIP_HUMAN	166.35	15	1.01E+07	12	12	13	116533	Myosin phosphatase Rho-interacting protein OS=Homo sapiens OX=9606 GN=MPRIP PE=1 SV=3
100	114	Q5T749|KPRP_HUMAN	165.7	20	2.80E+07	9	9	13	64136	Keratinocyte proline-rich protein OS=Homo sapiens OX=9606 GN=KPRP PE=1 SV=1
87	84	Q92614|MY18A_HUMAN	165.37	9	1.31E+07	14	14	16	233113	Unconventional myosin-XVIIIa OS=Homo sapiens OX=9606 GN=MYO18A PE=1 SV=3
116	152	P31944|CASPE_HUMAN	164.48	36	1.34E+07	10	10	11	27680	Caspase-14 OS=Homo sapiens OX=9606 GN=CASP14 PE=1 SV=2
112	96	P29508|SPB3_HUMAN	164.36	31	6.96E+06	12	5	13	44565	Serpin B3 OS=Homo sapiens OX=9606 GN=SERPINB3 PE=1 SV=2
103	115	P02768|ALBU_HUMAN	162.59	16	7.07E+06	11	6	14	69367	Albumin OS=Homo sapiens OX=9606 GN=ALB PE=1

										SV=2
96	97	P22735|TGM1_HUMAN	159.01	22	1.46E+07	16	16	16	89787	Protein-glutamine gamma-glutamyltransferase K OS=Homo sapiens OX=9606 GN=TGM1 PE=1 SV=4
91	85	P04899|GNAI2_HUMAN	156.69	40	1.31E+07	14	8	18	40451	Guanine nucleotide-binding protein G(i) subunit alpha-2 OS=Homo sapiens OX=9606 GN=GNAI2 PE=1 SV=3
95	127	P51991|ROA3_HUMAN	156.62	29	1.47E+07	10	8	15	39595	Heterogeneous nuclear ribonucleoprotein A3 OS=Homo sapiens OX=9606 GN=HNRNPA3 PE=1 SV=2
109	95	Q86SQ0|PHLB2_HUMAN	154.42	10	8.98E+06	12	12	12	142158	Pleckstrin homology-like domain family B member 2 OS=Homo sapiens OX=9606 GN=PHLDB2 PE=1 SV=2
101	130	P04406|G3P_HUMAN	153.72	27	1.87E+07	9	8	15	36053	Glyceraldehyde-3-phosphate dehydrogenase OS=Homo sapiens OX=9606 GN=GAPDH PE=1 SV=3
110	144	O75494|SRS10_HUMAN	153.66	34	1.88E+07	10	10	12	31301	Serine/arginine-rich splicing factor 10 OS=Homo sapiens OX=9606 GN=SRSF10 PE=1 SV=1
67	108	P46781|RS9_HUMAN	153.33	48	8.14E+07	16	15	27	22591	40S ribosomal protein S9 OS=Homo

										sapiens OX=9606 GN=RPS9 PE=1 SV=3
89	182	P51636|CAV2_HUMAN	152.57	56	1.19E+08	7	7	17	18291	Caveolin-2 OS=Homo sapiens OX=9606 GN=CAV2 PE=1 SV=2
118	154	P47929|LEG7_HUMAN	152.4	69	2.17E+07	9	9	10	15075	Galectin-7 OS=Homo sapiens OX=9606 GN=LGALS7 PE=1 SV=2
105	105	P68363|TBA1B_HUMAN	151.69	33	2.00E+07	11	11	14	50152	Tubulin alpha-1B chain OS=Homo sapiens OX=9606 GN=TUBA1B PE=1 SV=1
111	103	P08754|GNAI3_HUMAN	151.1	31	7.79E+06	11	5	13	40532	Guanine nucleotide-binding protein G(i) subunit alpha-3 OS=Homo sapiens OX=9606 GN=GNAI3 PE=1 SV=3
94	180	P18621|RL17_HUMAN	150.75	36	2.90E+07	7	7	15	21397	60S ribosomal protein L17 OS=Homo sapiens OX=9606 GN=RPL17 PE=1 SV=3
120	155	P04792|HSPB1_HUMAN	149.96	52	2.03E+07	7	7	11	22783	Heat shock protein beta-1 OS=Homo sapiens OX=9606 GN=HSPB1 PE=1 SV=2
102	146	P07910|HNRPC_HUMAN	149.53	23	2.68E+07	8	8	11	33670	Heterogeneous nuclear ribonucleoproteins C1/C2 OS=Homo sapiens OX=9606 GN=HNRNPC PE=1 SV=4

153	251	P81605|DCD_HUMAN	148.96	56	2.28E+07	6	6	7	11284	Dermcidin OS=Homo sapiens OX=9606 GN=DCD PE=1 SV=2
125	126	P48594|SPB4_HUMAN	148.38	22	1.89E+06	9	2	9	44854	Serpin B4 OS=Homo sapiens OX=9606 GN=SERPINB4 PE=1 SV=2
117	187	P62081|RS7_HUMAN	148.03	35	2.55E+07	7	7	11	22127	40S ribosomal protein S7 OS=Homo sapiens OX=9606 GN=RPS7 PE=1 SV=1
107	142	Q8WVV4|POF1B_HUMAN	145.52	15	1.82E+07	9	9	13	68065	Protein POF1B OS=Homo sapiens OX=9606 GN=POF1B PE=1 SV=3
135	135	Q15365|PCBP1_HUMAN	143.64	33	1.11E+07	8	5	9	37498	Poly(rC)-binding protein 1 OS=Homo sapiens OX=9606 GN=PCBP1 PE=1 SV=2
132	221	Q9NZT1|CALL5_HUMAN	140.55	51	5.72E+07	8	8	9	15893	Calmodulin-like protein 5 OS=Homo sapiens OX=9606 GN=CALML5 PE=1 SV=2
119	250	P31151|S10A7_HUMAN	140.22	48	1.70E+07	6	6	11	11471	Protein S100-A7 OS=Homo sapiens OX=9606 GN=S100A7 PE=1 SV=4
99	162	P46778|RL21_HUMAN	139.27	40	1.90E+07	11	10	14	18565	60S ribosomal protein L21 OS=Homo sapiens OX=9606 GN=RPL21 PE=1 SV=2
104	128	Q5D862|FILA2_HUMAN	138.68	6	2.73E+07	10	8	12	248072	Filaggrin-2 OS=Homo sapiens OX=9606

										GN=FLG2 PE=1 SV=1
114	148	O15182|CETN3_HUMAN	135.95	47	1.89E+07	8	8	10	19550	Centrin-3 OS=Homo sapiens OX=9606 GN=CETN3 PE=1 SV=2
126	139	P62995|TRA2B_HUMAN	134.94	23	1.47E+07	6	5	7	33666	Transformer-2 protein homolog beta OS=Homo sapiens OX=9606 GN=TRA2B PE=1 SV=1
136	153	Q06830|PRDX1_HUMAN	133.91	35	8.38E+06	8	7	9	22110	Peroxiredoxin-1 OS=Homo sapiens OX=9606 GN=PRDX1 PE=1 SV=1
165	191	P25311|ZA2G_HUMAN	132.93	19	4.41E+06	5	5	5	34259	Zinc-alpha-2-glycoprotein OS=Homo sapiens OX=9606 GN=AZGP1 PE=1 SV=2
141	167	Q99700|ATX2_HUMAN	132.28	5	2.93E+06	4	4	5	140283	Ataxin-2 OS=Homo sapiens OX=9606 GN=ATXN2 PE=1 SV=2
148	195	P16615|AT2A2_HUMAN	130.7	6	2.95E+06	6	5	6	114757	Sarcoplasmic/endoplasmic reticulum calcium ATPase 2 OS=Homo sapiens OX=9606 GN=ATP2A2 PE=1 SV=1
159	189	Q96P63|SPB12_HUMAN	129.36	16	8.66E+06	6	5	6	46276	Serpin B12 OS=Homo sapiens OX=9606 GN=SERPINB12 PE=1 SV=1
137	164	Q7L014|DDX46_HUMAN	128.28	6	3.50E+06	7	6	8	117362	Probable ATP-dependent RNA helicase DDX46 OS=Homo sapiens OX=9606 GN=DDX46

										PE=1 SV=2
106	66	O43795|MYO1B_HUMAN	127.68	10	6.75E+06	10	7	11	131985	Unconventional myosin-Ib OS=Homo sapiens OX=9606 GN=MYO1B PE=1 SV=3
145	174	P05089|ARGI1_HUMAN	126.82	19	7.92E+06	6	6	7	34735	Arginase-1 OS=Homo sapiens OX=9606 GN=ARG1 PE=1 SV=2
124	107	P05023|AT1A1_HUMAN	124.09	10	5.31E+06	9	7	9	112896	Sodium/potassium-transporting ATPase subunit alpha-1 OS=Homo sapiens OX=9606 GN=ATP1A1 PE=1 SV=1
164	181	Q99873|ANM1_HUMAN	121.84	20	2.89E+06	6	6	6	42462	Protein arginine N-methyltransferase 1 OS=Homo sapiens OX=9606 GN=PRMT1 PE=1 SV=3
150	188	P28074|PSB5_HUMAN	121.81	26	2.70E+06	6	6	7	28480	Proteasome subunit beta type-5 OS=Homo sapiens OX=9606 GN=PSMB5 PE=1 SV=3
169	274	Q15517|CDSN_HUMAN	119.92	7	1.22E+07	3	3	5	51522	Corneodesmosin OS=Homo sapiens OX=9606 GN=CDSN PE=1 SV=3
180	208	P11142|HSP7C_HUMAN	119.11	10	9.71E+05	5	2	5	70898	Heat shock cognate 71 kDa protein OS=Homo sapiens OX=9606 GN=HSPA8 PE=1 SV=1
154	211	Q9Y5S9|RBM8A_HUMAN	117.8	31	1.15E+07	4	4	6	19889	RNA-binding protein 8A OS=Homo

										sapiens OX=9606 GN=RBM8A PE=1 SV=1
162	264	P06702|S10A9_HUMAN	117.38	44	7.73E+06	4	4	5	13242	Protein S100-A9 OS=Homo sapiens OX=9606 GN=S100A9 PE=1 SV=1
151	201	O75400|PR40A_HUMAN	115.19	6	4.05E+06	6	6	6	108805	Pre-mRNA-processing factor 40 homolog A OS=Homo sapiens OX=9606 GN=PRPF40A PE=1 SV=2
152	218	Q9Y446|PKP3_HUMAN	113.05	7	3.76E+06	5	5	5	87082	Plakophilin-3 OS=Homo sapiens OX=9606 GN=PKP3 PE=1 SV=1
140	118	Q6IQ23|PKHA7_HUMAN	111.82	6	3.54E+06	7	7	7	127135	Pleckstrin homology domain-containing family A member 7 OS=Homo sapiens OX=9606 GN=PLEKHA7 PE=1 SV=2
127	168	P62987|RL40_HUMAN	111.81	46	2.90E+07	6	6	10	14728	Ubiquitin-60S ribosomal protein L40 OS=Homo sapiens OX=9606 GN=UBA52 PE=1 SV=2
146	175	P07437|TBB5_HUMAN	111.45	17	2.52E+06	6	3	7	49671	Tubulin beta chain OS=Homo sapiens OX=9606 GN=TUBB PE=1 SV=2
185	254	P0DP24|CALM2_HUMAN	110.54	40	8.22E+06	4	4	5	16838	Calmodulin-2 OS=Homo sapiens OX=9606 GN=CALM2 PE=1 SV=1
175	233	P04040|CATA_HU	110.0	9	1.33E	3	3	3	5975	Catalase

		MAN	5		+06				6	OS=Homo sapiens OX=9606 GN=CAT PE=1 SV=3
128	173	P02545|LMNA_HUMAN	106.23	8	2.95E+06	7	5	8	74140	Prelamin-A/C OS=Homo sapiens OX=9606 GN=LMNA PE=1 SV=1
133	220	Q9UN81|LORF1_HUMAN	106.19	13	5.08E+06	4	4	7	40056	LINE-1 retrotransposable element ORF1 protein OS=Homo sapiens OX=9606 GN=L1RE1 PE=1 SV=1
149	184	Q15366|PCBP2_HUMAN	104.43	21	4.72E+06	6	3	7	38580	Poly(rC)-binding protein 2 OS=Homo sapiens OX=9606 GN=PCBP2 PE=1 SV=1
189	222	P32969|RL9_HUMAN	103.95	32	8.54E+06	5	5	5	21863	60S ribosomal protein L9 OS=Homo sapiens OX=9606 GN=RPL9 PE=1 SV=1
218	271	P63104|1433Z_HUMAN	103.7	14	1.88E+06	3	2	3	27745	14-3-3 protein zeta/delta OS=Homo sapiens OX=9606 GN=YWHAZ PE=1 SV=1
183	227	P04075|ALDOA_HUMAN	103.16	13	3.40E+06	5	5	5	39420	Fructose-bisphosphate aldolase A OS=Homo sapiens OX=9606 GN=ALDOA PE=1 SV=2
232	263	P06748|NPM_HUMAN	102.51	23	5.91E+06	4	4	4	32575	Nucleophosmin OS=Homo sapiens OX=9606 GN=NPM1 PE=1 SV=2
181	212	P07339|CATD_HU	101.4	13	4.81E	5	5	5	4455	Cathepsin D

		MAN	3		+06				2	OS=Homo sapiens OX=9606 GN=CTSD PE=1 SV=1
182	228	O75223|GGCT_HUMAN	101.27	23	4.80E+06	4	4	4	21008	Gamma-glutamylcyclotransferase OS=Homo sapiens OX=9606 GN=GGCT PE=1 SV=1
167	214	P20930|FILA_HUMAN	101.19	2	3.37E+06	5	4	5	435180	Filaggrin OS=Homo sapiens OX=9606 GN=FLG PE=1 SV=3
192	294	P01040|CYTA_HUMAN	100.31	45	6.24E+06	4	4	5	11006	Cystatin-A OS=Homo sapiens OX=9606 GN=CSTA PE=1 SV=1
184	241	Q14574|DSC3_HUMAN	99.47	7	5.36E+06	4	4	4	99969	Desmocollin-3 OS=Homo sapiens OX=9606 GN=DSC3 PE=1 SV=3
147	186	Q9BQG0|MBB1A_HUMAN	98.24	6	3.18E+06	7	7	7	148854	Myb-binding protein 1A OS=Homo sapiens OX=9606 GN=MYBBP1A PE=1 SV=2
231	261	Q9HCY8|S10AE_HUMAN	96.24	40	4.46E+06	4	3	4	11662	Protein S100-A14 OS=Homo sapiens OX=9606 GN=S100A14 PE=1 SV=1
176	257	P13639|EF2_HUMAN	95.34	5	2.05E+06	5	4	5	95338	Elongation factor 2 OS=Homo sapiens OX=9606 GN=EEF2 PE=1 SV=4
160	210	P68371|TBB4B_HUMAN	94.21	14	1.07E+06	5	2	6	49831	Tubulin beta-4B chain OS=Homo sapiens OX=9606 GN=TUBB4B

										PE=1 SV=1
144	145	Q00059|TFAM_HUMAN	93.22	24	5.68E+06	7	6	7	29097	Transcription factor A mitochondrial OS=Homo sapiens OX=9606 GN=TFAM PE=1 SV=1
156	190	Q9UKM9|RALY_HUMAN	92.37	14	3.67E+06	5	5	6	32463	RNA-binding protein Raly OS=Homo sapiens OX=9606 GN=RALY PE=1 SV=1
217	267	P0DMV8|HS71A_HUMAN	92.27	8	8.43E+05	4	2	4	70052	Heat shock 70 kDa protein 1A OS=Homo sapiens OX=9606 GN=HSPA1A PE=1 SV=1
274	305	P10412|H14_HUMAN	91.81	15	7.46E+05	3	2	3	21865	Histone H1.4 OS=Homo sapiens OX=9606 GN=H1-4 PE=1 SV=2
129	209	P01857|IGHG1_HUMAN	88.89	20	2.47E+07	5	5	7	36106	Immunoglobulin heavy constant gamma 1 OS=Homo sapiens OX=9606 GN=IGHG1 PE=1 SV=1
190	231	P11021|BIP_HUMAN	88.52	8	1.37E+06	4	2	4	72333	Endoplasmic reticulum chaperone BiP OS=Homo sapiens OX=9606 GN=HSPA5 PE=1 SV=2
158	287	P31947|1433S_HUMAN	88.47	16	2.46E+06	4	2	4	27774	14-3-3 protein sigma OS=Homo sapiens OX=9606 GN=SFN PE=1 SV=1
299	308	P31949|S10AB_HUMAN	87.53	24	3.14E+06	3	3	3	11740	Protein S100-A11 OS=Homo sapiens OX=9606

										GN=S100A11 PE=1 SV=2
306	343	P32119|PRDX2_HUMAN	87.15	11	5.16E+05	2	1	2	21892	Peroxiredoxin-2 OS=Homo sapiens OX=9606 GN=PRDX2 PE=1 SV=5
191	249	O75477|ERLN1_HUMAN	87.03	13	3.94E+06	4	4	4	39171	Erlin-1 OS=Homo sapiens OX=9606 GN=ERLIN1 PE=1 SV=2
170	286	Q13867|BLMH_HUMAN	86.43	7	9.14E+06	4	4	4	52562	Bleomycin hydrolase OS=Homo sapiens OX=9606 GN=BLMH PE=1 SV=1
161	216	Q14134|TRI29_HUMAN	86.28	9	3.83E+06	5	5	6	65835	Tripartite motif-containing protein 29 OS=Homo sapiens OX=9606 GN=TRIM29 PE=1 SV=2
166	229	Q09666|AHNK_HUMAN	86	1	3.73E+06	5	5	5	629114	Neuroblast differentiation-associated protein AHNAK OS=Homo sapiens OX=9606 GN=AHNAK PE=1 SV=2
142	266	Q13045|FLII_HUMAN	85.49	3	9.14E+05	5	4	7	144751	Protein flightless-1 homolog OS=Homo sapiens OX=9606 GN=FLII PE=1 SV=2
262	346	Q92817|EVPL_HUMAN	84.66	1	9.52E+05	3	2	3	231602	Envoplakin OS=Homo sapiens OX=9606 GN=EVPL PE=1 SV=3
301	314	P40938|RFC3_HUMAN	83.72	10	1.17E+06	3	3	3	40556	Replication factor C subunit 3 OS=Homo sapiens

										OX=9606 GN=RFC3 PE=1 SV=2
134	245	P08670|VIME_HUMAN	82.8	8	7.22E+05	5	1	7	53652	Vimentin OS=Homo sapiens OX=9606 GN=VIM PE=1 SV=4
212	235	O75342|LX12B_HUMAN	81.84	6	2.19E+06	4	4	4	80356	Arachidonate 12-lipoxygenase 12R-type OS=Homo sapiens OX=9606 GN=ALOX12B PE=1 SV=1
273	258	Q96J01|THOC3_HUMAN	81.69	12	2.43E+06	3	3	3	38772	THO complex subunit 3 OS=Homo sapiens OX=9606 GN=THOC3 PE=1 SV=1
233	277	Q8IXT5|RB12B_HUMAN	80.36	3	1.11E+06	4	3	4	118103	RNA-binding protein 12B OS=Homo sapiens OX=9606 GN=RBM12B PE=1 SV=2
390	415	Q13247|SRSF6_HUMAN	79.62	6	1.46E+06	2	2	2	39587	Serine/arginine-rich splicing factor 6 OS=Homo sapiens OX=9606 GN=SRSF6 PE=1 SV=2
236	337	O75694|NU155_HUMAN	79.6	2	1.29E+06	3	2	3	155199	Nuclear pore complex protein Nup155 OS=Homo sapiens OX=9606 GN=NUP155 PE=1 SV=1
296	183	P51149|RAB7A_HUMAN	79.34	18	2.83E+06	3	3	3	23490	Ras-related protein Rab-7a OS=Homo sapiens OX=9606 GN=RAB7A PE=1 SV=1
310	417	Q9C0B9|ZCHC2_HUMAN	79.33	2	1.74E+06	2	2	3	125936	Zinc finger CCHC domain-

										containing protein 2 OS=Homo sapiens OX=9606 GN=ZCCHC2 PE=1 SV=6
298	291	P10599|THIO_HUMAN	79.09	30	6.62E+06	3	3	3	11737	Thioredoxin OS=Homo sapiens OX=9606 GN=TXN PE=1 SV=3
157	344	O60437|PEPL_HUMAN	76.7	2	5.26E+06	4	2	5	204745	Periplakin OS=Homo sapiens OX=9606 GN=PPL PE=1 SV=4
234	285	Q9Y3F4|STRAP_HUMAN	76.51	12	1.84E+06	3	3	3	38438	Serine-threonine kinase receptor-associated protein OS=Homo sapiens OX=9606 GN=STRAP PE=1 SV=1
276	288	P00338|LDHA_HUMAN	76.47	8	1.72E+06	3	3	3	36689	L-lactate dehydrogenase A chain OS=Homo sapiens OX=9606 GN=LDHA PE=1 SV=2
302	322	A8K2U0|A2ML1_HUMAN	76.38	3	1.94E+06	3	3	3	161106	Alpha-2-macroglobulin-like protein 1 OS=Homo sapiens OX=9606 GN=A2ML1 PE=1 SV=3
168	237	P14618|KPYM_HUMAN	76.37	9	4.02E+06	4	4	4	57937	Pyruvate kinase PKM OS=Homo sapiens OX=9606 GN=PKM PE=1 SV=4
297	301	P50213|IDH3A_HUMAN	75.06	9	2.35E+06	3	3	3	39592	Isocitrate dehydrogenase [NAD] subunit alpha mitochondrial OS=Homo

										sapiens OX=9606 GN=IDH3A PE=1 SV=1
312	427	P05109|S10A8_HUMAN	73.78	19	3.13E+06	2	2	3	10835	Protein S100-A8 OS=Homo sapiens OX=9606 GN=S100A8 PE=1 SV=1
216	248	Q96QA5|GSDMA_HUMAN	73.72	10	1.83E+06	4	4	4	49365	Gasdermin-A OS=Homo sapiens OX=9606 GN=GSDMA PE=1 SV=4
453	447	Q02539|H11_HUMAN	73.32	8	7.47E+05	2	1	2	21842	Histone H1.1 OS=Homo sapiens OX=9606 GN=H1-1 PE=1 SV=3
251	307	Q14157|UBP2L_HUMAN	73.07	3	1.32E+06	3	3	3	114534	Ubiquitin-associated protein 2-like OS=Homo sapiens OX=9606 GN=UBAP2L PE=1 SV=2
260	280	P35251|RFC1_HUMAN	72.97	2	6.98E+05	2	2	2	128254	Replication factor C subunit 1 OS=Homo sapiens OX=9606 GN=RFC1 PE=1 SV=4
383	358	O96000|NDUBA_HUMAN	72.22	12	1.35E+06	2	2	2	20777	NADH dehydrogenase [ubiquinone] 1 beta subcomplex subunit 10 OS=Homo sapiens OX=9606 GN=NDUFB10 PE=1 SV=3
221	345	Q6ZVX7|FBX50_HUMAN	71.59	8	2.08E+06	2	2	4	30847	F-box only protein 50 OS=Homo sapiens OX=9606 GN=NCCRP1 PE=1 SV=1
385	377	P62491|RB11A_HUMAN	71.33	11	5.75E+05	2	2	2	24394	Ras-related protein Rab-11A

										OS=Homo sapiens OX=9606 GN=RAB11A PE=1 SV=3
386	369	P06733|ENOA_HUMAN	70.89	6	1.33E+06	2	2	2	47169	Alpha-enolase OS=Homo sapiens OX=9606 GN=ENO1 PE=1 SV=2
442	302	P61026|RAB10_HUMAN	69.88	11	4.05E+05	2	1	2	22541	Ras-related protein Rab-10 OS=Homo sapiens OX=9606 GN=RAB10 PE=1 SV=1
275	315	Q8WWC4|MAIP1_HUMAN	69.56	11	1.65E+06	3	3	3	32545	m-AAA protease-interacting protein 1 mitochondrial OS=Homo sapiens OX=9606 GN=MAIP1 PE=1 SV=1
220	281	P08865|RSSA_HUMAN	69.22	13	2.29E+06	3	3	3	32854	40S ribosomal protein SA OS=Homo sapiens OX=9606 GN=RPSA PE=1 SV=4
311	422	Q01469|FABP5_HUMAN	67.36	19	2.12E+06	3	3	3	15164	Fatty acid-binding protein 5 OS=Homo sapiens OX=9606 GN=FABP5 PE=1 SV=3
441	421	Q9UI42|CBPA4_HUMAN	67.23	4	8.68E+05	2	2	2	47351	Carboxypeptidase A4 OS=Homo sapiens OX=9606 GN=CPA4 PE=1 SV=2
465	348	Q6NZI2|CAVN1_HUMAN	66.7	5	9.22E+05	1	1	2	43476	Caveolae-associated protein 1 OS=Homo sapiens OX=9606 GN=CAVIN1 PE=1 SV=1

300	312	Q02543|RL18A_HUMAN	66.67	17	1.51E+06	3	3	3	20762	60S ribosomal protein L18a OS=Homo sapiens OX=9606 GN=RPL18A PE=1 SV=2
384	349	Q13595|TRA2A_HUMAN	65.7	7	3.56E+05	2	1	2	32689	Transformer-2 protein homolog alpha OS=Homo sapiens OX=9606 GN=TRA2A PE=1 SV=1
235	325	P05141|ADT2_HUMAN	65.4	10	2.21E+06	3	3	4	32852	ADP/ATP translocase 2 OS=Homo sapiens OX=9606 GN=SLC25A5 PE=1 SV=7
444	429	Q15185|TEBP_HUMAN	65.23	19	2.59E+06	2	2	2	18697	Prostaglandin E synthase 3 OS=Homo sapiens OX=9606 GN=PTGES3 PE=1 SV=1
307	347	P36952|SPB5_HUMAN	64.65	5	9.96E+05	2	2	2	42100	Serpin B5 OS=Homo sapiens OX=9606 GN=SERPINB5 PE=1 SV=2
641	368	Q58FF7|H90B3_HUMAN	64.51	2	1.27E+06	1	1	1	68325	Putative heat shock protein HSP 90-beta-3 OS=Homo sapiens OX=9606 GN=HSP90AB3P PE=5 SV=1
219	283	Q9H5Q4|TFB2M_HUMAN	64.05	9	1.46E+06	3	3	3	45349	Dimethyladenosine transferase 2 mitochondrial OS=Homo sapiens OX=9606 GN=TFB2M PE=1 SV=1
439	419	Q96FQ6|S10AG_HUMAN	63.92	22	1.57E+06	2	2	2	11801	Protein S100-A16 OS=Homo sapiens OX=9606 GN=S100A16 PE=1 SV=1

449	437	P00403|COX2_HUMAN	63.79	7	8.51E+05	2	2	2	25565	Cytochrome c oxidase subunit 2 OS=Homo sapiens OX=9606 GN=MT-CO2 PE=1 SV=1
450	434	P29692|EF1D_HUMAN	63.75	13	1.66E+06	2	2	2	31122	Elongation factor 1-delta OS=Homo sapiens OX=9606 GN=EEF1D PE=1 SV=5
303	321	Q8WUY1|THEM6_HUMAN	63.5	12	1.44E+06	3	3	3	23865	Protein THEM6 OS=Homo sapiens OX=9606 GN=THEM6 PE=1 SV=2
277	376	Q15070|OXA1L_HUMAN	63.15	4	1.01E+06	2	2	2	48548	Mitochondrial inner membrane protein OXA1L OS=Homo sapiens OX=9606 GN=OXA1L PE=1 SV=3
440	420	P62805|H4_HUMAN	63.04	20	1.23E+06	2	2	2	11367	Histone H4 OS=Homo sapiens OX=9606 GN=H4C1 PE=1 SV=2
650	558	P46776|RL27A_HUMAN	62.98	7	4.07E+05	1	1	1	16561	60S ribosomal protein L27a OS=Homo sapiens OX=9606 GN=RPL27A PE=1 SV=2
445	426	P47914|RL29_HUMAN	61.13	14	3.64E+06	2	2	2	17752	60S ribosomal protein L29 OS=Homo sapiens OX=9606 GN=RPL29 PE=1 SV=2
259	284	O95239|KIF4A_HUMAN	58.91	3	9.50E+05	3	3	3	139881	Chromosome-associated kinesin KIF4A OS=Homo sapiens OX=9606 GN=KIF4A PE=1 SV=3

305	156	P35237|SPB6_HUMAN	58.82	7	1.50E+06	2	2	2	42622	Serpin B6 OS=Homo sapiens OX=9606 GN=SERPINB6 PE=1 SV=3
362	320	O43684|BUB3_HUMAN	58.71	5	6.15E+05	2	2	2	37155	Mitotic checkpoint protein BUB3 OS=Homo sapiens OX=9606 GN=BUB3 PE=1 SV=1
467	565	P35321|SPR1A_HUMAN	57.04	9	2.10E+06	1	1	2	9877	Cornifin-A OS=Homo sapiens OX=9606 GN=SPRR1A PE=1 SV=2
446	428	Q9Y383|LC7L2_HUMAN	56.35	6	8.36E+05	2	2	2	46514	Putative RNA-binding protein Luc7-like 2 OS=Homo sapiens OX=9606 GN=LUC7L2 PE=1 SV=2
452	442	P35250|RFC2_HUMAN	55.49	7	9.74E+05	2	2	2	39157	Replication factor C subunit 2 OS=Homo sapiens OX=9606 GN=RFC2 PE=1 SV=3
365	166	O14974|MYPT1_HUMAN	55.21	1	5.65E+05	1	1	1	115281	Protein phosphatase 1 regulatory subunit 12A OS=Homo sapiens OX=9606 GN=PPP1R12A PE=1 SV=1
455	440	Q5T8P6|RBM26_HUMAN	55.17	2	4.32E+05	2	1	2	113597	RNA-binding protein 26 OS=Homo sapiens OX=9606 GN=RBM26 PE=1 SV=3
280	446	Q96FX8|PERP_HUMAN	53.99	12	7.00E+06	2	2	3	21386	p53 apoptosis effector related to PMP-22 OS=Homo sapiens

										OX=9606 GN=PERP PE=1 SV=1
206	328	Q15477|SKIV2_HUMAN	53.62	2	3.33E+05	3	1	3	137755	Helicase SKI2W OS=Homo sapiens OX=9606 GN=SKIV2L PE=1 SV=3
364	379	P07477|TRY1_HUMAN	53.41	6	1.01E+07	2	1	2	26558	Trypsin-1 OS=Homo sapiens OX=9606 GN=PRSS1 PE=1 SV=1
171	198	P78332|RBM6_HUMAN	53.39	2	4.84E+05	3	2	3	128644	RNA-binding protein 6 OS=Homo sapiens OX=9606 GN=RBM6 PE=1 SV=5
454	450	P23396|RS3_HUMAN	52.55	9	9.17E+05	2	2	2	26688	40S ribosomal protein S3 OS=Homo sapiens OX=9606 GN=RPS3 PE=1 SV=2
699	572	Q5T750|XP32_HUMAN	52.54	3	5.62E+06	1	1	1	26238	Skin-specific protein 32 OS=Homo sapiens OX=9606 GN=XP32 PE=1 SV=1
304	79	Q9H307|PININ_HUMAN	50.34	3	1.38E+06	2	2	2	81628	Pinin OS=Homo sapiens OX=9606 GN=PNN PE=1 SV=5
387	381	Q92928|RAB1C_HUMAN	49.97	9	8.17E+05	2	1	2	22017	Putative Ras-related protein Rab-1C OS=Homo sapiens OX=9606 GN=RAB1C PE=5 SV=2
464	393	P26038|MOES_HUMAN	49.82	2	6.77E+05	1	1	1	67820	Moesin OS=Homo sapiens OX=9606 GN=MSN PE=1 SV=3
447	234	O43896|KIF1C_HU	49.02	2	2.70E	2	1	2	1229	Kinesin-like

		MAN			+05				47	protein KIF1C OS=Homo sapiens OX=9606 GN=KIF1C PE=1 SV=3
388	436	Q9NV92|NFIP2_HUMAN	48.43	10	1.27E+06	2	2	2	36390	NEDD4 family-interacting protein 2 OS=Homo sapiens OX=9606 GN=NDFIP2 PE=1 SV=2
691	563	P27482|CALL3_HUMAN	47.76	11	8.90E+05	1	1	1	16891	Calmodulin-like protein 3 OS=Homo sapiens OX=9606 GN=CALML3 PE=1 SV=2
693	456	P62861|RS30_HUMAN	47.19	17	1.38E+06	1	1	1	6648	40S ribosomal protein S30 OS=Homo sapiens OX=9606 GN=FAU PE=1 SV=1
692	560	Q9BVC6|TM109_HUMAN	47.05	5	4.72E+05	1	1	1	26210	Transmembrane protein 109 OS=Homo sapiens OX=9606 GN=TMEM109 PE=1 SV=1
466	561	P62891|RL39_HUMAN	46.55	20	4.70E+06	1	1	2	6407	60S ribosomal protein L39 OS=Homo sapiens OX=9606 GN=RPL39 PE=1 SV=2
208	352	Q9UPN3|MACF1_HUMAN	46.48	0	1.22E+06	2	1	2	838323	Microtubule-actin cross-linking factor 1 isoforms 1/2/3/5 OS=Homo sapiens OX=9606 GN=MACF1 PE=1 SV=4
560	259	P49419|AL7A1_HUMAN	45.55	3	4.45E+05	1	1	1	58487	Alpha-aminoadipic semialdehyde dehydrogenase OS=Homo

										sapiens OX=9606 GN=ALDH7A1 PE=1 SV=5
697	530	P07384|CAN1_HUMAN	45.49	1	7.70E+05	1	1	1	81890	Calpain-1 catalytic subunit OS=Homo sapiens OX=9606 GN=CAPN1 PE=1 SV=1
694	498	P04083|ANXA1_HUMAN	45.37	3	3.55E+05	1	1	1	38714	Annexin A1 OS=Homo sapiens OX=9606 GN=ANXA1 PE=1 SV=2
222	364	Q6UWP8|SBSN_HUMAN	45.32	3	6.80E+05	1	1	3	60541	Suprabasin OS=Homo sapiens OX=9606 GN=SBSN PE=1 SV=2
695	564	P28072|PSB6_HUMAN	45.03	4	3.85E+05	1	1	1	25358	Proteasome subunit beta type-6 OS=Homo sapiens OX=9606 GN=PSMB6 PE=1 SV=4
451	441	P35268|RL22_HUMAN	44.64	19	1.07E+06	2	2	2	14787	60S ribosomal protein L22 OS=Homo sapiens OX=9606 GN=RPL22 PE=1 SV=2
696	519	Q9UBS4|DJB11_HUMAN	44.54	3	2.38E+05	1	1	1	40514	DnaJ homolog subfamily B member 11 OS=Homo sapiens OX=9606 GN=DNAJB11 PE=1 SV=1
197	402	Q03001|DYST_HUMAN	43.83	0	6.20E+06	2	1	3	860679	Dystonin OS=Homo sapiens OX=9606 GN=DST PE=1 SV=4
698	567	P60174|TPIS_HUMAN	43.45	5	3.34E+05	1	1	1	26669	Triosephosphate isomerase OS=Homo sapiens

										OX=9606 GN=TPI1 PE=1 SV=4
389	395	Q92878|RAD50_HUMAN	43.22	1	6.27E+05	1	1	1	153892	DNA repair protein RAD50 OS=Homo sapiens OX=9606 GN=RAD50 PE=1 SV=1
645	527	P60842|IF4A1_HUMAN	43.12	2	8.57E+05	1	1	1	46154	Eukaryotic initiation factor 4A-I OS=Homo sapiens OX=9606 GN=EIF4A1 PE=1 SV=1
640	473	P23490|LORI_HUMAN	41.49	3	8.55E+05	1	1	1	25761	Loricrin OS=Homo sapiens OX=9606 GN=LORICRIN PE=1 SV=2
458	71	Q92900|RENT1_HUMAN	41.43	1	3.89E+05	1	1	1	124345	Regulator of nonsense transcripts 1 OS=Homo sapiens OX=9606 GN=UPF1 PE=1 SV=2
261	330	P53621|COPA_HUMAN	41.38	1	2.96E+05	1	1	1	138345	Coatomer subunit alpha OS=Homo sapiens OX=9606 GN=COPA PE=1 SV=2
292	4293	O15230|LAMA5_HUMAN	40.63	0	2.40E+05	2	1	2	399740	Laminin subunit alpha-5 OS=Homo sapiens OX=9606 GN=LAMA5 PE=1 SV=8
320	529	Q7Z6R9|AP2D_HUMAN	40.17	2	3.30E+06	2	1	3	49578	Transcription factor AP-2-delta OS=Homo sapiens OX=9606 GN=TFAP2D PE=1 SV=1
700	521	P62917|RL8_HUMAN	40.15	4	2.02E+05	1	1	1	28025	60S ribosomal protein L8 OS=Homo sapiens

										OX=9606 GN=RPL8 PE=1 SV=2
701	578	P60900|PSA6_HUMAN	39.95	5	4.25E+05	1	1	1	27399	Proteasome subunit alpha type-6 OS=Homo sapiens OX=9606 GN=PSMA6 PE=1 SV=1
702	569	O14818|PSA7_HUMAN	39.1	4	1.44E+05	1	1	1	27887	Proteasome subunit alpha type-7 OS=Homo sapiens OX=9606 GN=PSMA7 PE=1 SV=1
703	568	Q9ULR0|ISY1_HUMAN	38.69	5	5.04E+05	1	1	1	32992	Pre-mRNA-splicing factor ISY1 homolog OS=Homo sapiens OX=9606 GN=ISY1 PE=1 SV=3
704	579	P11279|LAMP1_HUMAN	38.58	2	9.95E+05	1	1	1	44882	Lysosome-associated membrane glycoprotein 1 OS=Homo sapiens OX=9606 GN=LAMP1 PE=1 SV=3
707	600	Q13510|ASAH1_HUMAN	38.16	2	3.65E+05	1	1	1	44660	Acid ceramidase OS=Homo sapiens OX=9606 GN=ASAH1 PE=1 SV=5
345	703	Q12802|AKP13_HUMAN	38.04	0	2.65E+08	1	1	1	307550	A-kinase anchor protein 13 OS=Homo sapiens OX=9606 GN=AKAP13 PE=1 SV=2
460	463	Q9NWB6|ARGL1_HUMAN	37.29	3	5.42E+05	1	1	1	33216	Arginine and glutamate-rich protein 1 OS=Homo sapiens OX=9606 GN=ARGLU1

										PE=1 SV=1
705	596	P46783|RS10_HUMAN	37.24	8	1.01E+06	1	1	1	18898	40S ribosomal protein S10 OS=Homo sapiens OX=9606 GN=RPS10 PE=1 SV=1
457	454	Q02878|RL6_HUMAN	37.12	8	7.77E+05	2	2	2	32728	60S ribosomal protein L6 OS=Homo sapiens OX=9606 GN=RPL6 PE=1 SV=3
643	493	Q9Y5B9|SP16H_HUMAN	36.92	1	1.32E+06	1	1	1	119914	FACT complex subunit SPT16 OS=Homo sapiens OX=9606 GN=SUPT16H PE=1 SV=1
706	575	P26373|RL13_HUMAN	36.53	4	3.18E+05	1	1	1	24261	60S ribosomal protein L13 OS=Homo sapiens OX=9606 GN=RPL13 PE=1 SV=4
400	750	Q7Z406|MYH14_HUMAN	36.16	1	7.00E+05	2	1	2	227868	Myosin-14 OS=Homo sapiens OX=9606 GN=MYH14 PE=1 SV=2
315	464	Q15834|CC85B_HUMAN	35.34	5	5.11E+05	2	1	3	22091	Coiled-coil domain-containing protein 85B OS=Homo sapiens OX=9606 GN=CCDC85B PE=1 SV=2
639	598	P83731|RL24_HUMAN	35.3	5	3.01E+05	1	1	1	17779	60S ribosomal protein L24 OS=Homo sapiens OX=9606 GN=RPL24 PE=1 SV=1
708	610	P35249|RFC4_HUMAN	34.87	2	6.89E+05	1	1	1	39682	Replication factor C subunit 4 OS=Homo sapiens OX=9606

										GN=RFC4 PE=1 SV=2
709	8862	P61247|RS3A_HUMAN	34.53	6	7.04E+05	1	1	1	29945	40S ribosomal protein S3a OS=Homo sapiens OX=9606 GN=RPS3A PE=1 SV=2
644	309	Q00839|HNRPU_HUMAN	34.07	2	5.51E+05	1	1	1	90585	Heterogeneous nuclear ribonucleoprotein U OS=Homo sapiens OX=9606 GN=HNRNPU PE=1 SV=6
710	608	Q9BSD7|NTPCR_HUMAN	33.92	8	5.14E+05	1	1	1	20713	Cancer-related nucleoside-triphosphatase OS=Homo sapiens OX=9606 GN=NTPCR PE=1 SV=1
462	472	Q7L2H7|EIF3M_HUMAN	32.67	8	1.02E+06	2	2	2	42503	Eukaryotic translation initiation factor 3 subunit M OS=Homo sapiens OX=9606 GN=EIF3M PE=1 SV=1
263	661	O60293|ZC3H1_HUMAN	31.98	1	3.90E+06	1	1	2	226354	Zinc finger C3H1 domain-containing protein OS=Homo sapiens OX=9606 GN=ZFC3H1 PE=1 SV=3
711	633	P19652|A1AG2_HUMAN	31.44	7	8.14E+05	1	1	1	23603	Alpha-1-acid glycoprotein 2 OS=Homo sapiens OX=9606 GN=ORM2 PE=1 SV=2
713	635	Q9UNX3|RL26L_HUMAN	31.35	5	1.94E+05	1	1	1	17256	60S ribosomal protein L26-like 1 OS=Homo sapiens OX=9606 GN=RPL26L1

										PE=1 SV=1
649	645	O95347|SMC2_HUMAN	30.68	1	2.00E+05	1	1	1	135656	Structural maintenance of chromosomes protein 2 OS=Homo sapiens OX=9606 GN=SMC2 PE=1 SV=2
712	605	P62937|PPIA_HUMAN	30.55	5	2.67E+05	1	1	1	18012	Peptidyl-prolyl cis-trans isomerase A OS=Homo sapiens OX=9606 GN=PPIA PE=1 SV=2
646	489	Q13185|CBX3_HUMAN	30.44	8	4.28E+05	1	1	1	20811	Chromobox protein homolog 3 OS=Homo sapiens OX=9606 GN=CBX3 PE=1 SV=4
647	469	A8K8P3|SFI1_HUMAN	30.13	1	4.68E+05	1	1	1	147663	Protein SFI1 homolog OS=Homo sapiens OX=9606 GN=SFI1 PE=1 SV=2
714	642	P40926|MDHM_HUMAN	29.98	3	5.38E+05	1	1	1	35503	Malate dehydrogenase mitochondrial OS=Homo sapiens OX=9606 GN=MDH2 PE=1 SV=3
642	459	P31327|CPSM_HUMAN	29.97	1	3.76E+05	1	1	1	164939	Carbamoyl-phosphate synthase [ammonia] mitochondrial OS=Homo sapiens OX=9606 GN=CPS1 PE=1 SV=2
479	802	Q7RTR2|NLRC3_HUMAN	29.86	1	1.94E+07	1	1	1	114657	NLR family CARD domain-containing protein 3 OS=Homo sapiens

										OX=9606 GN=NLRC3 PE=1 SV=2
648	549	P13473|LAMP2_HUMAN	29.83	2	3.60E+05	1	1	1	44961	Lysosome-associated membrane glycoprotein 2 OS=Homo sapiens OX=9606 GN=LAMP2 PE=1 SV=2
715	638	P30041|PRDX6_HUMAN	29.51	4	4.00E+05	1	1	1	25035	Peroxiredoxin-6 OS=Homo sapiens OX=9606 GN=PRDX6 PE=1 SV=3
717	639	O95433|AHSA1_HUMAN	28.45	4	1.53E+06	1	1	1	38274	Activator of 90 kDa heat shock protein ATPase homolog 1 OS=Homo sapiens OX=9606 GN=AHSA1 PE=1 SV=1
721	662	O15427|MOT4_HUMAN	26.87	2	2.69E+05	1	1	1	49469	Monocarboxylate transporter 4 OS=Homo sapiens OX=9606 GN=SLC16A3 PE=1 SV=1
718	524	P0C0S5|H2AZ_HUMAN	26.52	7	2.62E+05	1	1	1	13553	Histone H2A.Z OS=Homo sapiens OX=9606 GN=H2AZ1 PE=1 SV=2
719	665	P05198|IF2A_HUMAN	26.49	3	4.59E+05	1	1	1	36112	Eukaryotic translation initiation factor 2 subunit 1 OS=Homo sapiens OX=9606 GN=EIF2S1 PE=1 SV=3
720	684	P13489|RINI_HUMAN	26.39	3	6.16E+05	1	1	1	49974	Ribonuclease inhibitor OS=Homo sapiens OX=9606 GN=RNH1 PE=1 SV=2

722	664	P62249|RS16_HUMAN	25.79	7	4.37E+05	1	1	1	16445	40S ribosomal protein S16 OS=Homo sapiens OX=9606 GN=RPS16 PE=1 SV=2
732	805	Q6K0P9|IFIX_HUMAN	25.73	1	8.40E+05	1	1	1	55065	Pyrin and HIN domain-containing protein 1 OS=Homo sapiens OX=9606 GN=PYHIN1 PE=1 SV=1
396	882	Q86YW0|PLCZ1_HUMAN	25.64	1	2.98E+07	1	1	2	70411	1-phosphatidylinositol 4 5-bisphosphate phosphodiesterase zeta-1 OS=Homo sapiens OX=9606 GN=PLCZ1 PE=1 SV=1
724	689	Q9HD33|RM47_HUMAN	25.64	3	2.15E+05	1	1	1	29450	39S ribosomal protein L47 mitochondrial OS=Homo sapiens OX=9606 GN=MRPL47 PE=1 SV=2
727	744	Q9HAR2|AGRL3_HUMAN	25.36	1	6.22E+05	1	1	1	161812	Adhesion G protein-coupled receptor L3 OS=Homo sapiens OX=9606 GN=ADGRL3 PE=1 SV=2
726	537	Q8WXE9|STON2_HUMAN	25.34	1	1.25E+06	1	1	1	101165	Stonin-2 OS=Homo sapiens OX=9606 GN=STON2 PE=1 SV=1
725	727	P61313|RL15_HUMAN	25.19	4	1.85E+05	1	1	1	24146	60S ribosomal protein L15 OS=Homo sapiens OX=9606 GN=RPL15 PE=1 SV=2

653	8869	P12814|ACTN1_HUMAN	24.37	1	2.79E+05	1	1	1	103058	Alpha-actinin-1 OS=Homo sapiens OX=9606 GN=ACTN1 PE=1 SV=2
728	818	Q8WWU5|TCP11_HUMAN	23.81	2	4.71E+05	1	1	1	56141	T-complex protein 11 homolog OS=Homo sapiens OX=9606 GN=TCP11 PE=1 SV=1
477	643	P61221|ABCE1_HUMAN	23.6	2	4.79E+07	1	1	1	67314	ATP-binding cassette sub-family E member 1 OS=Homo sapiens OX=9606 GN=ABCE1 PE=1 SV=1
651	724	Q9Y5B6|PAXB1_HUMAN	23.23	1	1.49E+04	1	1	1	104804	PAX3- and PAX7-binding protein 1 OS=Homo sapiens OX=9606 GN=PAXBP1 PE=1 SV=2
729	696	A0A0C4DH42|HV366_HUMAN	23.08	16	4.86E+05	1	1	1	12698	Immunoglobulin heavy variable 3-66 OS=Homo sapiens OX=9606 GN=IGHV3-66 PE=3 SV=1
730	8894	Q01105|SET_HUMAN	23.06	3	1.09E+06	1	1	1	33489	Protein SET OS=Homo sapiens OX=9606 GN=SET PE=1 SV=3
563	2607	Q5SNV9|CA167_HUMAN	22.83	0	0.00E+00	1	1	1	162422	Uncharacterized protein C1orf167 OS=Homo sapiens OX=9606 GN=C1orf167 PE=2 SV=2
733	445	Q9P0K7|RAI14_HUMAN	22.52	1	5.02E+06	1	1	1	110041	Ankycorbin OS=Homo sapiens OX=9606 GN=RAI14

										PE=1 SV=2
734	756	O75030|MITF_HUMAN	22.49	2	3.22E+06	1	1	1	58796	Microphthalmia-associated transcription factor OS=Homo sapiens OX=9606 GN=MITF PE=1 SV=2
735	850	P62244|RS15A_HUMAN	22.08	6	3.97E+05	1	1	1	14840	40S ribosomal protein S15a OS=Homo sapiens OX=9606 GN=RPS15A PE=1 SV=2
655	876	Q6AWC2|WWC2_HUMAN	21.82	1	3.57E+05	1	1	1	133891	Protein WWC2 OS=Homo sapiens OX=9606 GN=WWC2 PE=1 SV=2
593	8888	P38606|VATA_HUMAN	21.73	2	4.09E+05	1	1	1	68304	V-type proton ATPase catalytic subunit A OS=Homo sapiens OX=9606 GN=ATP6V1A PE=1 SV=2
652	492	P67936|TPM4_HUMAN	21.55	4	6.64E+05	1	1	1	28522	Tropomyosin alpha-4 chain OS=Homo sapiens OX=9606 GN=TPM4 PE=1 SV=3
559	418	Q6PGP7|TTC37_HUMAN	21.32	1	3.41E+05	1	1	1	175485	Tetratricopeptide repeat protein 37 OS=Homo sapiens OX=9606 GN=TTC37 PE=1 SV=1
656	550	A6NFT4|CFA73_HUMAN	21.32	4	9.24E+05	1	1	1	35914	Cilia- and flagella-associated protein 73 OS=Homo sapiens OX=9606 GN=CFAP73 PE=2 SV=3
739	8913	Q96S07|PRR25_HUMAN	21.08	2	0.00E+00	1	1	1	40999	Proline-rich protein 25 OS=Homo

										sapiens OX=9606 GN=PRR25 PE=4 SV=1
737	923	Q5SRD1|TI23B_HUMAN	21.06	9	8.03E+05	1	1	1	19670	Mitochondrial import inner membrane translocase subunit Tim23B OS=Homo sapiens OX=9606 GN=TIMM23B PE=1 SV=3
590	776	P10586|PTPRF_HUMAN	21.01	0	1.15E+06	1	1	1	212877	Receptor-type tyrosine-protein phosphatase F OS=Homo sapiens OX=9606 GN=PTPRF PE=1 SV=2
738	900	O00763|ACACB_HUMAN	20.95	1	1.35E+06	1	1	1	276539	Acetyl-CoA carboxylase 2 OS=Homo sapiens OX=9606 GN=ACACB PE=1 SV=3
402	487	Q66GS9|CP135_HUMAN	20.94	1	8.18E+06	1	1	1	133490	Centrosomal protein of 135 kDa OS=Homo sapiens OX=9606 GN=CEP135 PE=1 SV=2
741	890	P62280|RS11_HUMAN	20.74	5	2.47E+05	1	1	1	18431	40S ribosomal protein S11 OS=Homo sapiens OX=9606 GN=RPS11 PE=1 SV=3
690	1425	Q400G9|AMZ1_HUMAN	20.66	1	5.59E+05	1	1	1	54924	Archaemetzincin-1 OS=Homo sapiens OX=9606 GN=AMZ1 PE=2 SV=1
599	1019	O14647|CHD2_HUMAN	20.31	1	6.21E+05	1	1	1	211342	Chromodomain-helicase-DNA-binding protein 2 OS=Homo sapiens OX=9606 GN=CHD2

Table S14. Mass spectrometry data for VAMP8 binding proteins in 5-8F cell line.

Protein Group	Protein ID	Accession	-10lgP	Coverage (%)	Area 5-8F	#Peptides	#Unique	#Spec 5-8F	Avg. Mass	Description
2	16	Q15149|PLEC_HUMAN	258.89	14	5.57E+07	64	59	67	531796	Plectin OS=Homo sapiens OX=9606 GN=PLEC PE=1 SV=3
1	26	Q8WWM7|ATX2L_HUMAN	238.86	39	2.40E+08	42	42	81	113374	Ataxin-2-like protein OS=Homo sapiens OX=9606 GN=ATXN2L PE=1 SV=2
5	27	Q08211|DHX9_HUMAN	219.4	31	6.10E+07	40	38	45	140958	ATP-dependent RNA helicase A OS=Homo sapiens OX=9606 GN=DHX9 PE=1 SV=4
16	32	Q8WWI1|LMO7_HUMAN	200.61	16	4.40E+08	24	24	26	192694	LIM domain only protein 7 OS=Homo sapiens OX=9606 GN=LMO7 PE=1 SV=3
9	30	Q9Y2W1|TR150_HUMAN	200.37	31	1.12E+08	29	28	41	108666	Thyroid hormone receptor-associated protein 3 OS=Homo sapiens OX=9606 GN=THRAP3 PE=1 SV=2
15	66	O43795|MYO1B_HUMAN	200.32	25	3.85E+07	26	26	31	131985	Unconventional myosin-Ib OS=Homo sapiens OX=9606 GN=MYO1B PE=1 SV=3
19	71	Q92900|RENT1_HUMAN	200.1	25	3.18E+07	25	25	30	124345	Regulator of nonsense transcripts 1 OS=Homo sapiens OX=9606 GN=UPF1 PE=1 SV=2

8	23	Q9NYF8|BCLF1_HUMAN	199.27	26	1.07E+08	27	26	39	106122	Bcl-2-associated transcription factor 1 OS=Homo sapiens OX=9606 GN=BCLAF1 PE=1 SV=2
10	79	Q9H307|PININ_HUMAN	189.93	28	3.04E+07	26	26	32	81628	Pinin OS=Homo sapiens OX=9606 GN=PNN PE=1 SV=5
14	82	Q7L2E3|DHX30_HUMAN	186.11	23	2.34E+07	28	28	30	133938	ATP-dependent RNA helicase DHX30 OS=Homo sapiens OX=9606 GN=DHX30 PE=1 SV=1
33	60	Q9C0C2|TB182_HUMAN	182.71	15	1.19E+07	18	18	19	181795	182 kDa tankyrase-1-binding protein OS=Homo sapiens OX=9606 GN=TNKS1BP1 PE=1 SV=4
23	80	Q5T0W9|FA83B_HUMAN	178.85	23	1.94E+07	19	19	21	114799	Protein FAM83B OS=Homo sapiens OX=9606 GN=FAM83B PE=1 SV=1
30	11	Q6ZRV2|FA83H_HUMAN	174.27	19	1.65E+07	18	18	18	127122	Protein FAM83H OS=Homo sapiens OX=9606 GN=FAM83H PE=1 SV=3
24	31	Q9UPN4|CP131_HUMAN	173.51	19	9.64E+06	19	19	19	122149	Centrosomal protein of 131 kDa OS=Homo sapiens OX=9606 GN=CEP131 PE=1 SV=3
25	149	Q969G5|CAVN3_HUMAN	166.97	31	1.11E+08	12	12	21	27701	Caveolae-associated protein 3 OS=Homo sapiens OX=9606 GN=CAVIN3 PE=1 SV=3

38	50	P58107|EPIPL_HUMAN	160.54	3	2.91E+06	13	8	14	555666	Epiplakin OS=Homo sapiens OX=9606 GN=EPPK1 PE=1 SV=3
20	81	Q03135|CAV1_HUMAN	159.4	56	2.48E+08	13	13	28	20472	Caveolin-1 OS=Homo sapiens OX=9606 GN=CAV1 PE=1 SV=4
28	64	Q6WCQ1|MPRIP_HUMAN	157.28	17	1.14E+07	15	15	19	116533	Myosin phosphatase Rho-interacting protein OS=Homo sapiens OX=9606 GN=MPRIP PE=1 SV=3
34	84	Q92614|MY18A_HUMAN	154.69	6	8.30E+06	13	13	15	233113	Unconventional myosin-XVIIIa OS=Homo sapiens OX=9606 GN=MYO18A PE=1 SV=3
41	65	P42704|LPPRC_HUMAN	152.12	9	6.41E+06	13	13	13	157904	Leucine-rich PPR motif-containing protein mitochondrial OS=Homo sapiens OX=9606 GN=LRPPRC PE=1 SV=3
57	58	Q9NZB2|F120A_HUMAN	150.02	12	7.14E+06	10	10	10	121888	Constitutive coactivator of PPAR-gamma-like protein 1 OS=Homo sapiens OX=9606 GN=FAM120A PE=1 SV=2
21	108	P46781|RS9_HUMAN	142.44	41	6.55E+07	13	13	22	22591	40S ribosomal protein S9 OS=Homo sapiens OX=9606 GN=RPS9 PE=1 SV=3
54	182	P51636|CAV2_HUMAN	141.41	40	7.54E+07	6	5	11	18291	Caveolin-2 OS=Homo sapiens

										OX=9606 GN=CAV2 PE=1 SV=2
29	76	Q13435|SF3B2_HUMAN	138.17	17	1.35E+07	16	16	19	100228	Splicing factor 3B subunit 2 OS=Homo sapiens OX=9606 GN=SF3B2 PE=1 SV=2
40	49	Q15424|SAFB1_HUMAN	137.31	14	7.72E+06	13	7	14	102642	Scaffold attachment factor B1 OS=Homo sapiens OX=9606 GN=SAFB PE=1 SV=4
56	39	Q6PKG0|LARP1_HUMAN	133.18	9	8.46E+06	9	9	9	123510	La-related protein 1 OS=Homo sapiens OX=9606 GN=LARP1 PE=1 SV=2
64	146	P07910|HNRPC_HUMAN	126.19	21	8.36E+06	6	6	8	33670	Heterogeneous nuclear ribonucleoproteins C1/C2 OS=Homo sapiens OX=9606 GN=HNRNPC PE=1 SV=4
71	166	O14974|MYPT1_HUMAN	124.82	6	2.70E+06	5	5	5	115281	Protein phosphatase 1 regulatory subunit 12A OS=Homo sapiens OX=9606 GN=PPP1R12A PE=1 SV=1
70	77	P60709|ACTB_HUMAN	123.73	21	5.15E+06	7	7	8	41737	Actin cytoplasmic 1 OS=Homo sapiens OX=9606 GN=ACTB PE=1 SV=1
79	167	Q99700|ATX2_HUMAN	122.57	6	3.27E+06	6	6	6	140283	Ataxin-2 OS=Homo sapiens OX=9606 GN=ATXN2 PE=1 SV=2
59	72	P41252|SYIC_HU	120.	8	6.27E	10	10	10	1444	Isoleucine--tRNA

		MAN	67		+06				98	ligase cytoplasmic OS=Homo sapiens OX=9606 GN=IARS1 PE=1 SV=2
63	67	Q14151|SAFB2_HUMAN	117.65	8	7.47E+05	8	2	9	107474	Scaffold attachment factor B2 OS=Homo sapiens OX=9606 GN=SAFB2 PE=1 SV=1
84	171	Q8N163|CCAR2_HUMAN	117.15	7	2.66E+06	6	6	6	102902	Cell cycle and apoptosis regulator protein 2 OS=Homo sapiens OX=9606 GN=CCAR2 PE=1 SV=2
69	118	Q6IQ23|PKHA7_HUMAN	116.69	8	3.23E+06	8	8	8	127135	Pleckstrin homology domain-containing family A member 7 OS=Homo sapiens OX=9606 GN=PLEKHA7 PE=1 SV=2
81	223	Q4VCS5|AMOT_HUMAN	116.14	6	2.15E+06	5	5	5	118085	Angiomotin OS=Homo sapiens OX=9606 GN=AMOT PE=1 SV=1
66	180	P18621|RL17_HUMAN	113.77	35	8.35E+06	7	7	8	21397	60S ribosomal protein L17 OS=Homo sapiens OX=9606 GN=RPL17 PE=1 SV=3
74	144	O75494|SRS10_HUMAN	113.1	13	4.44E+06	5	5	7	31301	Serine/arginine-rich splicing factor 10 OS=Homo sapiens OX=9606 GN=SRSF10 PE=1 SV=1
61	107	P05023|AT1A1_HUMAN	109.2	9	4.01E+06	8	8	8	112896	Sodium/potassium-transporting ATPase subunit

										alpha-1 OS=Homo sapiens OX=9606 GN=ATP1A1 PE=1 SV=1
78	85	P04899|GNAI2_HUMAN	108.07	18	1.64E+06	6	4	6	40451	Guanine nucleotide-binding protein G(i) subunit alpha-2 OS=Homo sapiens OX=9606 GN=GNAI2 PE=1 SV=3
73	187	P62081|RS7_HUMAN	107.47	24	1.33E+07	6	6	7	22127	40S ribosomal protein S7 OS=Homo sapiens OX=9606 GN=RPS7 PE=1 SV=1
76	150	P84103|SRSF3_HUMAN	105.08	21	1.11E+07	4	4	5	19330	Serine/arginine-rich splicing factor 3 OS=Homo sapiens OX=9606 GN=SRSF3 PE=1 SV=1
62	88	P26640|SYVC_HUMAN	101.79	9	4.20E+06	9	9	9	140476	Valine--tRNA ligase OS=Homo sapiens OX=9606 GN=VARS1 PE=1 SV=4
83	156	P35237|SPB6_HUMAN	101.55	18	3.65E+06	6	6	6	42622	Serpin B6 OS=Homo sapiens OX=9606 GN=SERPINB6 PE=1 SV=3
92	183	P51149|RAB7A_HUMAN	99.6	27	2.45E+06	5	5	5	23490	Ras-related protein Rab-7a OS=Homo sapiens OX=9606 GN=RAB7A PE=1 SV=1
80	194	Q15029|U5S1_HUMAN	96.2	6	1.84E+06	5	5	5	109436	116 kDa U5 small nuclear ribonucleoprotein component OS=Homo sapiens

										OX=9606 GN=EFTUD2 PE=1 SV=1
85	211	Q9Y5S9|RBM8A_HUMAN	93.25	26	7.53E+06	5	5	6	19889	RNA-binding protein 8A OS=Homo sapiens OX=9606 GN=RBM8A PE=1 SV=1
72	139	P62995|TRA2B_HUMAN	92.52	23	4.07E+06	7	7	8	33666	Transformer-2 protein homolog beta OS=Homo sapiens OX=9606 GN=TRA2B PE=1 SV=1
94	252	Q86VP6|CAND1_HUMAN	90.27	3	1.07E+06	4	4	4	136375	Cullin-associated NEDD8-dissociated protein 1 OS=Homo sapiens OX=9606 GN=CAND1 PE=1 SV=2
67	168	P62987|RL40_HUMAN	88.27	46	7.06E+06	6	6	7	14728	Ubiquitin-60S ribosomal protein L40 OS=Homo sapiens OX=9606 GN=UBA52 PE=1 SV=2
93	103	P08754|GNAI3_HUMAN	87.59	15	2.76E+06	5	3	5	40532	Guanine nucleotide-binding protein G(i) subunit alpha-3 OS=Homo sapiens OX=9606 GN=GNAI3 PE=1 SV=3
95	201	O75400|PR40A_HUMAN	83.31	4	1.96E+06	4	4	4	108805	Pre-mRNA-processing factor 40 homolog A OS=Homo sapiens OX=9606 GN=PRPF40A PE=1 SV=2
97	277	Q8IXT5|RB12B_HUMAN	82.59	3	1.23E+06	3	3	3	118103	RNA-binding protein 12B OS=Homo sapiens OX=9606

										GN=RBM12B PE=1 SV=2
65	162	P46778|RL21_HUMAN	81.29	27	5.21E+06	7	7	7	18565	60S ribosomal protein L21 OS=Homo sapiens OX=9606 GN=RPL21 PE=1 SV=2
96	75	O75533|SF3B1_HUMAN	80.64	3	2.23E+06	4	4	4	145830	Splicing factor 3B subunit 1 OS=Homo sapiens OX=9606 GN=SF3B1 PE=1 SV=3
88	198	P78332|RBM6_HUMAN	78.42	6	1.69E+06	5	5	5	128644	RNA-binding protein 6 OS=Homo sapiens OX=9606 GN=RBM6 PE=1 SV=5
101	251	P81605|DCD_HUMAN	78.25	23	1.96E+06	3	3	3	11284	Dermcidin OS=Homo sapiens OX=9606 GN=DCD PE=1 SV=2
100	184	Q15366|PCBP2_HUMAN	76.75	10	2.74E+06	3	3	3	38580	Poly(rC)-binding protein 2 OS=Homo sapiens OX=9606 GN=PCBP2 PE=1 SV=1
89	234	O43896|KIF1C_HUMAN	76.71	3	1.67E+06	3	3	4	122947	Kinesin-like protein KIF1C OS=Homo sapiens OX=9606 GN=KIF1C PE=1 SV=3
104	258	Q96J01|THOC3_HUMAN	76.69	8	1.35E+06	3	3	3	38772	THO complex subunit 3 OS=Homo sapiens OX=9606 GN=THOC3 PE=1 SV=1
126	386	Q3MHD2|LSM12_HUMAN	73.98	11	1.39E+06	2	2	2	21701	Protein LSM12 homolog OS=Homo sapiens OX=9606 GN=LSM12

										PE=1 SV=2
103	208	P11142|HSP7C_HUMAN	73.74	6	1.04E+06	3	3	3	70898	Heat shock cognate 71 kDa protein OS=Homo sapiens OX=9606 GN=HSPA8 PE=1 SV=1
98	220	Q9UN81|LORF1_HUMAN	72.91	7	1.90E+06	2	2	3	40056	LINE-1 retrotransposable element ORF1 protein OS=Homo sapiens OX=9606 GN=L1RE1 PE=1 SV=1
87	329	P17661|DESM_HUMAN	72.27	3	1.13E+06	3	1	4	53536	Desmin OS=Homo sapiens OX=9606 GN=DES PE=1 SV=3
107	113	Q9BQE3|TBA1C_HUMAN	70.73	7	1.02E+06	3	3	3	49895	Tubulin alpha-1C chain OS=Homo sapiens OX=9606 GN=TUBA1C PE=1 SV=1
99	259	P49419|AL7A1_HUMAN	70.57	7	1.24E+06	3	3	3	58487	Alpha-aminoadipic semialdehyde dehydrogenase OS=Homo sapiens OX=9606 GN=ALDH7A1 PE=1 SV=5
106	281	P08865|RSSA_HUMAN	69.33	13	1.55E+06	3	3	3	32854	40S ribosomal protein SA OS=Homo sapiens OX=9606 GN=RPSA PE=1 SV=4
129	384	Q13751|LAMB3_HUMAN	66.54	2	6.73E+05	2	2	2	129572	Laminin subunit beta-3 OS=Homo sapiens OX=9606 GN=LAMB3 PE=1 SV=1
108	320	O43684|BUB3_HUMAN	64.68	8	6.66E+05	3	3	3	37155	Mitotic checkpoint protein BUB3 OS=Homo

										sapiens OX=9606 GN=BUB3 PE=1 SV=1
105	190	Q9UKM9|RALY_HUMAN	60.94	10	1.09E+06	3	3	3	32463	RNA-binding protein Raly OS=Homo sapiens OX=9606 GN=RALY PE=1 SV=1
128	148	O15182|CETN3_HUMAN	59.28	11	8.64E+05	2	2	2	19550	Centrin-3 OS=Homo sapiens OX=9606 GN=CETN3 PE=1 SV=2
178	181	Q99873|ANM1_HUMAN	59.15	3	7.72E+05	1	1	1	42462	Protein arginine N-methyltransferase 1 OS=Homo sapiens OX=9606 GN=PRMT1 PE=1 SV=3
109	323	P49756|RBM25_HUMAN	55.84	4	1.36E+06	3	3	3	100186	RNA-binding protein 25 OS=Homo sapiens OX=9606 GN=RBM25 PE=1 SV=3
91	209	P01857|IGHG1_HUMAN	55.57	5	1.40E+07	2	2	4	36106	Immunoglobulin heavy constant gamma 1 OS=Homo sapiens OX=9606 GN=IGHG1 PE=1 SV=1
110	327	Q5M775|CYTSB_HUMAN	52.9	2	8.91E+05	3	3	3	118585	Cytospin-B OS=Homo sapiens OX=9606 GN=SPECC1 PE=1 SV=1
127	302	P61026|RAB10_HUMAN	52.77	12	8.61E+05	2	2	2	22541	Ras-related protein Rab-10 OS=Homo sapiens OX=9606 GN=RAB10 PE=1 SV=1
114	385	Q8IWR0|Z3H7A_HUMAN	52.73	4	7.86E+05	2	2	2	110538	Zinc finger CCCH domain-containing

										protein 7A OS=Homo sapiens OX=9606 GN=ZC3H7A PE=1 SV=1
130	426	P47914|RL29_HUMAN	50.49	14	1.59E+06	2	2	2	17752	60S ribosomal protein L29 OS=Homo sapiens OX=9606 GN=RPL29 PE=1 SV=2
179	480	Q86V48|LUZP1_HUMAN	50.39	1	3.35E+05	1	1	1	120275	Leucine zipper protein 1 OS=Homo sapiens OX=9606 GN=LUZP1 PE=1 SV=2
131	309	Q00839|HNRPU_HUMAN	49.92	3	9.30E+05	2	2	2	90585	Heterogeneous nuclear ribonucleoprotein U OS=Homo sapiens OX=9606 GN=HNRNPU PE=1 SV=6
133	453	Q9Y4L1|HYOU1_HUMAN	47.6	2	6.63E+05	2	2	2	111335	Hypoxia up-regulated protein 1 OS=Homo sapiens OX=9606 GN=HYOU1 PE=1 SV=1
180	145	Q00059|TFAM_HUMAN	47.38	4	4.57E+05	1	1	1	29097	Transcription factor A mitochondrial OS=Homo sapiens OX=9606 GN=TFAM PE=1 SV=1
119	6284	Q8NFJ5|RAI3_HUMAN	46.35	6	1.95E+06	2	2	2	40251	Retinoic acid-induced protein 3 OS=Homo sapiens OX=9606 GN=GPRC5A PE=1 SV=2
164	222	P32969|RL9_HUMAN	45.66	5	3.34E+06	1	1	1	21863	60S ribosomal protein L9 OS=Homo sapiens OX=9606 GN=RPL9 PE=1

										SV=1
132	396	Q9Y6A4|CFA20_HUMAN	45.21	10	6.90E+05	2	2	2	22774	Cilia- and flagella-associated protein 20 OS=Homo sapiens OX=9606 GN=CFAP20 PE=1 SV=1
112	562	Q59GN2|R39L5_HUMAN	43.26	29	2.90E+06	2	1	3	6323	Putative 60S ribosomal protein L39-like 5 OS=Homo sapiens OX=9606 GN=RPL39P5 PE=5 SV=2
113	95	Q86SQ0|PHLB2_HUMAN	42.57	1	3.27E+05	1	1	1	142158	Pleckstrin homology-like domain family B member 2 OS=Homo sapiens OX=9606 GN=PHLDB2 PE=1 SV=2
120	443	P28370|SMCA1_HUMAN	41.57	2	6.49E+05	2	2	2	122605	Probable global transcription activator SNF2L1 OS=Homo sapiens OX=9606 GN=SMARCA1 PE=1 SV=2
157	456	P62861|RS30_HUMAN	41.19	17	7.97E+05	1	1	1	6648	40S ribosomal protein S30 OS=Homo sapiens OX=9606 GN=FAU PE=1 SV=1
121	379	P07477|TRY1_HUMAN	39.13	4	3.46E+06	1	1	1	26558	Trypsin-1 OS=Homo sapiens OX=9606 GN=PRSS1 PE=1 SV=1
181	560	Q9BVC6|TM109_HUMAN	38.91	5	3.19E+05	1	1	1	26210	Transmembrane protein 109 OS=Homo sapiens OX=9606 GN=TMEM109 PE=1 SV=1

86	703	Q12802|AKP13_HUMAN	38.42	0	3.25E+08	2	2	4	307550	A-kinase anchor protein 13 OS=Homo sapiens OX=9606 GN=AKAP13 PE=1 SV=2
182	576	Q13243|SRSF5_HUMAN	38.4	3	3.66E+05	1	1	1	31264	Serine/arginine-rich splicing factor 5 OS=Homo sapiens OX=9606 GN=SRSF5 PE=1 SV=1
183	440	Q5T8P6|RBM26_HUMAN	38.1	1	2.10E+05	1	1	1	113597	RNA-binding protein 26 OS=Homo sapiens OX=9606 GN=RBM26 PE=1 SV=3
158	367	B9A064|IGLL5_HUMAN	36.24	4	8.77E+05	1	1	1	23063	Immunoglobulin lambda-like polypeptide 5 OS=Homo sapiens OX=9606 GN=IGLL5 PE=2 SV=2
156	127	P51991|ROA3_HUMAN	35.5	3	5.20E+05	1	1	1	39595	Heterogeneous nuclear ribonucleoprotein A3 OS=Homo sapiens OX=9606 GN=HNRNPA3 PE=1 SV=2
184	495	P30876|RPB2_HUMAN	34.44	1	1.90E+05	1	1	1	133896	DNA-directed RNA polymerase II subunit RPB2 OS=Homo sapiens OX=9606 GN=POLR2B PE=1 SV=1
185	337	O75694|NU155_HUMAN	33.89	1	2.93E+05	1	1	1	155199	Nuclear pore complex protein Nup155 OS=Homo sapiens OX=9606 GN=NUP155 PE=1 SV=1
165	629	O60232|ZNRD2_HUMAN	30.58	4	4.21E+05	1	1	1	21474	Protein ZNRD2 OS=Homo

										sapiens OX=9606 GN=ZNRD2 PE=1 SV=1
159	6285	O15372|EIF3H_HUMAN	29.63	3	4.95E+05	1	1	1	39930	Eukaryotic translation initiation factor 3 subunit H OS=Homo sapiens OX=9606 GN=EIF3H PE=1 SV=1
163	488	Q8IUD2|RB6I2_HUMAN	28.95	1	3.01E+05	1	1	1	128086	ELKS/Rab6-interacting/CAST family member 1 OS=Homo sapiens OX=9606 GN=ERC1 PE=1 SV=1
166	29	P15924|DESP_HUMAN	28.26	0	1.30E+05	1	1	1	331774	Desmoplakin OS=Homo sapiens OX=9606 GN=DSP PE=1 SV=3
187	616	Q8NI27|THOC2_HUMAN	28.24	1	1.94E+05	1	1	1	182774	THO complex subunit 2 OS=Homo sapiens OX=9606 GN=THOC2 PE=1 SV=2
188	263	P06748|NPM_HUMAN	27.64	3	4.99E+05	1	1	1	32575	Nucleophosmin OS=Homo sapiens OX=9606 GN=NPM1 PE=1 SV=2
161	368	Q58FF7|H90B3_HUMAN	27.18	2	4.57E+05	1	1	1	68325	Putative heat shock protein HSP 90-beta-3 OS=Homo sapiens OX=9606 GN=HSP90AB3P PE=5 SV=1
190	631	Q96Q15|SMG1_HUMAN	26.24	0	4.36E+06	1	1	1	410501	Serine/threonine-protein kinase SMG1 OS=Homo sapiens OX=9606 GN=SMG1 PE=1 SV=3

191	618	Q8IWX8|CHERP_HUMAN	25.52	1	2.60E+05	1	1	1	103702	Calcium homeostasis endoplasmic reticulum protein OS=Homo sapiens OX=9606 GN=CHERP PE=1 SV=3
168	24	P14923|PLAK_HUMAN	25.03	2	9.17E+05	1	1	1	81745	Junction plakoglobin OS=Homo sapiens OX=9606 GN=JUP PE=1 SV=3
160	195	P16615|AT2A2_HUMAN	25.03	1	5.12E+05	1	1	1	114757	Sarcoplasmic/endoplasmic reticulum calcium ATPase 2 OS=Homo sapiens OX=9606 GN=ATP2A2 PE=1 SV=1
162	307	Q14157|UBP2L_HUMAN	24.58	1	3.52E+05	1	1	1	114534	Ubiquitin-associated protein 2-like OS=Homo sapiens OX=9606 GN=UBAP2L PE=1 SV=2
195	765	Q15020|SART3_HUMAN	24.14	1	2.65E+05	1	1	1	109935	Squamous cell carcinoma antigen recognized by T-cells 3 OS=Homo sapiens OX=9606 GN=SART3 PE=1 SV=1
193	493	Q9Y5B9|SP16H_HUMAN	23.59	1	3.89E+05	1	1	1	119914	FACT complex subunit SPT16 OS=Homo sapiens OX=9606 GN=SUPT16H PE=1 SV=1
194	348	Q6NZI2|CAVN1_HUMAN	23.36	2	2.73E+05	1	1	1	43476	Caveolae-associated protein 1 OS=Homo sapiens OX=9606 GN=CAVIN1

										PE=1 SV=1
169	365	O15042|SR140_HUMAN	22.54	1	1.05E+06	1	1	1	118292	U2 snRNP-associated SURP motif-containing protein OS=Homo sapiens OX=9606 GN=U2SURP PE=1 SV=2
196	537	Q8WXE9|STON2_HUMAN	22.27	1	8.71E+05	1	1	1	101165	Stonin-2 OS=Homo sapiens OX=9606 GN=STON2 PE=1 SV=1
197	280	P35251|RFC1_HUMAN	21.76	1	2.17E+05	1	1	1	128254	Replication factor C subunit 1 OS=Homo sapiens OX=9606 GN=RFC1 PE=1 SV=4
199	6323	Q6P4H8|ACKMT_HUMAN	20.34	3	8.25E+05	1	1	1	26110	ATP synthase subunit C lysine N-methyltransferase OS=Homo sapiens OX=9606 GN=ATPSCKMT PE=1 SV=2
170	5104	Q02224|CENPE_HUMAN	20.07	0	2.56E+05	1	1	1	316414	Centromere-associated protein E OS=Homo sapiens OX=9606 GN=CENPE PE=1 SV=2

Table S15. Genes whose predicted expression was associated with NPC risk at P between Bonferroni corrected and False Discovery Rate (FDR) corrected value

Gene	Chr	Start	End	Type[a]	Model[b]	Top eQTL SNP[c]
GGCX	2	85771843	85788670	protein_coding	GTEx.Esophagus_Mucosa-MetaXcan	-
MTMR12	5	32227100	32313093	protein_coding	GTEx.Esophagus_Mucosa-MetaXcan	-
HLA-A	6	29909037	29913661	protein_coding	GTEx.Esophagus_Mucosa-FUSION	rs2524005
PPP1R11	6	30034486	30038110	protein_coding	GTEx.Esophagus_Mucosa-FUSION	rs385492
TRIM31	6	30070674	30080867	protein_coding	GTEx.Esophagus_Mucosa-MetaXcan	-
ABCF1	6	30539170	30564956	protein_coding	GTEx.Esophagus_Mucosa-MetaXcan	-
VARS2	6	30881982	30894236	protein_coding	GTEx.Esophagus_Mucosa-MetaXcan	-
					GTEx.Esophagus_Mucosa-FUSION	rs1265048
HCG22	6	31021227	31027667	lncRNA	GTEx.Esophagus_Mucosa-MetaXcan	-
PSORS1C1	6	31082527	31107869	protein_coding	GTEx.Esophagus_Mucosa-MetaXcan	-
NFKBIL1	6	31514647	31526606	protein_coding	GTEx.Esophagus_Mucosa-MetaXcan	-
C6orf48	6	31802385	31807543	lncRNA	GTEx.Esophagus_Mucosa-FUSION	rs2293861
C4A	6	31949834	31970458	protein_coding	GTEx.Esophagus_Mucosa-MetaXcan	-
PRRT1	6	32116136	32120860	protein_coding	GTEx.Esophagus_Mucosa-FUSION	rs9271055
HLA-DRB5	6	32485130	32498064	protein_coding	GTEx.Esophagus_Mucosa-MetaXcan	-
HLA-DRB1	6	32546546	32557625	protein_coding	GTEx.Esophagus_Mucosa-MetaXcan	-
HLA-DPA1	6	33032346	33048552	protein_coding	GTEx.Esophagus_Mucosa-MetaXcan	-
UQCC2	6	33662070	33679504	protein_coding	GTEx.Esophagus_Mucosa-MetaXcan	-
ESYT2	7	158523686	158622944	protein_coding	GTEx.Esophagus_Mucosa-MetaXcan	-
					GTEx.Esophagus_Mucosa-FUSION	rs896860
GALT	9	34638130	34651032	protein_coding	GTEx.Esophagus_Mucosa-MetaXcan	-
NOL8	9	95059640	95087918	protein_coding	GTEx.Esophagus_Mucosa-FUSION	rs11794346
INO80E	16	30007036	30017114	protein_coding	GTEx.Esophagus_Mucosa-MetaXcan	-
YPEL3	16	30103635	30108236	protein_coding	GTEx.Esophagus_Mucosa-MetaXcan	-
SEPTIN1	16	30389454	30407312	protein_coding	GTEx.Esophagus_Mucosa-FUSION	rs8050812
TTLL9	20	30458505	30532766	protein_coding	GTEx.Esophagus_Mucosa-MetaXcan	-

a lncRNA: long non-coding RNAs.

b GTEx.Esophagus_Mucosa-MetaXcan: The models building using the GTEx esophagus mucosa tissue dataset computed in MetaXcan software;

GTEx.Esophagus_Mucosa-FUSION: The models building using the GTEx esophagus mucosa tissue dataset computed in FUSION software.

c Variant most significantly associated with the gene expression from FUSION result; -: not available from MetaXcan result.

Table S16. List of specific primers.

Primer Names	Sequences (5′-3′)
VAMP8-q-F	AATGATCGTGTGCGGAACCT
VAMP8-q-R	TGAAGTGCTCAGATGTGGCT
CCND1-q-F	GCTGCGAAGTGGAAACCATC
CCND1-q-R	CCTCCTTCTGCACACATTTGAA
CDK2-q-F	CCAGGAGTTACTTCTATGCCTGA
CDK2-q-R	TTCATCCAGGGGAGGTACAAC
IL1B-q-F	ATGATGGCTTATTACAGTGGCAA
IL1B-q-R	GTCGGAGATTCGTAGCTGGA
MMP1-q-F	AAAATTACACGCCAGATTTGCC
MMP1-q-R	GGTGTGACATTACTCCAGAGTTG
MMP9-q-F	TGTACCGCTATGGTTACACTCG
MMP9-q-R	GGCAGGGACAGTTGCTTCT
ACTB-q-F	GGCGGCACCACCATGTACCCT
ACTB-q-R	AGGGGCCGGACTCGTCATACT
VAMP8-CDS-F	CCGGAATTCATTCACTTACTGACCGGCCT
VAMP8-CDS-R	GTCGCGGCCGCTGGGAGAGGTTCCCTGTTAC
VAMP8-3′UTR-F	CCGCTCGAGGTAACAGGGAACCTCTCCCACCTGC
VAMP8-3′UTR-R	GTCGCGGCCGCATAGGGATACTGAAAGGTCTTTATTACCC
rs1058588-Mut-F	CCCACCTGCCCTTCTTTTCAGGGACAACCCTC
rs1058588-Mut-R	GAGGGTTGTCCCTGAAAAGAAGGGCAGGTGGG
VAMP8-3′UTR-Mut-F	CTCCCACCTGCAACGCTGTCAAGGGACAACCC
VAMP8-3′UTR-Mut-R	GGGTTGTCCCTTGACAGCGTTGCAGGTGGGAG

Figure 3A

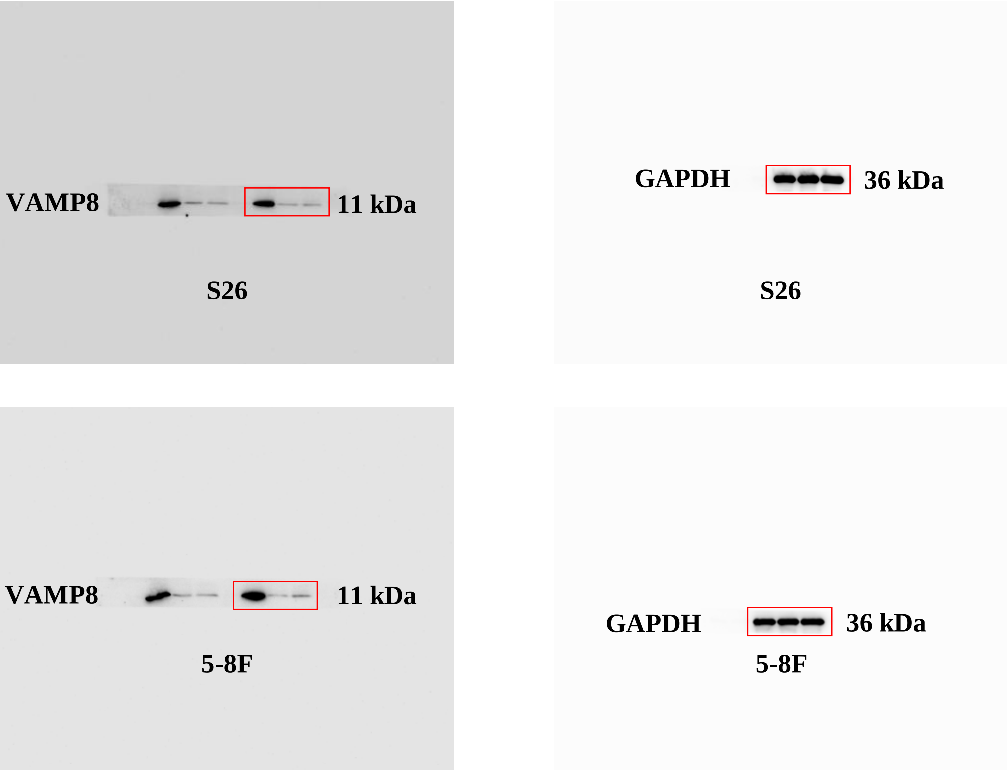

Figure 5A

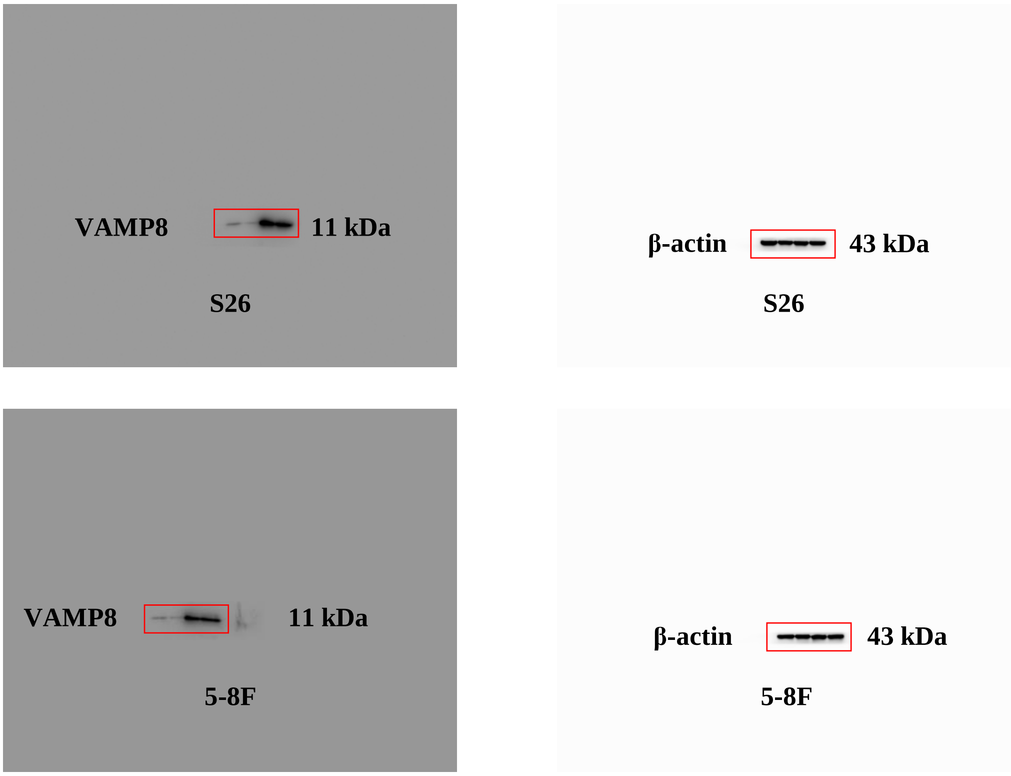

Figure 6D

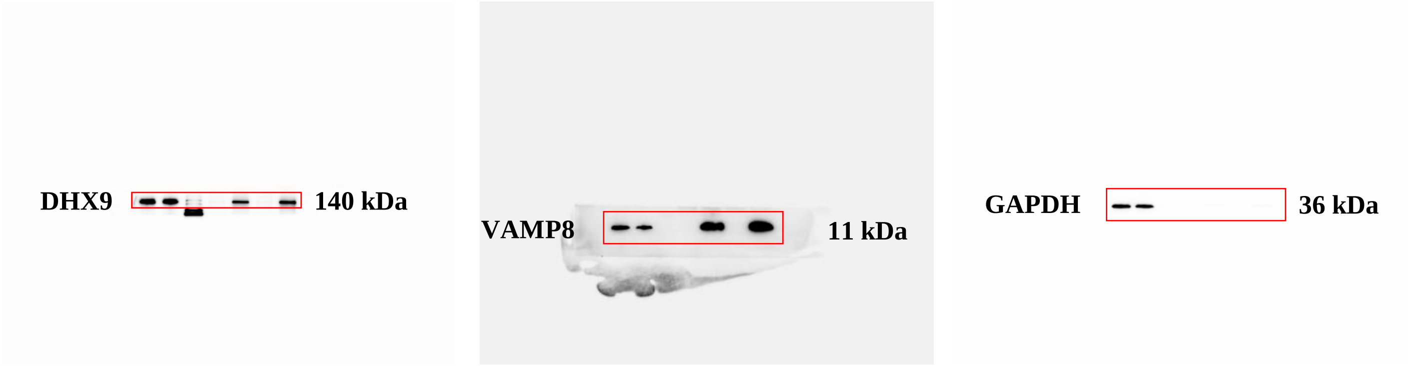

Figure 6F

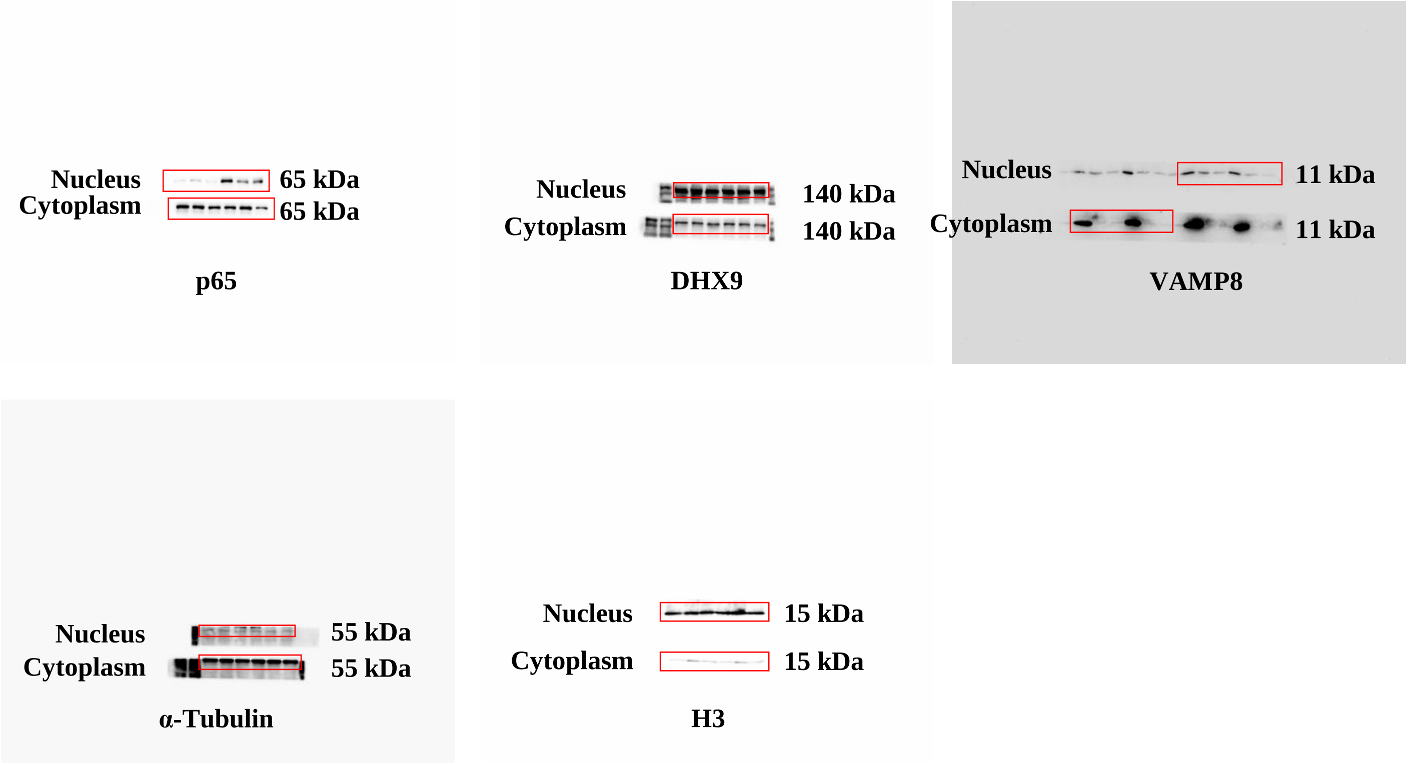

Figure 6G

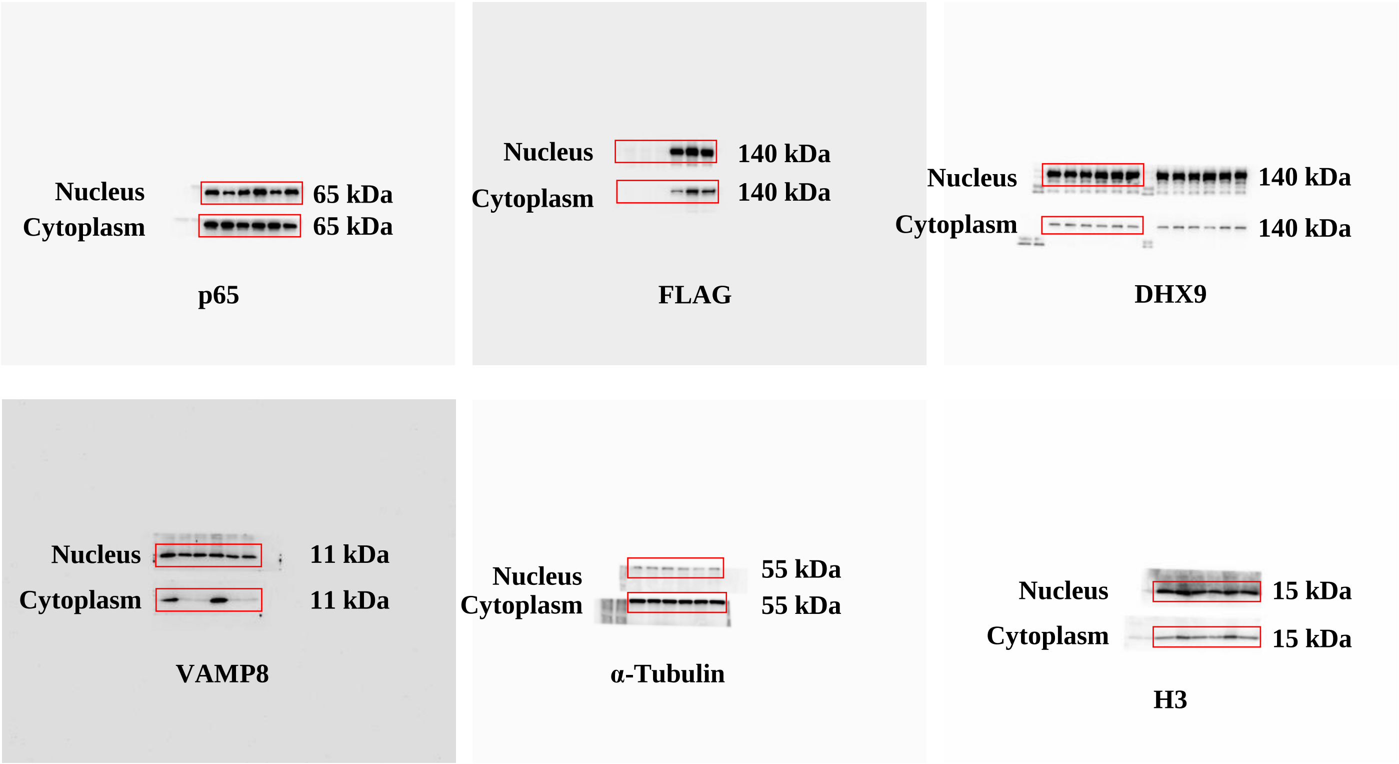
Nucleus
Cytoplasm
65 kDa
65 kDa
p65
Nucleus
Cytoplasm
140 kDa
140 kDa
FLAG
Nucleus
Cytoplasm
140 kDa
140 kDa
DHX9
Nucleus
Cytoplasm
11 kDa
11 kDa
VAMP8
Nucleus
Cytoplasm
55 kDa
55 kDa
α-Tubulin
Nucleus
Cytoplasm
15 kDa
15 kDa
H3

Figure S5C

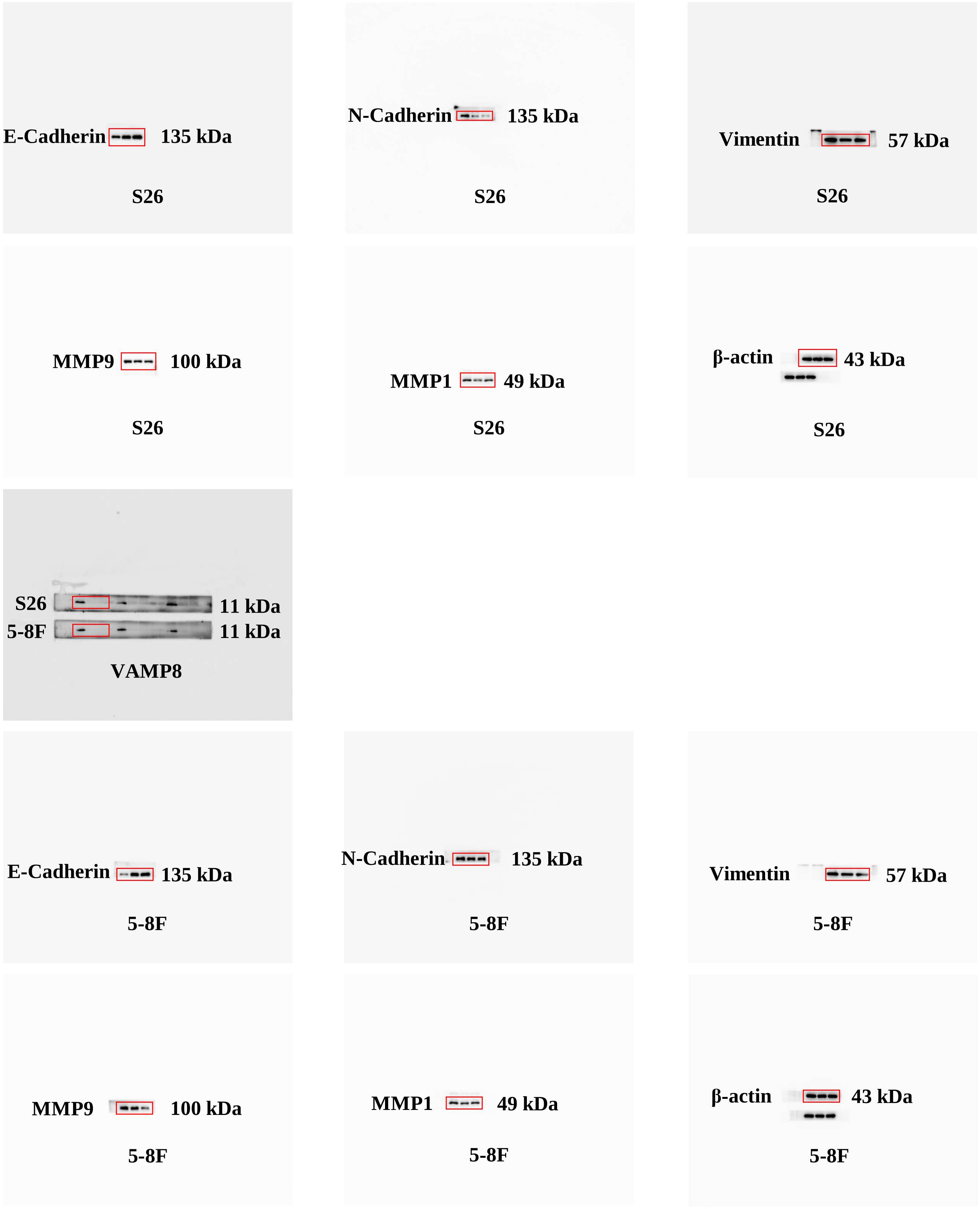

E-Cadherin
135 kDa
S26
N-Cadherin
135 kDa
S26
Vimentin
57 kDa
S26
MMP9
100 kDa
S26
MMP1
49 kDa
S26
β-actin
43 kDa
S26
S26
5-8F
11 kDa
11 kDa
VAMP8
E-Cadherin
135 kDa
5-8F
N-Cadherin
135 kDa
5-8F
Vimentin
57 kDa
5-8F
MMP9
100 kDa
5-8F
MMP1
49 kDa
5-8F
β-actin
43 kDa
5-8F

Figure S6F

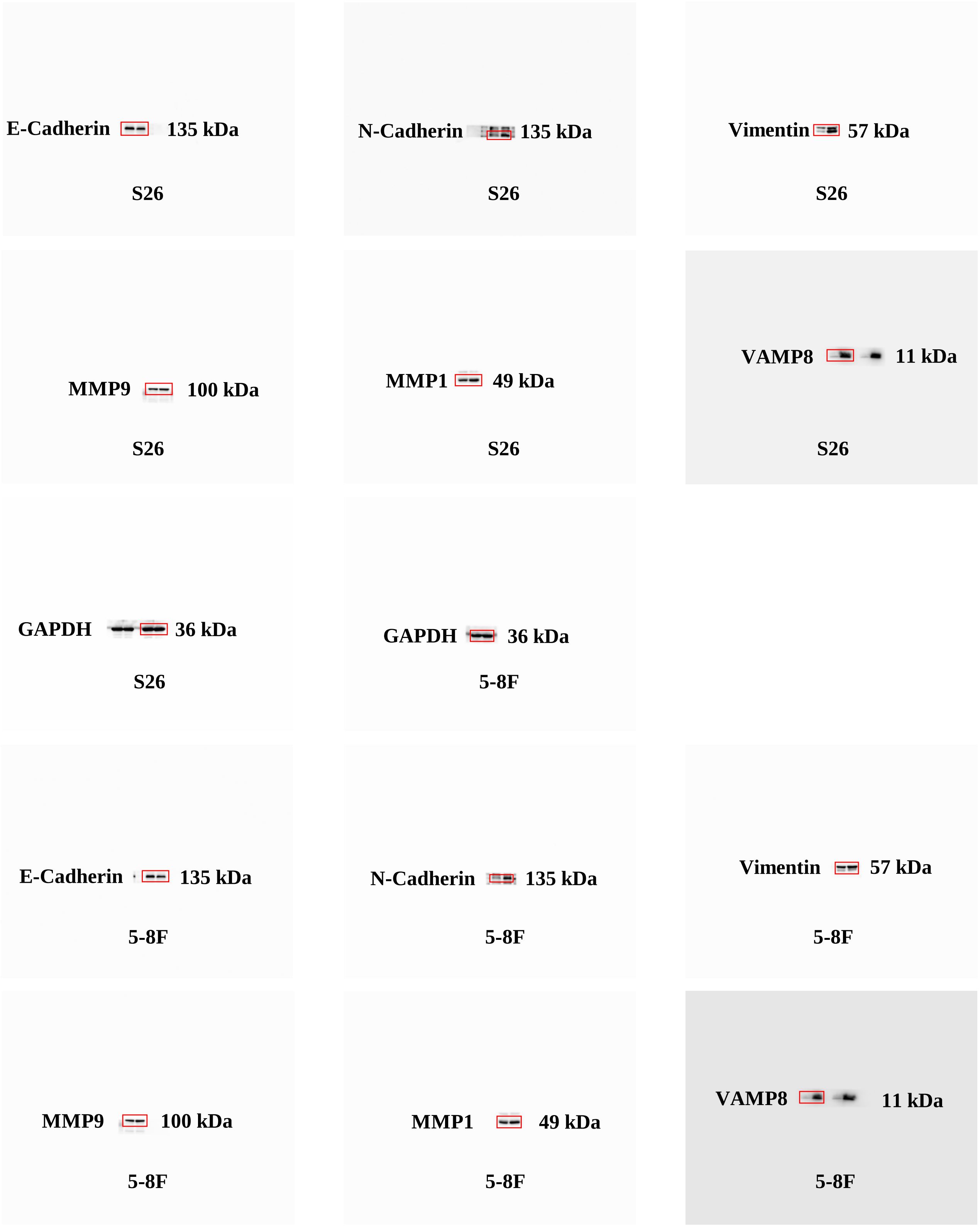
E-Cadherin 135 kDa
S26
N-Cadherin 135 kDa
S26
Vimentin 57 kDa
S26
MMP9 100 kDa
S26
MMP1 49 kDa
S26
VAMP8 11 kDa
S26
GAPDH 36 kDa
S26
GAPDH 36 kDa
5-8F
E-Cadherin 135 kDa
5-8F
N-Cadherin 135 kDa
5-8F
Vimentin 57 kDa
5-8F
MMP9 100 kDa
5-8F
MMP1 49 kDa
5-8F
VAMP8 11 kDa
5-8F

Figure S7C

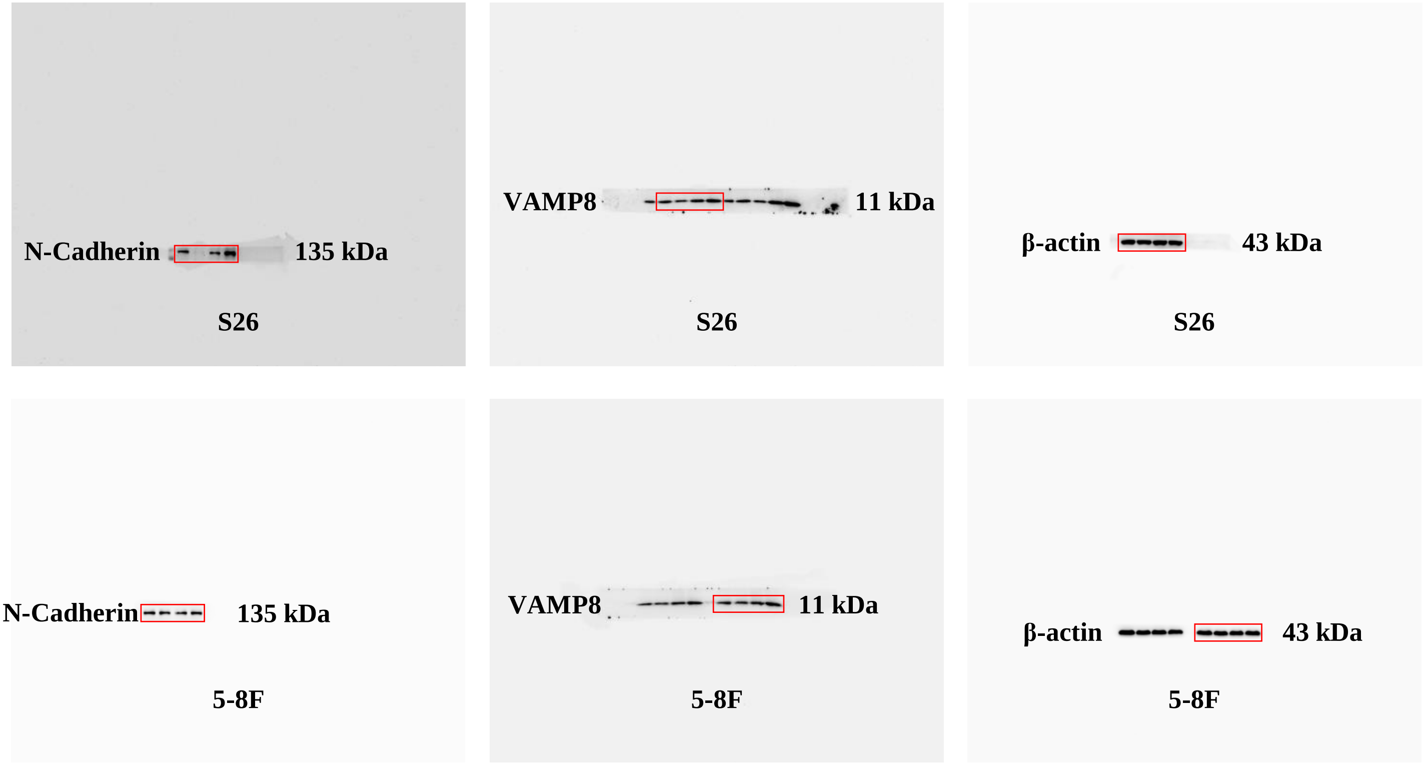
N-Cadherin
135 kDa
S26
VAMP8
11 kDa
S26
β-actin
43 kDa
S26
N-Cadherin
135 kDa
5-8F
VAMP8
11 kDa
5-8F
β-actin
43 kDa
5-8F

Figure S9D

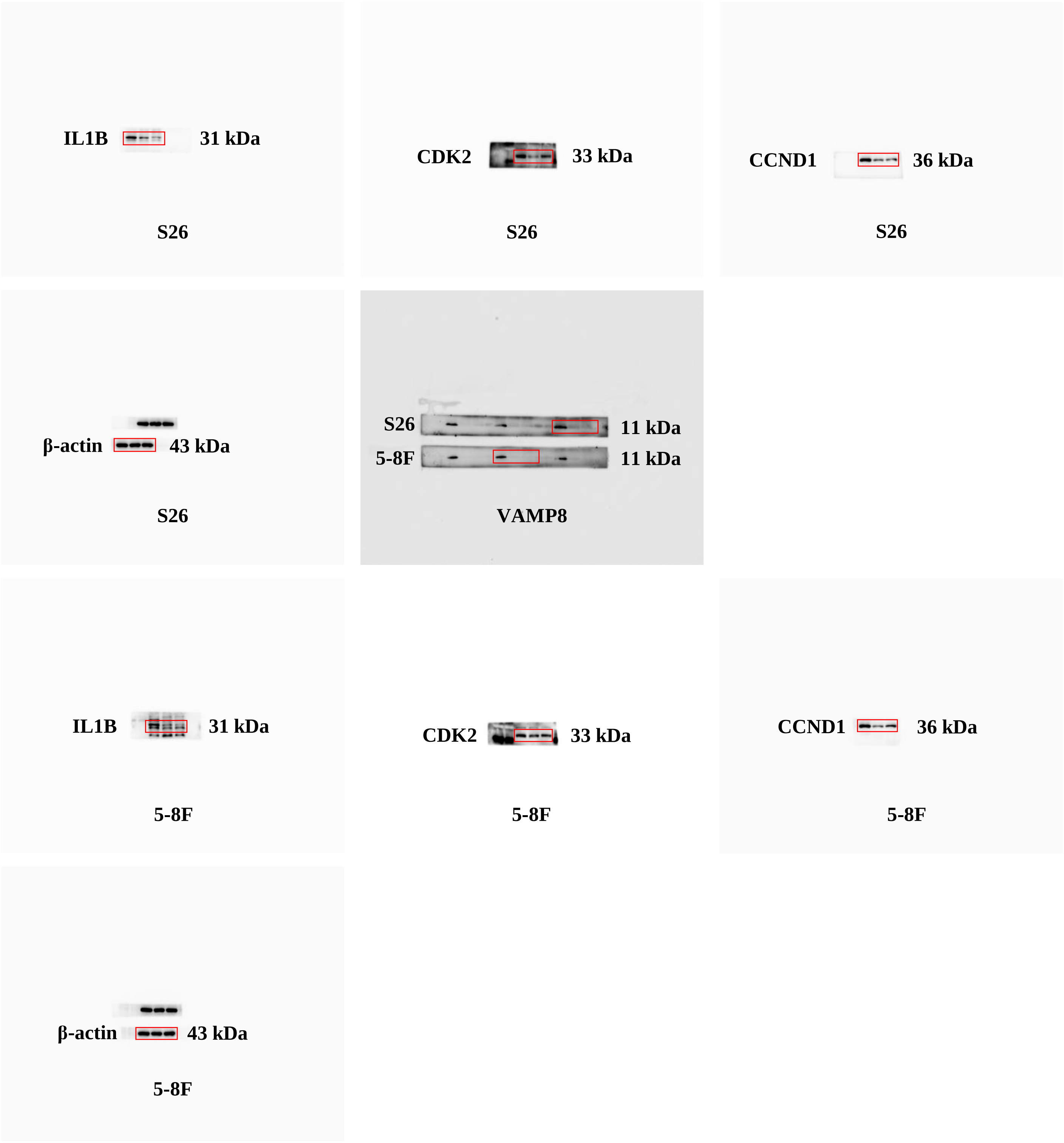
IL1B
31 kDa
S26
CDK2
33 kDa
S26
CCND1
36 kDa
S26
β-actin
43 kDa
S26
S26
11 kDa
5-8F
11 kDa
VAMP8
IL1B
31 kDa
5-8F
CDK2
33 kDa
5-8F
CCND1
36 kDa
5-8F
β-actin
43 kDa
5-8F

Figure S10D-E

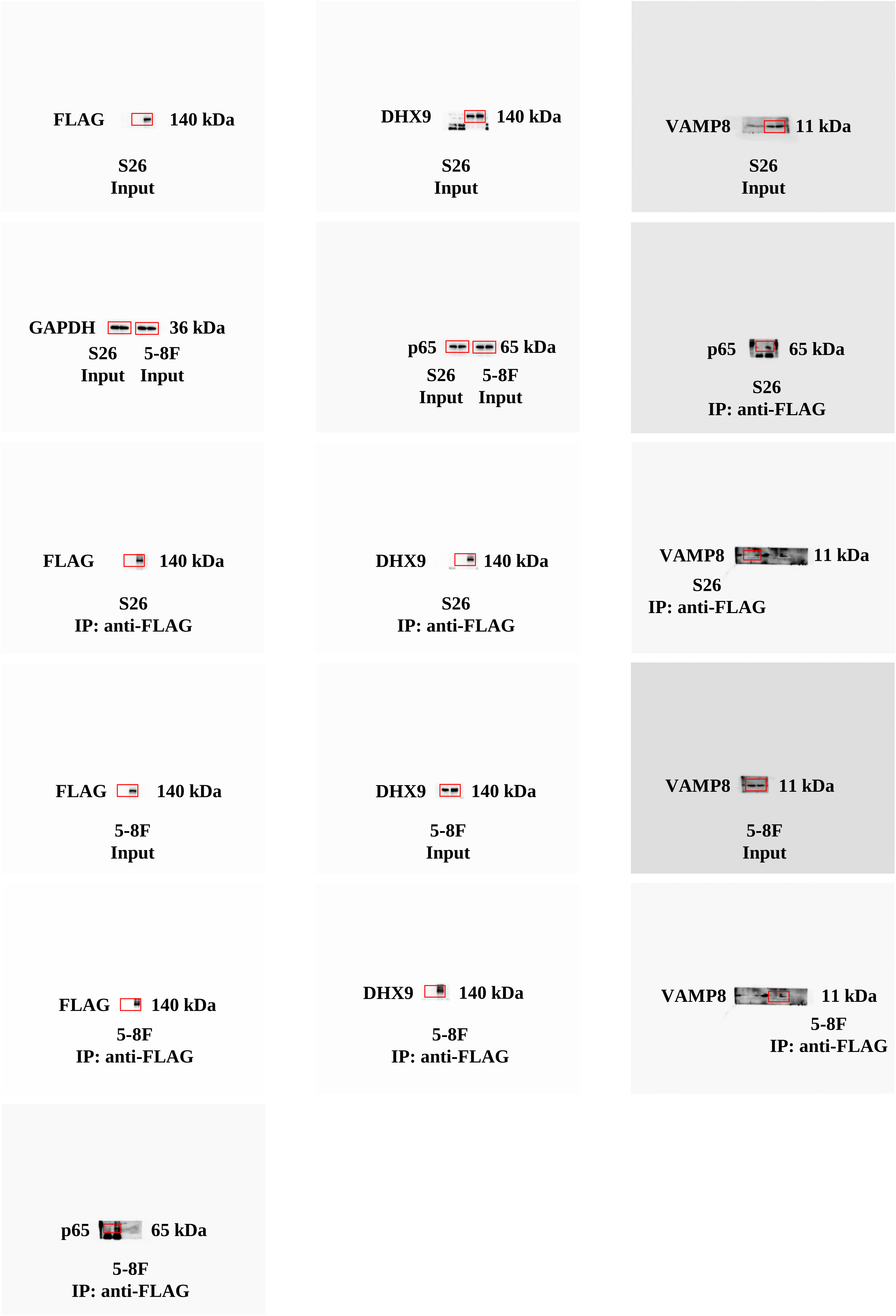

Figure S10F

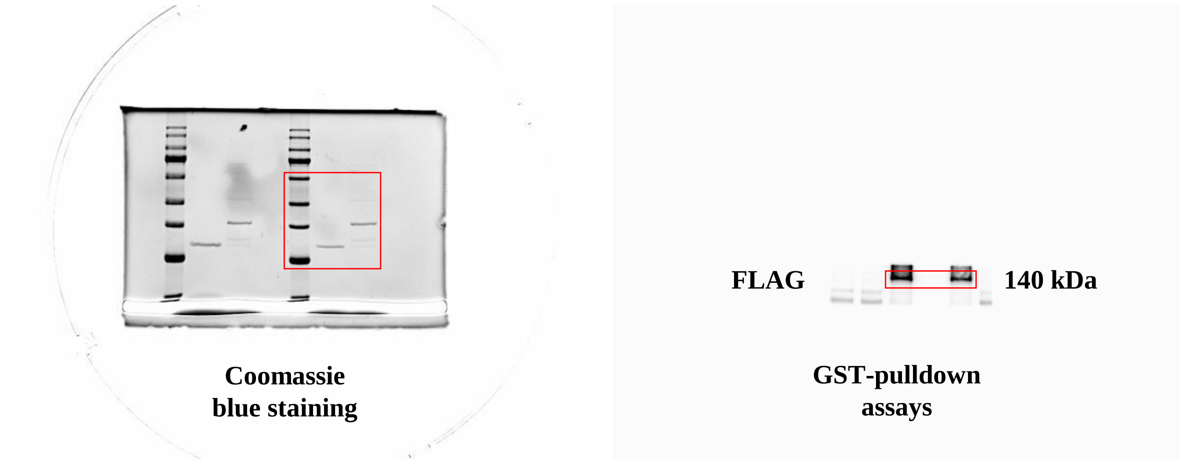

Figure S11B

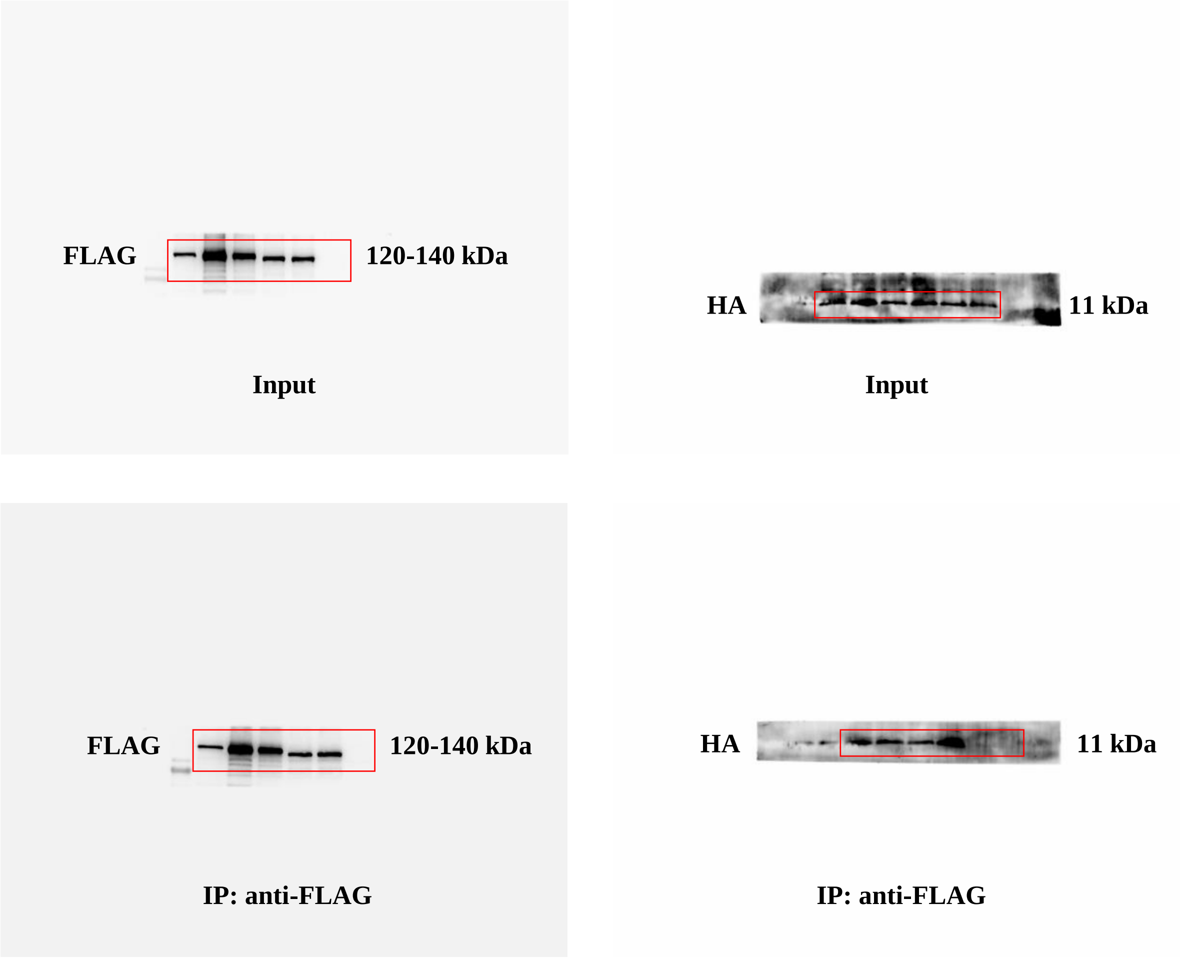

Supplement: Supplementary file 1 — Supporting Information [file ADVS-12-2412580-s001.pdf]
